# Dipropylamine for 9-Fluorenylmethyloxycarbonyl (Fmoc) Deprotection with Reduced Aspartimide Formation in Solid-Phase Peptide Synthesis

**DOI:** 10.1021/acsomega.2c07861

**Published:** 2023-01-23

**Authors:** Hippolyte Personne, Thissa N. Siriwardena, Sacha Javor, Jean-Louis Reymond

**Affiliations:** † Department of Chemistry, Biochemistry and Pharmaceutical Sciences, University of Bern, Freiestrasse 3, CH-3012 Bern, Switzerland; ‡ Shanghai Space Peptides Pharmaceutical Co., Ltd., Shanghai 201210, China

## Abstract

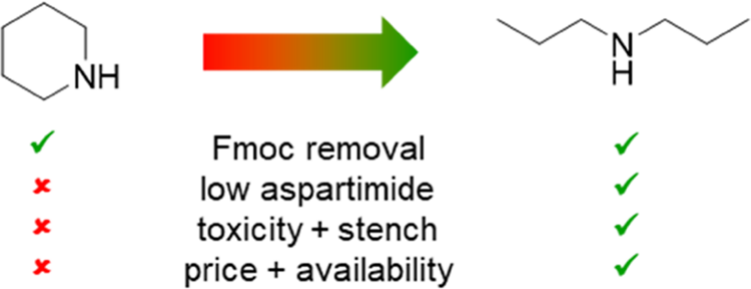

Herein, we report dipropylamine (DPA) as a fluorenylmethyloxycarbonyl (Fmoc) deprotection reagent to strongly reduce aspartimide formation compared to piperidine (PPR) in high-temperature (60 °C) solid-phase peptide synthesis (SPPS). In contrast to PPR, DPA is readily available, inexpensive, low toxicity, and nonstench. DPA also provides good yields in SPPS of non-aspartimide-prone peptides and peptide dendrimers.

## Introduction

**Figure 1:**
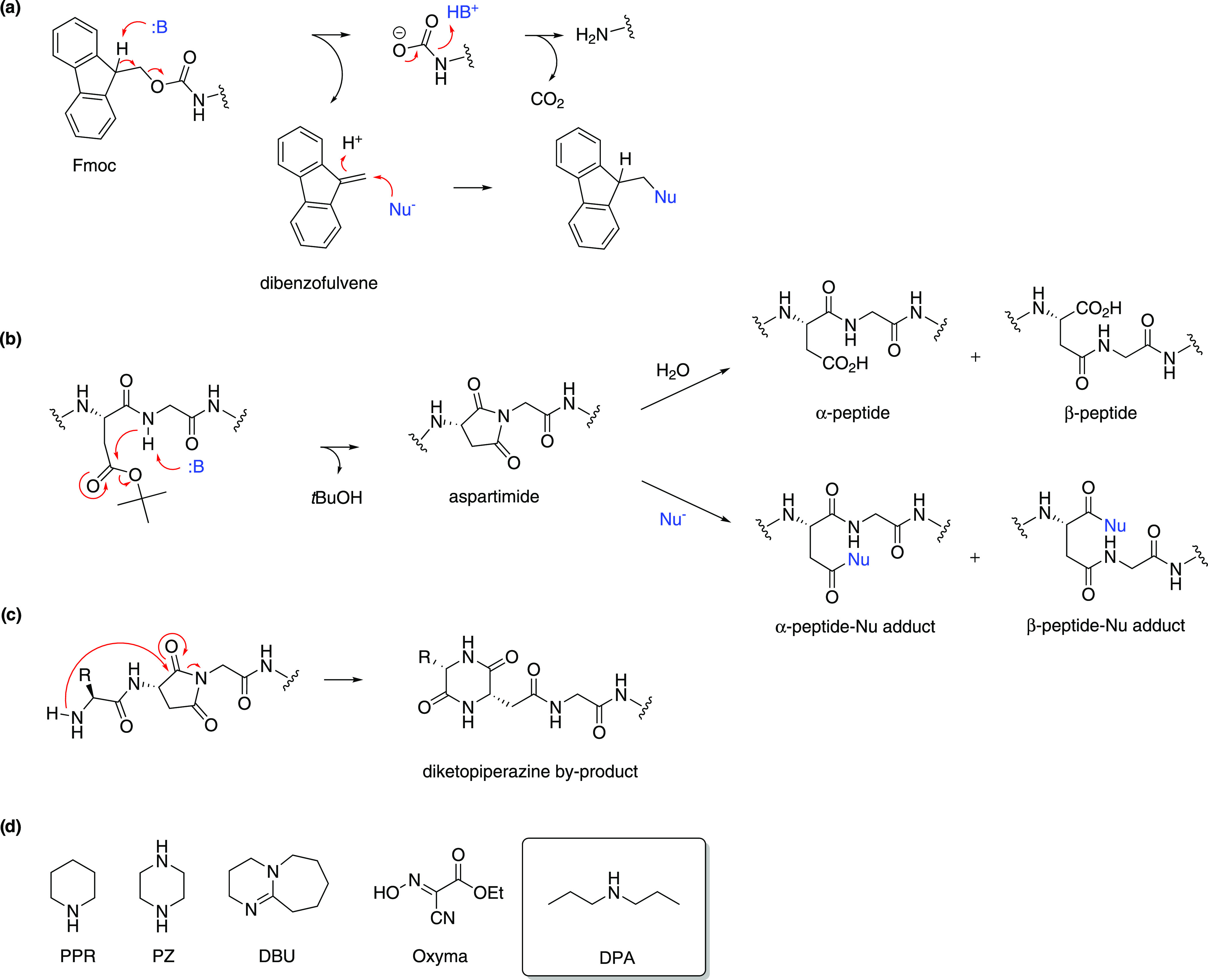
(a) Mechanism of Fmoc deprotection and trapping of dibenzofulvene. (b) Mechanism of aspartimide formation, its hydrolysis to α- or β-peptides, and its ring-opening by nucleophilic attack to α- or β-peptide-nucleophile adducts. (c) Mechanism of diketopiperazine byproduct formation. (d) Structural formulae of reagents used for Fmoc removal.

Solid-phase peptide synthesis (SPPS) with fluorenylmethyloxycarbonyl (Fmoc) as the α-amino protecting group for amino acid building blocks is currently the dominant synthesis method for peptide research and manufacturing. The Fmoc protecting group is removed by a base, which triggers β-elimination of carbamic acid followed by the formation of an adduct with the dibenzofulvene (DBF) byproduct (Figure 1a) with a nucleophile.^1^ Piperidine (PPR) is currently the most widely used Fmoc removal reagent. However, in addition to its toxicity and regulation, PPR induces the formation of aspartimide in some aspartic acid-containing sequences, which can hydrolyze to α- or β-peptides, react again with the nucleophile to form peptide-base derivatives, or induce an intramolecular formation of the terminating diketopiperazine byproduct by nucleophilic attack of the deprotected amino group of the next amino acid (Figure 1b,c).^2−6^

PPR can be replaced by a mixture of piperazine (PZ) as the nucleophile and 1,8-diazabicyclo[5.4.0]undec-7-ene (DBU) as the base^7^ or simply DBU without added nucleophile (Figure 1d);^8^ however, DBU is quite expensive and produces a considerable amount of aspartimide for aspartimide-prone sequences. One can also add weak acids such as formic acid or ethyl cyanohydroxyiminoacetate (Oxyma) to temper the basicity of the PPR solution to reduce aspartimide formation.^9^ However, this still does not solve the cost, stench, and availability issues of PPR.

Alternative bases^10−13^ or aspartate side-chain protecting groups^14−17^ have been reported to overcome the limitations of PPR or PZ/DBU; however, none of them combines low cost and convenient use with low aspartimide and good yields. Here, we searched for PPR alternatives in the context of a high-temperature (60 °C) SPPS protocol with Oxyma and *N*,*N*′-diisopropylcarbodiimide (DIC) as coupling reagents^18^ and *N*,*N*-dimethylformamide (DMF) as the solvent, which in our hands work excellently for a variety of peptides, cyclic peptides, and peptide dendrimers.^19−22^ We noted that diethylamine (DEA, b.p. 55 °C) has been used for Fmoc removal in process-scale SPPS.^23^ We therefore set out to test the less-volatile dipropylamine (DPA, b.p. 110 °C), which is advantageously cheaper than both DEA and dibutylamine (DBA).

## Results and Discussion

### Aspartimide-Prone Sequences

Due to the lower basicity of DPA (p*K*_a_ = 10.9) compared to PPR (p*K*_a_ = 11.1), we investigated whether DPA might solve the issue of aspartimide formation in SPPS of aspartimide-prone sequences using the prototypical test case hexapeptide **1** (VKDGYI) and compared it to other Fmoc deprotecting reagents.

Aspartimide formation is catalyzed by relatively strong bases, and lowering the basicity allows one to reduce the formation of this side product. For instance, the crude product of hexapeptide **1** synthesized using PPR for Fmoc removal contained 17% aspartimide. The results were even worse with DBU, which is a stronger base than PPR. In this case, purity was only 52% due to 25% aspartimide and 23% byproducts. Furthermore, using PZ/DBU only gave byproducts (Table 1).

**Table 1:** Screening of Deprotection Conditions for Low Aspartimide Formation in Hexapeptide **1** (VKDGYI)

Fmoc deprotection reagent^a^	temperature (°C)	crude yield^b^ (%)	product ratio^c^(%)
20% PPR	60	47	83/17/0
2% DBU	60	26	52/25/23
5% PZ + 2% DBU	60	0	0/0/100
25% DPA	60	53	96/4/0
25% DEA	60	46	89/8/3
25% DBA	60	52	93/4/3
20% PPR + 0.5 M Oxyma	60	17	93/6/1
5% PZ + 2% DBU + 0.5 M Oxyma	60	22	86/13/1
20% DPA + 0.5 M Oxyma	60	45	93/6/1
20% PPR	90	28	70/20/10
25% DPA	90	34	78/11/11

a PPR, piperidine; PZ, piperazine; DBU, 1,8-diazabicyclo[5.4.0]undec-7-ene; DPA, dipropylamine; DEA, diethylamine; DBA, dibutylamine. Percentages (%) are in w/v in the case of PZ and in v/v otherwise.

b Crude yield is calculated as follows: (crude mass/molecular weight of desired peptide)/(mass of resin × resin loading) × % of desired product content in crude.

c Product ratio was determined by LC analysis and is given as follows: % desired product/% aspartimide/% other byproducts. The main byproduct observed was the diketopiperazine terminating sequence (mass 576.3 Da); see HRMS data in the Supporting Information.

By contrast, the crude product of hexapeptide **1** synthesized using DPA for Fmoc removal was 96% pure and contained only 4% aspartimide as the only detectable byproduct. We obtained similar SPPS yields with hexapeptide **1** using the secondary aliphatic amines DEA and DBA for Fmoc removal, although some byproducts were also observed, whereas sterically hindered diisobutylamine (DIBA) only gave byproducts (Table S1). Furthermore, we did not detect any trace of **1β** (VKD(β)GYI), the β-peptide analogue of hexapeptide **1**, which can potentially be formed by reopening of the aspartimide, upon ^1^H NMR analysis in comparison with an independently synthesized β-peptide sample (Supporting Information Figures S1 and S2).

Note that aspartimide formation was strongly reduced by adding 0.5 M Oxyma or hydroxybenzotriazole (HOBt) as weak acids to PPR (Tables 1 and S1), reproducing published results.^9^ Adding Oxyma also allowed one to obtain the product with PZ/DBU; however, adding Oxyma to DPA did not reduce aspartimide formation further compared to DPA alone. When tested at 90 °C, SPPS of hexapeptide **1** with PPR gave 20% aspartimide in the crude and only 11% with DPA for Fmoc removal, showing that DPA was also superior to PPR in terms of low aspartimide at high temperatures (Table 1).

We further tested DPA on other aspartimide-prone sequences, hexapeptide **2**^6^ and analogues of hexapeptides **1** and **2** with various Asp-X motives (Table 2). Aspartimide content was fourfold lower with DPA in contrast to PPR in the case of hexapeptide **2**. Substitution of glycine by arginine showed again a reduction of aspartimide formation and a yield increase using DPA with hexapeptides **3** and **4**. Substitution by a cysteine (hexapeptide **5**) gave similar results with both bases, and almost no aspartimide was observed for the substitution with alanine (hexapeptide **6**).

**Table 2:** Aspartimide Formation in Other Aspartimide-Prone Peptide Sequences

Cpd. sequence^a^	Fmoc deprotection reagent^b^	crude yield^c^(%)	product ratio^d^ (%)
hexapeptide **2** GDGAKF	20% PPR	41	67/32/1
	25% DPA	49	84/8/8
hexapeptide **3** VKDRYI	20% PPR	40	84/8/8
	25% DPA	43	90/4/6
hexapeptide **4** GDRAKF	20% PPR	51	96/3/1
	25% DPA	63	99/0/1
hexapeptide **5** VKDCYI	20% PPR	53	90/5/5
	25% DPA	48	88/4/8
hexapeptide **6** VKDAYI	20% PPR	55	97/1/2
	25% DPA	51	96/1/3

a One-letter code for amino acids. C-termini are carboxamide.

b SPPS was carried at 60 °C. PPR, piperidine; DPA, dipropylamine. Percentages (%) are in v/v.

c Crude yield is calculated as explained in Table 1.

d Product ratio was determined by LC analysis and is given as follows: % desired product/% aspartimide/% other byproducts.

Finally, we performed the synthesis of hexapeptide **7** bearing a glutamic instead of the aspartic acid to investigate glutarimide formation, but none was observed for all of the conditions tested (Supporting Information Table S1).

### Fmoc Removal by DPA in Solution

**Figure 2:**
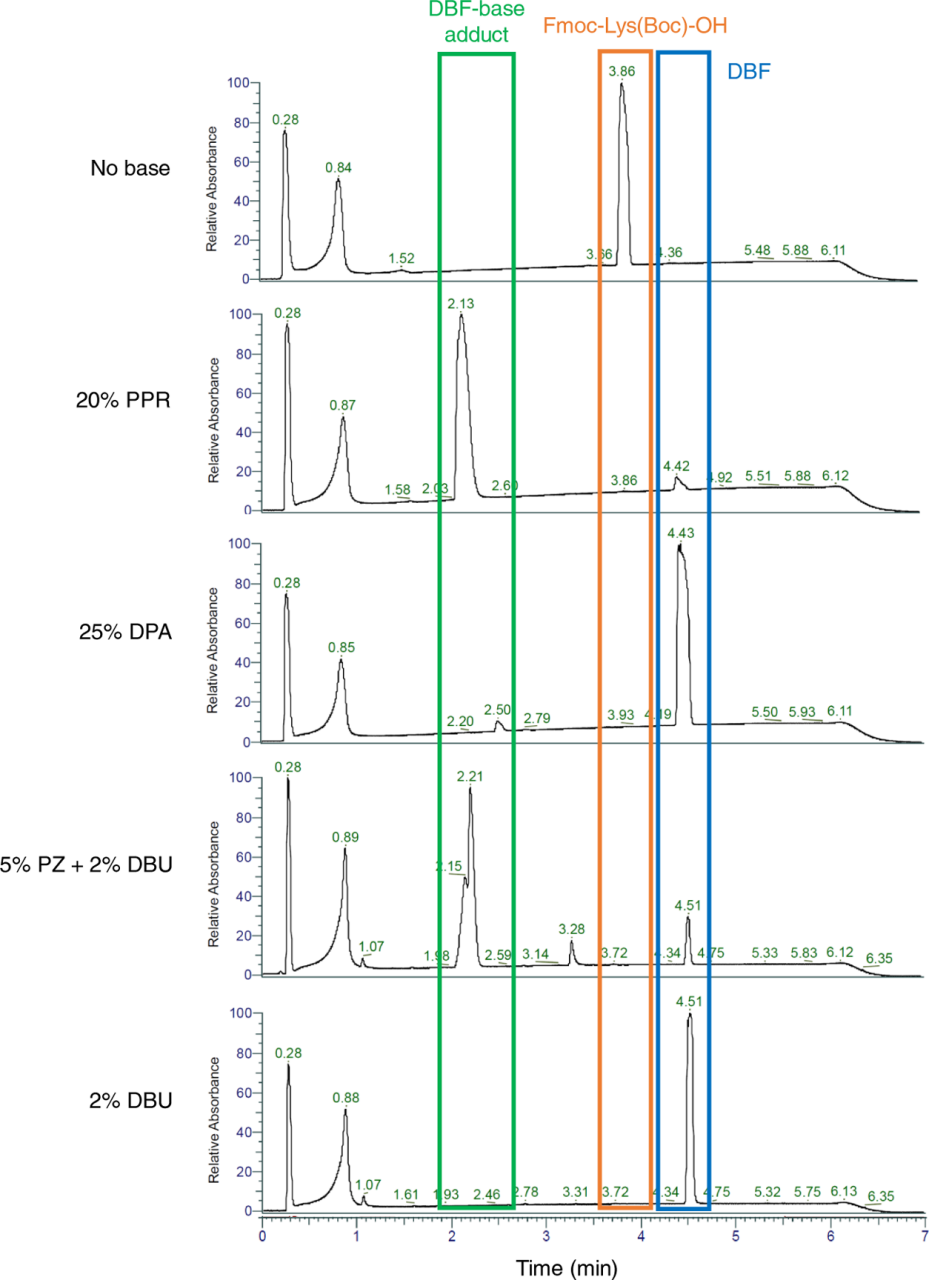
Liquid-phase Fmoc deprotection of Fmoc-Lys(Boc)-OH in DMF at room temperature for 30 min analyzed by HPLC (λ = 214 nm). Fmoc-Lys(Boc)-OH (*t*_R_ = 3.86 min) and (b) Fmoc-Phe-OH (*t*_R_ = 3.85 min). DBF-PPR adduct (*t*_R_ = 2.13 min), DBF-DPA adduct (*t*_R_ = 2. 50 min), PZ-DBF adduct (*t*_R_ = 2.21 min), and DBF (*t*_R_ = 4.42–4.51 min) can be observed. DBF, dibenzofulvene; PPR, piperidine; DPA, dipropylamine; PZ, piperazine; DBU, 1,8-diazabicyclo[5.4.0]undec-7-ene.

Following the deprotection of the amino acid building block Fmoc-Lys(Boc)-OH in solution using high-performance liquid chromatography (HPLC) confirmed the formation of DBF as well as adduct formation with the base, according to the general deprotection mechanism (Figure 1). We found that 25% DPA in DMF rapidly released DBF with only a small amount of adduct formation (Figure 2; see Figure S3 for examples with Fmoc-Phe-OH and Fmoc-PEG-OH). Similar effects occurred with DEA and DBA, consistent with hexapeptide **1** syntheses data (Table 1), while the hindered secondary amines diisopropylamine (DIPA) and DIBA gave only partial deprotection (Supporting Information Figure S3). By comparison, PZ/DBU and PPR led to the maximum adduct formation, while DBU produced no adduct.

### Fmoc Deprotection with Linear Peptides

We next tested our DPA protocol with the linear peptide drugs Afamelanotide (13 residues) and Bivalirudin (20 residues). In both cases, DPA performed well, independent of sequence length, with similar purities compared to PPR. We also observed excellent yields with both bases for Bivalirudin. We further tested Bivalirudin synthesis at 90 °C, which provided the desired product for both PPR and DPA, however with a comparable reduction in yield compared to the 60 °C protocol (Table 3).

**Table 3:** Syntheses of Peptide Drugs Using Piperidine and Dipropylamine as Fmoc Removal Agents

Cpd. sequence^a^	Fmoc deprotection reagent^b^	temperature (°C)	crude yield^c^(%)	crude purity^d^ (%)	isolated yield^e^ (%)
Afamelanotide	20% PPR	60	70	46	17
Ac-SYSNleEHfRWGKPV	25% DPA	60	45	50	10
Bivalirudin	20% PPR	60	n.d.	77	46
fPRPGGGGNGDFEEIPEEYL-OH	25% DPA	60	n.d.	77	39
	20% PPR	90	6.6	28	n.d.
	25% DPA	90	4.6	25	n.d.

a One-letter code for amino acids, D-amino acids in lower case. C-terminus is carboxamide for Afamelanotide and carboxyl for Bivalirudin. Ac, acetyl group; Nle, norleucine.

b PPR, piperidine; DPA, dipropylamine. Percentages (%) are in v/v.

c Crude yield is calculated as explained in Table 1.

d The crude product after resin cleavage was precipitated, washed, dried, lyophilized and analyzed by analytical HPLC to determine the percentage of desired product and other byproducts.

e Isolated yields were calculated after preparative RP-HPLC purification according to the amount of resin and its indicated loading. n.d., not determined.

### Fmoc Deprotection in Peptide Dendrimers

We finally investigated Fmoc deprotection with DPA and other deprotection agents for SPPS of the peptide dendrimer **G1KL** at 60 °C, a prototypical first-generation peptide dendrimer (Table 4).^24^ Dendrimer SPPS is an interesting test case for Fmoc deprotection because it requires simultaneous Fmoc removal at the lysine α- and ε-amino groups at the branching point. SPPS with PPR gave good crude purity (90%) and a crude yield of 73%. Crude purity was higher with PZ/DBU (97%), but the yield was much lower (26%). Using 25% DPA for Fmoc removal provided slightly lower crude purities (88%) and yields (65%) than with PPR, but reducing the DPA to 20% gave lower crude purities (78%) and yields (35%). Interestingly, adding 1% DBU to 20% DPA did not increase yields, but adding 1% DBU to the sterically hindered DIPA, which itself was unable to remove Fmoc, gave crude purities and crude yields comparable to 20% DPA, suggesting that DBU alone was triggering Fmoc removal with DIPA. DPA gave lower but still good crude yields compared to PPR even at room temperature in the case of the second-generation dendrimer **G2KL**.^25^ For the third-generation dendrimer **G3KL**,^24^ both high- and room-temperatures syntheses gave very poor yields with DPA, while PPR worked well at both temperatures. Note that DBU alone gave yields comparable to PPR in this case showing that the difficulty of DPA with peptide dendrimer synthesis is not related to the lack of DBF adduct formation.

**Table 4:** Syntheses of Peptide Dendrimers with Various Fmoc Deprotection Conditions

Cpd. sequence^a^	Fmoc deprotection reagent^b^	temperature^c^ (°C)	crude yield^d^ (%)	crude purity^e^(%)
**G1KL** (KL)_2_*K*KL	20% PPR	60	73	90
	5% PZ + 2% DBU	60	26	97
	20% DPA	60	35	78
	25% DPA	60	65	88
	20% DPA + 1% DBU	60	34	85
	20% DIPA	60	0	0
	20% DIPA + 1% DBU	60	36	86
**G2KL** (KL)_4_(*K*KL)_2_*K*KL	20% PPR	r.t.	65	79
	5% PZ + 2% DBU	r.t.	54	74
	20% DPA	r.t.	42	82
	25% DPA	r.t.	46	80
**G3KL** (KL)_8_(*K*KL)_4_(*K*KL)_2_*K*KL	20% PPR	60	47	74
	20% PPR	r.t.	41	78
	25% DPA	60	n/a^f^	n/a^f^
	25% DPA	r.t.	12	29
	2% DBU	60	48	70

a One-letter code for amino acids, *K* indicates branching l-lysine. C-termini are carboxamide.

b PPR, piperidine; PZ, piperazine; DBU, 1,8-diazabicyclo[5.4.0]undec-7-ene; DPA, dipropylamine; DIPA, diisopropylamine. Percentages (%) are in w/v in the case of PZ and in v/v otherwise.

c r.t., room temperature.

d Crude yield is calculated as explained in Table 1.

e The crude product after resin cleavage was precipitated, washed, dried, lyophilized and analyzed by analytical HPLC to determine the percentage of desired product and other byproducts.

f Not applicable. Peak integration was not possible in the case of **G3KL**, 25% DPA, 60 °C due to byproducts/impurities in the crude, but traces of desired compounds were observed by HRMS (see the Supporting Information).

## Conclusions

In summary, the experiments above documenting 56 individual SPPS runs with 11 different peptides (Tables 1, 2, 3, 4 and Supporting Information Figure S1) provide strong evidence that DPA can be used as a Fmoc removal reagent in high-temperature SPPS. The key application for DPA is clearly the case of aspartimide-prone sequences, in which the formation of aspartimide and related byproducts is considerably reduced, and yields are substantially increased compared to PPR. Although generally lower yielding than PPR for challenging syntheses, DPA gave reasonable purities and yields for therapeutic linear peptides and first- and second-generation peptide dendrimers. Furthermore, DPA is unregulated, nonstench, and much cheaper than PPR.

## Material and Methods

### Material and Reagents

DMF (*N*,*N*-dimethylformamide) was purchased from Thommen-Furler AG; Oxyma Pure (hydroxyiminocyanoacetic acid ethyl ester) was purchased from SENN AG; DIC (*N*,*N*′-diisopropyl carbodiimide) was purchased from Iris BIOTECH GMBH; piperidine was purchased from Acros Organics; piperazine, butanol, and DBU (1,8-diazabicyclo[5.4.0]undec-7-ene) were purchased from Alfa Aesar; dipropylamine, diisopropylamine, diethylamine, dibutylamine, diisobutylamine, DMAP (4-dimethlyaminopyridine), HOBt (hydroxybenzotriazole), DIPEA (*N*,*N*-diisopropylethylamine), and DODT (2,2′-(ethylenedioxy)diethanethiol) were purchased from Sigma Aldrich; and triisopropylsilane and TFA (trifluoroacetic acid) were purchased from Fluorochem Ltd. For amino acid, Fmoc-Nle-OH was purchased from Iris BIOTECH GMBH, Fmoc-Asp-OtBu and Fmoc-Glu-OtBu were purchased from Novabiochem, and all of the other amino acids were obtained from Shanghai Space Peptides Pharmaceuticals Co., Ltd. Chemicals were used as supplied, and solvents were of technical grade. Amino acids were used as the following derivatives: Fmoc-Leu-OH, Fmoc-Lys(Boc)-OH, Fmoc-Lys(Fmoc)-OH, Fmoc-Val-OH, Fmoc-Asp(*^t^*Bu)-OH, Fmoc-Asp-O^*t*^Bu, Fmoc-Glu(*^t^*Bu)-OH, Fmoc-Glu-OtBu, Fmoc-Gly-OH, Fmoc-Tyr(tBu)-OH, Fmoc-Ile-OH, Fmoc-Ser-OH, Fmoc-Nle-OH, Fmoc-His(Trt)-OH, Fmoc-D-Phe-OH, Fmoc-Phe-OH, Fmoc-Arg(Pbf)-OH, Fmoc-Trp(Boc)-OH, Fmoc-Pro-OH, Fmoc-Cys(Trt)-OH, and Fmoc-Asn(Trt)-OH. Rink Amide AM LL resin was purchased from Novabiochem. Wang resin was purchased from Iris BIOTECH GMBH.

Analytical RP-HPLC was performed with an Ultimate 3000 Rapid Separation liquid chromatography-mass spectrometry (LC-MS) system (DAD-3000RS diode array detector) using an Acclaim RSLC 120 C18 column (2.2 μm, 120 Å, 3 mm × 50 mm, flow 1.2 mL/min) from Dionex. Data recording and processing were done with the Dionex Chromeleon Management System Version 6.80. All RP-HPLC were using HPLC-grade acetonitrile and Milli-Q deionized water. The elution solutions were as follows: A: Milli-Q deionized water containing 0.05% TFA; D: Milli-Q deionized water/acetonitrile (10:90, v/v) containing 0.05% TFA. Preparative RP-HPLC was performed with a Waters automatic Prep LC Controller System containing the four following modules: Waters 2489 ultraviolet/visible (UV/vis) detector, Waters 2545 pump, Waters Fraction Collector III, and Waters 2707 Autosampler. A Dr. Maisch GmbH Reprospher column (C18-DE, 100 mm × 30 mm, particle size 5 μm, pore size 100 Å, flow rate 40 mL/min) was used. Compounds were detected by UV absorption at 214 nm using a Waters 248 tunable absorbance detector. Data recording and processing were performed with Waters ChromScope version 1.40 from Waters Corporation. All RP-HPLC were using HPLC-grade acetonitrile and Milli-Q deionized water. The elution solutions were as follows: A Milli-Q deionized water containing 0.1% TFA; D Milli-Q deionized water/acetonitrile (10:90, v/v) containing 0.1% TFA. MS spectra, recorded on a Thermo Scientific LTQ OrbitrapXL, were provided by the MS analytical service of the Department of Chemistry, Biochemistry and Pharmaceutical Sciences at the University of Bern (group of PD Dr. Stefan Schürch).

### Solid-Phase Peptide Synthesis of Linear Peptides

All linear peptides were synthesized using standard 9-fluorenylmethoxycarbonyl (Fmoc) solid-phase peptide synthesis under nitrogen bubbling. All peptides were synthesized using the Rink Amide LL resin (0.26–0.29 mmol/g) except for Bivalirudin, for which Wang resin (1.2 mmol/g) was used. The resin was first deprotected twice for 1 and 4 min using the corresponding deprotection cocktail. For each amino acid, a double coupling was performed (2 × 8 min) using for each coupling 3 mL of 0.2 M of the corresponding Fmoc protected amino acid in DMF, 1.5 mL of 0.5 M Oxyma in DMF, and 2 mL of 0.5 M DIC in DMF. Double deprotection steps (1 and 4 min) were achieved using the corresponding deprotection solution.

For Bivalirudin, the first amino acid coupling was performed with the addition of DMAP (0.2 equiv).

For Afamelanotide, the acetylation of the N-terminus was performed on beads using a solution of 775 μL of acetic anhydride and 500 μL of DIPEA in 5 mL of DMF (twice 30 min at room temperature).

For syntheses at 90 °C, coupling times were 2 × 4 min and deprotection times were 0.5 and 2.5 min.

### Solid-Phase Peptide Synthesis of **G1KL**

All peptide dendrimers were synthesized using standard 9-fluorenylmethoxycarbonyl (Fmoc) solid-phase peptide synthesis under nitrogen bubbling using the Rink amide LL resin (0.26–0.29 mmol/g). Branching points consisted of Fmoc-Lys(Fmoc)-OH to obtain two free amines (α and ε) after Fmoc deprotection. Syntheses were performed as described above.

### Solid-Phase Peptide Synthesis of **G2KL**

Syntheses of **G2KL** were performed at room temperature with the same reagents as described above in stirred syringes. Double deprotections were performed for 2 × 10 min. Double coupling was performed for 2 × 1 h for the three first amino acids and the first generation, and a triple coupling was performed (3 × 1 h) for the second-generation residues.

### Solid-Phase Peptide Synthesis of **G3KL**

For syntheses performed at 60 °C, double deprotection was performed for 1 and 4 min and double coupling was performed for 2 × 8 min for the three first amino acids and the first generation. For the second generation, triple deprotection (1, 2, and 4 min) and a quadruple coupling (4 × 8 min) were performed. From the last branching lysine, quadruple deprotection (2, 4, 2, and 4 min) and seven couplings of 8 min were performed.

Syntheses at room temperature were performed with the same reagents as described above in stirred syringes. Double deprotections were performed for 2 × 10 min. For the three first amino acids and the first generation, double coupling was performed for 2 × 1 h. For the second generation, a triple coupling was performed for 3 × 1 h. For the last generation (two last amino acids), quintuple coupling was performed for 5 × 1 h.

### Cleavage from Resin

After the SPPS, peptide dendrimers were cleaved from the resin at room temperature using 7 mL of a mixture of trifluoroacetic acid/triisopropylsilane/mQ water (TFA/TIS/H_2_O) with the corresponding ratios of 94/5/1, except for hexapeptide **5**, for which a 7 mL TFA/TIS /DODT/H_2_O mixture was used with the corresponding ratios 94/2.5/2.5/1 for three hours. Peptides were then precipitated using approximately 25 mL of cold terbutylmethyl ether and centrifuged for 10 min at 4400 rpm. The supernatant was removed, and peptides were dried with argon before lyophilization and/or purification and LC-MS/high-resolution mass spectrometry (HRMS) analyses. All peptides were obtained as TFA salts.

### Fmoc Deprotection in Solution

A total of 50 mg of Fmoc-Lys(Boc)-OH, Fmoc-Phe-OH, or Fmoc-PEG-OH was dissolved in the corresponding deprotection condition in a total volume of 500 μL. Deprotection conditions used in DMF were 20% v/v piperidine, 25% v/v dipropylamine, 5% w/v piperazine + 2% v/v DBU, 2% v/v DBU, 25% v/v dipropylamine + 3% w/v piperazine, 25% v/v diethylamine, 25% v/v diisopropylamine, and 25% diisobutylamine. Reaction mixtures were stirred for 30 min at room temperature. After the reaction and for each condition, 10 μL was diluted in MeCN for a final volume of 1 mL. All samples were analyzed by analytical RP-HPLC-MS using solvents B (100% mQ water + 0.1% formic acid) and C (90% MeCN + 10% mQ water + 0.1% formic acid) with a gradient 100% B to 100% C in 5 min.

### ^1^H NMR Data Acquisition

^1^H NMR spectra were recorded on a Bruker Avance 300 spectrometer (300 MHz) at room temperature. Peptides analyzed by ^1^H NMR were purified using preparative RP-HPLC prior to data acquisition. Spectra analyses were performed using MestReNova v14.2.1. See the Supporting Information for measured spectra.

## Supporting Information Available

Extended data on SPPS yields with various Fmoc deprotection conditions (Tables S1); 1H NMR spectra of peptides **1** and **1β** (Figures S1 and S2); quantification of Fmoc deprotection and DBF formation in solution by LC/MS (Figure S3); and analytical HPLC and HRMS data for all peptides (PDF)
