# Supplementary material for: Dipropylamine for 9-Fluorenylmethyloxycarbonyl (Fmoc) Deprotection with Reduced Aspartimide Formation in Solid-Phase Peptide Synthesis
Source: ACS Omega. 2023 Jan 23;8(5):5050–6. doi: 10.1021/acsomega.2c07861 (PMC9910063; doi:10.1021/acsomega.2c07861)
Supplement: Supplementary file 1 — ao2c07861_si_001.pdf [file ao2c07861_si_001.pdf]

# Supporting information

# Dipropylamine for 9-fluorenylmethyloxycarbonyl (Fmoc) deprotection with reduced aspartimide formation in solid-phase peptide synthesis

Hippolyte Personne[†,1], Thissa N. Siriwardena[†,1,2], Sacha Javor[1] and Jean-Louis Reymond*[1]

[1]Department of Chemistry and Biochemistry, University of Bern, Freiestrasse 3, CH-3012 Bern, Switzerland, e-mail: jean-louis.reymond@unibe.ch

[2]Shanghai Space Peptides Pharmaceutical Co., Ltd. Shanghai 201210, China

[†]These authors contributed equally to this work.

## Table of Contents

**1. Supplementary tables and figures** .......... 2

**Table S1** .......... 2

**Figure S1** .......... 3

**Figure S2** .......... 4

**Figure S3** .......... 6

**2. Compound characterization** .......... 7

2.1 Hexapeptide **1** (VKDGYI) .......... 7

2.2 Hexapeptide **1β (**VKD(β)GYI) .......... 27

2.3 Hexapeptide **2** (GDGAKF) .......... 29

2.4 Hexapeptide **3** (VKDRYI) .......... 32

2.5 Hexapeptide **4** (GDRAKF) .......... 35

2.6 Hexapeptide **5** (VKDCYI) .......... 38

2.7 Hexapeptide **6** (VKDAYI) .......... 41

2.8 Hexapeptide **7** (VKEGYI) .......... 44

2.9 **Afamelanotide** (Ac-SYSNleEHfRWGKPV-$NH_2$) .......... 49

2.10 **Bivaluridin** (fPRPGGGGNGDFEEIPEEYL-OH) .......... 54

2.11 **G1KL** ($(KL)_2$*K*KL) .......... 63

2.12 **G2KL** ($(KL)_4$($K$KL$)_2$*K*KL) .......... 71

2.13 **G3KL** ($(KL)_8$($K$KL$)_4$($K$KL$)_2$*K*KL) .......... 80

# 1. Supplementary tables and figures

**Table S1**: Extended SPPS yields of peptide dendrimers and linear peptides using various Fmoc deprotection conditions.

| **Cpd.**<br>Sequence [a)] | **Deprotection condition** [b)] | **Crude purity** [c)] **%** | **Crude yield** [d)] **%** | **Isolated yield** [f)] **%** | **MS calc/obs (g/mol)** |
|---|---|---|---|---|---|
| Hexapeptide **1**<br>VKDGYI | 20% PPR, 60 °C | 83 (17 / 0) | 46.6 | 4.5 | 693.39/693.39 |
| | 20% PPR + 0.25 M Oxyma, 60 °C | 93 (7 / 0) | 22.1 | n.d. | 693.39/693.39 |
| | 20% PPR + 0.5 M Oxyma, 60 °C | 93 (6 / 1) | 16.8 | n.d. | 693.39/693.39 |
| | 5% PZ + 2% DBU, 60 °C | 0 (0 / 100) | 0 | n.d. | 693.39/- |
| | 5% PZ + 2% DBU + 0.25 M HOBt, 60 °C | 56 (27 / 17) | 9.2 | n.d. | 693.39/693.39 |
| | 5% PZ + 2% DBU + 0.5 M HOBt, 60 °C | 94 (5 / 1) | 10.4 | n.d. | 693.39/693.39 |
| | 5% PZ + 2% DBU + 0.25 M Oxyma, 60 °C | 76 (22 / 2) | 16.3 | n.d. | 693.39/693.39 |
| | 5% PZ + 2% DBU + 0.5 M Oxyma, 60 °C | 86 (13 / 1) | 21.8 | n.d. | 693.39/693.39 |
| | 2% DBU, 60 °C | 52 (25 / 23) | 25.7 | n.d. | 693.39/693.39 |
| | 20% DPA, 60 °C | 95 (5 / 0) | 49.3 | n.d. | 693.39/693.39 |
| | 20% DPA + 0.5 M Oxyma, 60 °C | 93 (6 / 1) | 45.4 | n.d. | 693.39/693.39 |
| | 25% DPA, 60 °C | 96 (4 / 0) | 52.9 | 16.0 | 693.39/693.39 |
| | 25% DEA, 50 °C | 86 (7 / 7) | 43.4 | n.d. | 693.39/693.39 |
| | 25% DEA, 60 °C | 89 (8 / 3) | 45.7 | n.d. | 693.39/693.39 |
| | 25% DBA, 60 °C | 93 (4 / 3) | 52.1 | n.d. | 693.39/693.39 |
| | 25% DIBA, 60 °C | 0 (0 / 100) | 0 | n.d. | 693.39/- |
| | 20% PPR, 90 °C | 70 (20 / 10) | 28.4 | n.d. | 693.39/693.39 |
| | 25% DPA, 90 °C | 78 (11 / 11) | 33.5 | n.d. | 693.39/693.39 |
| Hexapeptide **2**<br>GDGAKF | 20% PPR, 60 °C | 67 (32 / 1) | 40.9 | n.d. | 593.30/593.30 |
| | 25% DPA, 60 °C | 84 (8 / 8) | 49.2 | n.d. | 593.30/593.30 |
| Hexapeptide **3**<br>VKDRYI | 20% PPR, 60 °C | 84 (8 / 8) | 40.3 | n.d. | 792.47/792.47 |
| | 25% DPA, 60 °C | 90 (4 / 6) | 43.4 | n.d. | 792.47/792.47 |
| Hexapeptide **4**<br>GDRAKF | 20% PPR, 60 °C | 96 (3 / 1) | 50.6 | n.d. | 693.36/693.39 |
| | 25% DPA, 60 °C | 99 (0 / 1) | 62.5 | n.d. | 693.36/693.39 |
| Hexapeptide **5**<br>VKDCYI | 20% PPR, 60 °C | 90 (5 / 5) | 53.1 | n.d. | 739.37/739.38 |
| | 25% DPA, 60 °C | 88 (4 / 8) | 48.0 | n.d. | 739.37/739.38 |
| Hexapeptide **6**<br>VKDAYI | 20% PPR, 60 °C | 97 (1 / 2) | 54.7 | n.d. | 707.40/707.41 |
| | 25% DPA, 60 °C | 96 (1 / 3) | 51.3 | n.d. | 707.40/707.41 |
| Hexapeptide **7**<br>VKEGYI | 20% PPR, 60 °C | 98 (0 / 2) | 47.7 | n.d. | 707.40/707.41 |
| | 5% PZ + 2% DBU, 60 °C | 99 (0 / 1) | 52.4 | n.d. | 707.40/707.41 |
| | 20% DPA, 60 °C | 98 (0 / 2) | 44.3 | n.d. | 707.40/707.41 |
| | 20% DPA + 0.5M Oxyma, 60 °C | 94 (0 / 6) | 50.0 | n.d. | 707.40/707.41 |
| **Afamelanotide**<br>Ac-SYSNleEHfRWGKPV | 20% PPR, 60 °C | 46 | 69.8 | 16.8 | 1646.84/1646.84 |
| | 25% DPA, 60 °C | 50 | 45.1 | 9.9 | 1646.84/1646.84 |
| **Bivalirudin**<br>fPRPGGGGNGDFEEIPEEYL [a)] | 20% PPR, 60 °C | 77 | n.d. | 46.3 | 2179.99/2179.99 |
| | 25% DPA, 60 °C | 77 | n.d. | 38.7 | 2179.99/2179.99 |
| | 20% PPR, 90 °C | 28 | 6.6 | n.d. | 2179.99/2179.99 |
| | 25% DPA, 90 °C | 25 | 4.6 | n.d. | 2179.99/2179.99 |
| **G1KL**<br>$(KL)_2$*K*KL | 20% PPR, 60 °C | 90 | 72.5 | n.d. | 869.66/869.66 |
| | 5% PZ + 2% DBU, 60 °C | 97 | 26.2 | n.d. | 869.66/869.66 |
| | 20% DIPA, 60 °C | 0 | 0 | n.d. | 869.66/- |
| | 20% DIPA + 1% DBU, 60 °C | 86 | 35.6 | n.d. | 869.66/869.66 |
| | 20% DPA, 60 °C | 78 | 35.2 | n.d. | 869.66/869.66 |
| | 20% DPA + 1% DBU, 60 °C | 85 | 34.0 | n.d. | 869.66/869.66 |
| | 25% DPA, 60 °C | 88 | 64.5 | n.d. | 869.66/869.66 |
| **G2KL**<br>$(KL)_4$(*K*KL)$_2$*K*KL | 20% PPR, r.t. | 79 | 64.9 | n.d. | 2090.57/2090.56 |
| | 5% PZ + 2% DBU, r.t. | 74 | 53.9 | n.d. | 2090.57/2090.56 |
| | 20% DPA, r.t. | 82 | 42.2 | n.d. | 2090.57/2090.56 |
| | 25% DPA, r.t. | 80 | 46.4 | n.d. | 2090.57/2090.56 |
| **G3KL**<br>$(KL)_8$(*K*KL)$_4$(*K*KL)$_2$*K*KL | 20% PPR, 60 °C | 74 | 46.8 | n.d. | 4532.38/4532.39 |
| | 25% DPA, 60 °C | N/A [e)] | N/A [e)] | n.d. | 4532.38/4532.39 |
| | 25% DPA (+ 1% DBU last generation), 60 °C | N/A [e)] | N/A [e)] | n.d. | 4532.38/4532.39 |

| | | | | | |
|---|---|---|---|---|---|
| | 2% DBU, 60 °C<br>20% PPR, r. t.<br>25% DPA, r. t. | 70<br>78<br>29 | 48.4<br>40.5<br>12.3 | n.d.<br>n.d.<br>n.d. | 4532.38/4532.39<br>4532.38/4532.37<br>4532.38/4532.37 |

[a] One letter code for amino-acids, D- amino acids in lower case, K indicates branching L- lysine, C-termini are carboxamide except for Bivalirudin which is carboxyl. Ac =acetyl group, Nle = norleucine. [b] SPPS was carried out in DMF using Oxyma/DIC as coupling reagents and the indicated base for Fmoc removal. PPR = Piperidine, PZ = Piperazine, DBU = 1,8-diazabicyclo[5.4.0]undec-7-ene, DPA = Dipropylamine, DIPA = Diisopropylamine, DEA = Diethylamine, DBA = Dibutylamine, DIBA = Diisobutylamine. Percentages (%) are in w/v in case of PZ and in v/v otherwise. [c] Crude purity for hexapeptides **1-7** is given as follow: % desired product (% aspartimide or glutarimide / % other byproducts). The crude product after resin cleavage was precipitated, washed and dried, and analyzed by analytical HPLC to determine the percentage of desired product, aspartimide and other byproducts. [d] Crude yield is calculated as followed: (crude mass / molecular weight of desired peptide) / (mass of resin × resin loading) × % of desired product content in crude. [e] Not applicable. Peak integration was not possible in cases of **G3KL**, 25% DPA and 25% DPA + 1% DBU for the last generation, 60 °C due to byproducts / impurities in the crude but traces of desired compounds were observed by HRMS (see Supporting Information). [f] Isolated yields were calculated after preparative RP-HPLC purification according to the amount of resin and its indicated loading. n.d. = not determined.

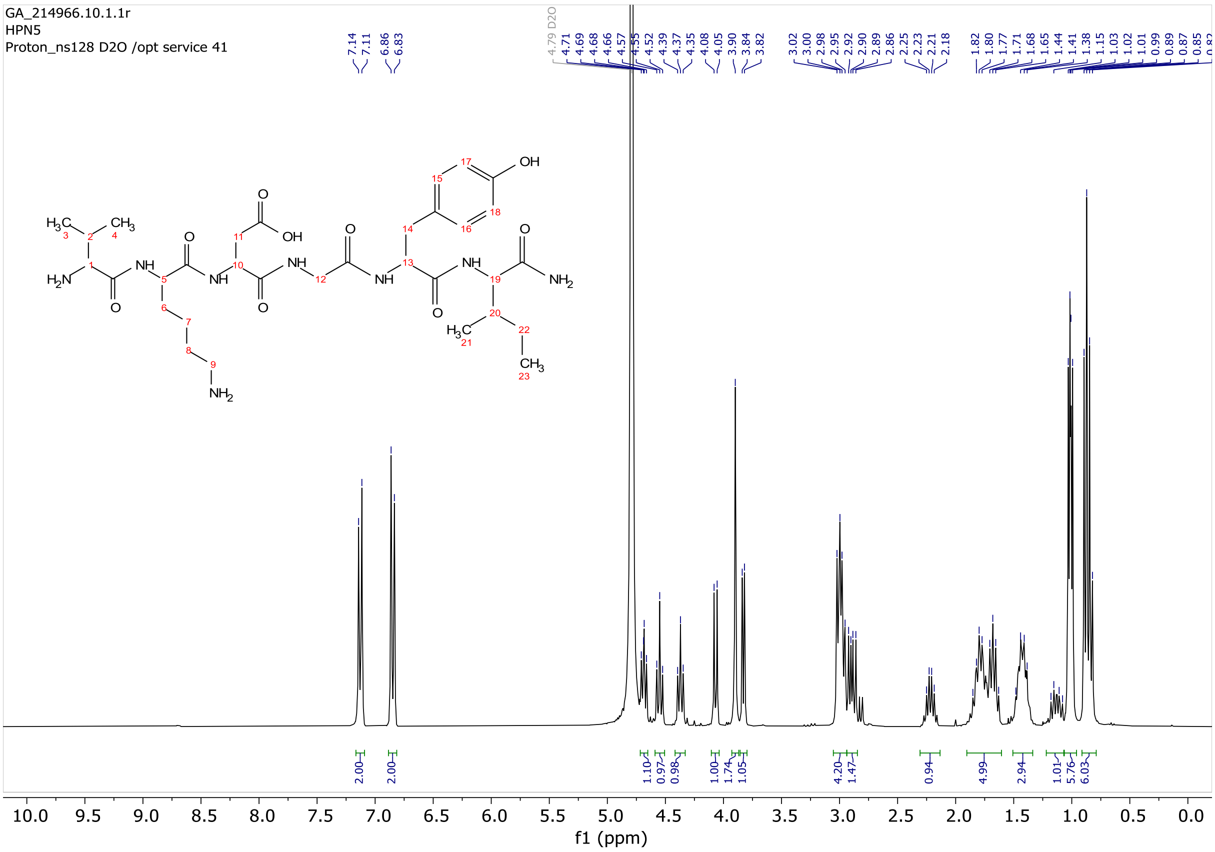


**Figure S1:** $^1$H NMR spectra of purified peptide **1** (VKDGYI) synthesized using 25% DPA. (300 MHz, $D_2O$) : δ = 7.13 (d, *J* = 8.5 Hz, 2H, C(15 and 16)), 6.85 (d, *J* = 8.5 Hz, 2H, C(17 and 18)), 4.69 (t, 1H, C(13)), 4.55 (t, *J* = 7.6 Hz, 1H, C(10)), 4.37 (t, *J* = 7.2 Hz, 1H, C(5)), 4.07 (d, *J* = 7.9 Hz, 1H, C(19)), 3.90 (s, 2H, C(12)), 3.83 (d, *J* = 5.8 Hz, 1H, C(1)), 3.02 – 2.86 (m, 6H, C(9, 11 and 14)), 2.28 – 2.15 (m, 1H, C(20)), 1.82 – 1.65 (m, 6H, C(6, 8 and 22)), 1.51 – 1.35 (m, 3H, C(2 and 7)), 1.01 (dd, *J* = 7.0, 4.3 Hz, 6H, C(21 and 23)), 0.89 – 0.82 (m, 6H, C(3 and 4)).

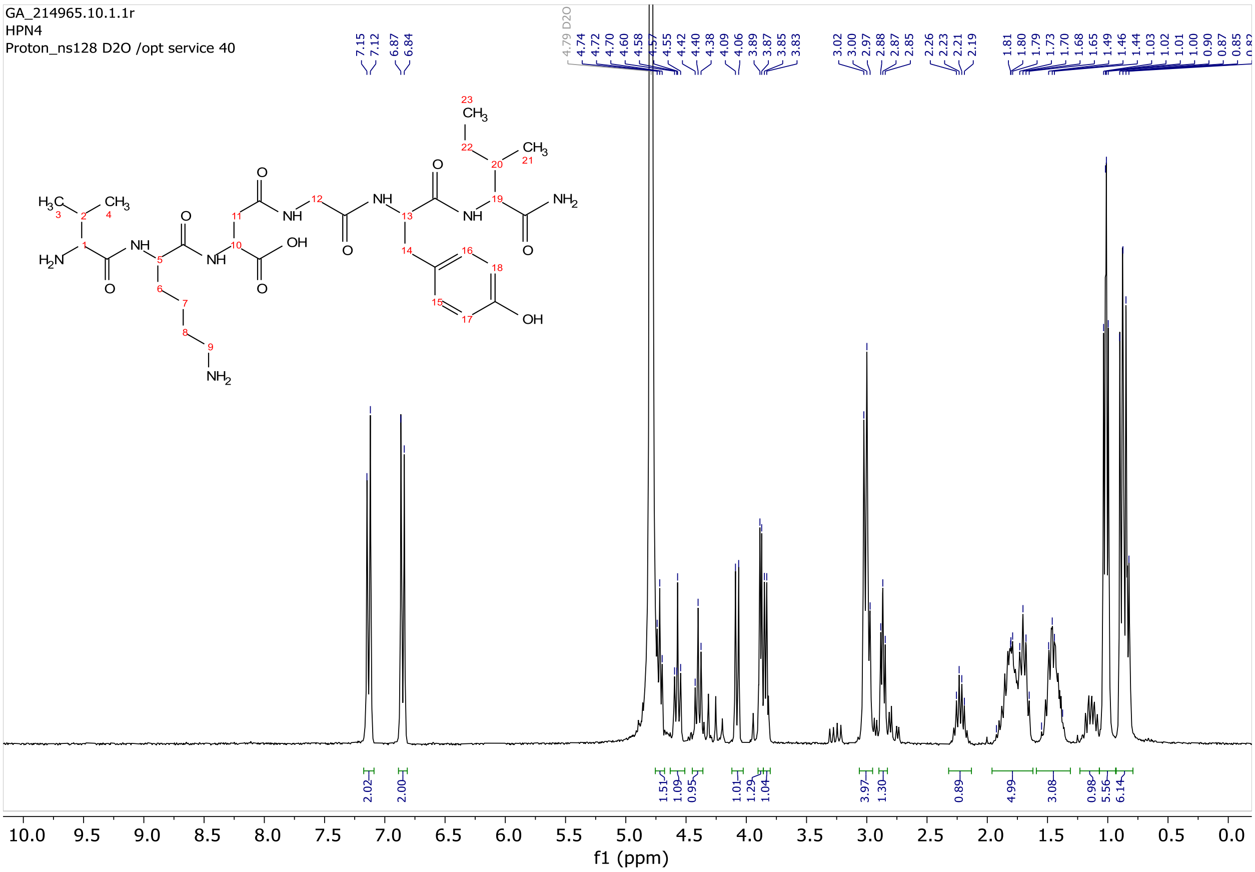


**Figure S2:** $^{1}$H NMR spectra of purified peptide **1β** (300 MHz, $D_2O$) : δ = 7.13 (d, $J$ = 8.6 Hz, 2H, C(15 and 16)), 6.85 (d, $J$ = 8.6 Hz, 2H, C(17 and 18)), 4.72 (t, 1H, C(13)), 4.57 (t, $J$ = 7.5 Hz, 1H, C(10)), 4.40 (t, $J$ = 7.3 Hz, 1H, C(5)), 4.08 (d, $J$ = 7.9 Hz, 1H, C(19)), 3.86 (m, 3H, C(1 and 12)), 3.00 (m, 4H, C(11 and 14)), 2.87 (t, $J$ = 5.6 Hz, 2H, C(9)), 2.29 – 2.16 (m, 1H, C(20)), 1.84 – 1.62 (m, 6H, C(6, 8 and 22)), 1.49 – 1.44 (m, 3H, C(2 and7)), 1.02 (dd, $J$ = 7.0, 4.6 Hz, 6H, C(21 and 23)), 0.93 – 0.79 (m, 6H, C(3 and 4)).

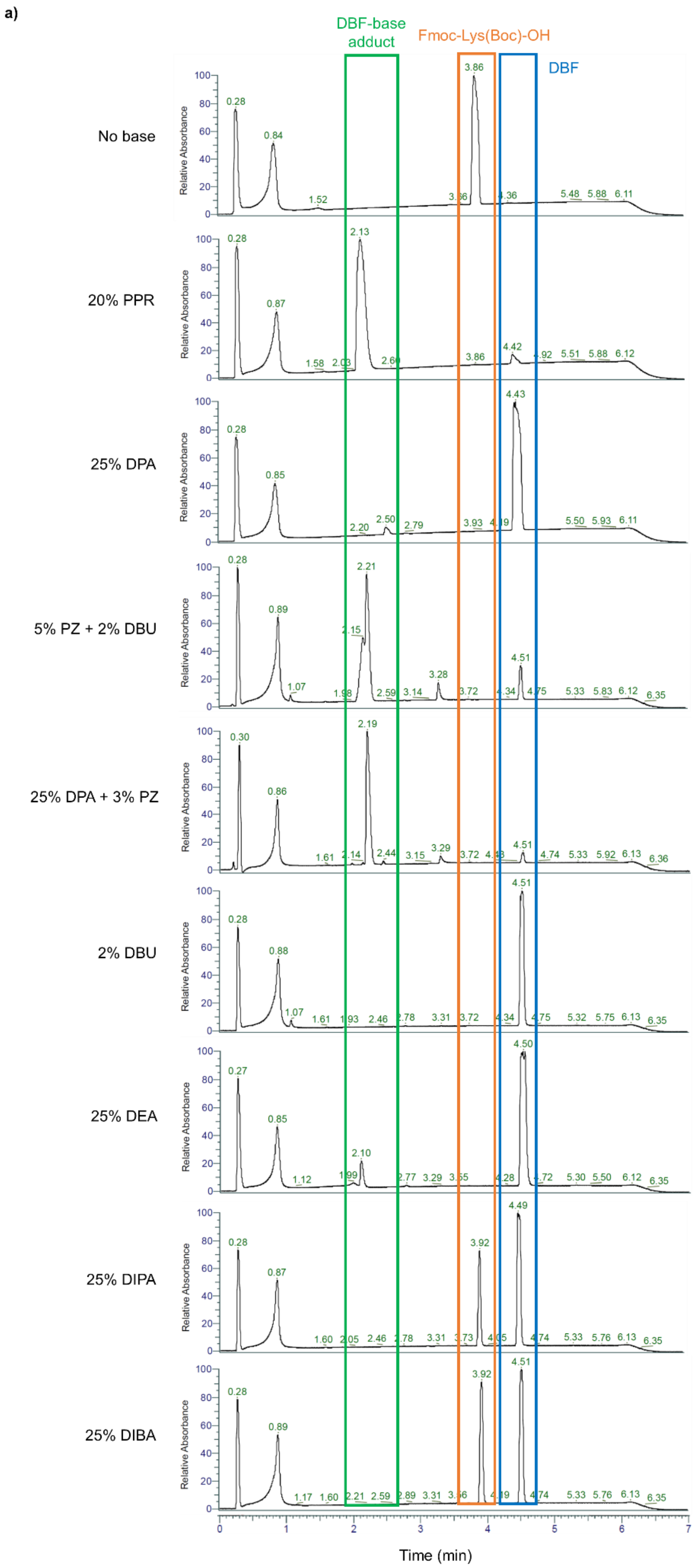
a)
DBF-base adduct
Fmoc-Lys(Boc)-OH
DBF
No base
20% PPR
25% DPA
5% PZ + 2% DBU
25% DPA + 3% PZ
2% DBU
25% DEA
25% DIPA
25% DIBA
Relative Absorbance
Time (min)

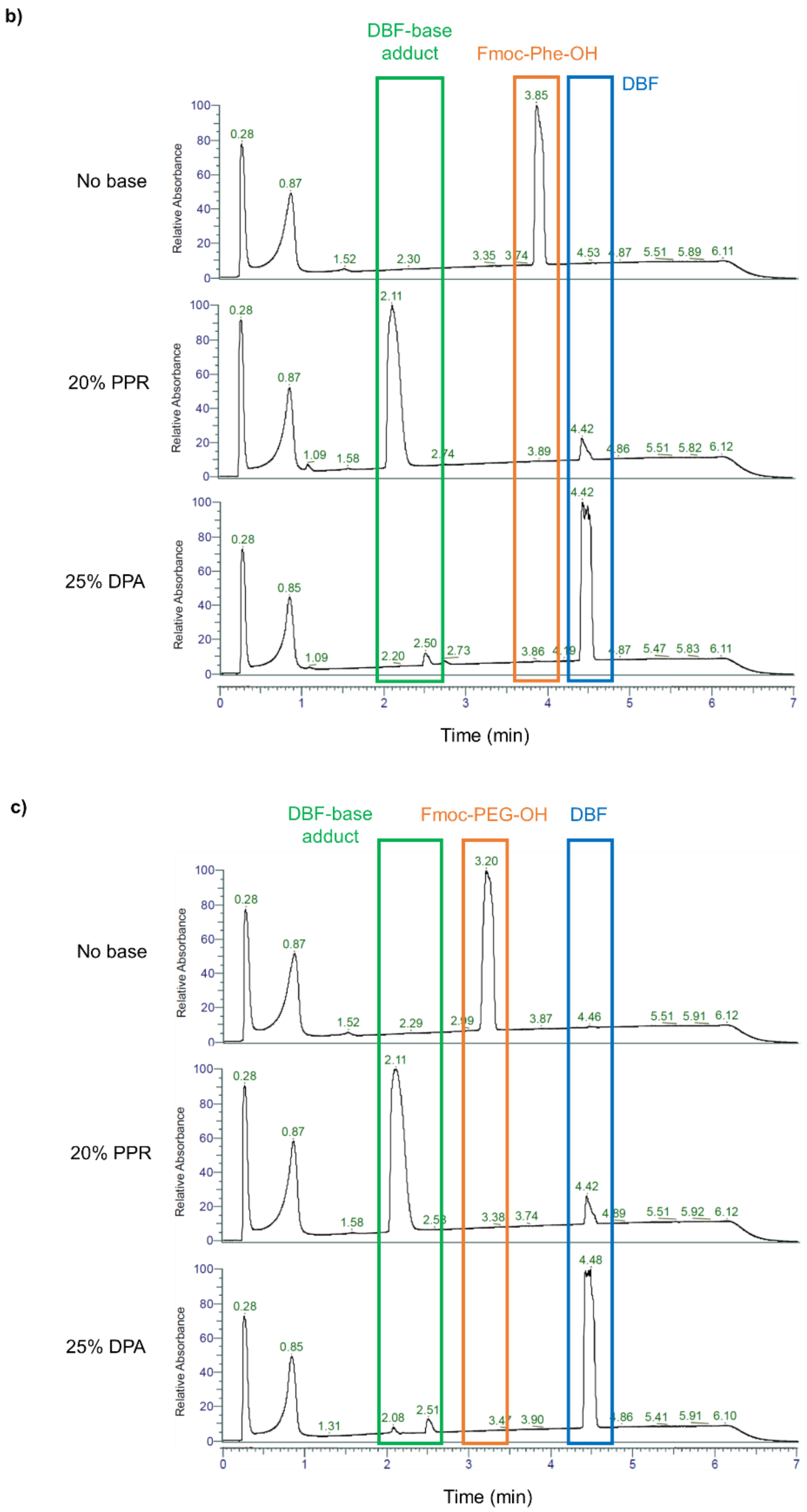


**Figure S3:** (previous page and above) Fmoc deprotection in DMF at room temperature during 30 minutes of a) Fmoc-Lys(Boc)-OH ($t_R$ = 3.86-3.92 min), b) Fmoc-Phe-OH ($t_R$ = 3.85 min) and c) Fmoc-PEG-OH ($t_R$ = 3.20 min). DBF-PPR adduct ($t_R$ = 2.11-2.13 min), DBF-PZ adduct ($t_R$ = 2.19-2.21 min) DBF-DPA adduct ($t_R$ = 2.50-2.51 min), DBF-DEA adduct ($t_R$ = 2.10 min) and DBF ($t_R$ = 4.42-4.51 min) can be observed. Deprotection was carried out at r.t. during 30 min. DBF = dibenzofulvene, PPR = Piperidine, DPA = Dipropylamine, PZ = Piperazine, DBU = 1,8-diazabicyclo[5.4.0]undec-7-ene, DEA = diethylamine, DIPA = Diisopropylamine, DIBA = Diisobutylamine.

# 2. Compound characterization

## 2.1 Hexapeptide **1** (VKDGYI)

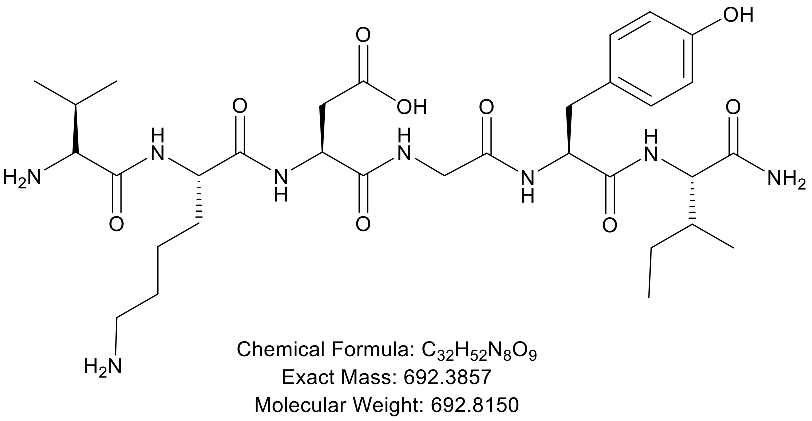

**VKDGYI** (20% v/v Piperidine, 60°C) was obtained as as white solid after preparative RP-HPLC (3.2 mg, 4.5%). Crude analysis: Analytical RP-HPLC: $t_R$ = 1.92 min (A/D 100:0 to 0:100 in 7.00 min, $\lambda$ = 214nm). HRMS (ESI+): $C_{32}H_{52}N_8O_9$ calc./obs. 693.39/693.39 Da $[M+H]^+$.

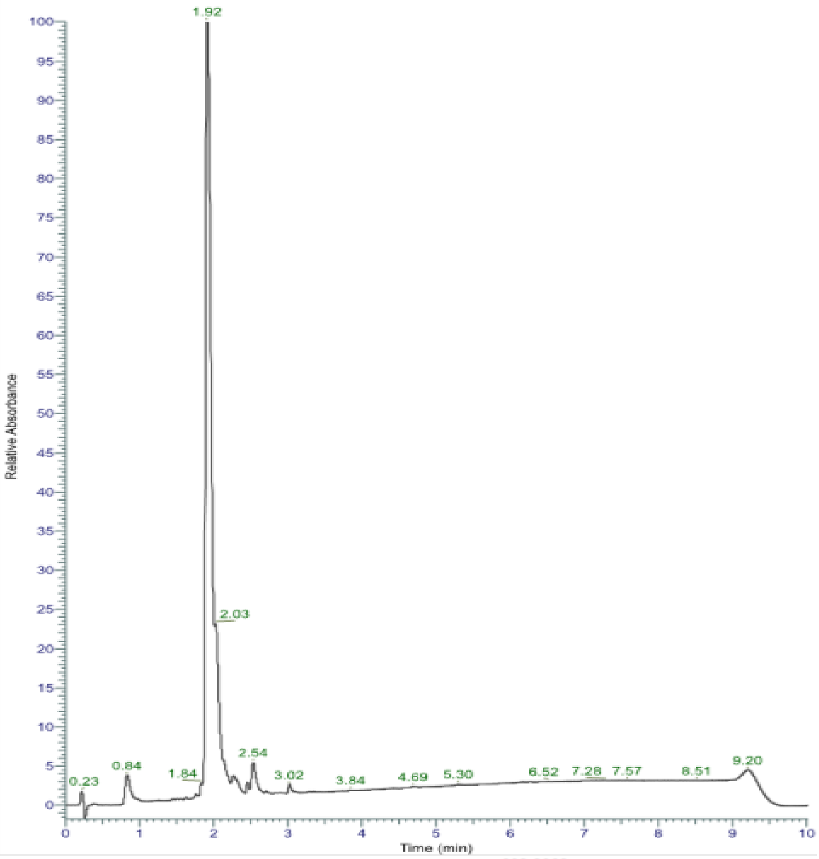


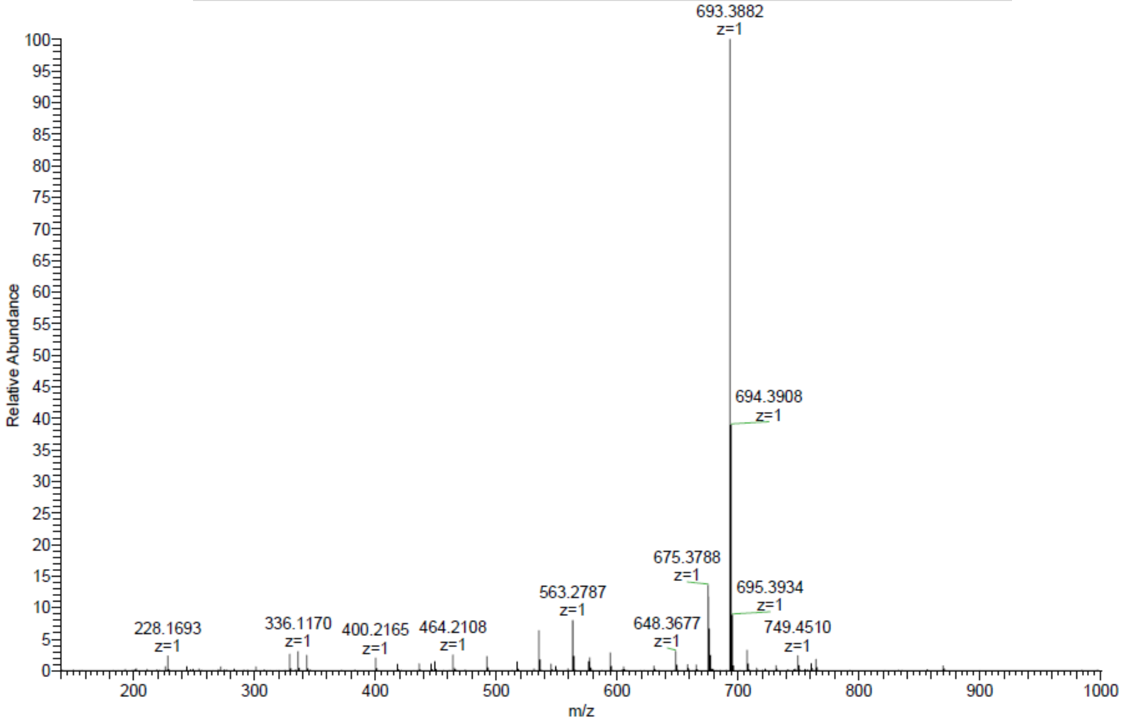

**VKDGYI** (20% v/v Piperidine + 0.25 M Oxyma, 60°C) was obtained as crude white solid after lyophilization (14.2 mg, 22.1%). Analytical RP-HPLC: $t_R$ = 1.91 min (A/D 100:0 to 0:100 in 7.00 min, λ = 214nm). HRMS (ESI+): $C_{32}H_{52}N_8O_9$ calc./obs. 693.39/693.39 Da $[M+H]^+$.

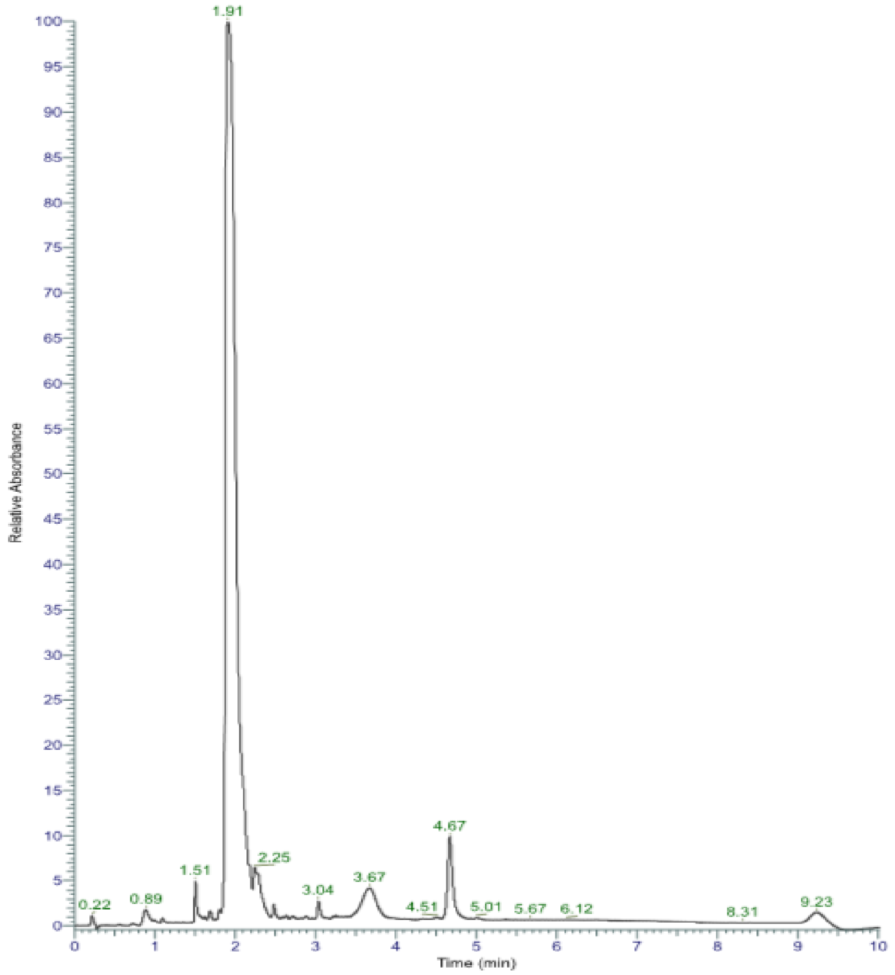


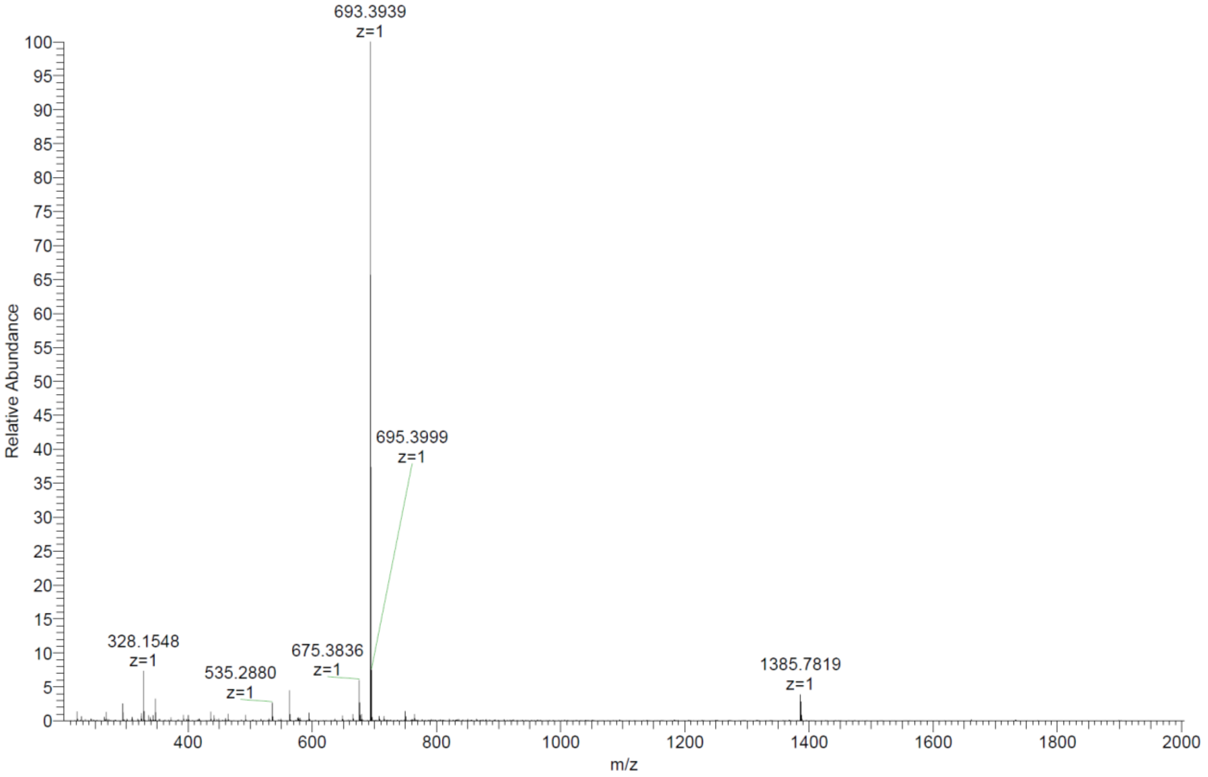

**VKDGYI** (20% v/v Piperidine + 0.5 M Oxyma, 60°C) was obtained as crude white solid after lyophilization (10.8 mg, 16.8%). Analytical RP-HPLC: $t_R$ = 1.99 min (A/D 100:0 to 0:100 in 7.00 min, λ = 214nm). HRMS (ESI+): $C_{32}H_{52}N_8O_9$ calc./obs. 693.39/693.39 Da $[M+H]^+$.

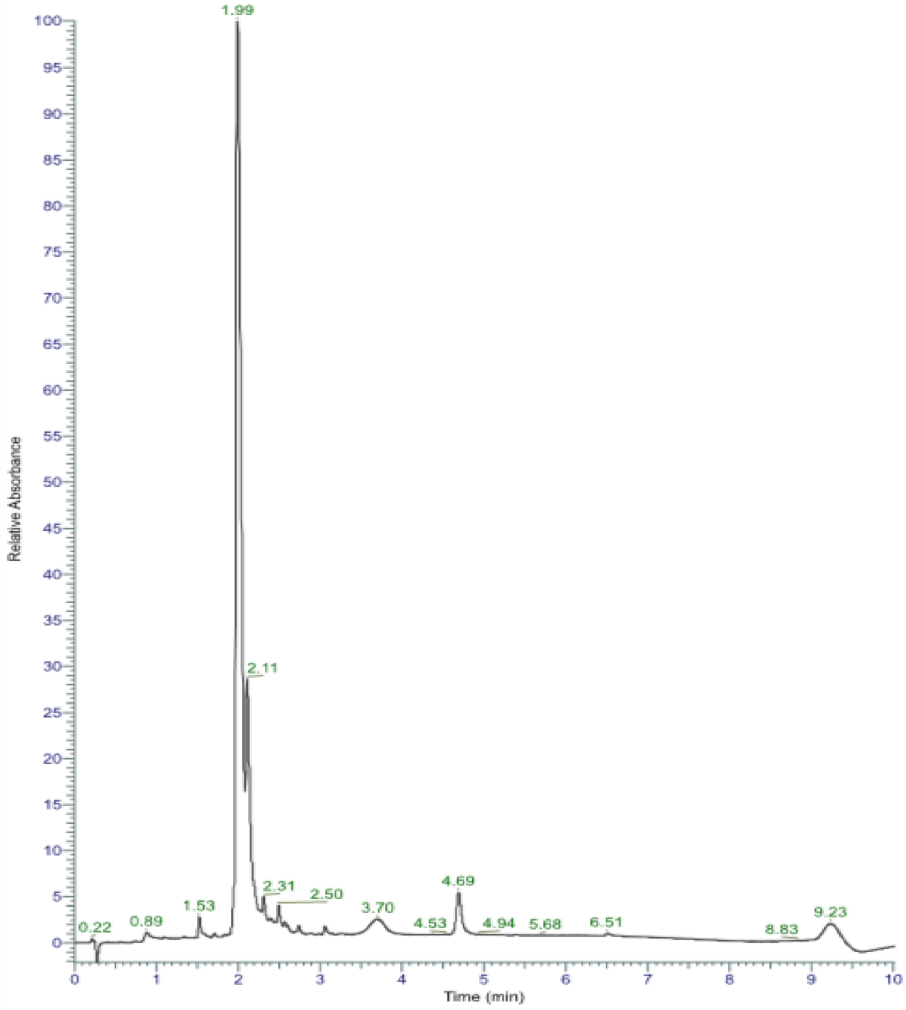


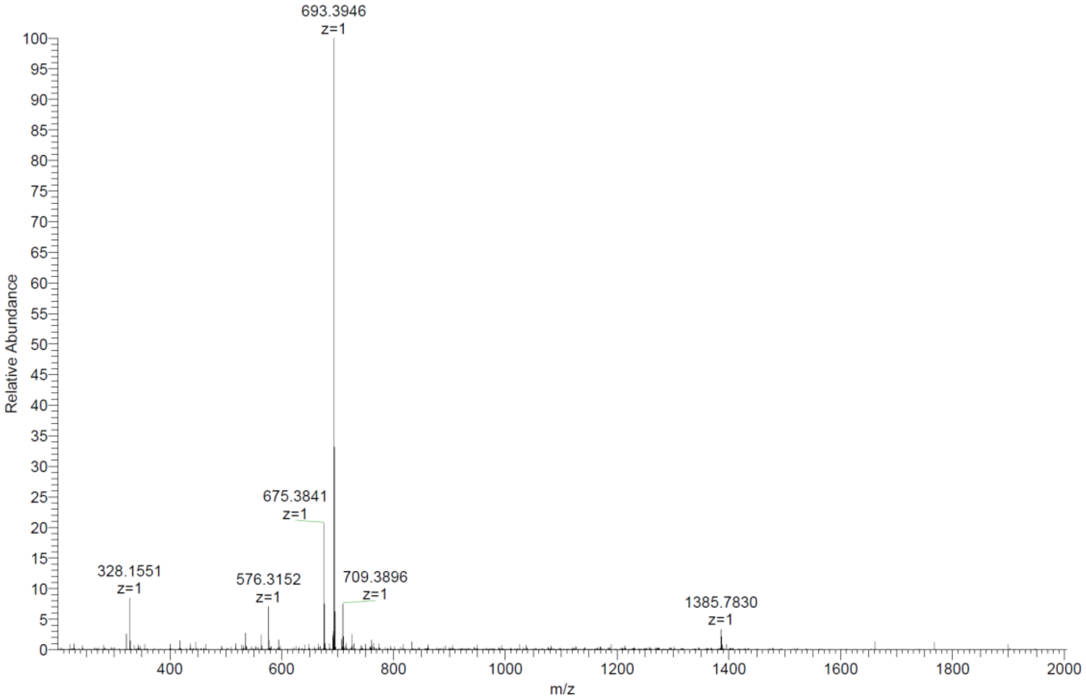

**VKDGYI** (5% w/v Piperazine + 2% v/v DBU, 60°C) was obtained as crude white solid after lyophilization (5.7 mg, 0.0%). Analytical RP-HPLC: $t_R$ = - min (A/D 100:0 to 0:100 in 7.00 min, $\lambda$ = 214nm). HRMS (ESI+): $C_{32}H_{52}N_8O_9$ calc./obs. 693.39/- Da $[M+H]^+$. (No compound observed).

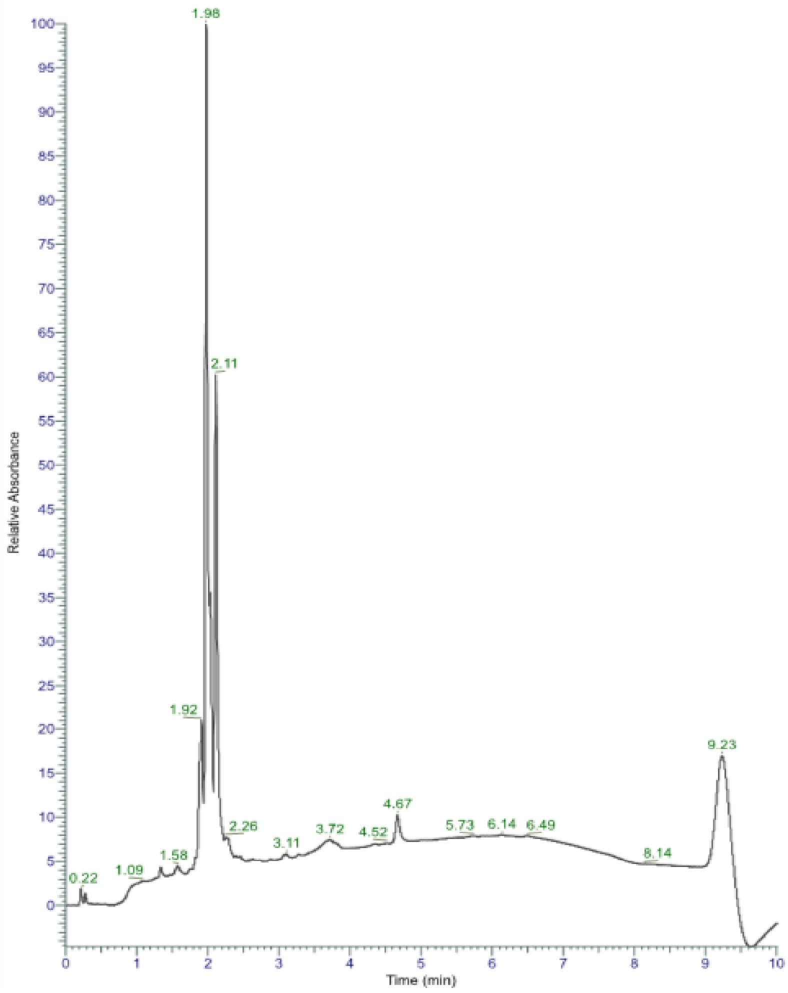


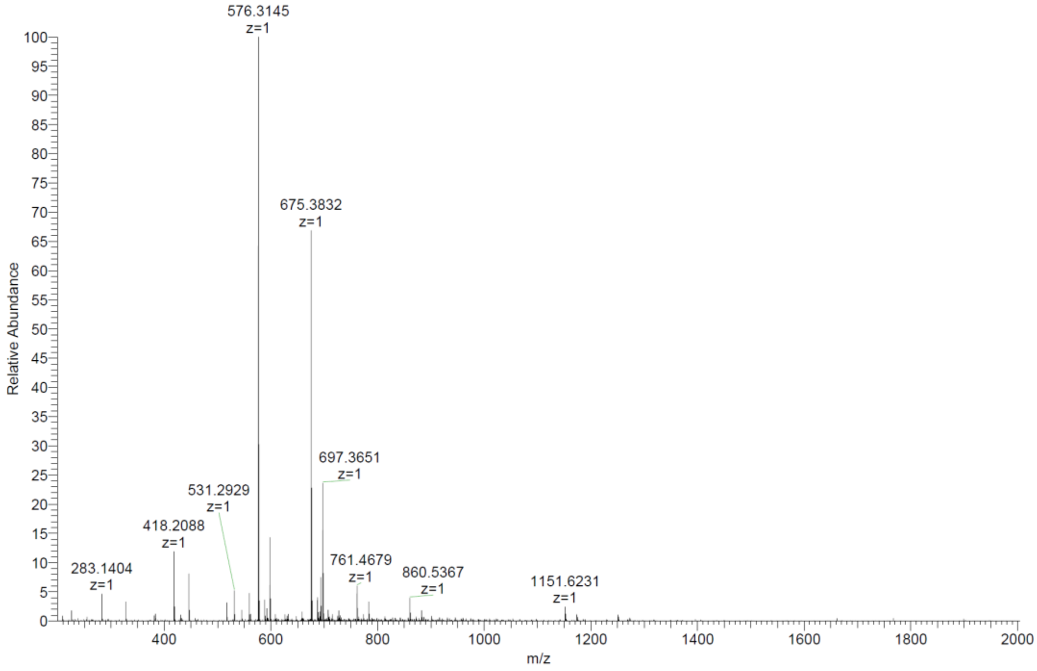

**VKDGYI** (5% w/v Piperazine + 2% v/v DBU + 0.25 M HOBt, 60°C) was obtained as crude white solid after lyophilization (9.8 mg, 9.2%). Analytical RP-HPLC: $t_R$ = 2.01 min (A/D 100:0 to 0:100 in 7.00 min, λ = 214nm). HRMS (ESI+): $C_{32}H_{52}N_8O_9$ calc./obs. 693.39/693.39 Da $[M+H]^+$.

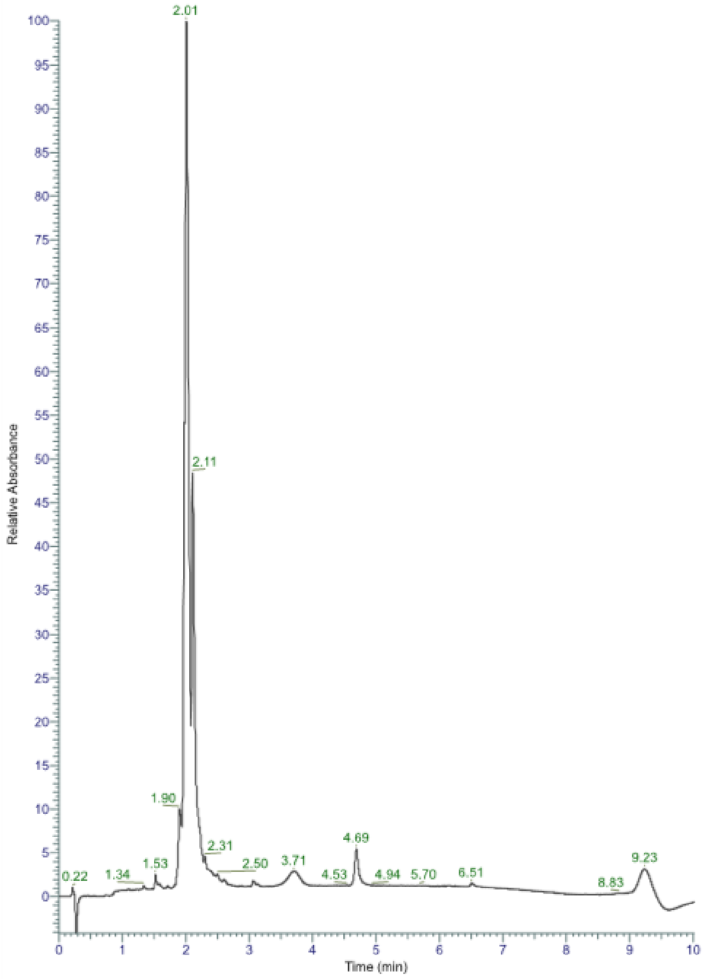


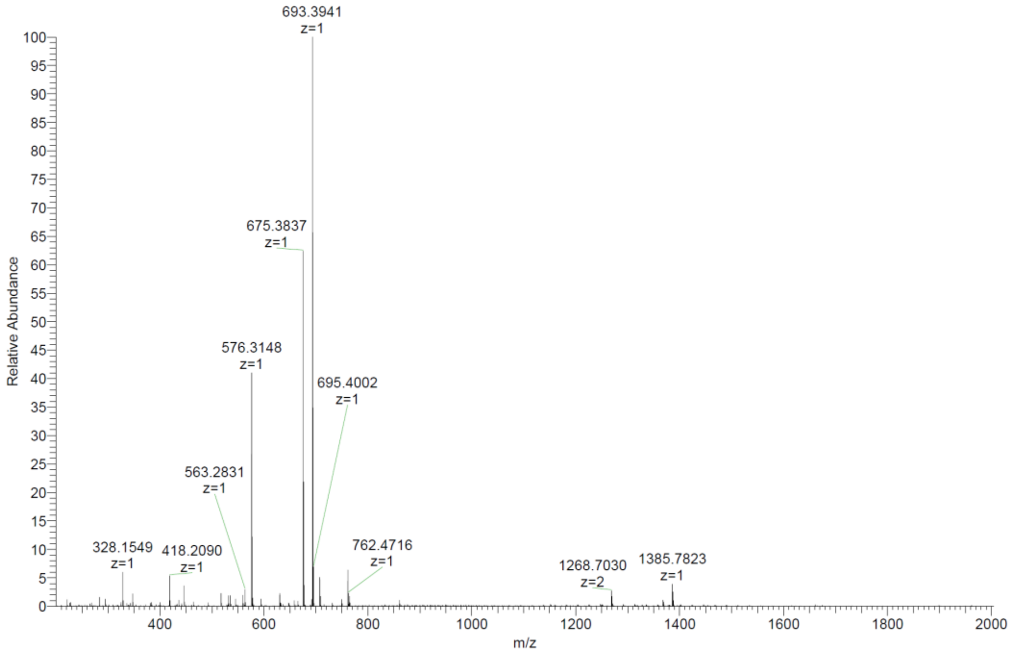

**VKDGYI** (5% w/v Piperazine + 2% v/v DBU + 0.5 M HOBt, 60°C) was obtained as crude white solid after lyophilization (6.6 mg, 10.4%). Analytical RP-HPLC: $t_R$ = 1.92 min (A/D 100:0 to 0:100 in 7.00 min, λ = 214nm). HRMS (ESI+): $C_{32}H_{52}N_8O_9$ calc./obs. 693.39/693.39 Da $[M+H]^+$.

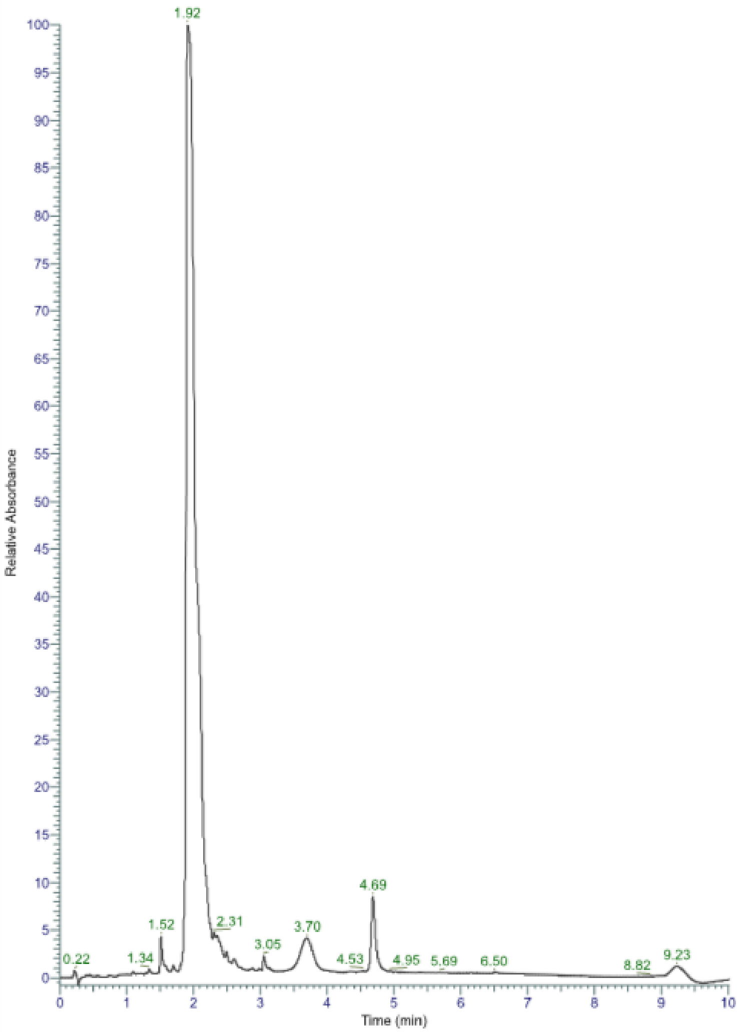


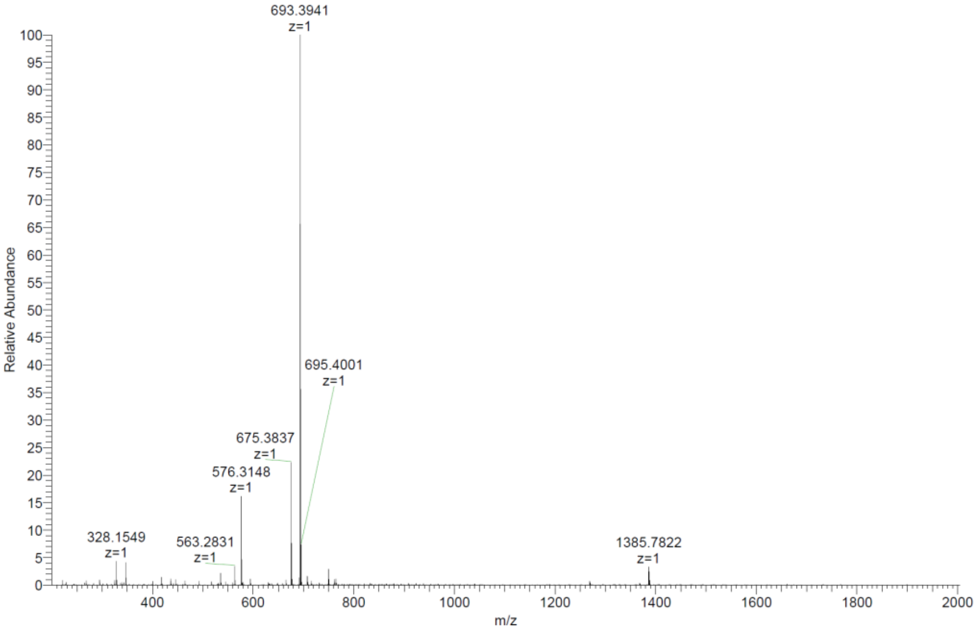

**VKDGYI** (5% w/v Piperazine + 2% v/v DBU + 0.25 M Oxyma, 60°C) was obtained as crude white solid after lyophilization (12.8 mg, 16.3%). Analytical RP-HPLC: $t_R$ = 1.93 min (A/D 100:0 to 0:100 in 7.00 min, λ = 214nm). HRMS (ESI+): $C_{32}H_{52}N_8O_9$ calc./obs. 693.39/693.39 Da $[M+H]^+$.

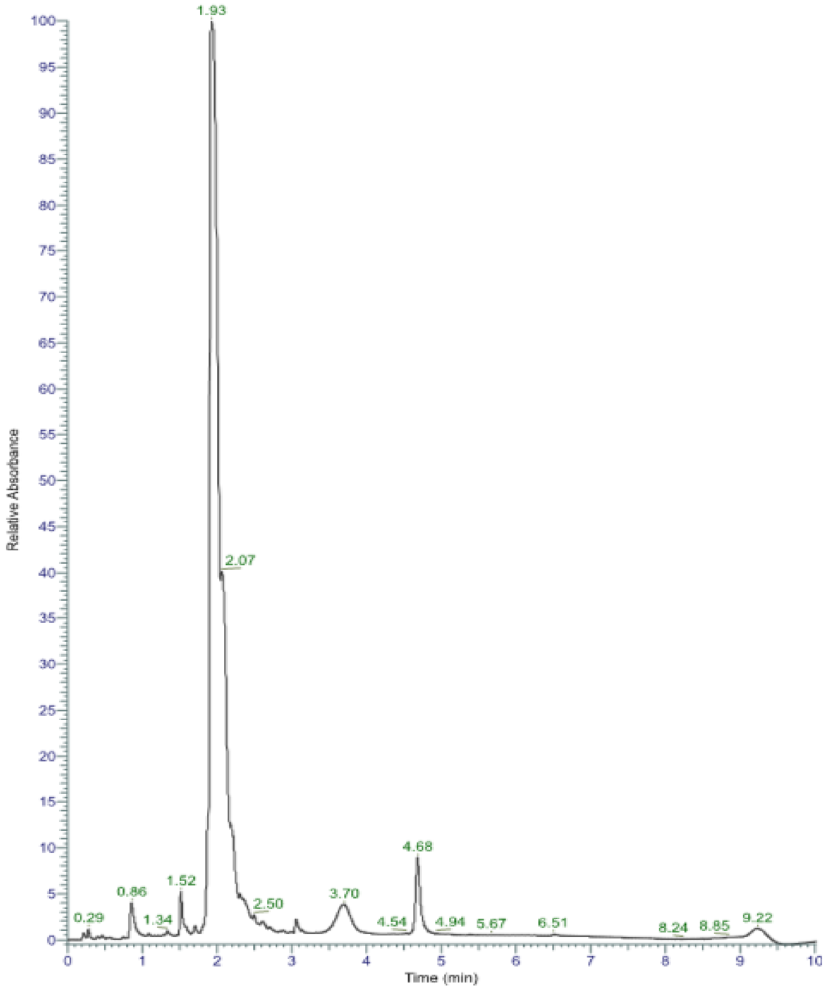


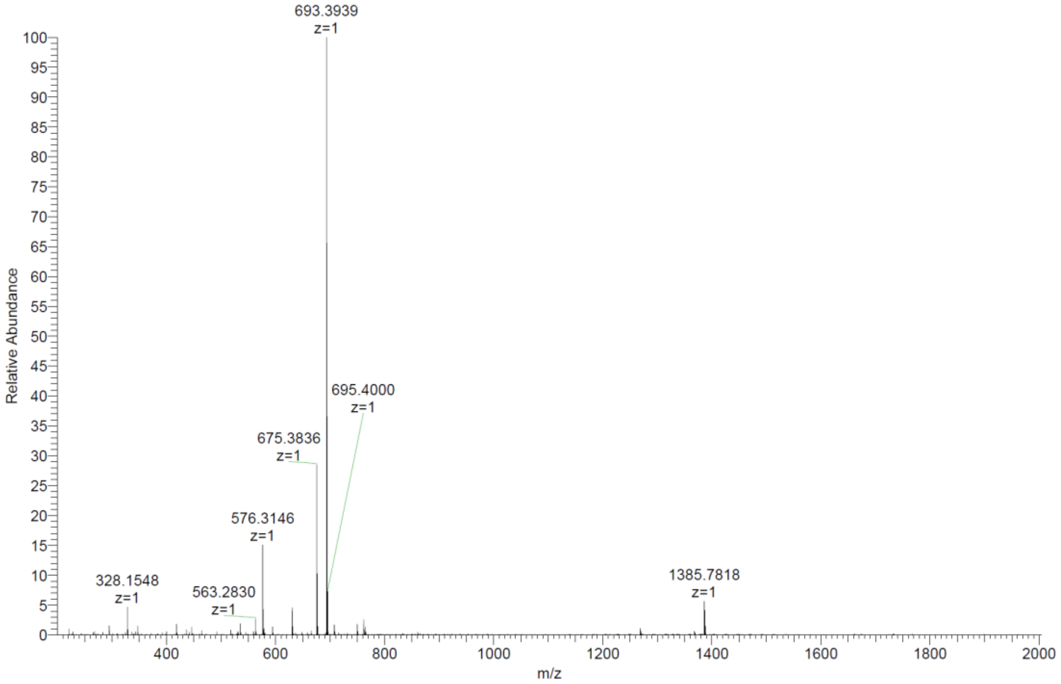

**VKDGYI** (5% w/v Piperazine + 2% v/v DBU + 0.5 M Oxyma, 60°C) was obtained as crude white solid after lyophilization (15.2 mg, 21.8%). Analytical RP-HPLC: $t_R$ = 1.97 min (A/D 100:0 to 0:100 in 7.00 min, λ = 214nm). HRMS (ESI+): $C_{32}H_{52}N_8O_9$ calc./obs. 693.39/693.39 Da $[M+H]^+$.

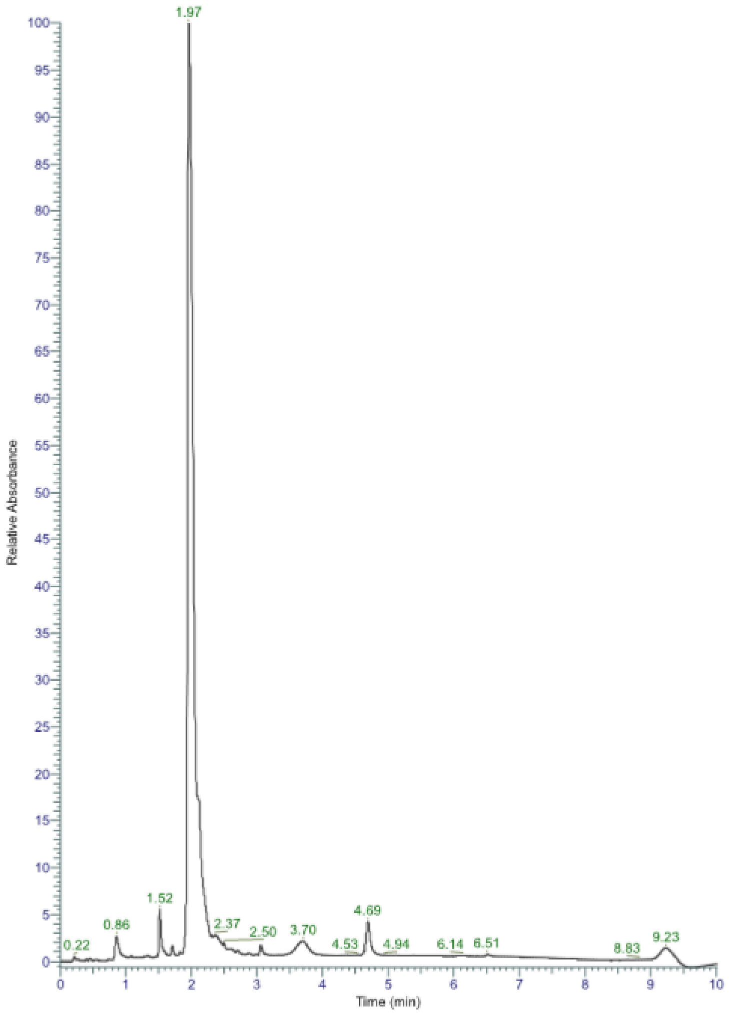


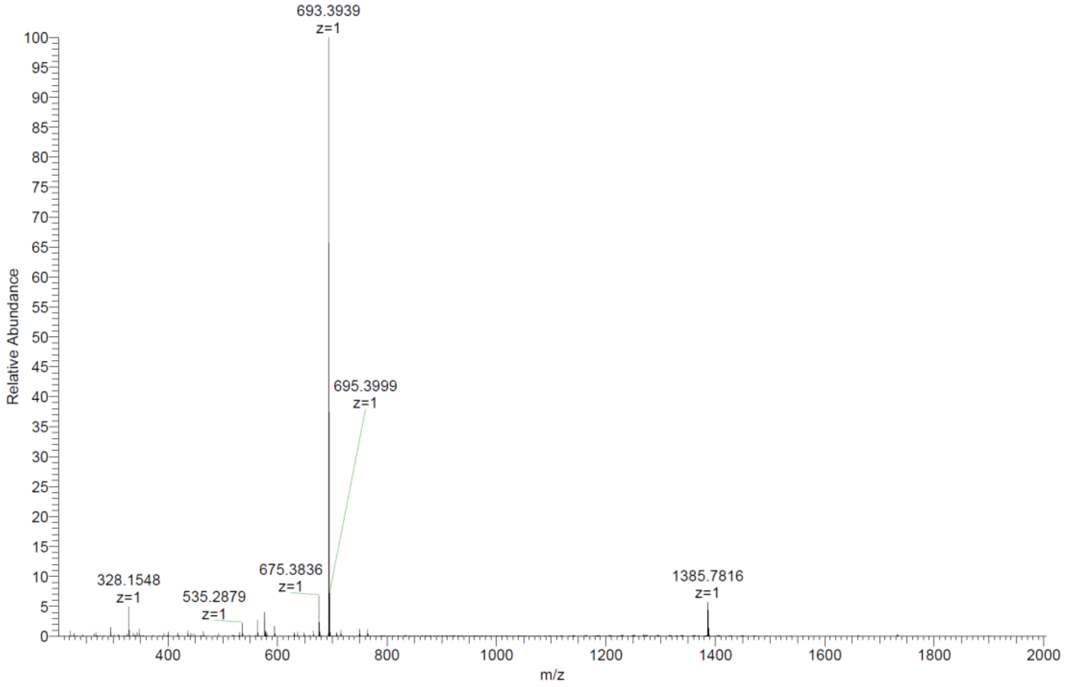

**VKDGYI** (2% v/v DBU, 60°C) was obtained as crude white solid after lyophilization (36.6 mg, 25.7%). Analytical RP-HPLC: $t_R$ = 2.05 min (A/D 100:0 to 0:100 in 7.00 min, λ = 214nm). HRMS (ESI+): $C_{32}H_{52}N_8O_9$ calc./obs. 693.39/693.39 Da $[M+H]^+$.

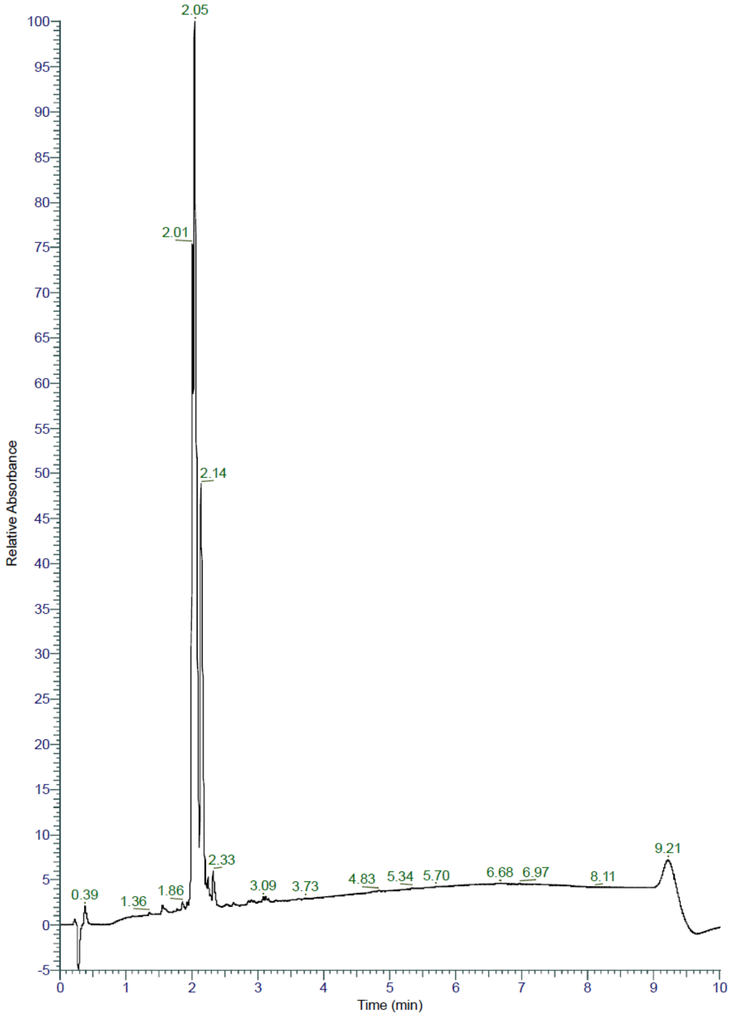


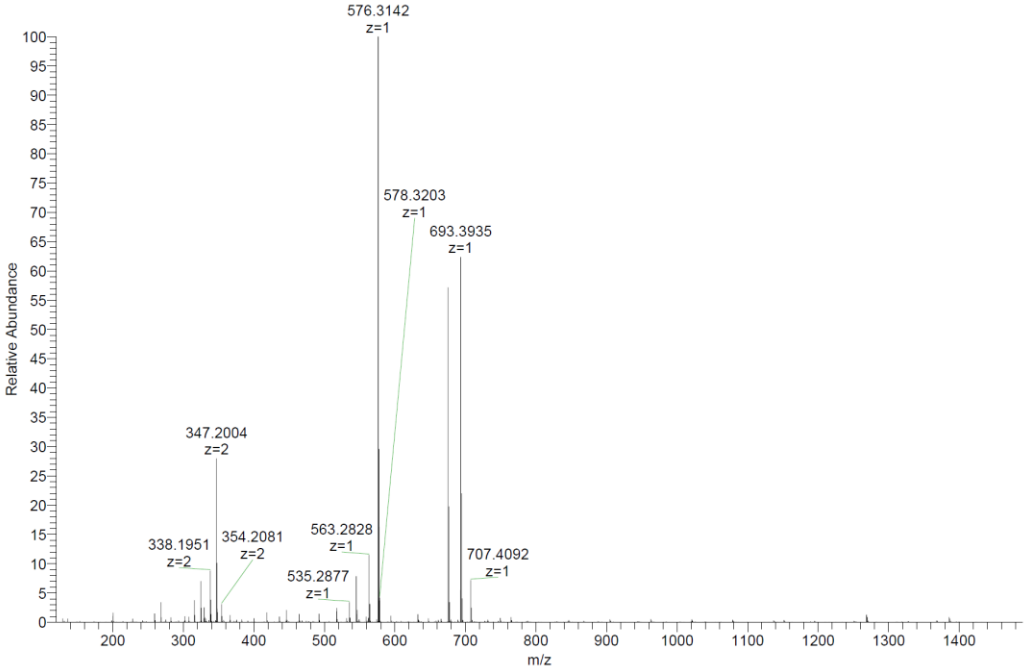

**VKDGYI** (20% v/v Dipropylamine, 60°C) was obtained as crude white solid after lyophilization (37.3 mg, 49.3%). Analytical RP-HPLC: $t_R$ = 1.84 min (A/D 100:0 to 0:100 in 7.00 min, λ = 214nm). HRMS (ESI+): $C_{32}H_{52}N_8O_9$ calc./obs. 693.39/693.39 Da $[M+H]^+$.

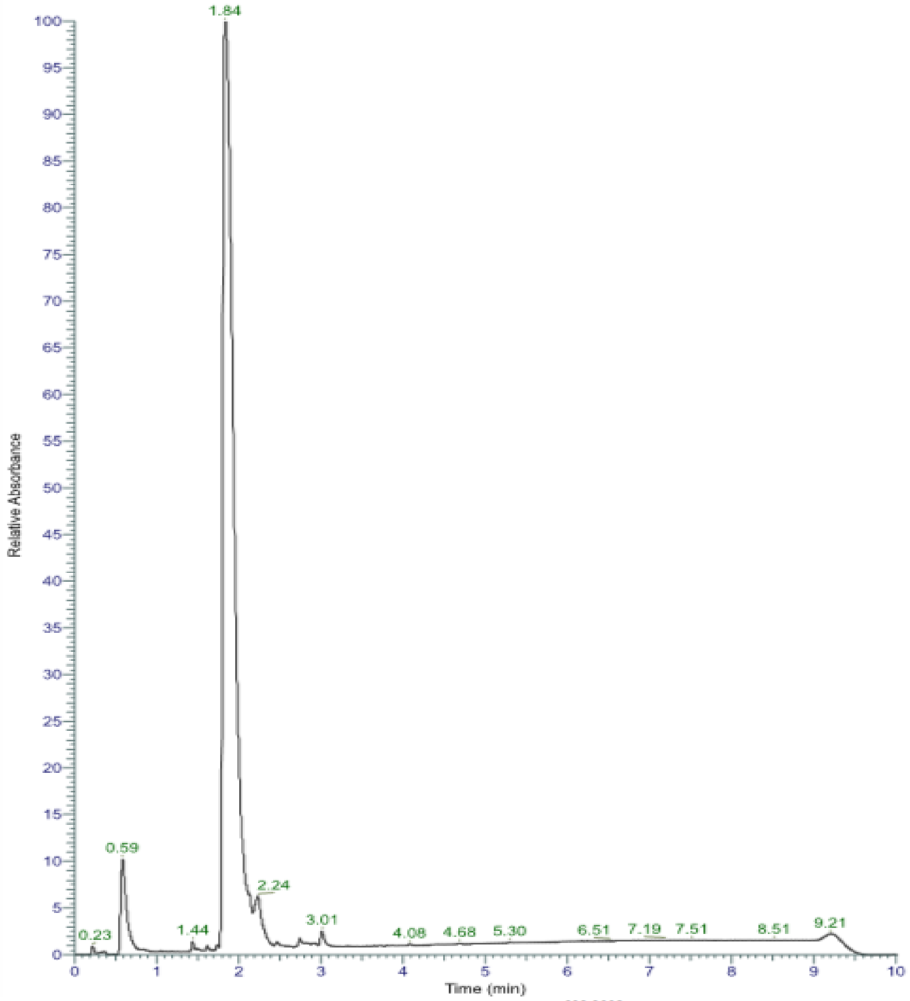


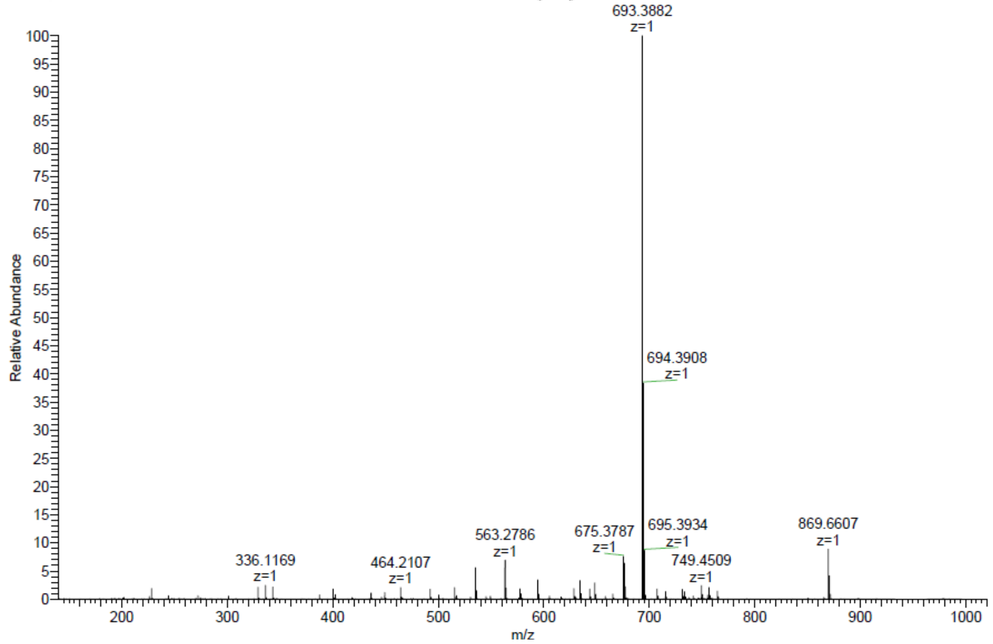

**VKDGYI** (25% v/v Dipropylamine, 60°C) was obtained as as white solid after preparative RP-HPLC (11.5 mg, 16.0%). Analytical RP-HPLC: $t_R$ = 1.20 min (A/D 100:0 to 0:100 in 3.50 min, $\lambda$ = 214nm). MS (ESI+): $C_{32}H_{52}N_8O_9$ calc./obs. 693.39/693.42 Da $[M+H]^+$.

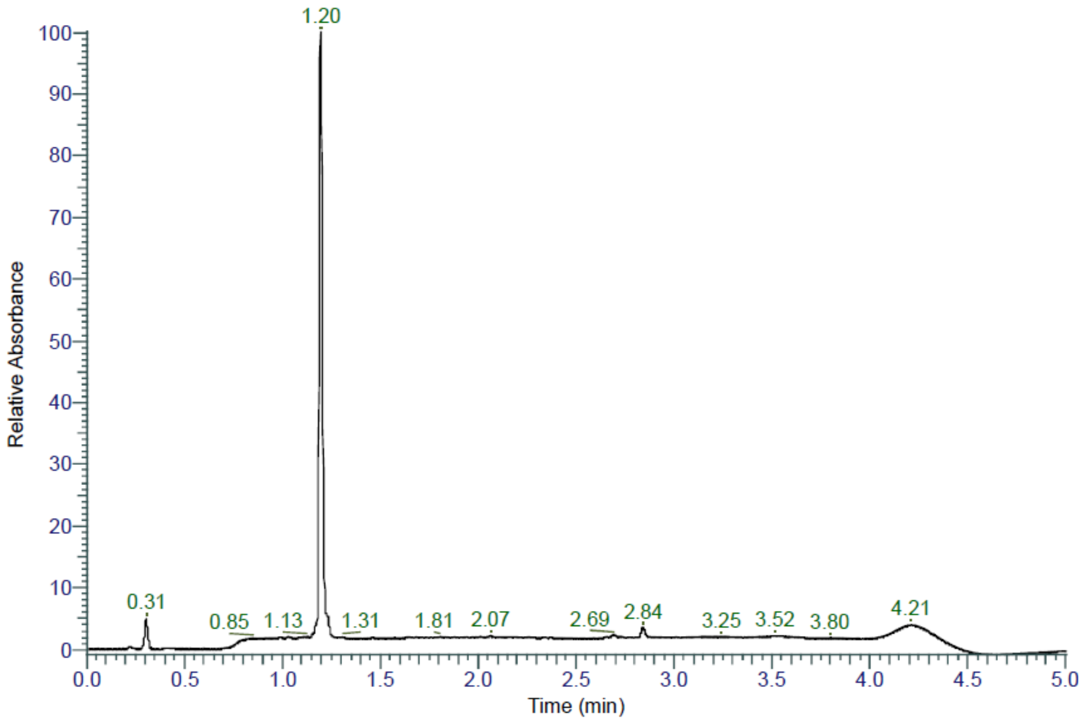


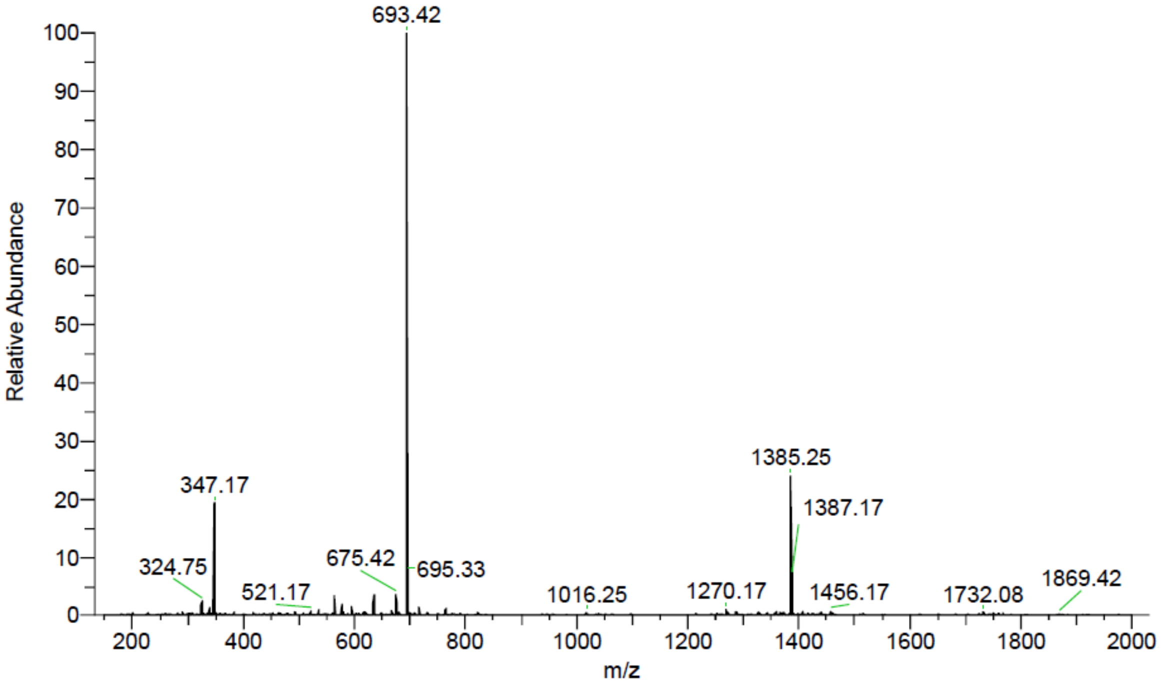

**VKDGYI** (25% v/v Dipropylamine, 60°C) crude: Analytical RP-HPLC: $t_R$ = 1.96 min (A/D 100:0 to 0:100 in 7.00 min, λ = 214nm). HRMS (ESI+): $C_{32}H_{52}N_8O_9$ calc./obs. 693.39/693.39 Da $[M+H]^+$.

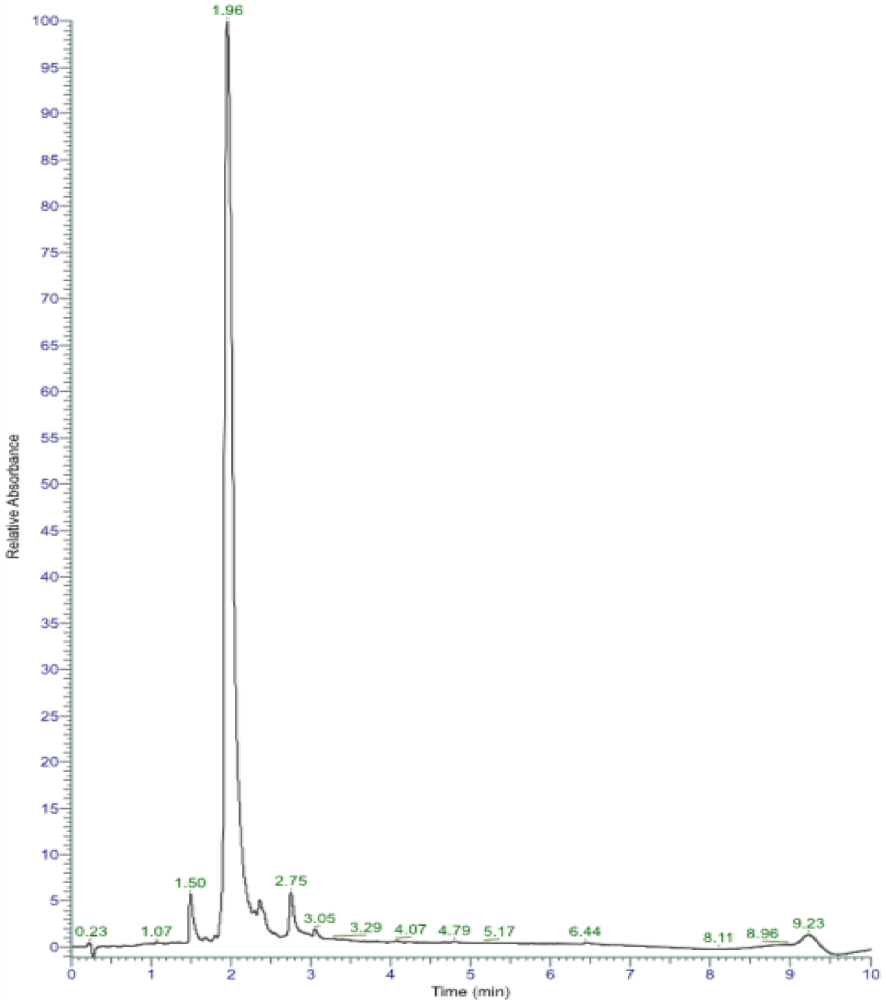


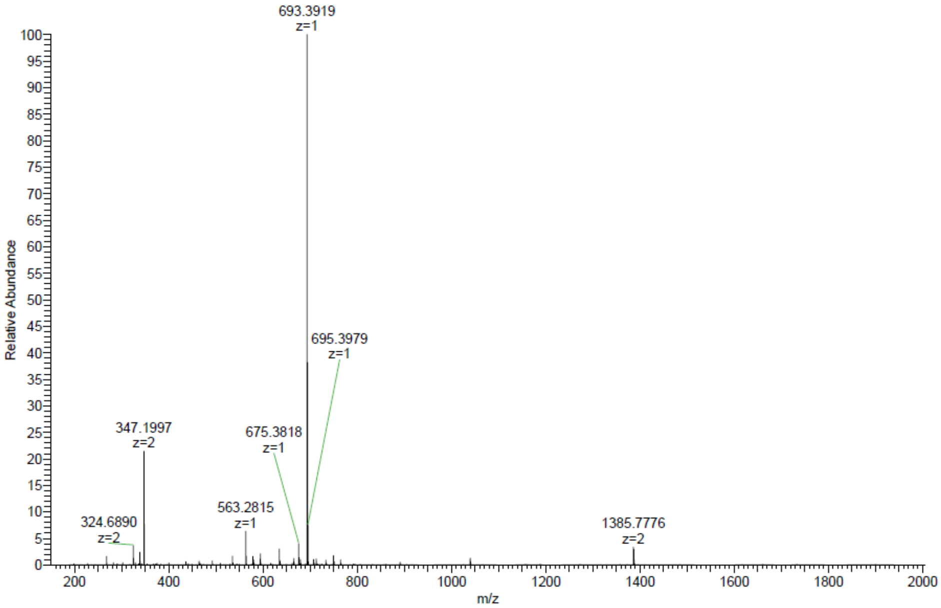

**VKDGYI** (20% v/v Dipropylamine + 0.5 M Oxyma, 60°C) was obtained as crude white solid after lyophilization (35.1 mg, 45.4%). Analytical RP-HPLC: $t_R$ = 1.92 min (A/D 100:0 to 0:100 in 7.00 min, λ = 214nm). HRMS (ESI+): $C_{32}H_{52}N_8O_9$ calc./obs. 693.39/693.39 Da $[M+H]^+$.

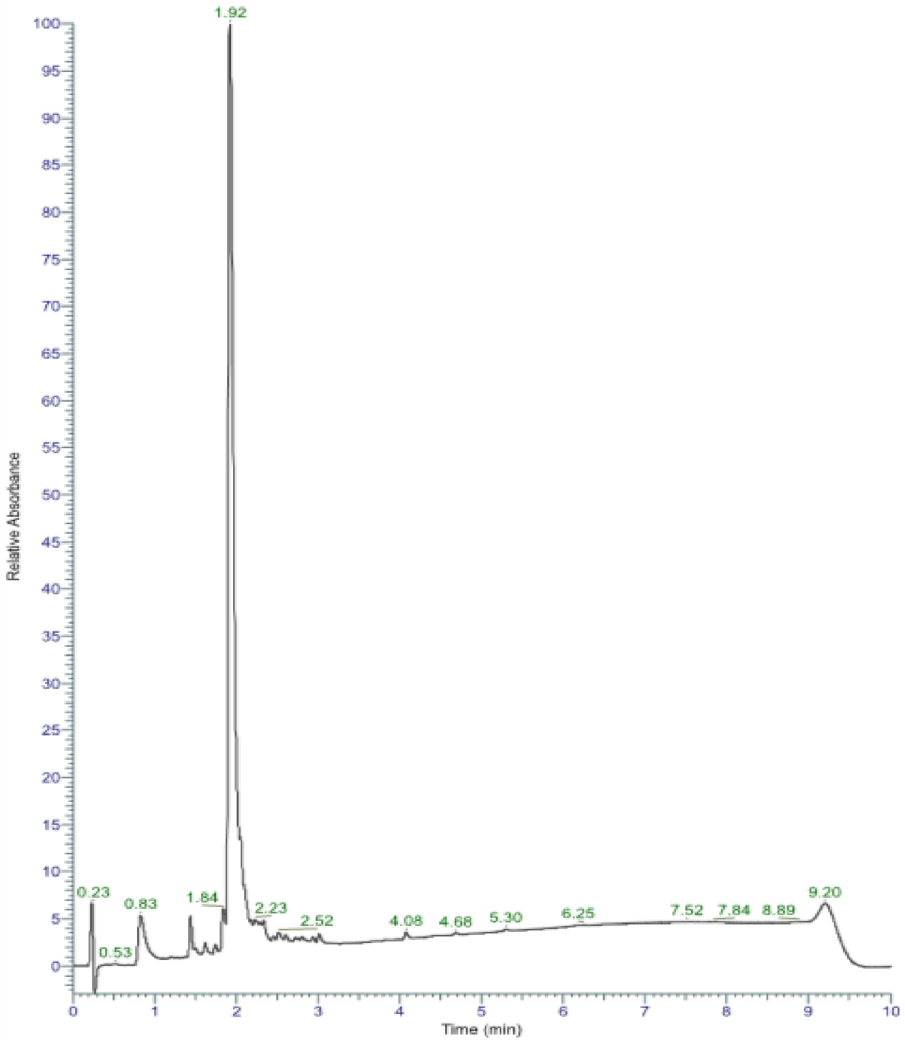


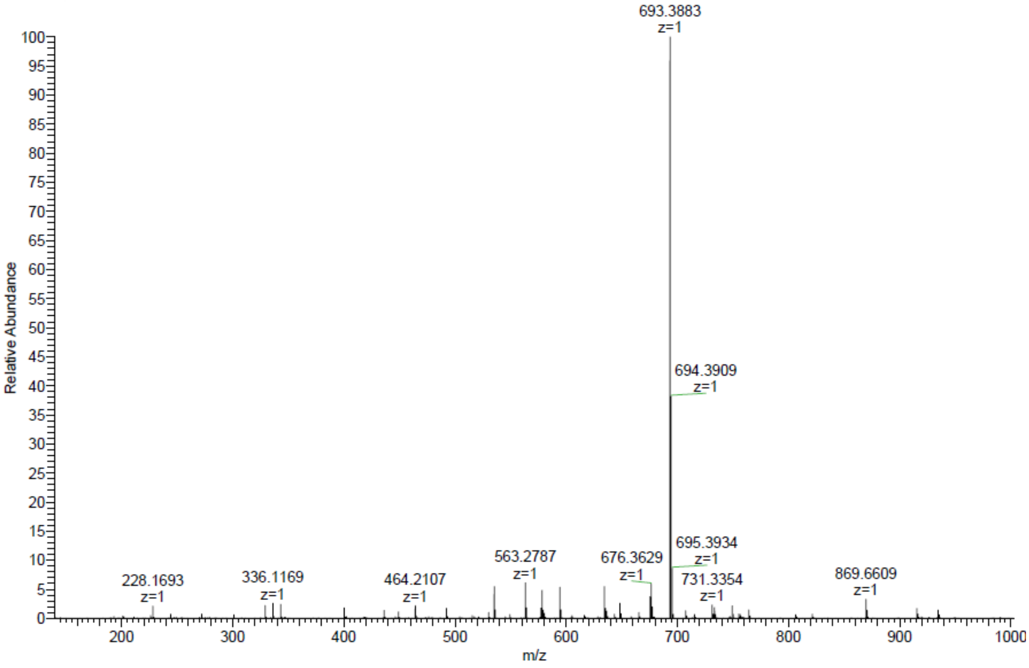

**VKDGYI** (25% v/v Diethylamine, 50°C) was obtained as crude white solid after lyophilization (40.5 mg, 43.4%). Analytical RP-HPLC: $t_R$ = 1.98 min (A/D 100:0 to 0:100 in 7.00 min, λ = 214nm). HRMS (ESI+): $C_{32}H_{52}N_8O_9$ calc./obs. 693.39/693.39 Da [M+H]$^+$.

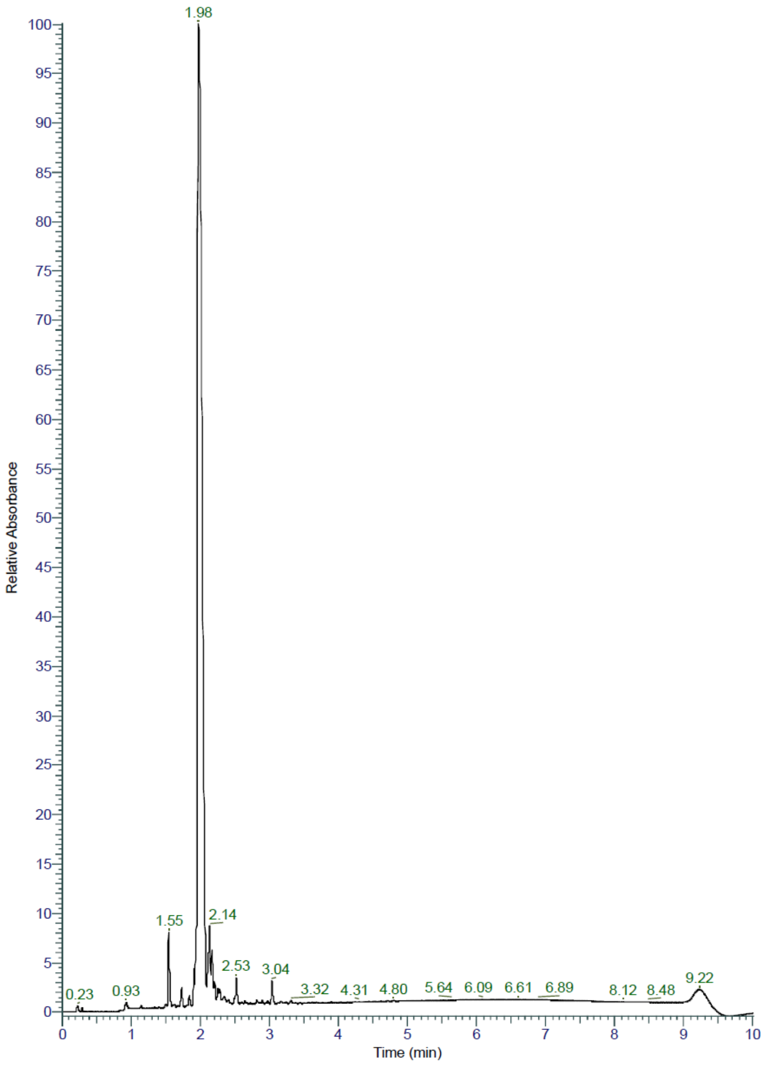


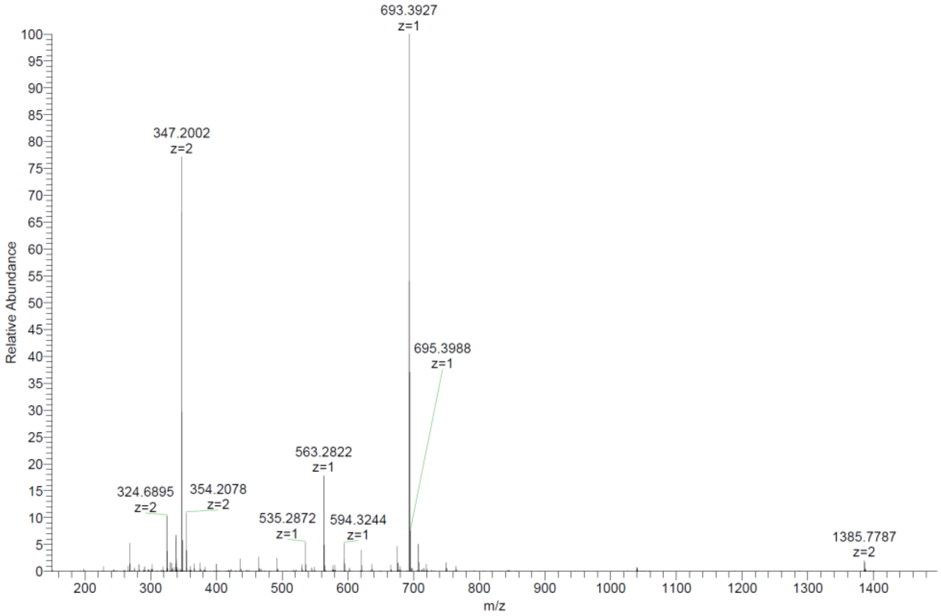

**VKDGYI** (25% v/v Diethylamine, 60°C) was obtained as crude white solid after lyophilization (40.7 mg, 45.7%). Analytical RP-HPLC: $t_R$ = 2.04 min (A/D 100:0 to 0:100 in 7.00 min, λ = 214nm). HRMS (ESI+): $C_{32}H_{52}N_8O_9$ calc./obs. 693.39/693.39 Da $[M+H]^+$.

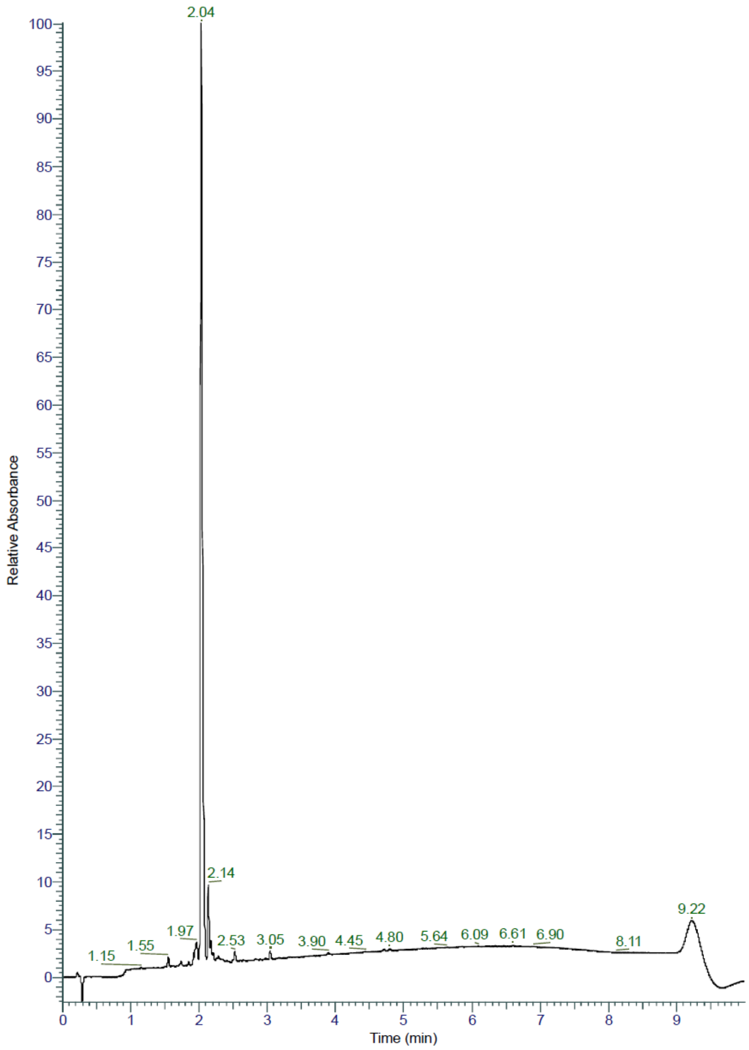


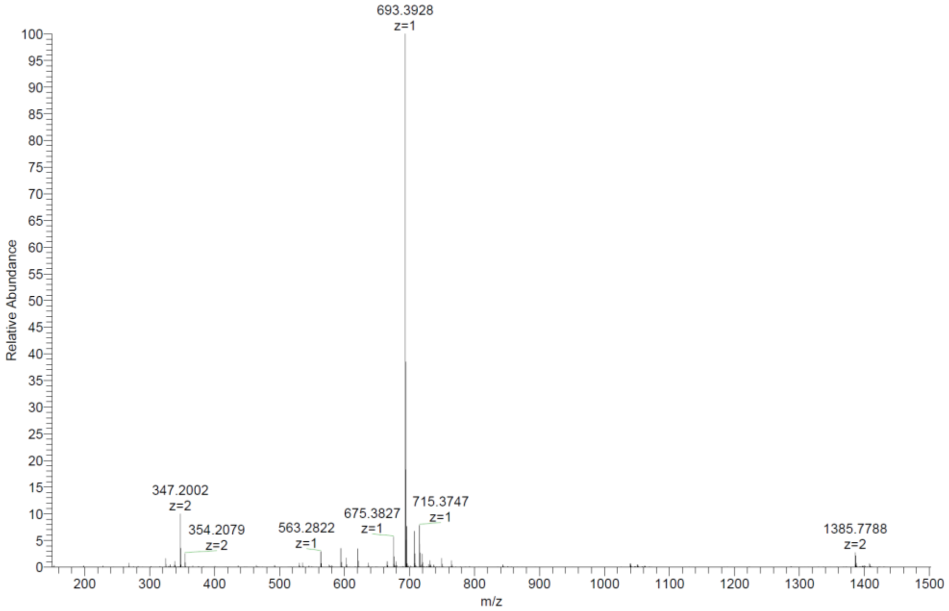

**VKDGYI** (25% v/v Dibutylamine, 60°C) was obtained as crude white solid after lyophilization (45.1 mg, 52.1%). Analytical RP-HPLC: $t_R$ = 2.02 min (A/D 100:0 to 0:100 in 7.00 min, λ = 214nm). HRMS (ESI+): $C_{32}H_{52}N_8O_9$ calc./obs. 693.39/693.39 Da $[M+H]^+$.

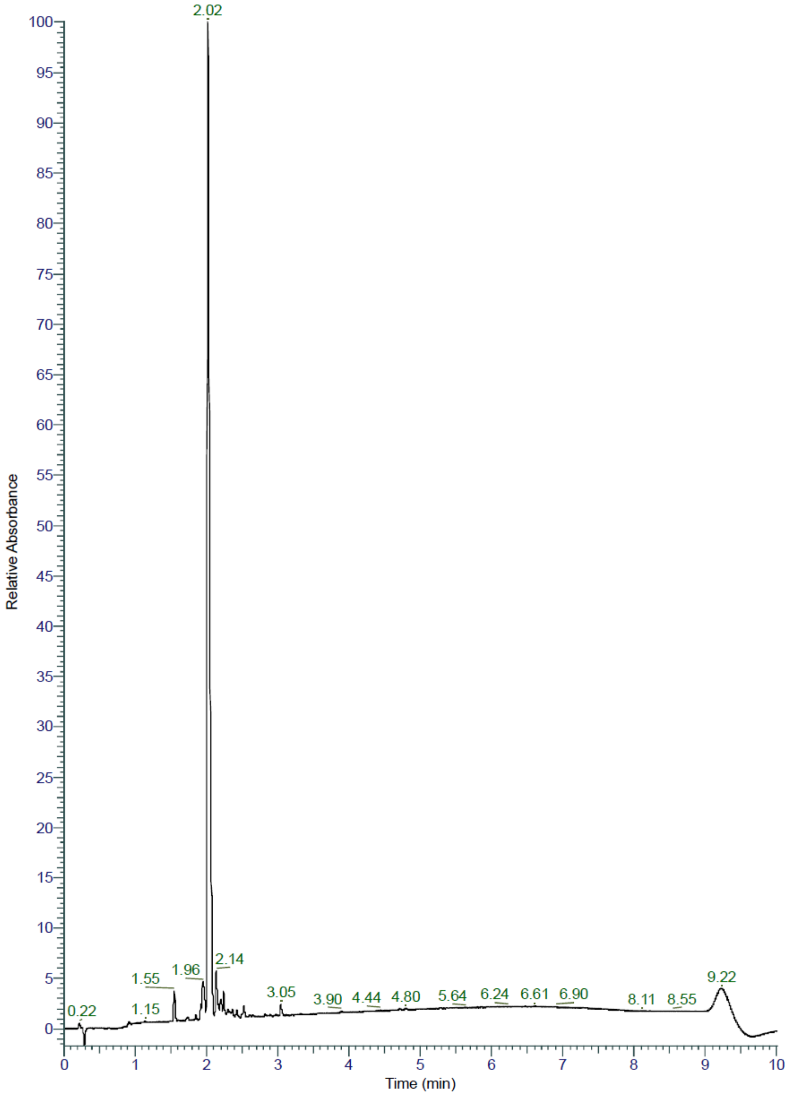


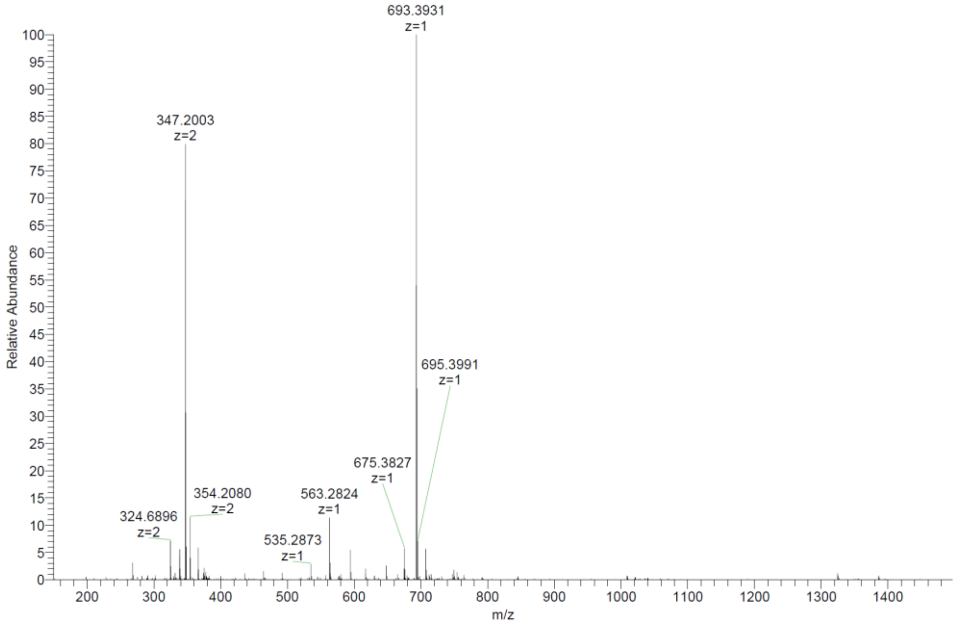

**VKDGYI** (25% v/v Diisobutylamine, 60°C) was obtained as crude white solid after lyophilization (0.4 mg, 0.0%). Analytical RP-HPLC: $t_R$ = - min (A/D 100:0 to 0:100 in 7.00 min, λ = 214nm). HRMS (ESI+): $C_{32}H_{52}N_8O_9$ calc./obs. 693.39/- Da $[M+H]^+$ (no compound observed).

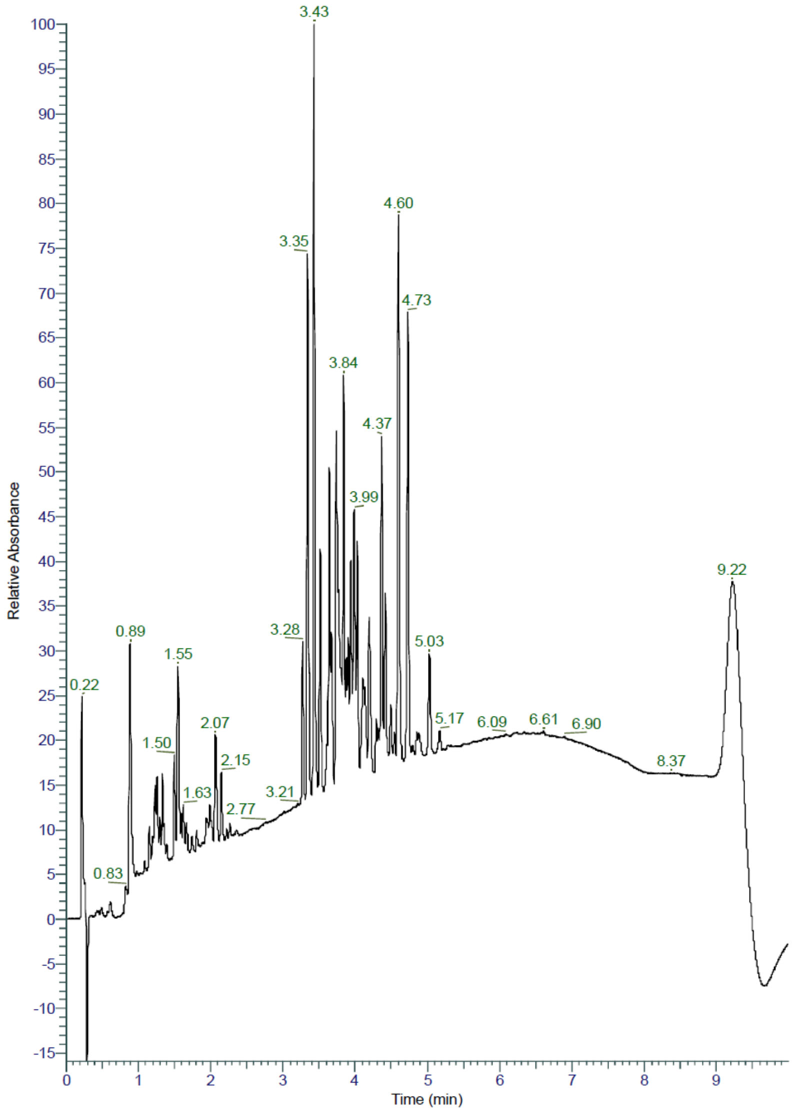


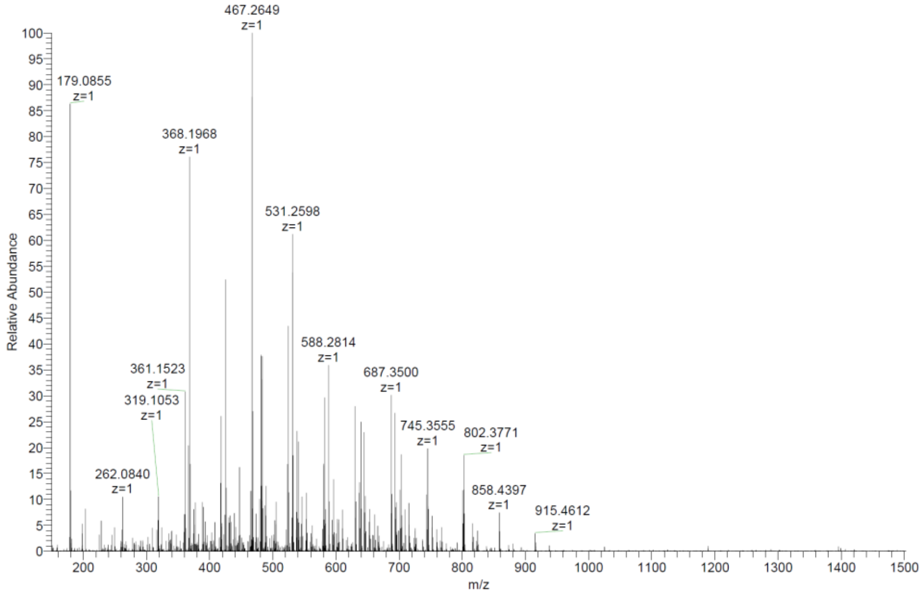

**VKDGYI** (20% v/v Piperidine, 90°C) was obtained as crude white solid after lyophilization (22.8 mg, 28.4%). Analytical RP-HPLC: $t_R$ = 1.99 min (A/D 100:0 to 0:100 in 7.00 min, λ = 214nm). HRMS (ESI+): $C_{32}H_{52}N_8O_9$ calc./obs. 693.39/693.39 Da $[M+H]^+$.

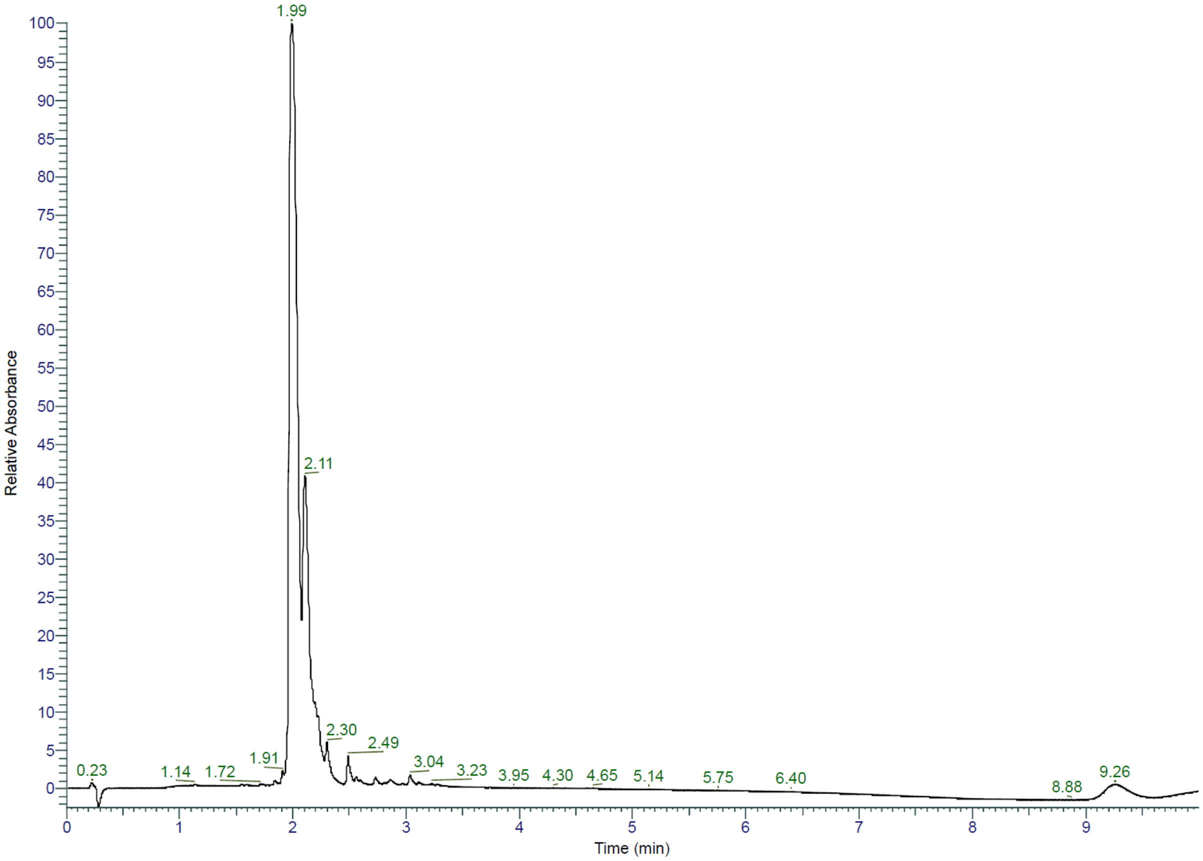


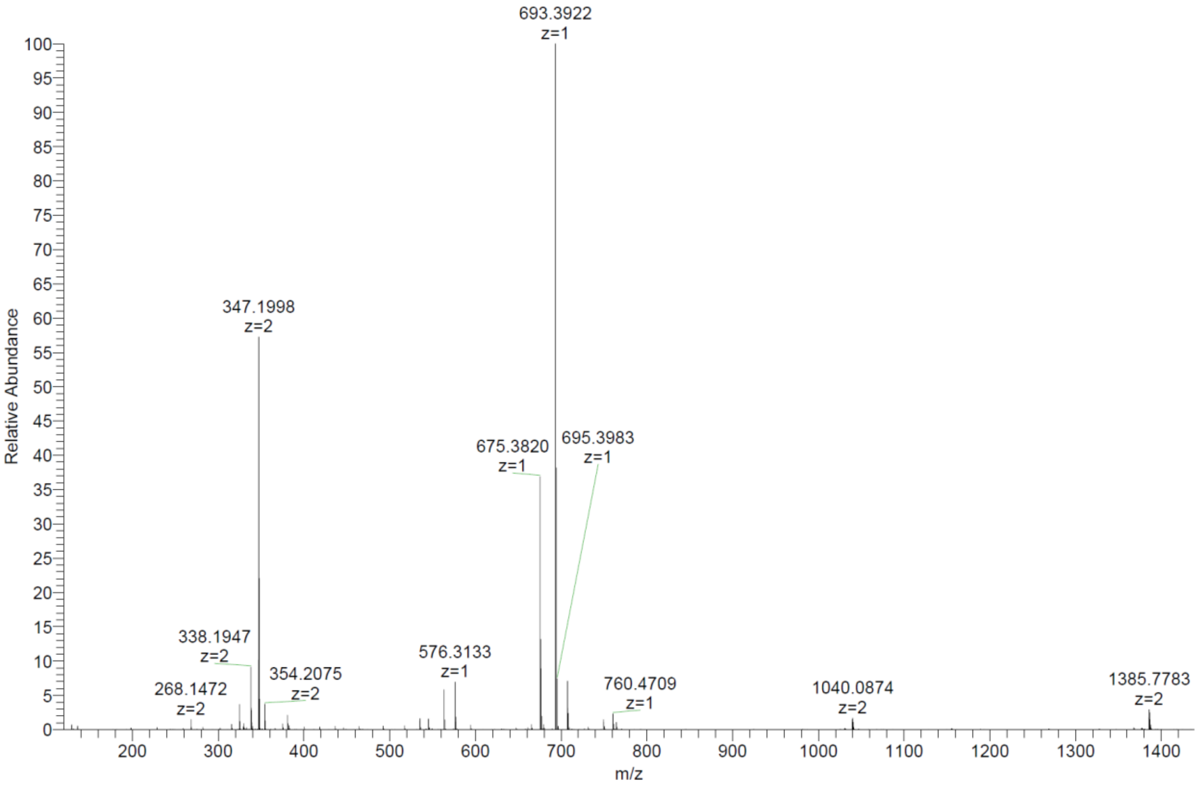

**VKDGYI** (25% v/v Dipropylamine, 90°C) was obtained as crude white solid after lyophilization (26.8 mg, 33.5%). Analytical RP-HPLC: $t_R$ = 2.01 min (A/D 100:0 to 0:100 in 7.00 min, $\lambda$ = 214nm). HRMS (ESI+): $C_{32}H_{52}N_8O_9$ calc./obs. 693.39/693.39 Da $[M+H]^+$.

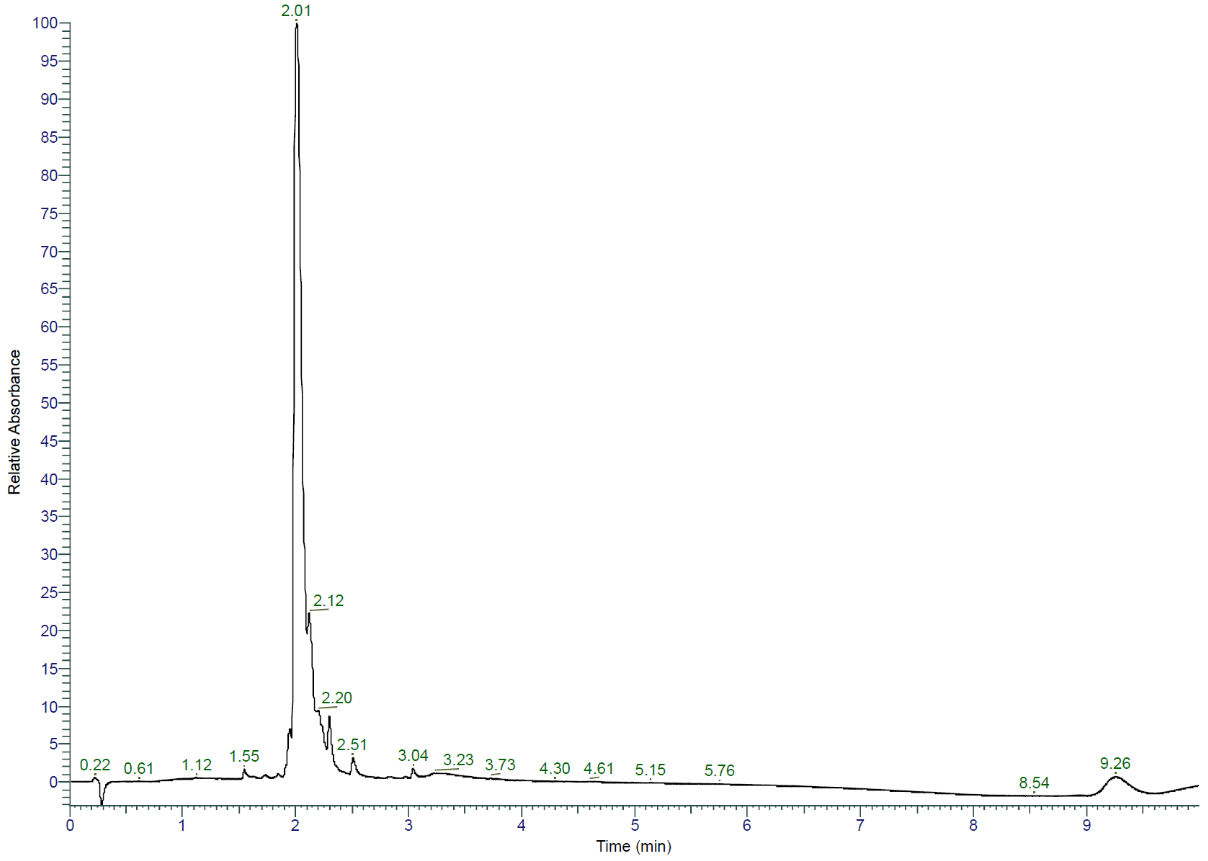


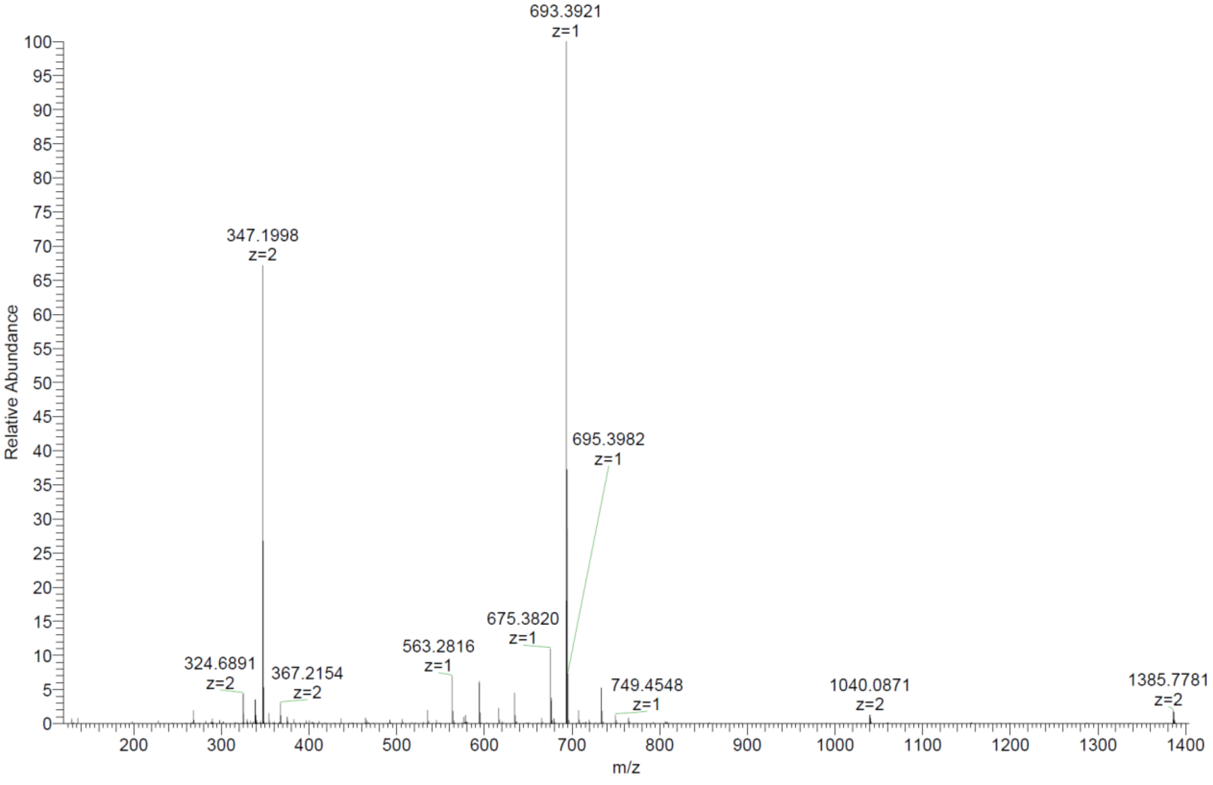

## 2.2 Hexapeptide **1β** (VKD(β)GYI)

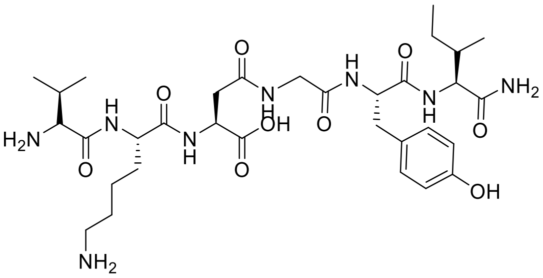


Chemical Formula: $C_{32}H_{52}N_8O_9$
Exact Mass: 692.3857
Molecular Weight: 692.8150

Hexapeptide **1 β (**VKD(β)GYI**)** was obtained as white solid after preparative RP-HPLC (6.4 mg, 14.2%). Analytical RP-HPLC: $t_R$ = 1.19 min (A/D 100:0 to 0:100 in 3.50 min, λ = 214nm). MS (ESI+): $C_{32}H_{52}N_8O_9$ calc./obs. 693.39/693.42 Da $[M+H]^+$.

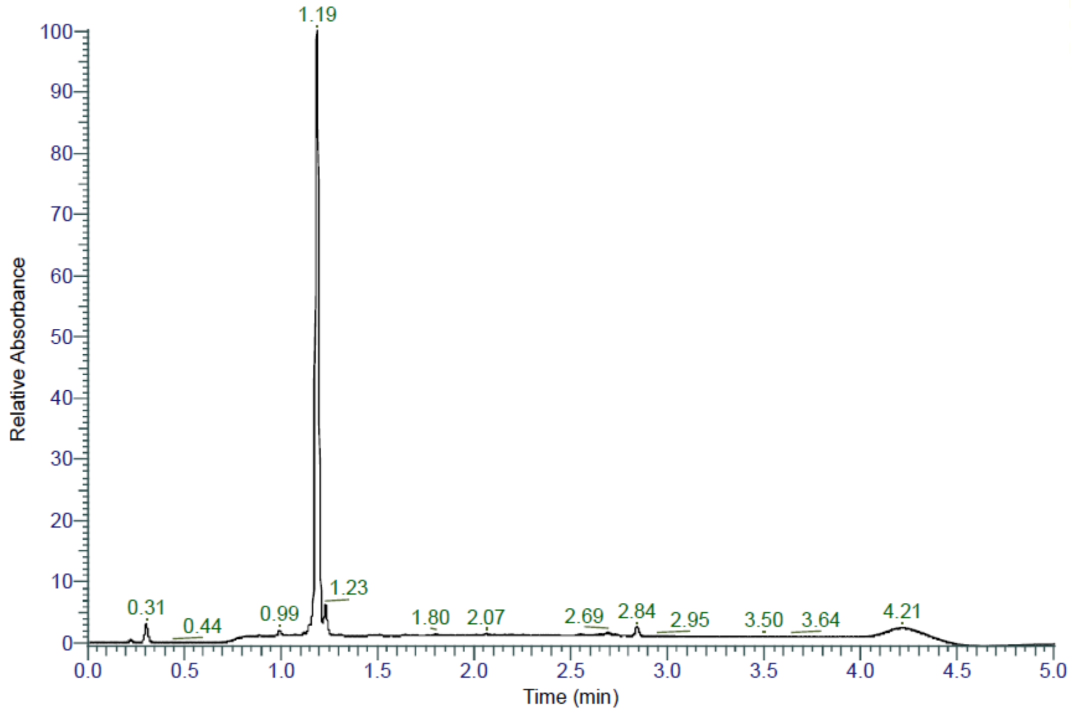


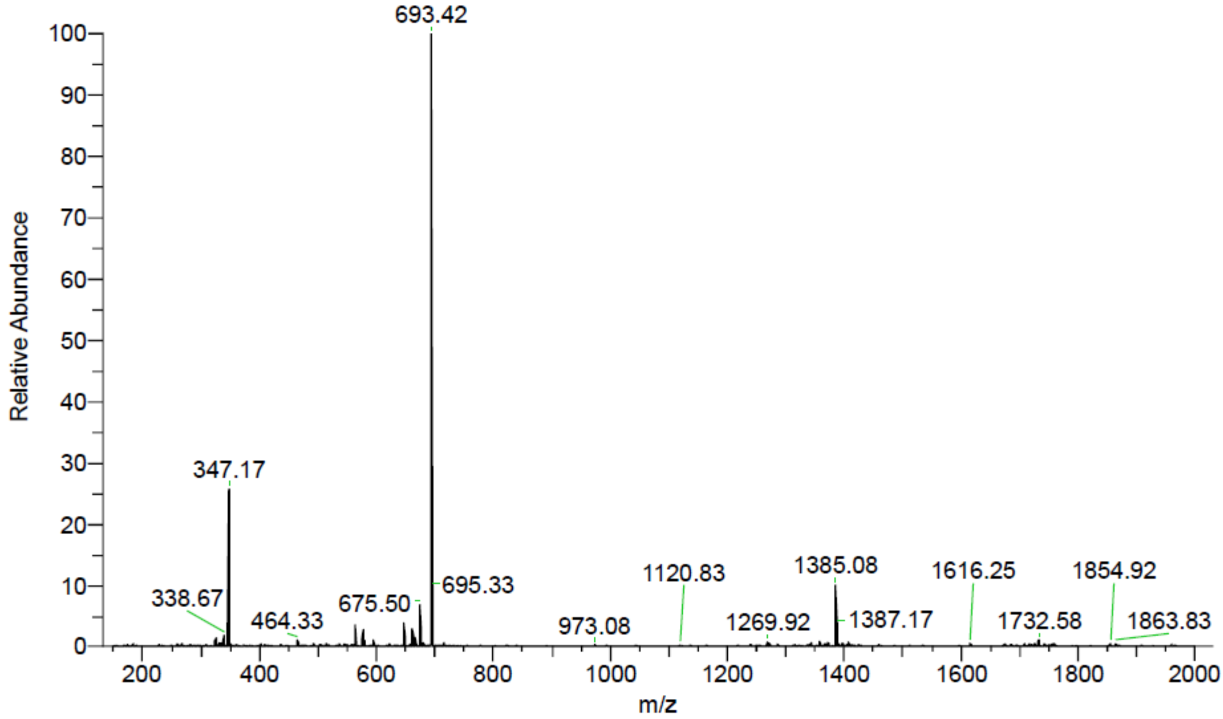

## 2.3 Hexapeptide **2** (GDGAKF)

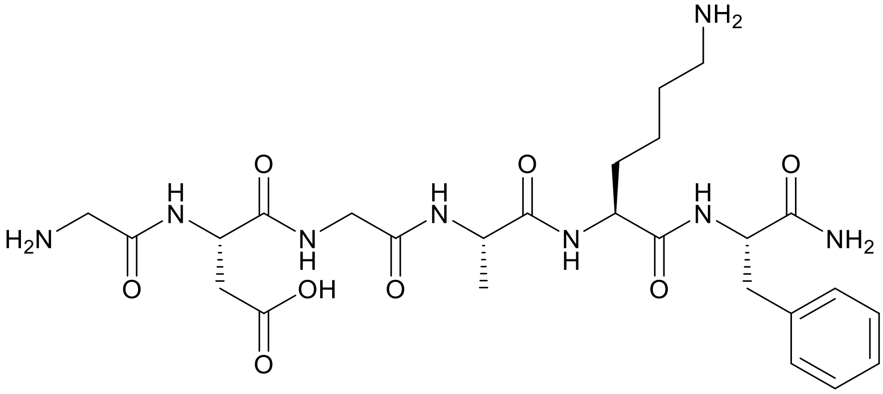


Chemical Formula: $C_{26}H_{40}N_8O_8$
Exact Mass: 592.2969
Molecular Weight: 592.6540

**GDGAKF** (20% v/v Piperidine, 60°C) was obtained as crude white solid after lyophilization (40.6 mg, 40.9%). Analytical RP-HPLC: $t_R$ = 1.75 min (A/D 100:0 to 0:100 in 7.00 min, λ = 214nm). HRMS (ESI+): $C_{26}H_{40}N_8O_8$ calc./obs. 593.30/593.30 Da $[M+H]^+$.

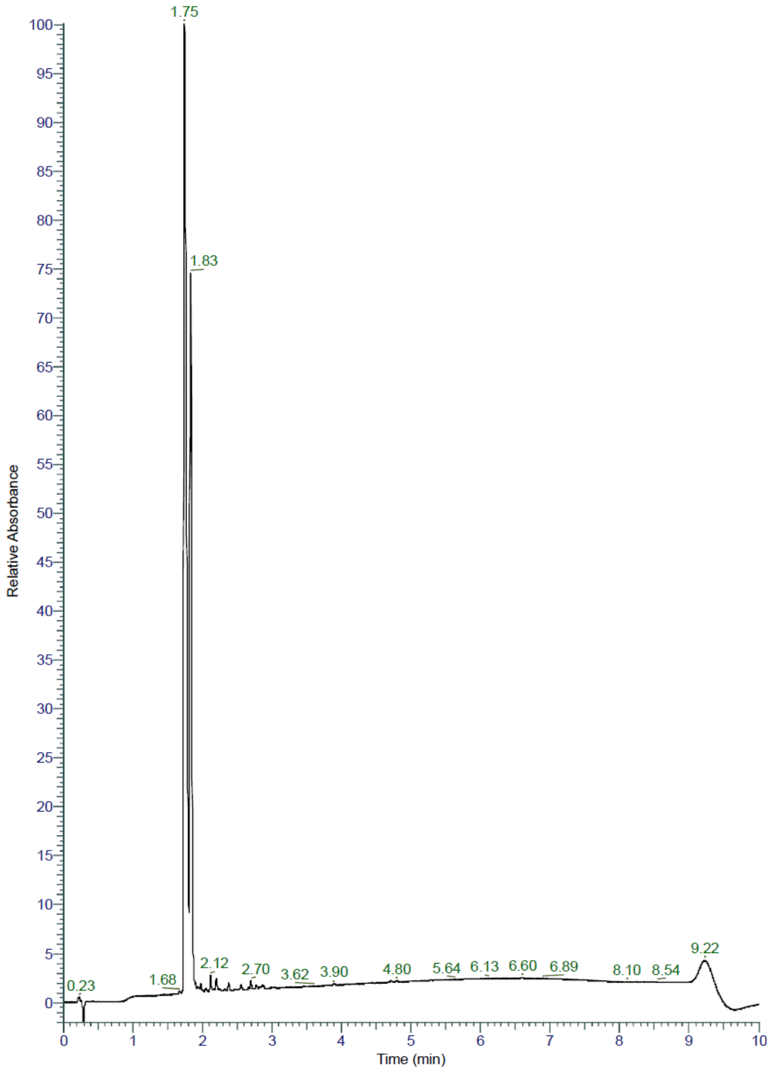


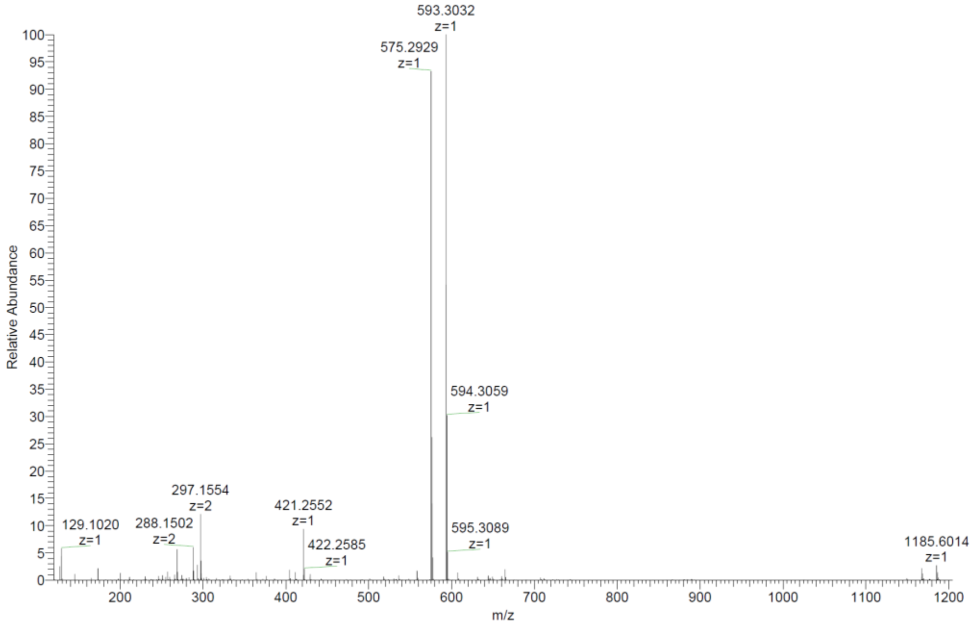

**GDGAKF** (25% v/v Dipropylamine, 60°C) was obtained as crude white solid after lyophilization (38.9 mg, 49.2%). Analytical RP-HPLC: $t_R$ = 1.76 min (A/D 100:0 to 0:100 in 7.00 min, λ = 214nm). HRMS (ESI+): $C_{26}H_{40}N_8O_8$ calc./obs. 593.30/593.30 Da $[M+H]^+$.

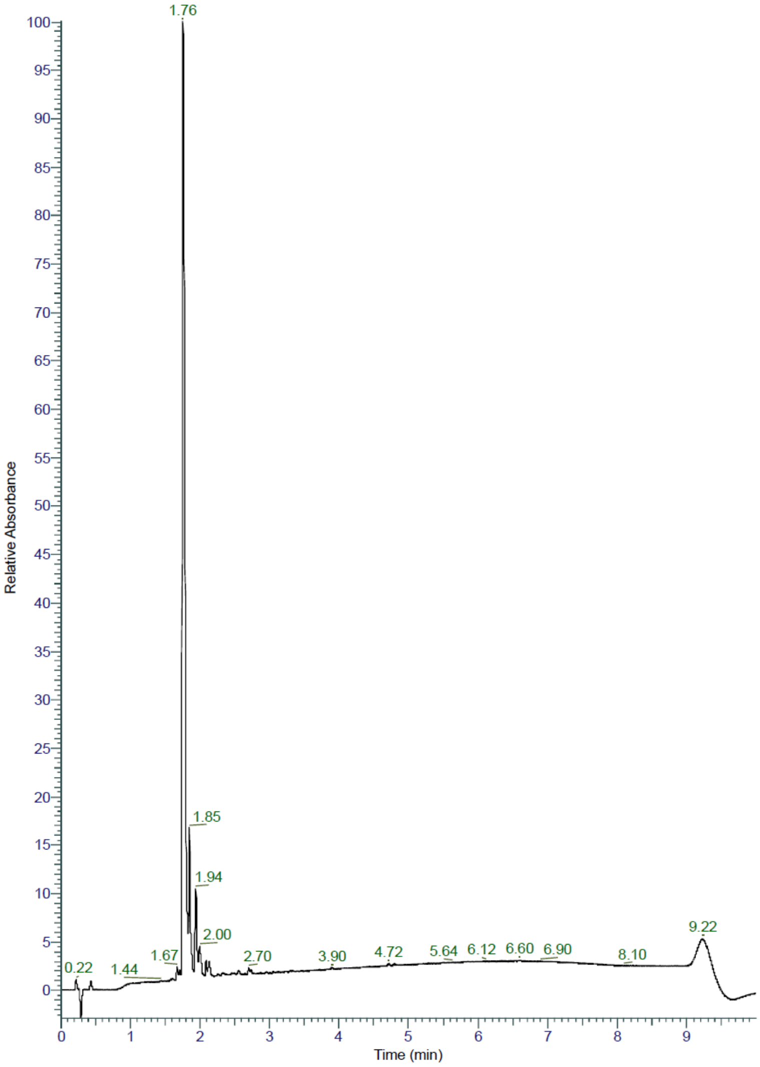


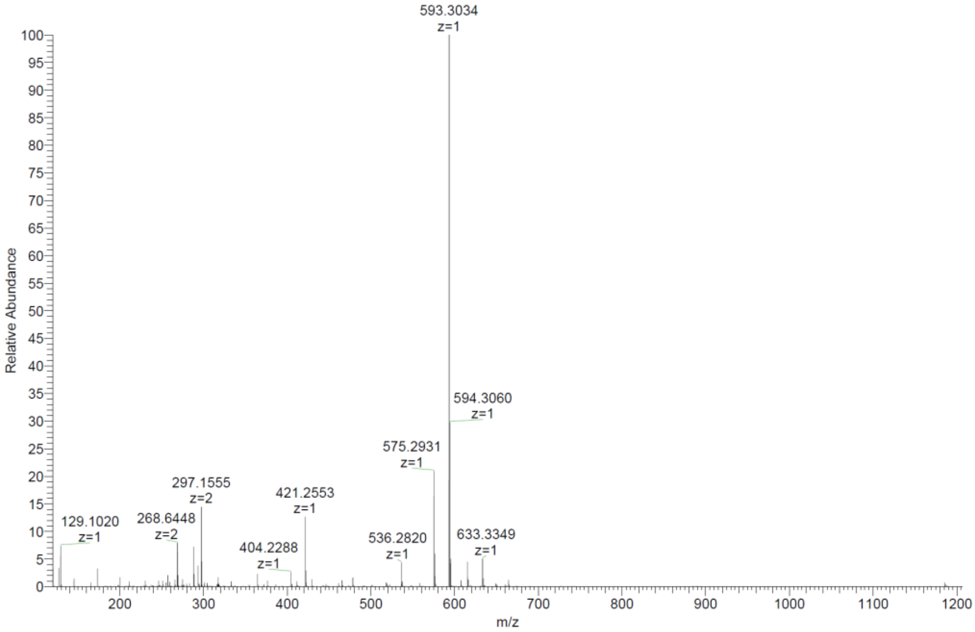

## 2.4 Hexapeptide **3** (VKDRYI)

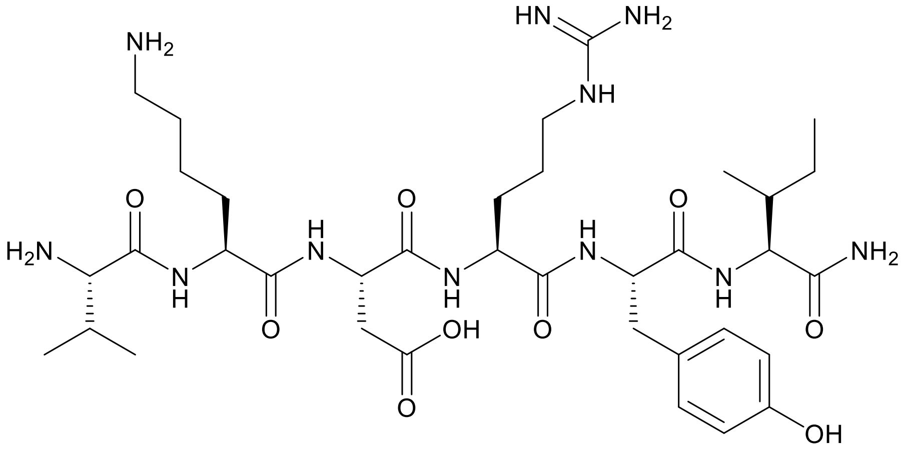


Chemical Formula: $C_{36}H_{61}N_{11}O_9$
Exact Mass: 791.4654
Molecular Weight: 791.9520

**VKDRYI** (20% v/v Piperidine, 60°C) was obtained as crude white solid after lyophilization (44.0 mg, 40.3%). Analytical RP-HPLC: $t_R$ = 1.99 min (A/D 100:0 to 0:100 in 7.00 min, λ = 214nm). HRMS (ESI+): $C_{36}H_{61}N_{11}O_9$ calc./obs. 792.47/792.47 Da $[M+H]^+$.

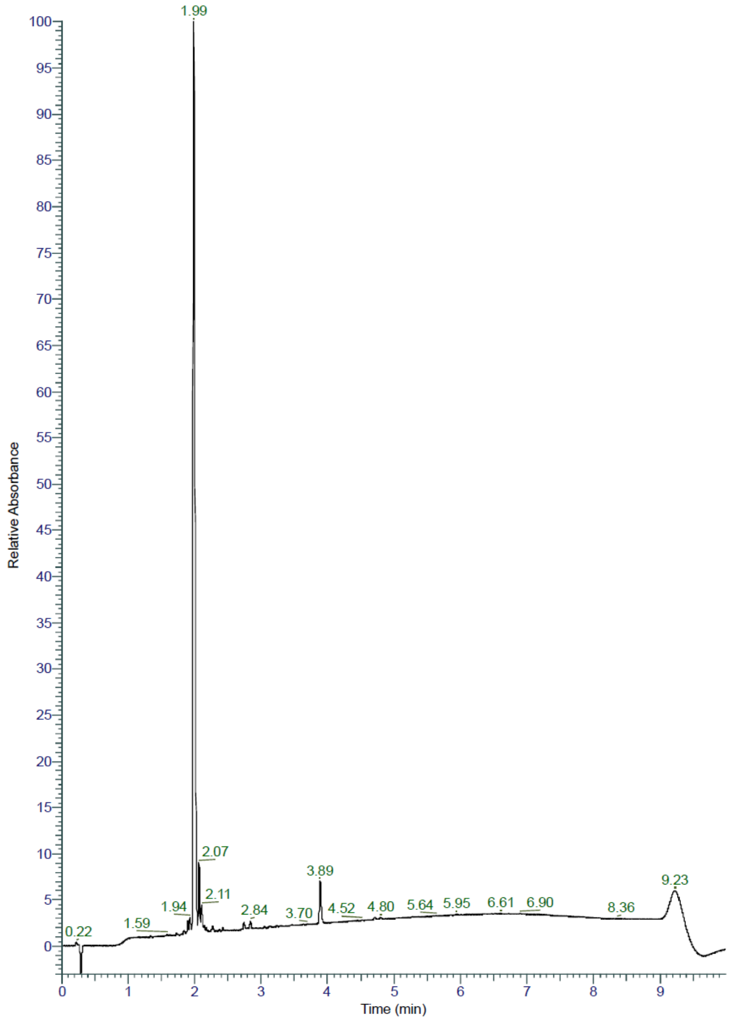


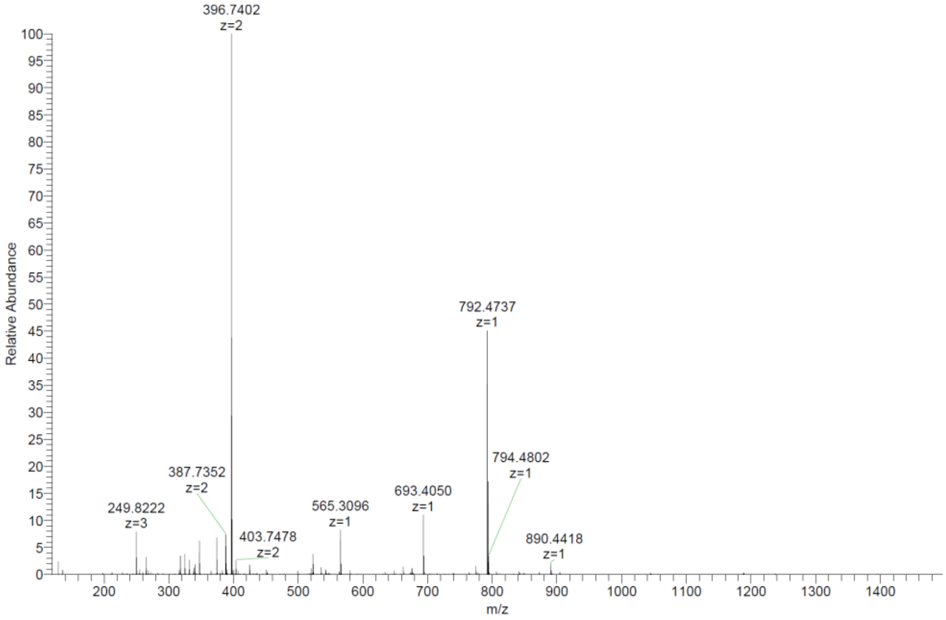

**VKDRYI** (25% v/v Dipropylamine, 60°C) was obtained as crude white solid after lyophilization (44.2 mg, 43.4%). Analytical RP-HPLC: $t_R$ = 2.00 min (A/D 100:0 to 0:100 in 7.00 min, λ = 214nm). HRMS (ESI+): $C_{36}H_{61}N_{11}O_9$ calc./obs. 792.47/792.47 Da $[M+H]^+$.

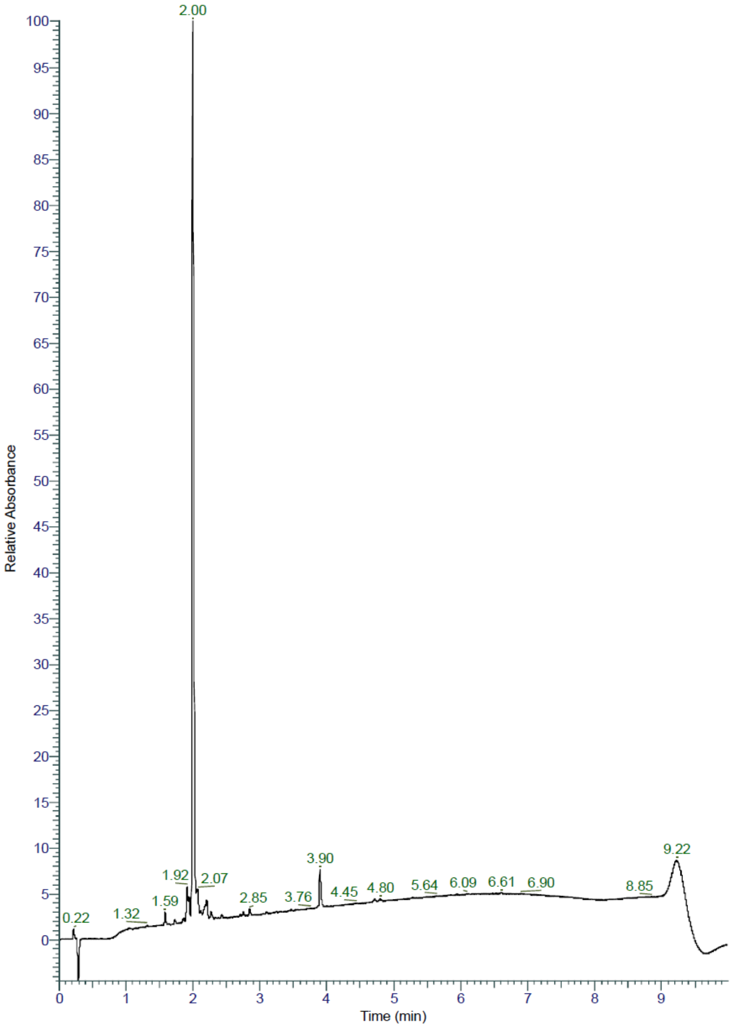


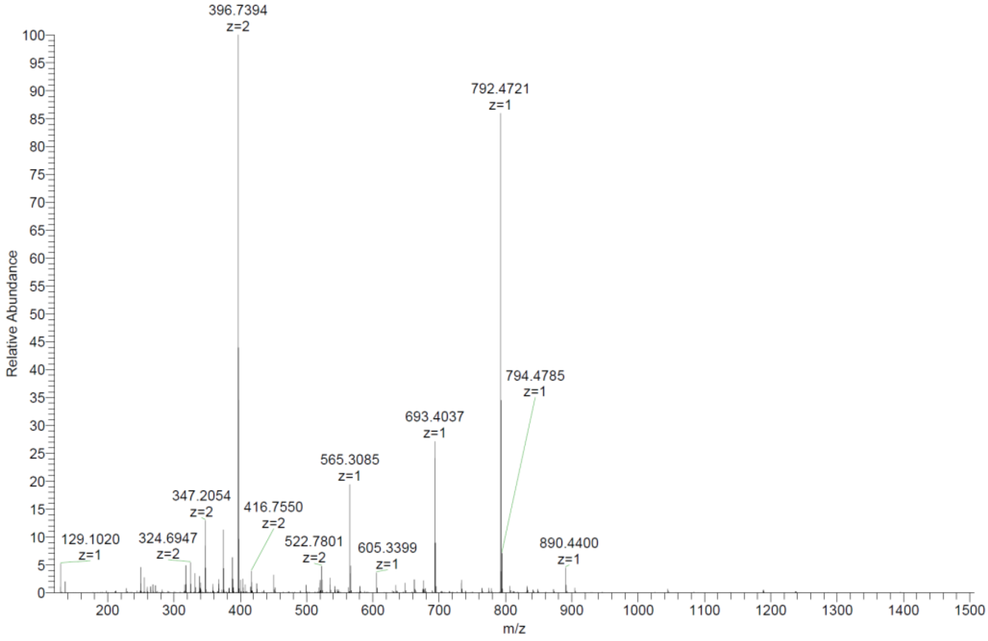

2.5 Hexapeptide **4** (GDRAKF)

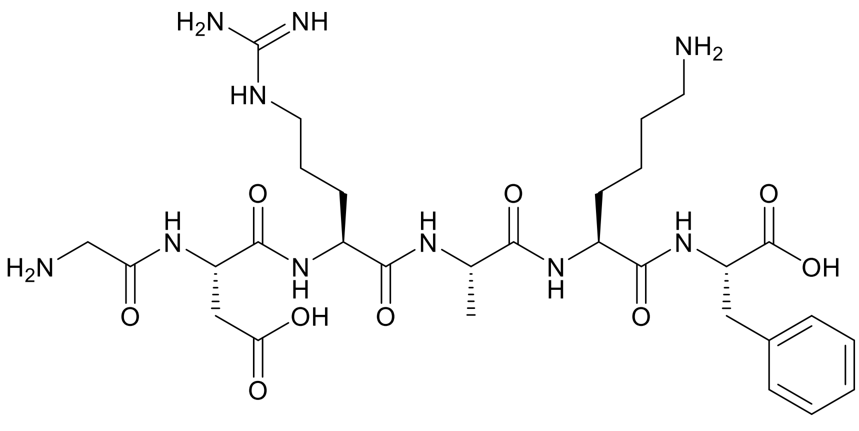


Chemical Formula: $C_{30}H_{48}N_{10}O_9$
Exact Mass: 692.3606
Molecular Weight: 692.7750

**GDRAKF** (20% v/v Piperidine, 60°C) was obtained as crude white solid after lyophilization (44.2 mg, 50.6%). Analytical RP-HPLC: $t_R$ = 1.84 min (A/D 100:0 to 0:100 in 7.00 min, λ = 214nm). HRMS (ESI+): $C_{30}H_{48}N_{10}O_9$ calc./obs. 693.36/693.39Da $[M+H]^+$.

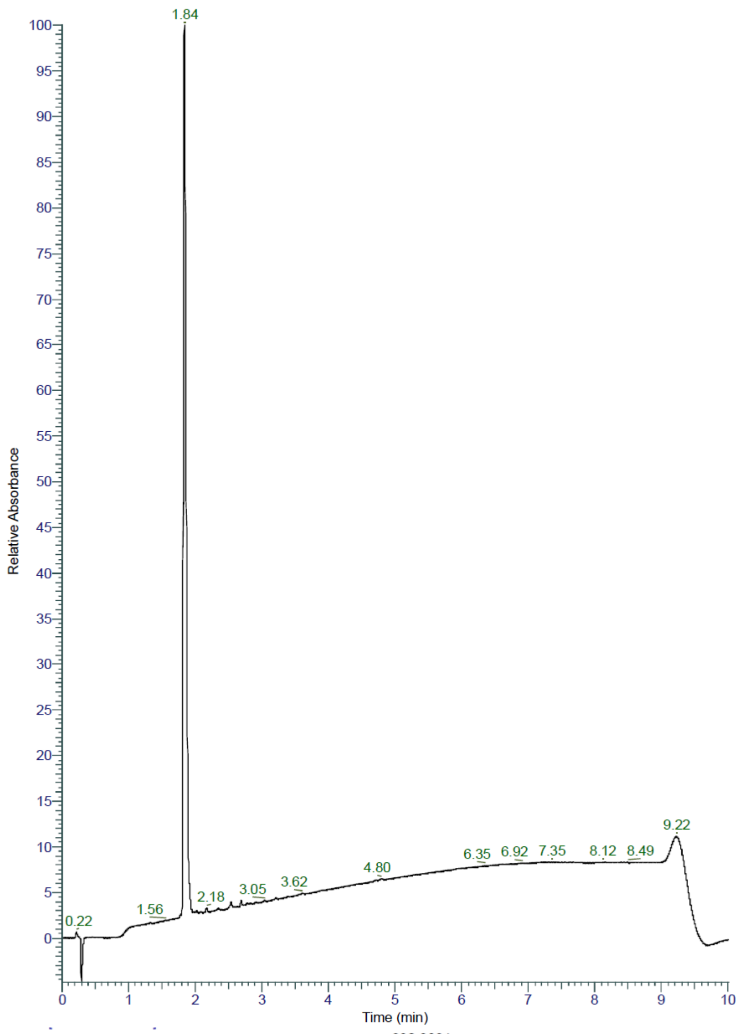


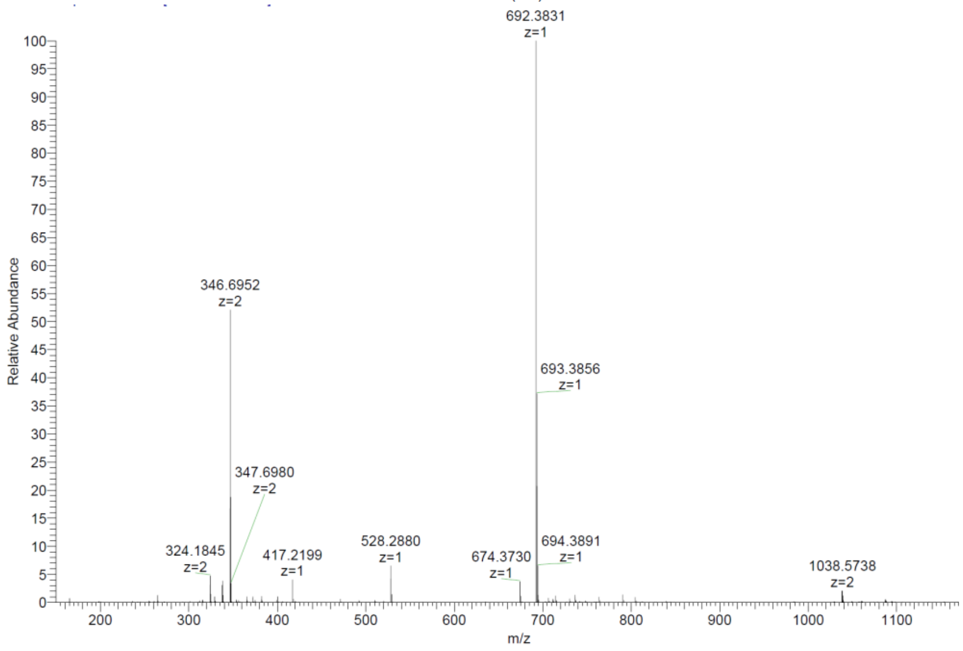

**GDRAKF** (25% v/v Dipropylamine, 60°C) was obtained as crude white solid after lyophilization (52.9 mg, 62.5%). Analytical RP-HPLC: $t_R$ = 1.86 min (A/D 100:0 to 0:100 in 7.00 min, λ = 214nm). HRMS (ESI+): $C_{30}H_{48}N_{10}O_9$ calc./obs. 693.36/693.39Da $[M+H]^+$.

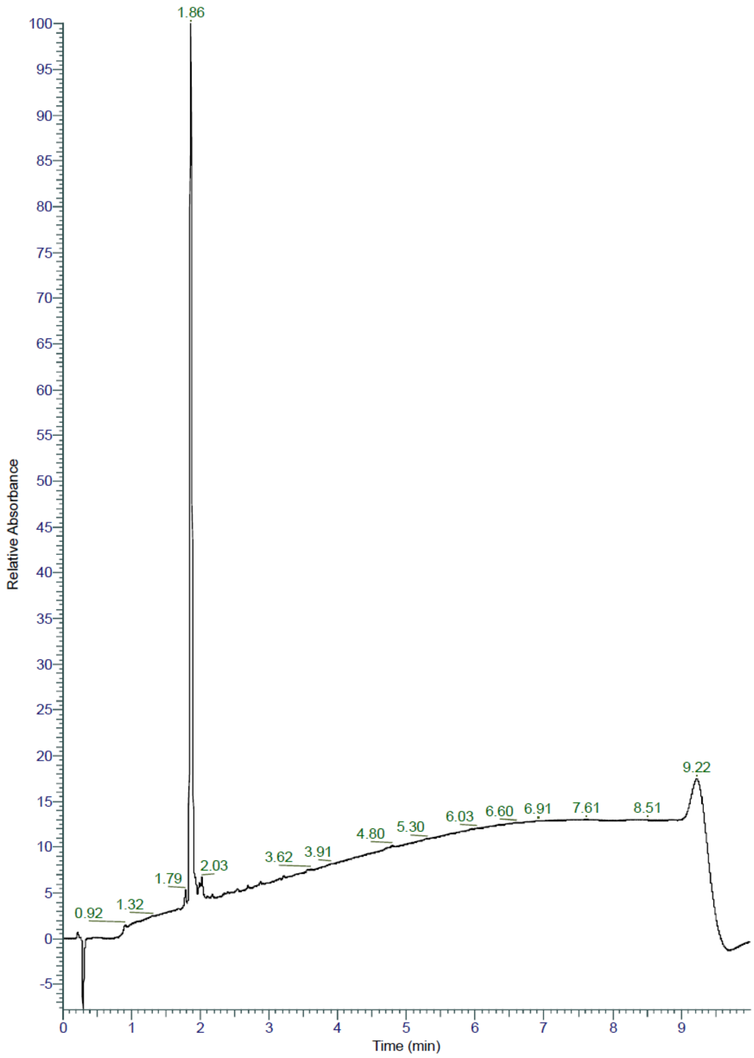


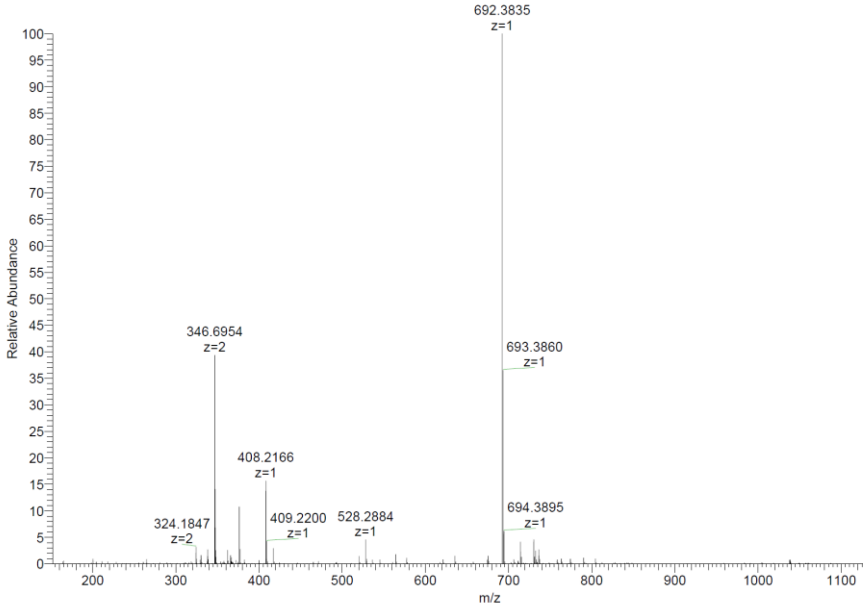

## 2.6 Hexapeptide **5** (VKDCYI)

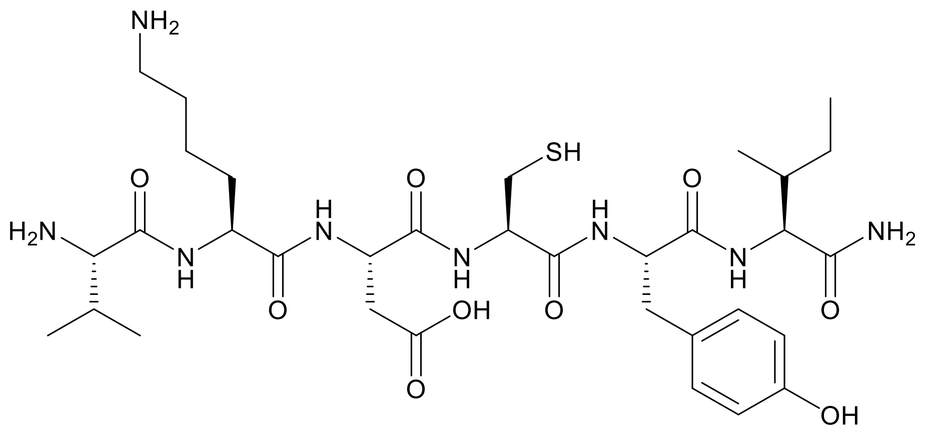


Chemical Formula: $C_{33}H_{54}N_8O_9S$
Exact Mass: 738.3734
Molecular Weight: 738.9020

**VKDCYI** (20% v/v Piperidine, 60°C) was obtained as crude white solid after lyophilization (46.2 mg, 53.1%). Analytical RP-HPLC: $t_R$ = 2.23 min (A/D 100:0 to 0:100 in 7.00 min, λ = 214nm). HRMS (ESI+): $C_{33}H_{54}N_8O_9S$ calc./obs. 739.37/739.38 Da $[M+H]^+$.

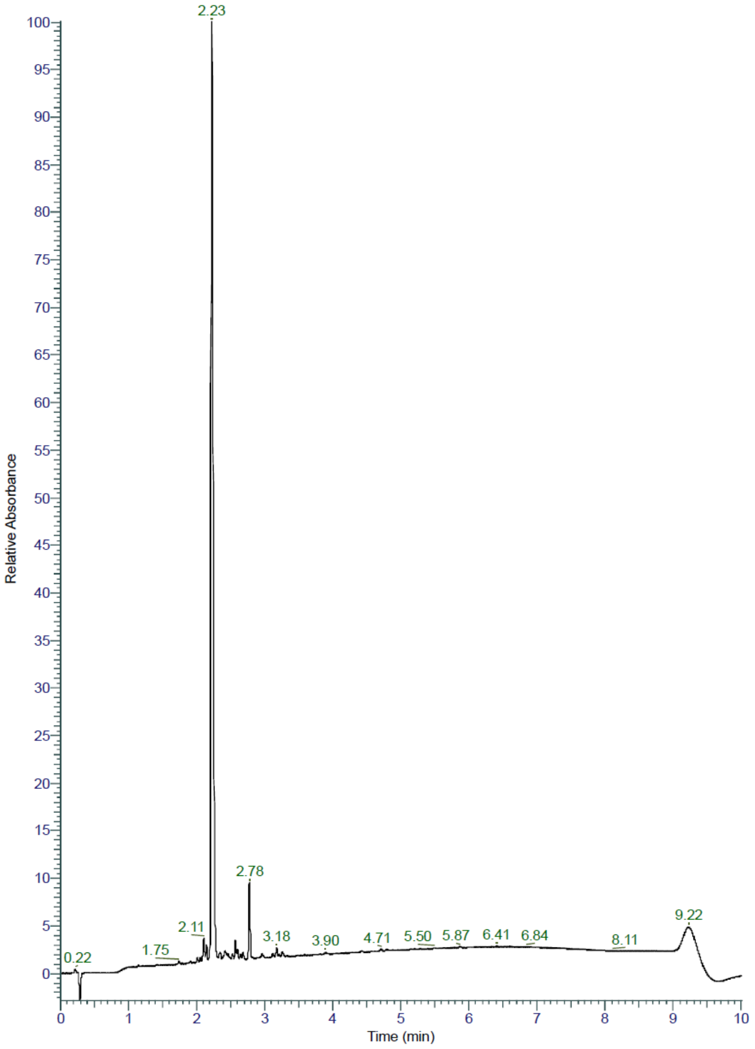


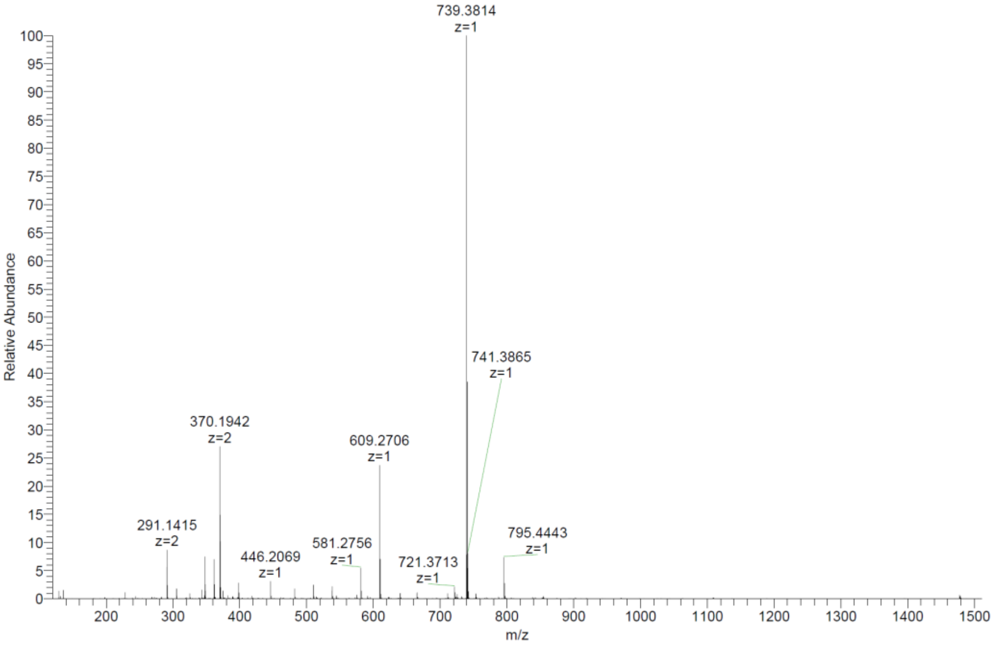

**VKDCYI** (25% v/v Dipropylamine, 60°C) was obtained as crude white solid after lyophilization (42.7 mg, 48.0%). Analytical RP-HPLC: $t_R$ = 2.23 min (A/D 100:0 to 0:100 in 7.00 min, λ = 214nm). HRMS (ESI+): $C_{33}H_{54}N_8O_9S$ calc./obs. 739.37/739.38 Da $[M+H]^+$.

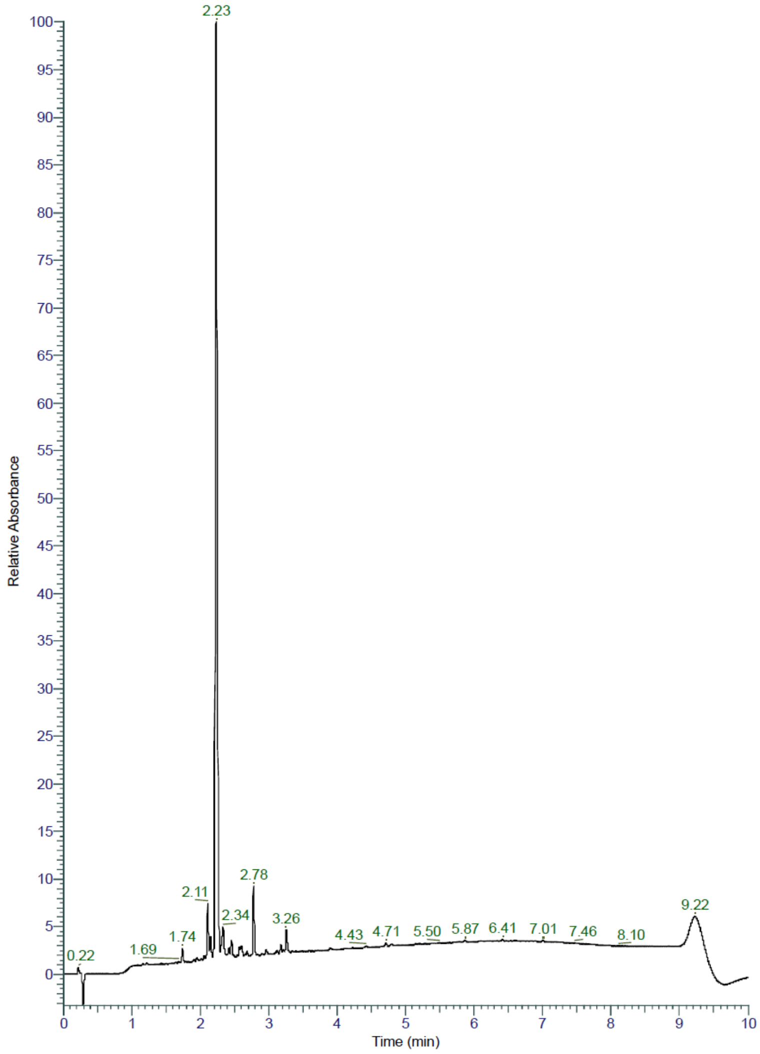


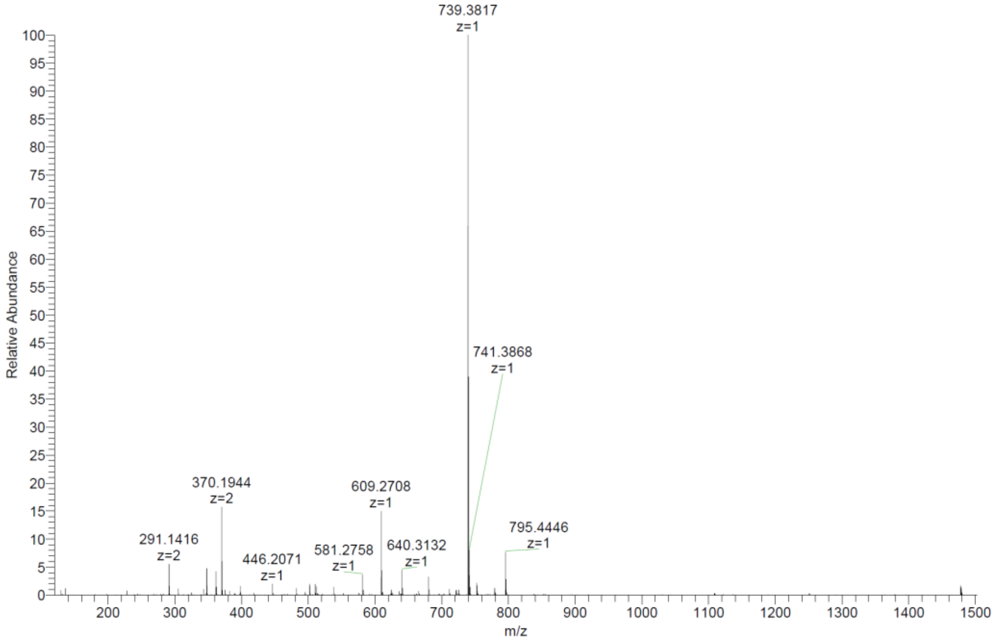

## 2.7 Hexapeptide **6** (VKDAYI)

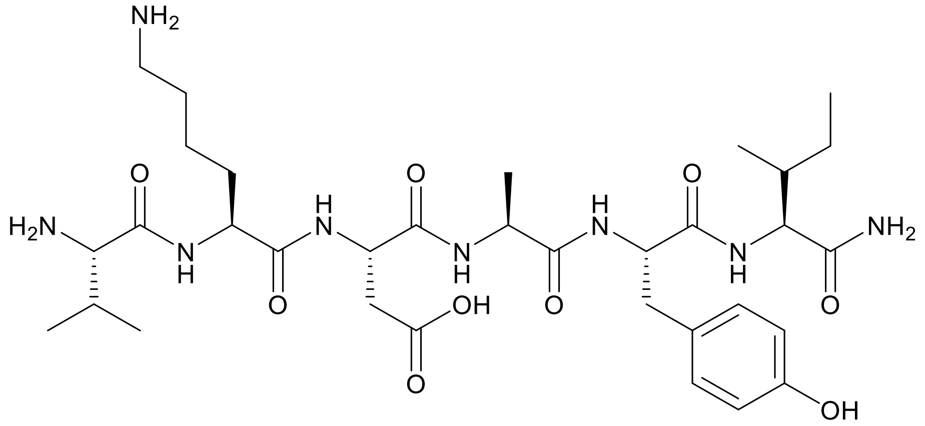


Chemical Formula: $C_{33}H_{54}N_8O_9$
Exact Mass: 706.4014
Molecular Weight: 706.8420

**VKDAYI** (20% v/v Piperidine, 60°C) was obtained as crude white solid after lyophilization (42.7 mg, 54.7%). Analytical RP-HPLC: $t_R$ = 2.09 min (A/D 100:0 to 0:100 in 7.00 min, λ = 214nm). HRMS (ESI+): $C_{33}H_{54}N_8O_9$ calc./obs. 707.40/707.41 Da $[M+H]^+$.

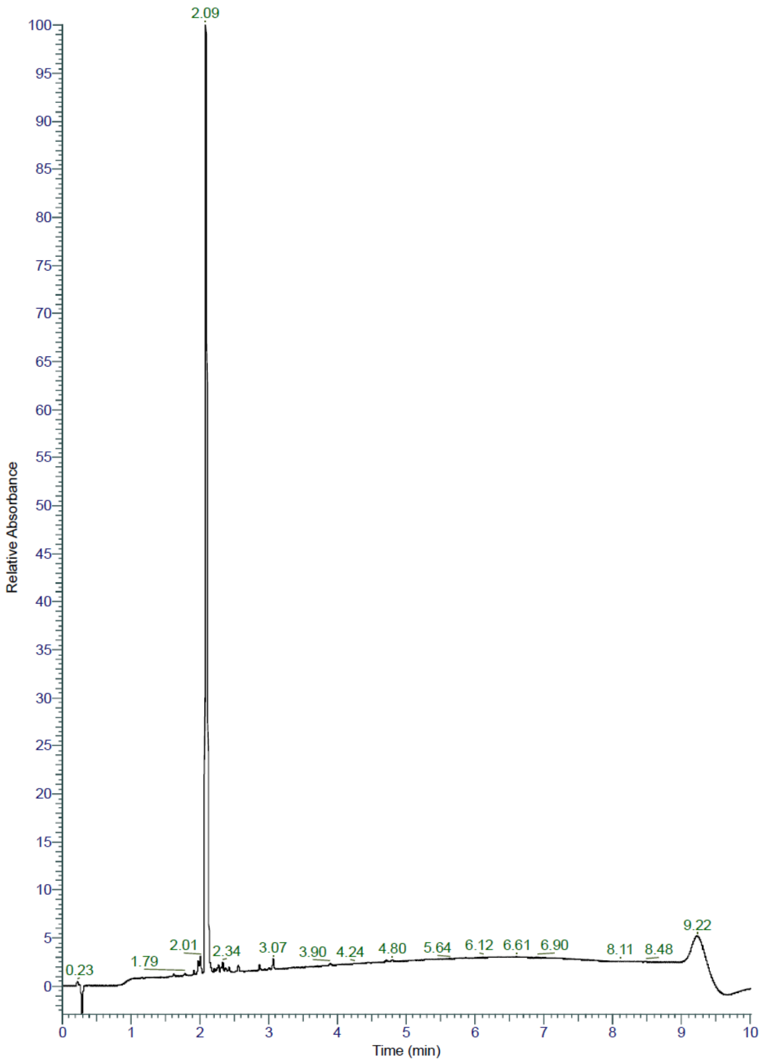


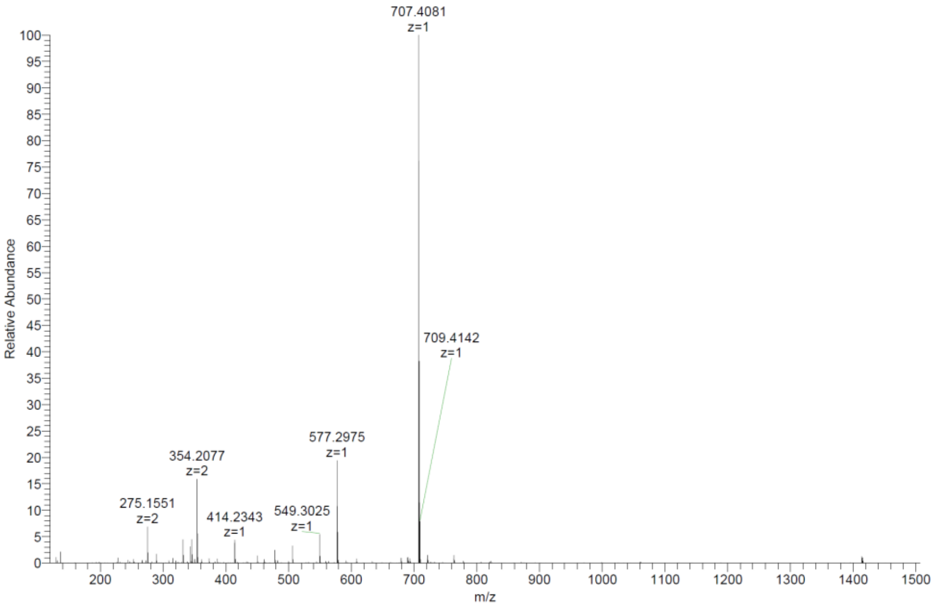

**VKDAYI** (25% v/v Dipropylamine, 60°C) was obtained as crude white solid after lyophilization (40.4 mg, 51.3%). Analytical RP-HPLC: $t_R$ = 2.09 min (A/D 100:0 to 0:100 in 7.00 min, λ = 214nm). HRMS (ESI+): $C_{33}H_{54}N_8O_9$ calc./obs. 707.40/ Da $[M+H]^+$.

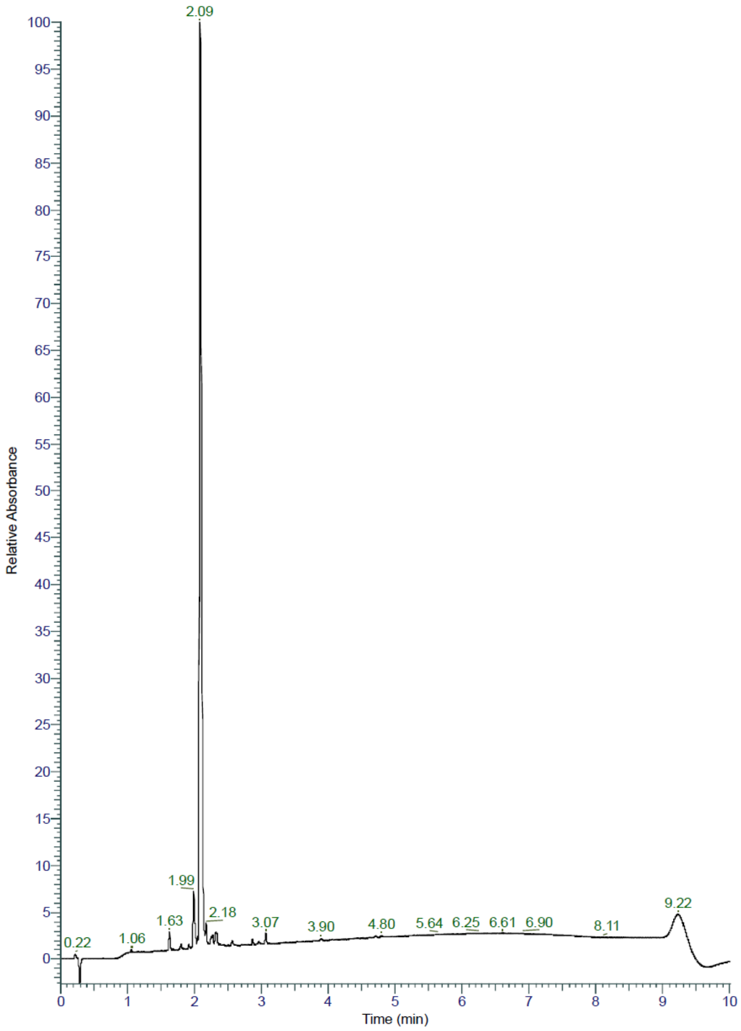


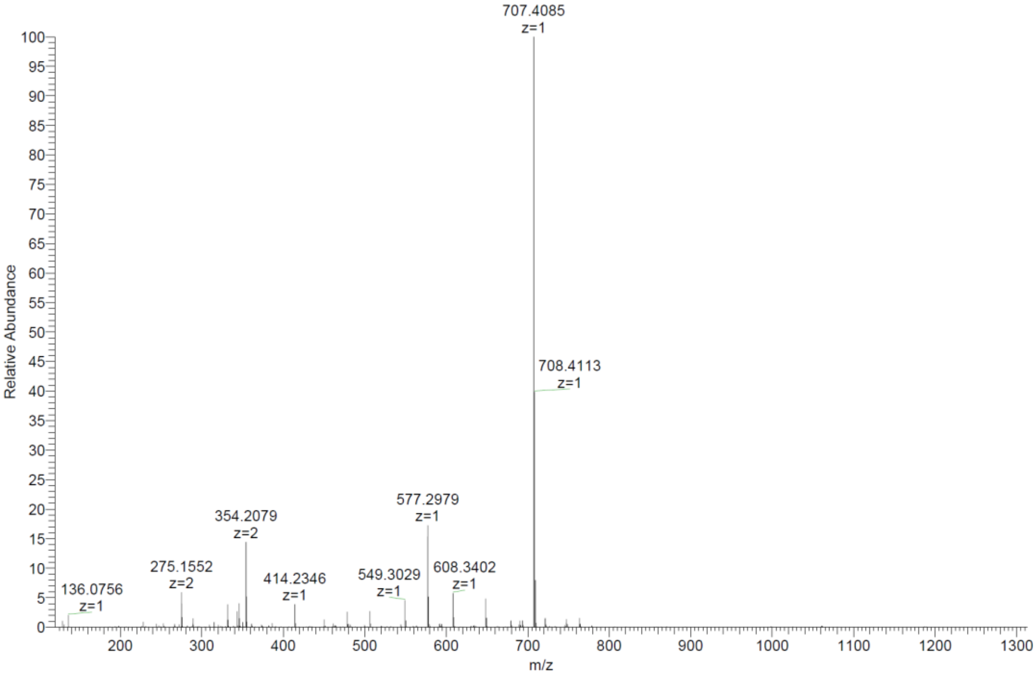

## 2.8 Hexapeptide **7** (VKEGYI)

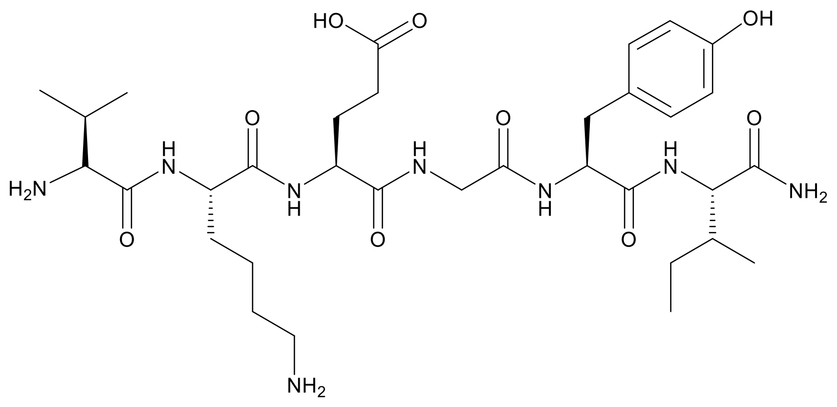


Chemical Formula: $C_{33}H_{54}N_8O_9$
Exact Mass: 706.4014
Molecular Weight: 706.8420

**VKEGYI** (20% v/v Piperidine, 60°C) was obtained as crude white solid after lyophilization (34.8 mg, 47.7%). Analytical RP-HPLC: $t_R$ = 1.99 min (A/D 100:0 to 0:100 in 7.00 min, λ = 214nm). HRMS (ESI+): $C_{33}H_{54}N_8O_9$ calc./obs. 707.40/707.41 Da $[M+H]^+$.

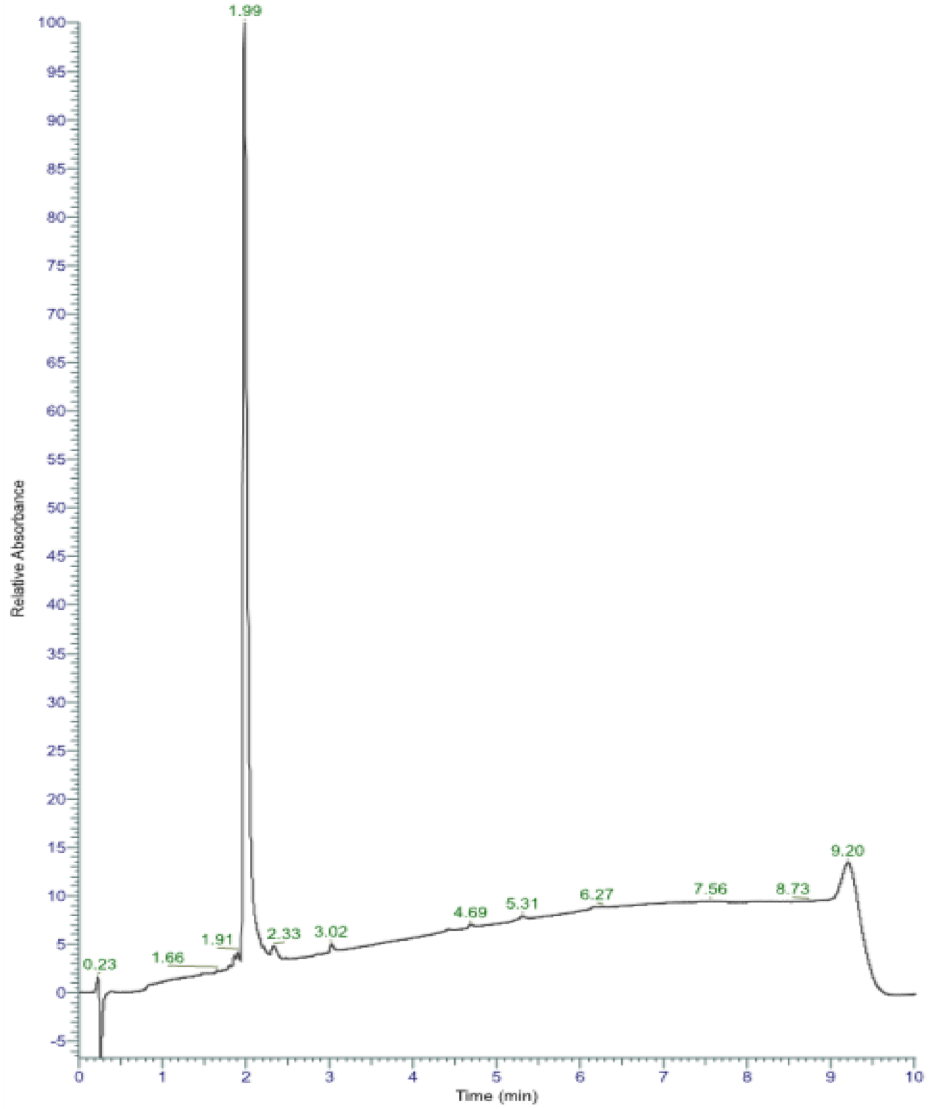


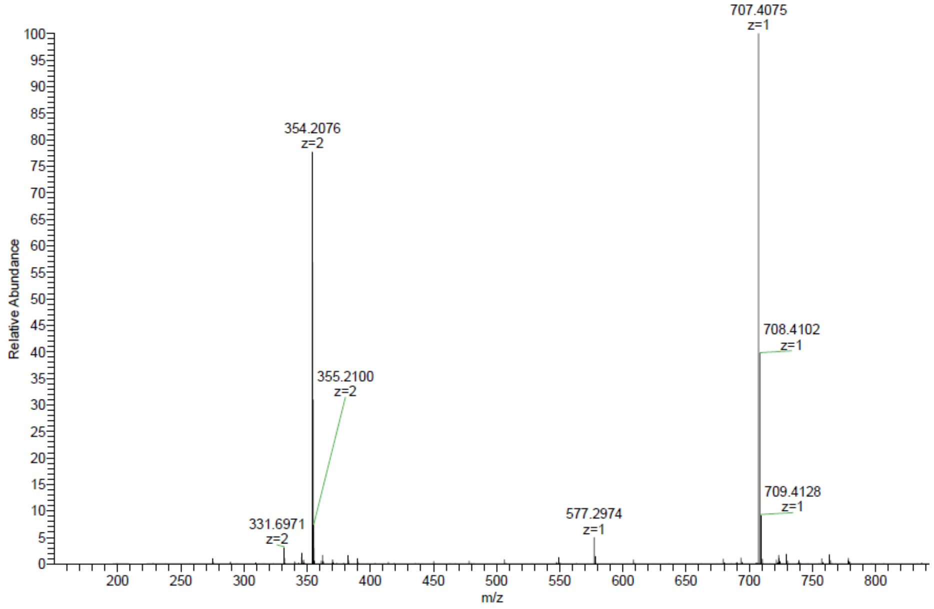

**VKEGYI** (5% w/v Piperazine + 2% v/v DBU, 60°C) was obtained as crude white solid after lyophilization (38.2 mg, 52.4%). Analytical RP-HPLC: $t_R$ = 1.90 min (A/D 100:0 to 0:100 in 7.00 min, λ = 214nm). HRMS (ESI+): $C_{33}H_{54}N_8O_9$ calc./obs. 707.40/707.41 Da $[M+H]^+$.

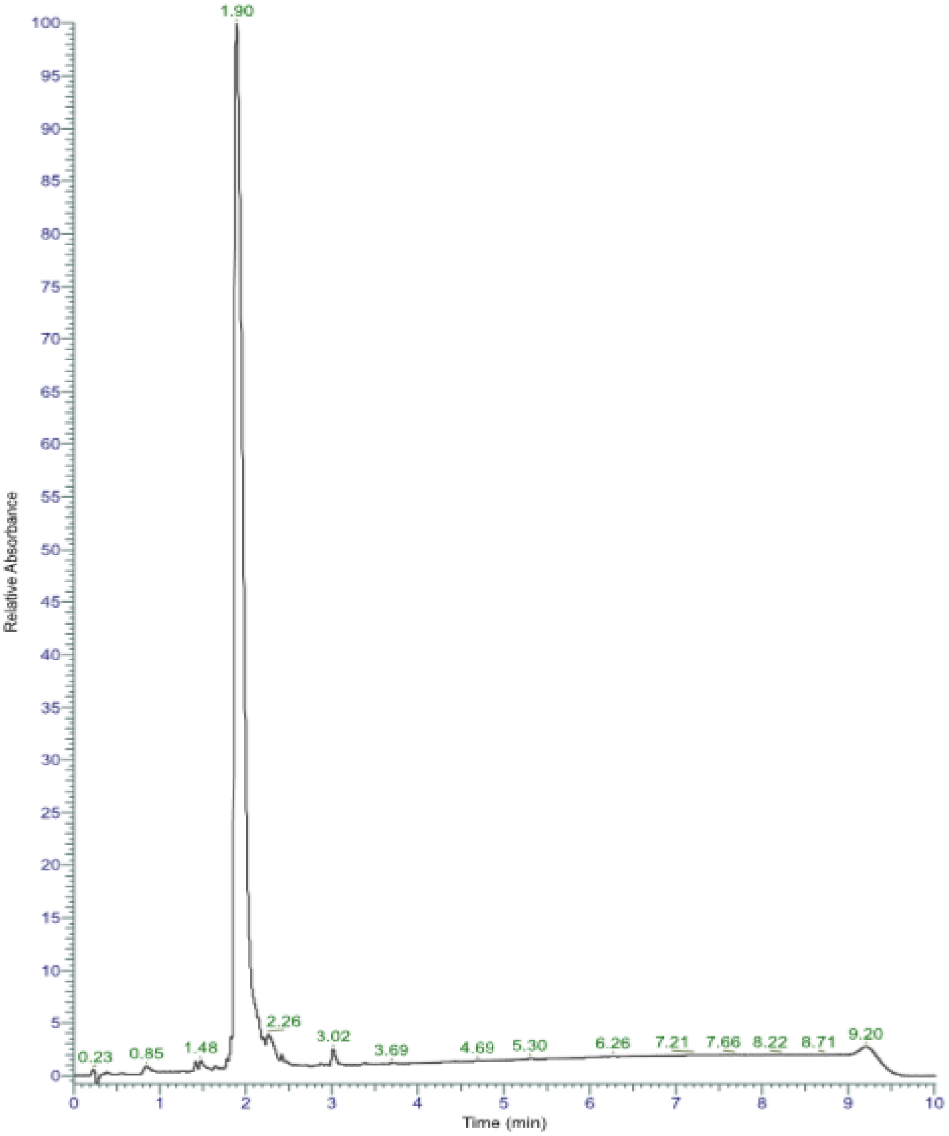


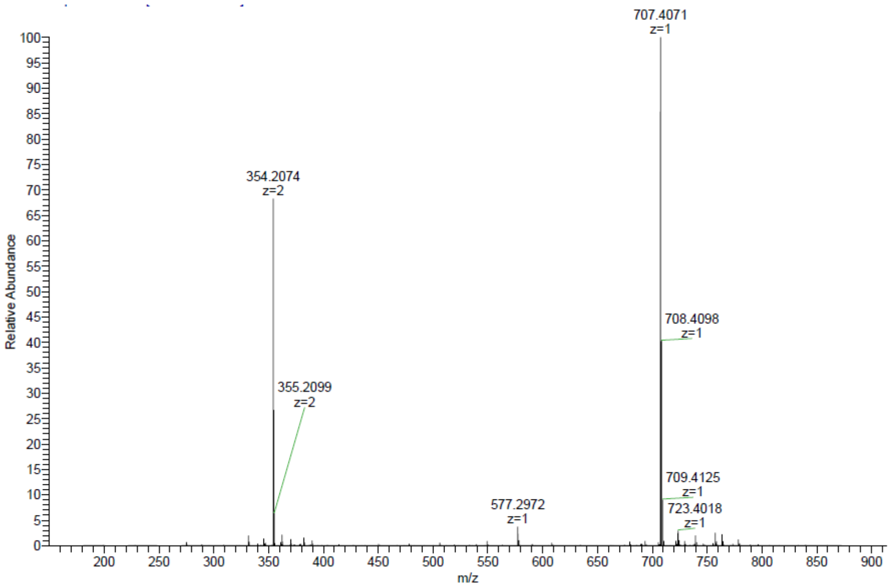

**VKEGYI** (20% v/v Dipropylamine, 60°C) was obtained as crude white solid after lyophilization (32.3 mg, 44.3%). Analytical RP-HPLC: $t_R$ = 1.98 min (A/D 100:0 to 0:100 in 7.00 min, λ = 214nm). HRMS (ESI+): $C_{33}H_{54}N_8O_9$ calc./obs. 707.40/707.41 Da $[M+H]^+$.

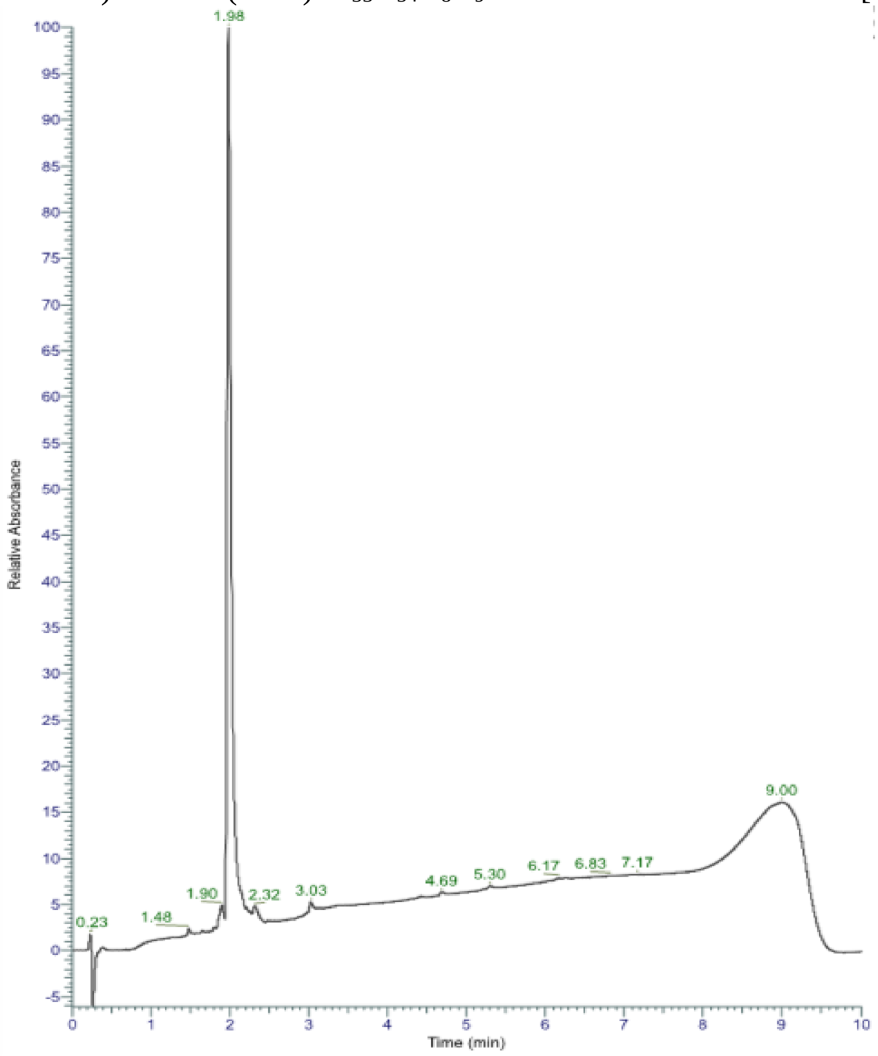


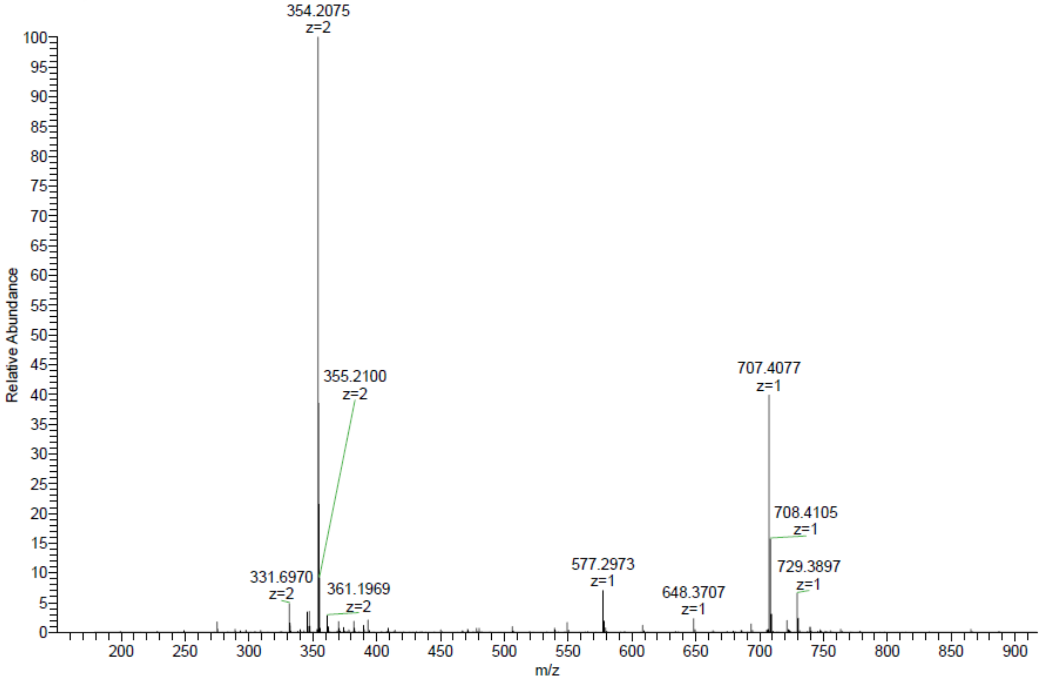

**VKEGYI** (20% v/v Dipropylamine + 0.5 M Oxyma, 60°C) was obtained as crude white solid after lyophilization (36.5 mg, 50.0%). Analytical RP-HPLC: $t_R$ = 1.93 min (A/D 100:0 to 0:100 in 7.00 min, $\lambda$ = 214nm). HRMS (ESI+): $C_{33}H_{54}N_8O_9$ calc./obs. 707.40/707.41 Da $[M+H]^+$.

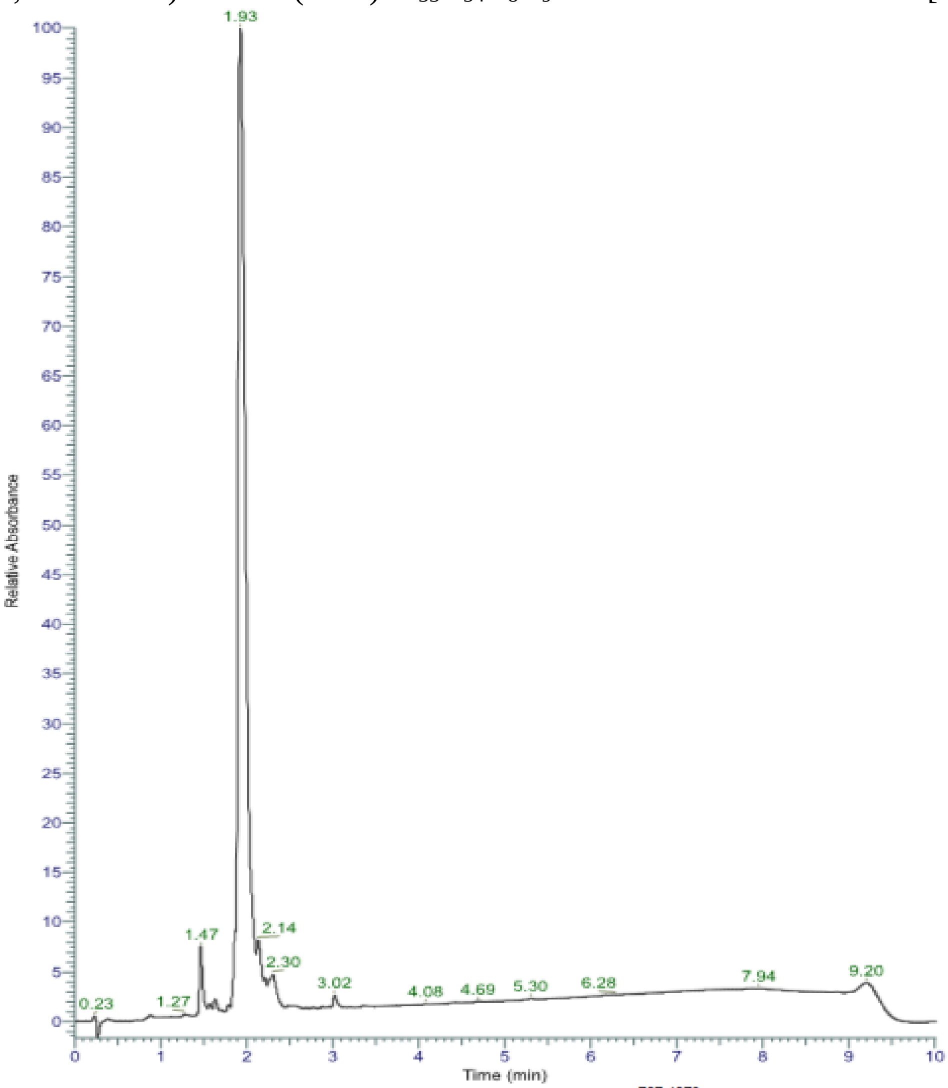


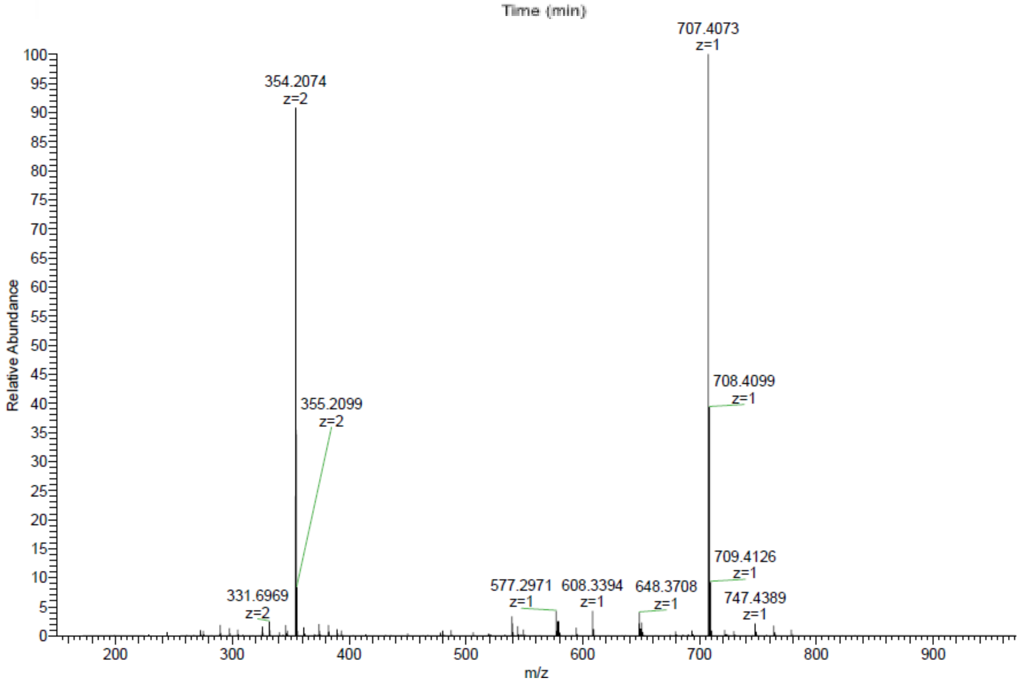

## 2.9 **Afamelanotide** (Ac-SYSNleEHfRWGKPV-$NH_2$)

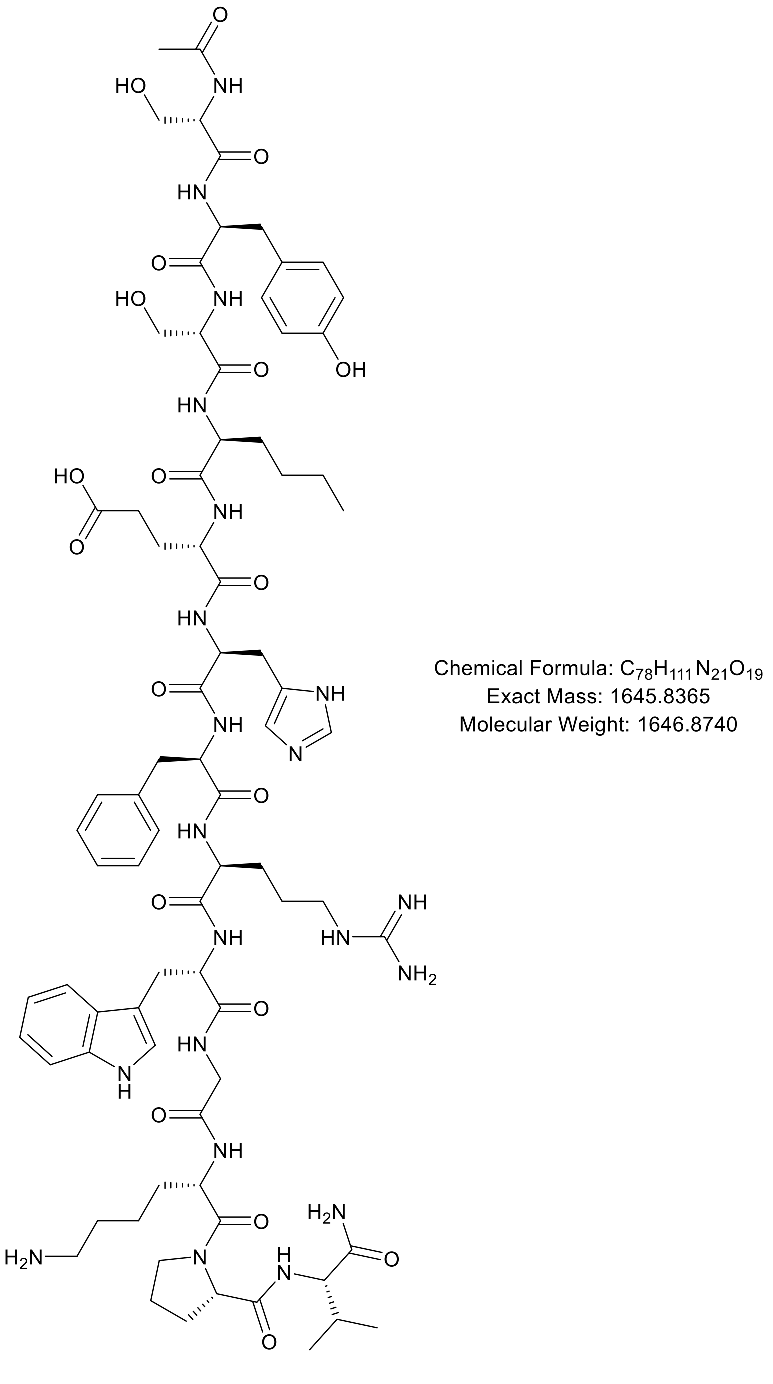


**Afamelanotide** (20% v/v Piperidine, 60°C) was obtained as foamy white solid after preparative RP-HPLC (21.6 mg, 16.8%). Analytical RP-HPLC: $t_R$ = 1.43 min (A/D 100:0 to 0:100 in 3.50 min, $\lambda$ = 214nm). HRMS (ESI+): $C_{78}H_{111}N_{21}O_{19}$ calc./obs. 1646.84/1646.84 Da $[M+H]^+$ .

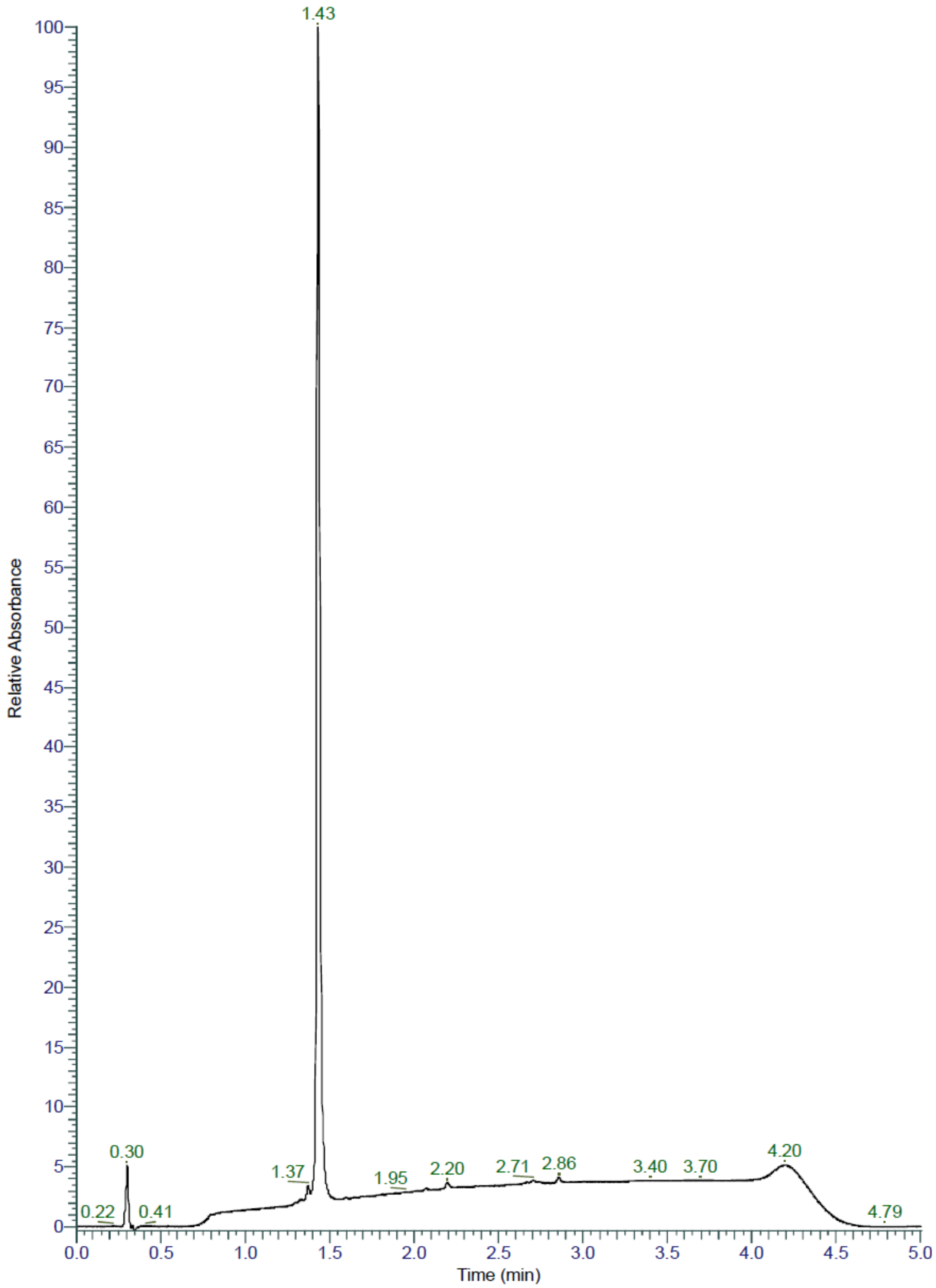
1.43
0.30
0.22
0.41
1.37
1.95
2.20
2.71
2.86
3.40
3.70
4.20
4.79
Relative Absorbance
Time (min)
0
5
10
15
20
25
30
35
40
45
50
55
60
65
70
75
80
85
90
95
100
0.0
0.5
1.0
1.5
2.0
2.5
3.0
3.5
4.0
4.5
5.0

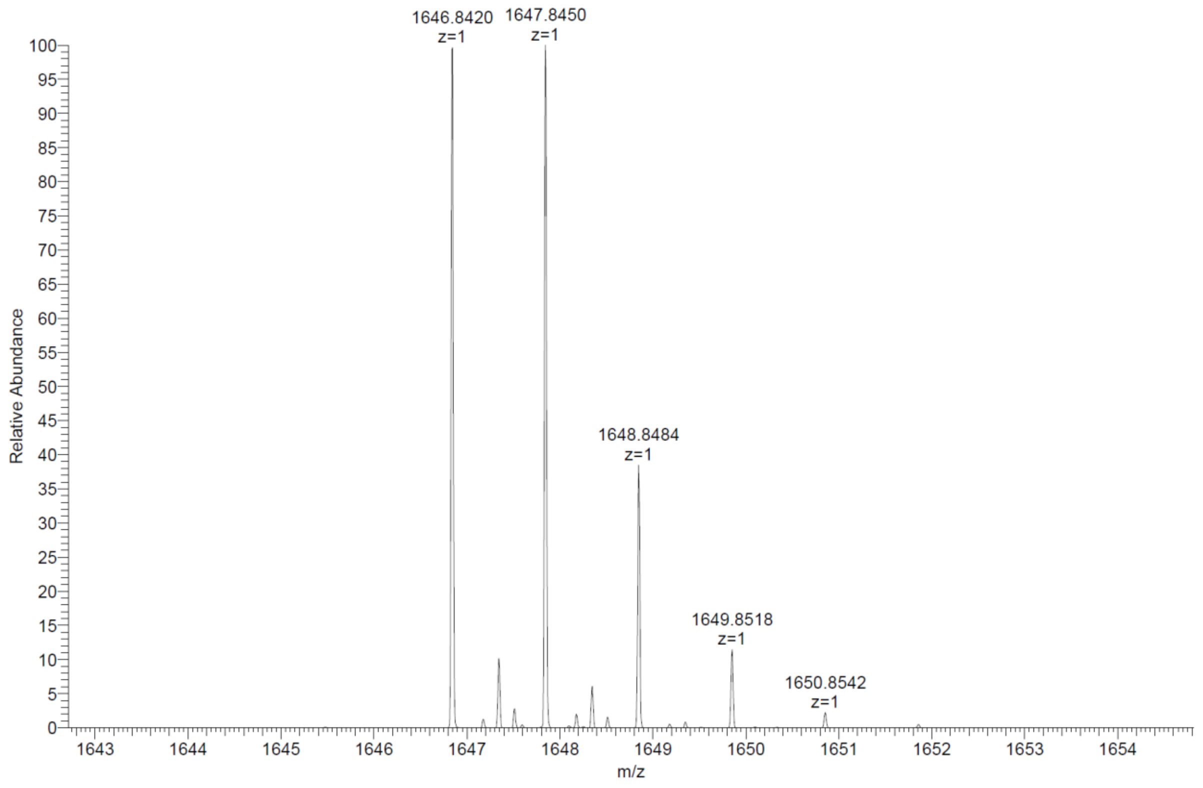
1646.8420
z=1
1647.8450
z=1
1648.8484
z=1
1649.8518
z=1
1650.8542
z=1
Relative Abundance
m/z

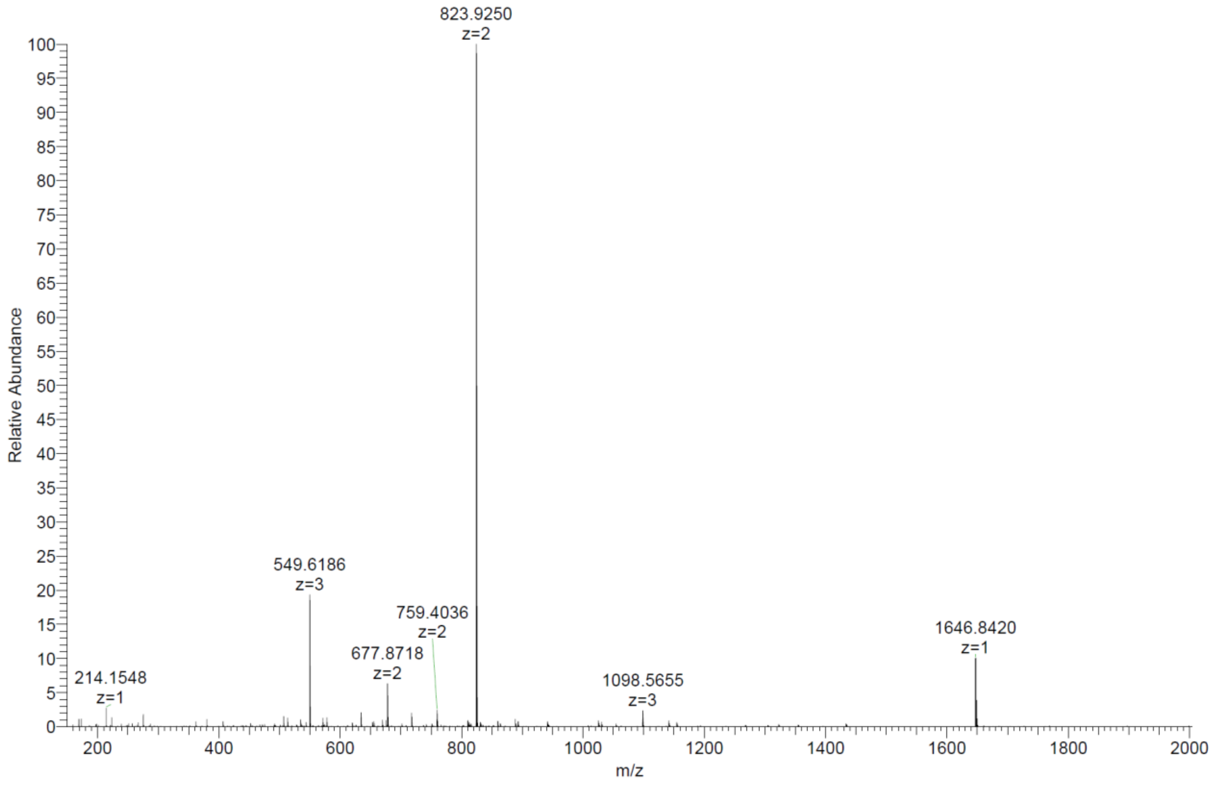
823.9250
z=2
549.6186
z=3
759.4036
z=2
677.8718
z=2
214.1548
z=1
1098.5655
z=3
1646.8420
z=1
Relative Abundance
m/z

**Afamelanotide** (25% v/v Dipropylamine, 60°C) was obtained as foamy white solid after preparative RP-HPLC (12.7 mg, 9.9%). Analytical RP-HPLC: $t_R$ = 1.44 min (A/D 100:0 to 0:100 in 3.50 min, λ = 214nm). HRMS (ESI+): $C_{78}H_{111}N_{21}O_{19}$ calc./obs. 1646.84/1646.84 Da $[M+H]^+$ .

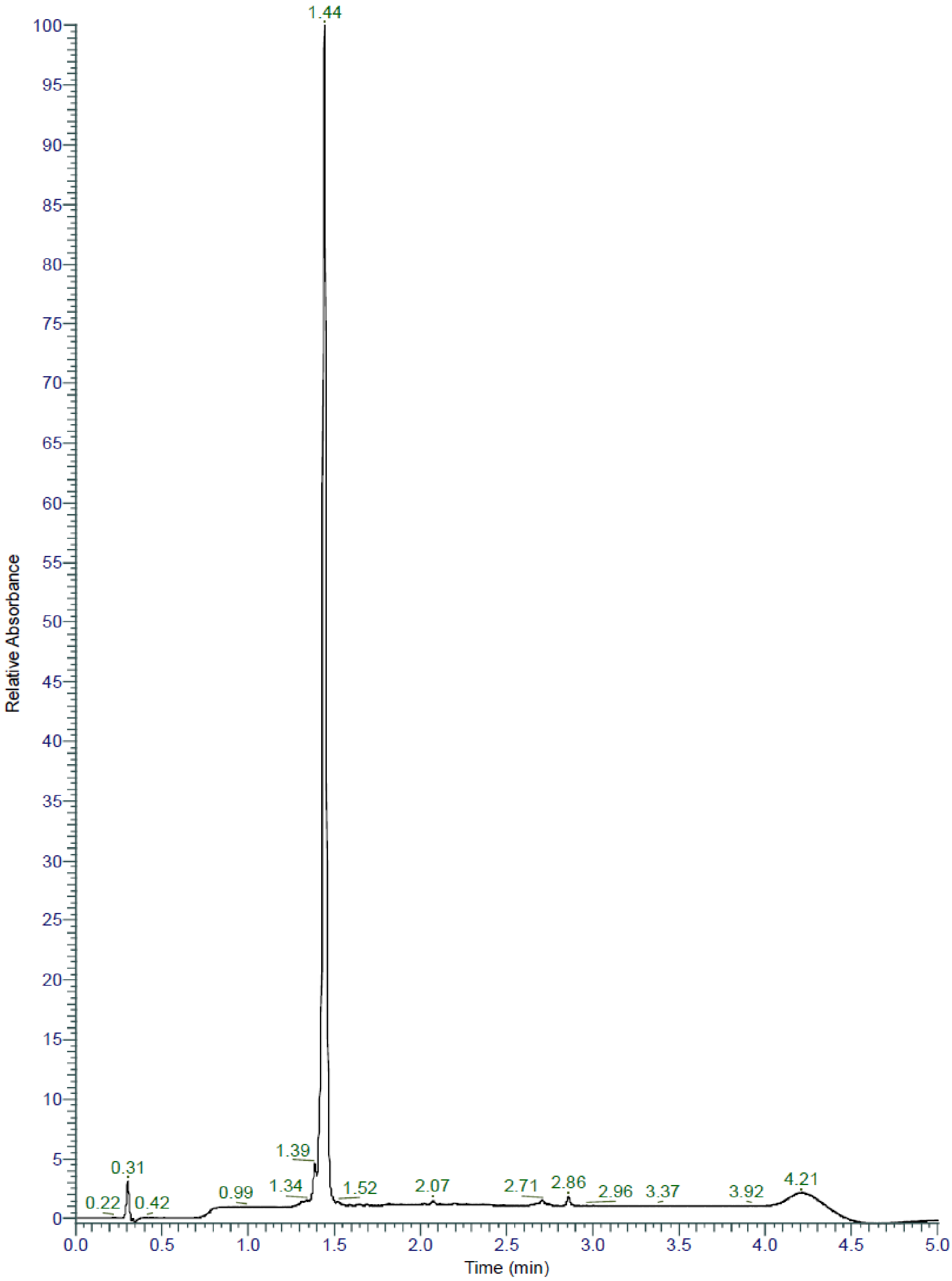

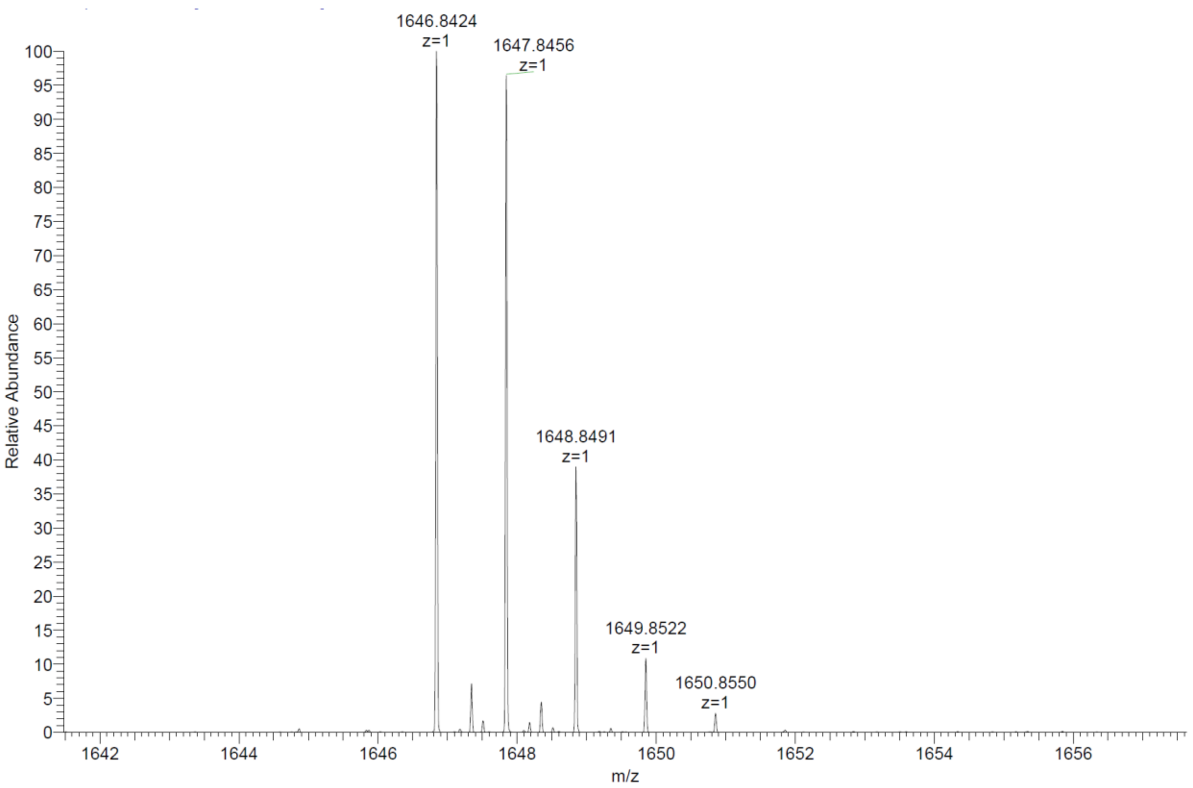
1646.8424
z=1
1647.8456
z=1
1648.8491
z=1
1649.8522
z=1
1650.8550
z=1
Relative Abundance
m/z

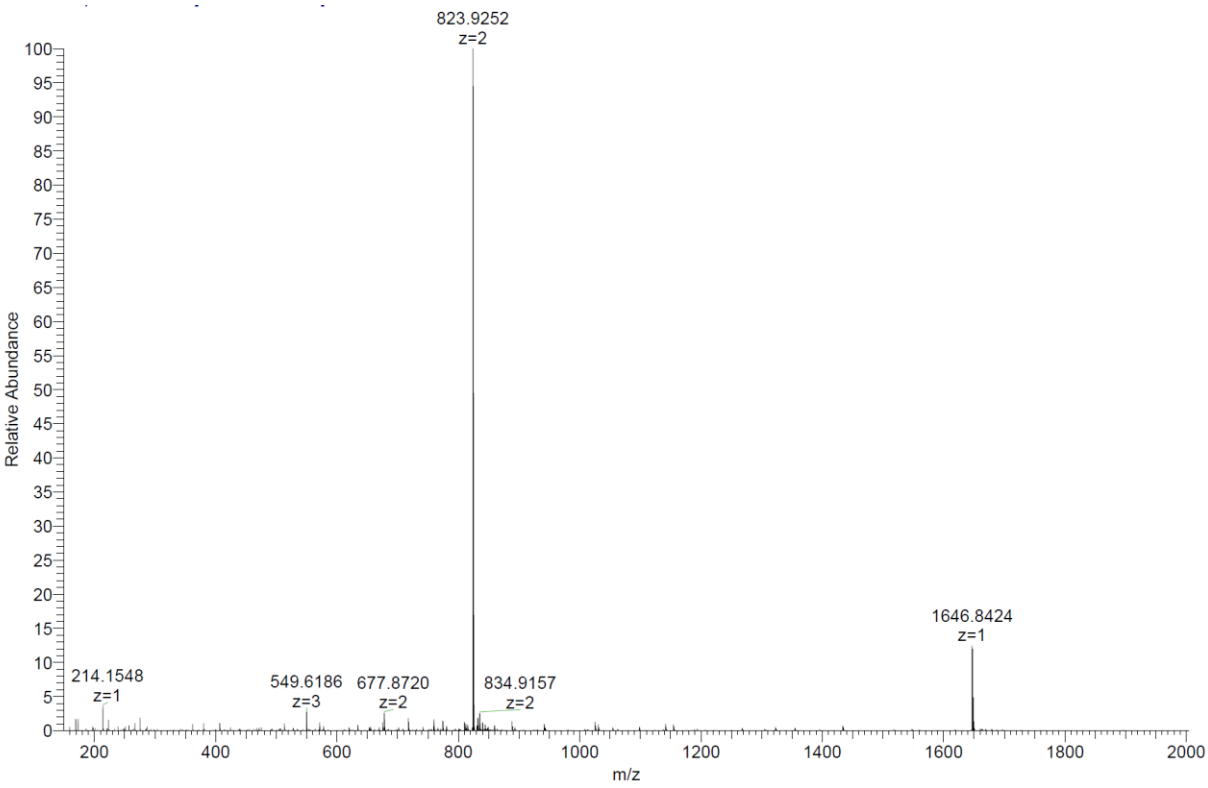
823.9252
z=2
1646.8424
z=1
214.1548
z=1
549.6186
z=3
677.8720
z=2
834.9157
z=2
Relative Abundance
m/z

## 2.10 **Bivaluridin** (fPRPGGGGNGDFEEIPEEYL-OH)

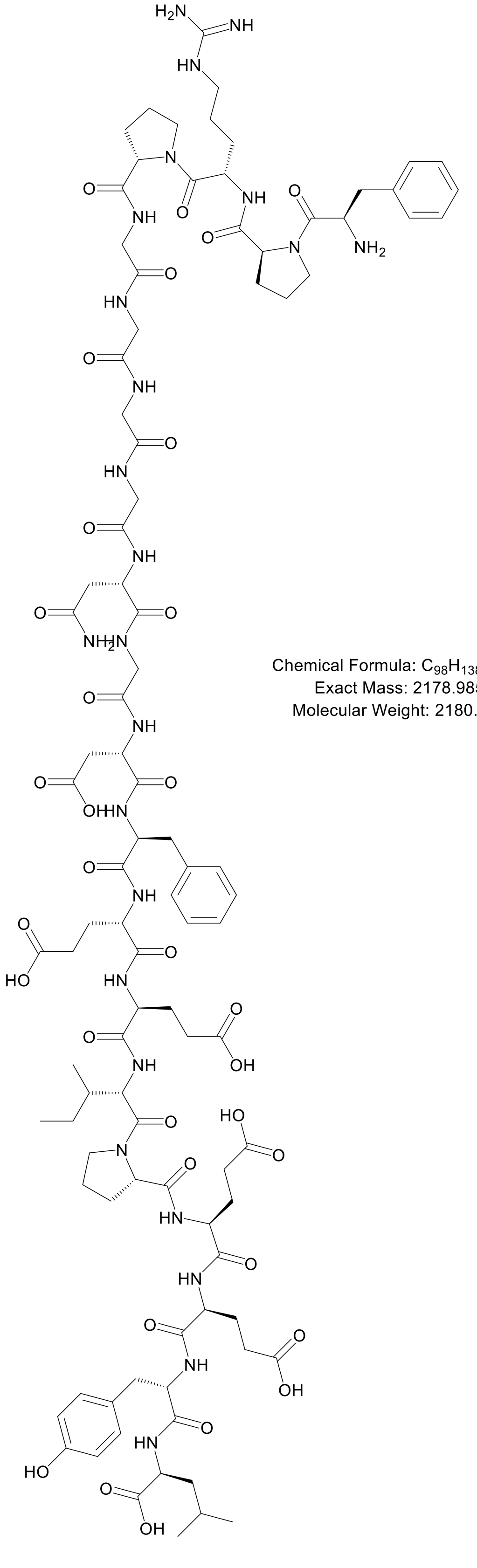

Chemical Formula: $C_{98}H_{138}N_{24}O_{33}$
Exact Mass: 2178.9858
Molecular Weight: 2180.3170

**Bivaluridin** (20% v/v Piperidine, 60°C) was obtained as foamy white solid after preparative RP-HPLC (111.6 mg, 46.3%). Analytical RP-HPLC: $t_R$ = 1.52 min (A/D 100:0 to 0:100 in 3.50 min, $\lambda$ = 214nm). HRMS (ESI+): $C_{98}H_{138}N_{24}O_{33}$ calc./obs. 2179.99/2179.99 Da $[M+H]^+$.

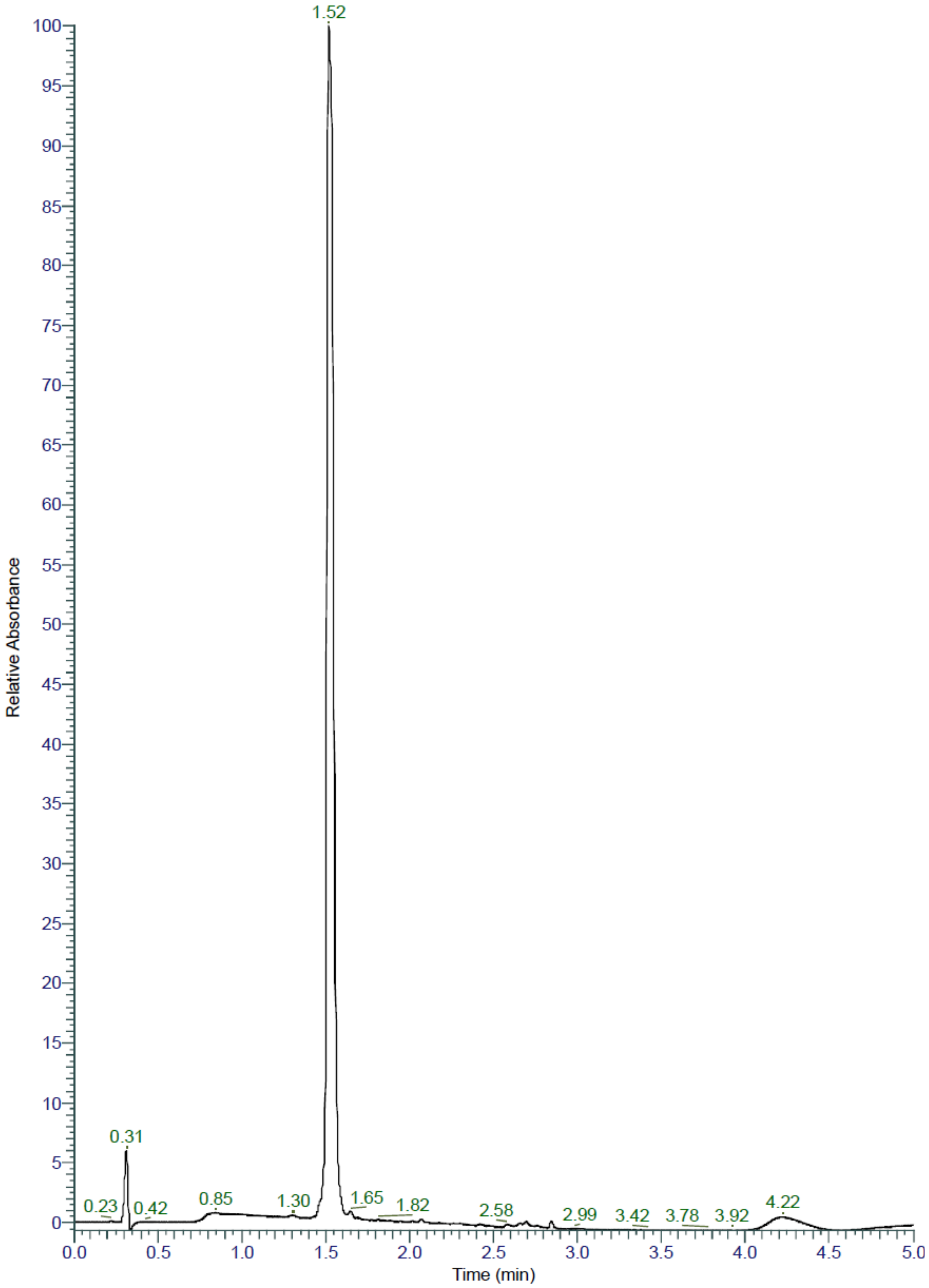

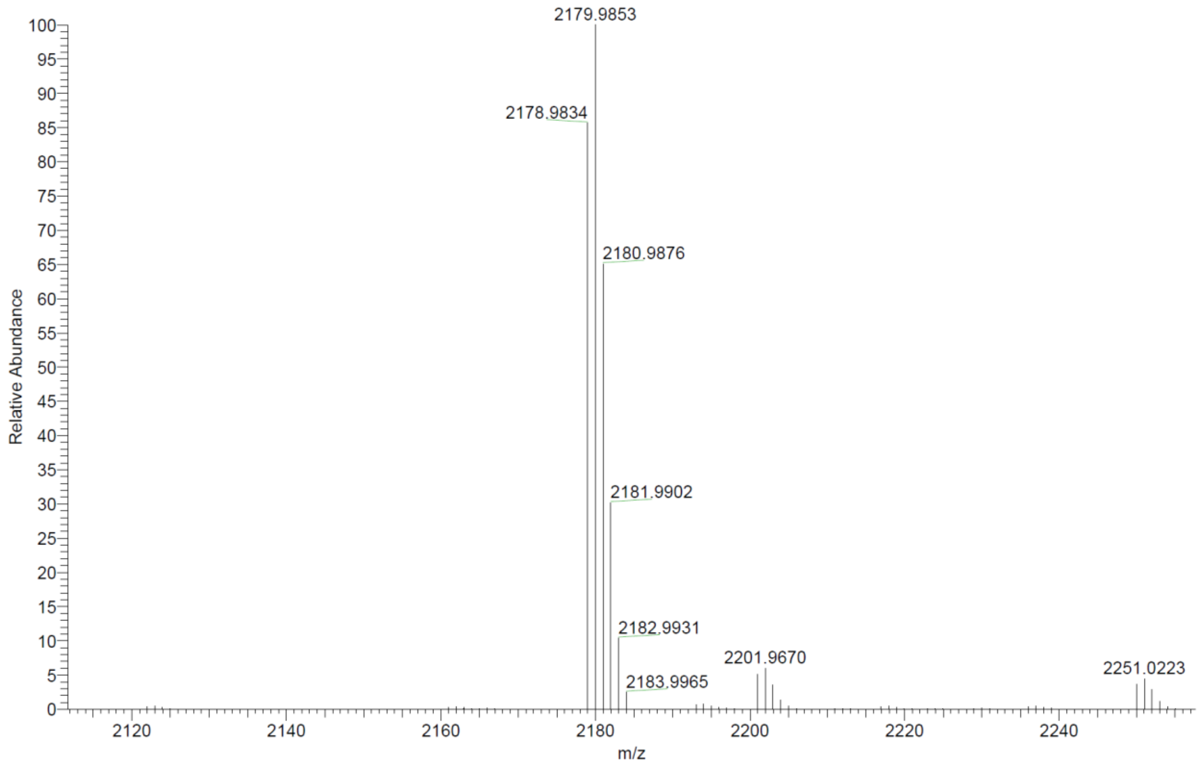
2179.9853
2178.9834
2180.9876
2181.9902
2182.9931
2183.9965
2201.9670
2251.0223
Relative Abundance
m/z

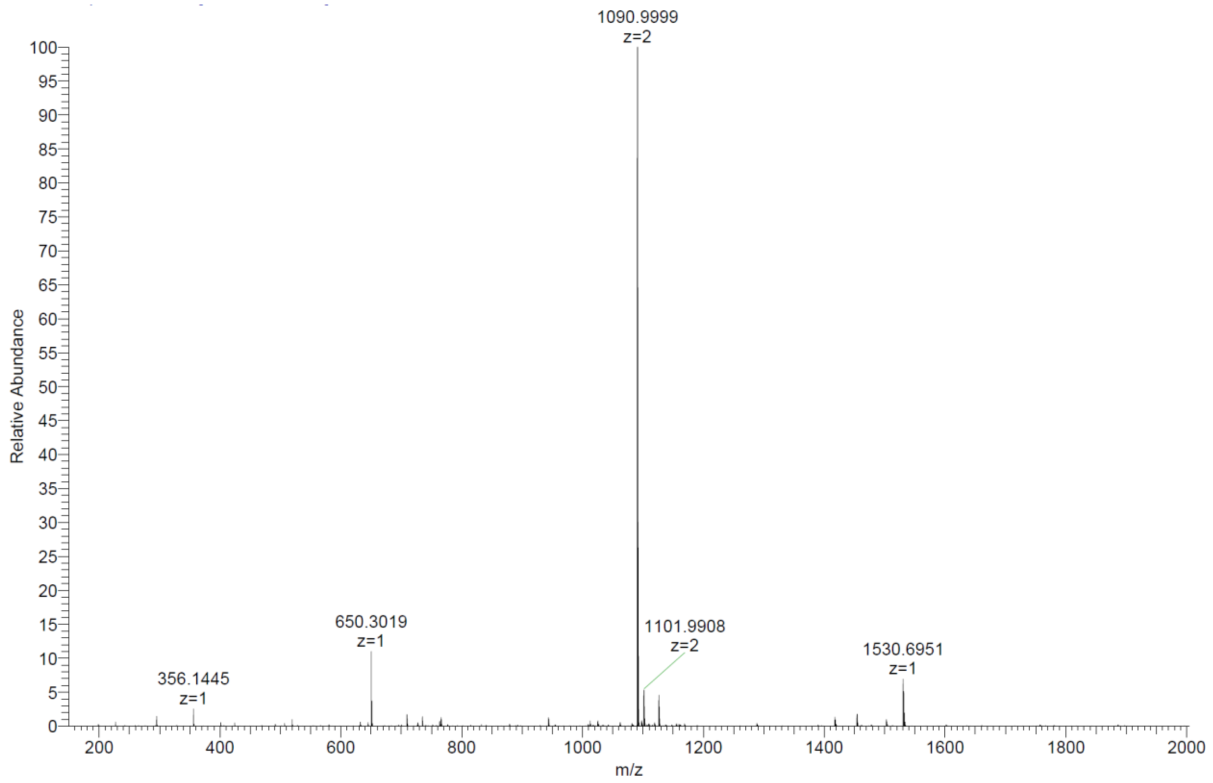
1090.9999
z=2
650.3019
z=1
356.1445
z=1
1101.9908
z=2
1530.6951
z=1
Relative Abundance
m/z

**Bivaluridin** (25% v/v Dipropylamine, 60°C) was obtained as foamy white solid after preparative RP-HPLC (93.3 mg, 38.7%). Analytical RP-HPLC: $t_R$ = 1.52 min (A/D 100:0 to 0:100 in 3.50 min, λ = 214nm). HRMS (ESI+): $C_{98}H_{138}N_{24}O_{33}$ calc./obs. 2179.99/2179.99 Da $[M+H]^+$.

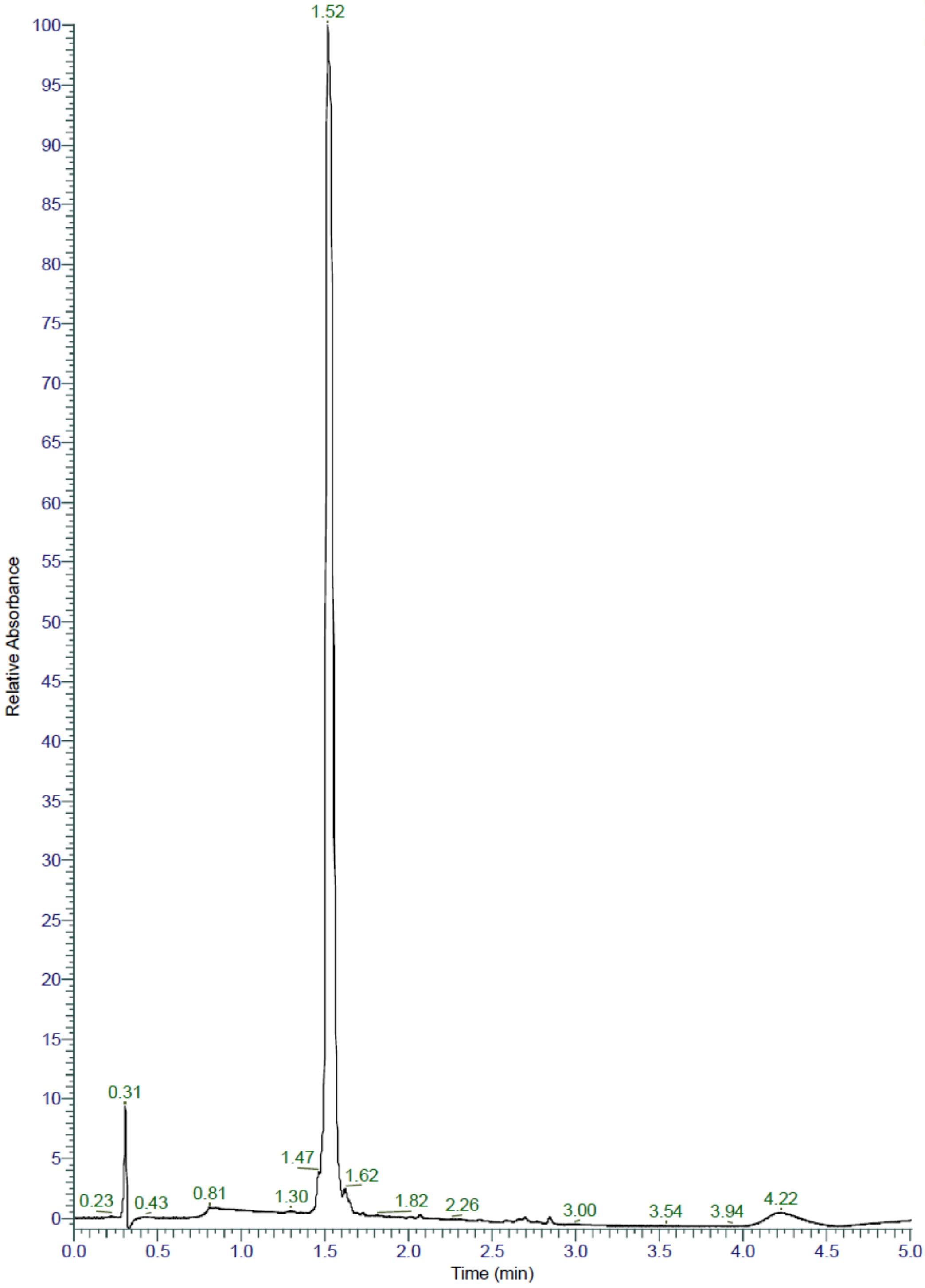

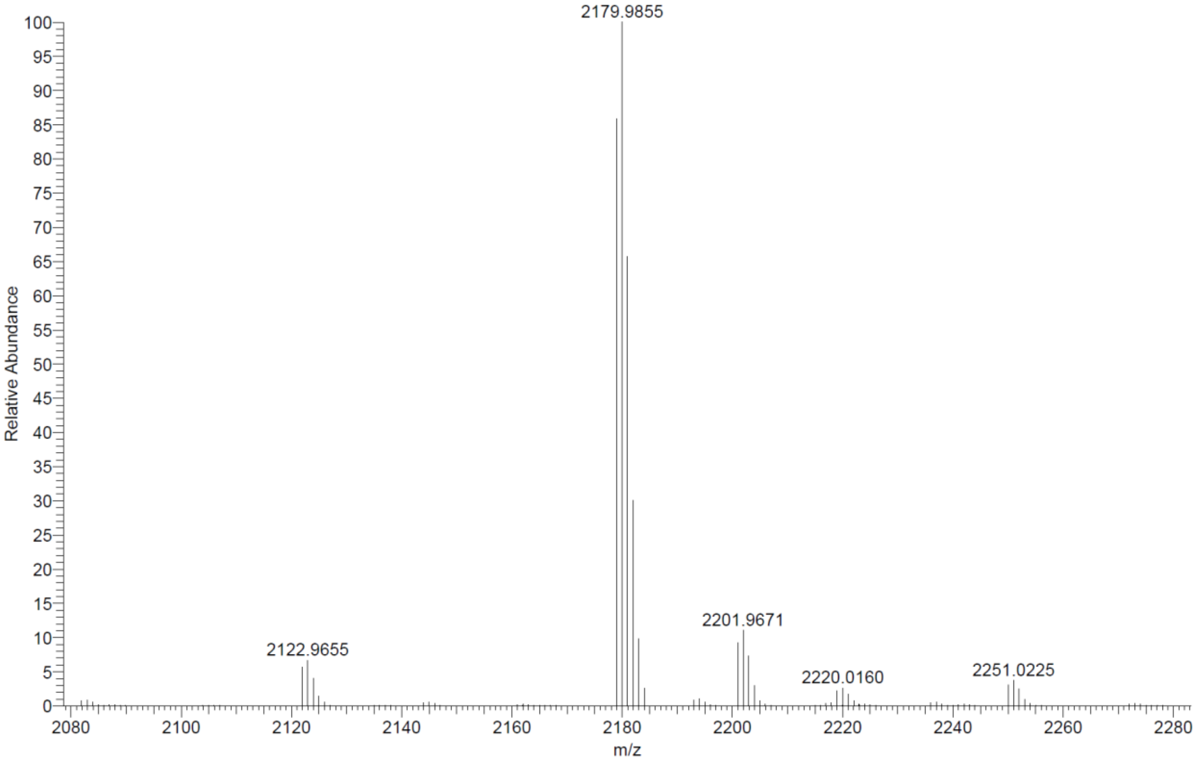
2179.9855
2122.9655
2201.9671
2220.0160
2251.0225
Relative Abundance
m/z

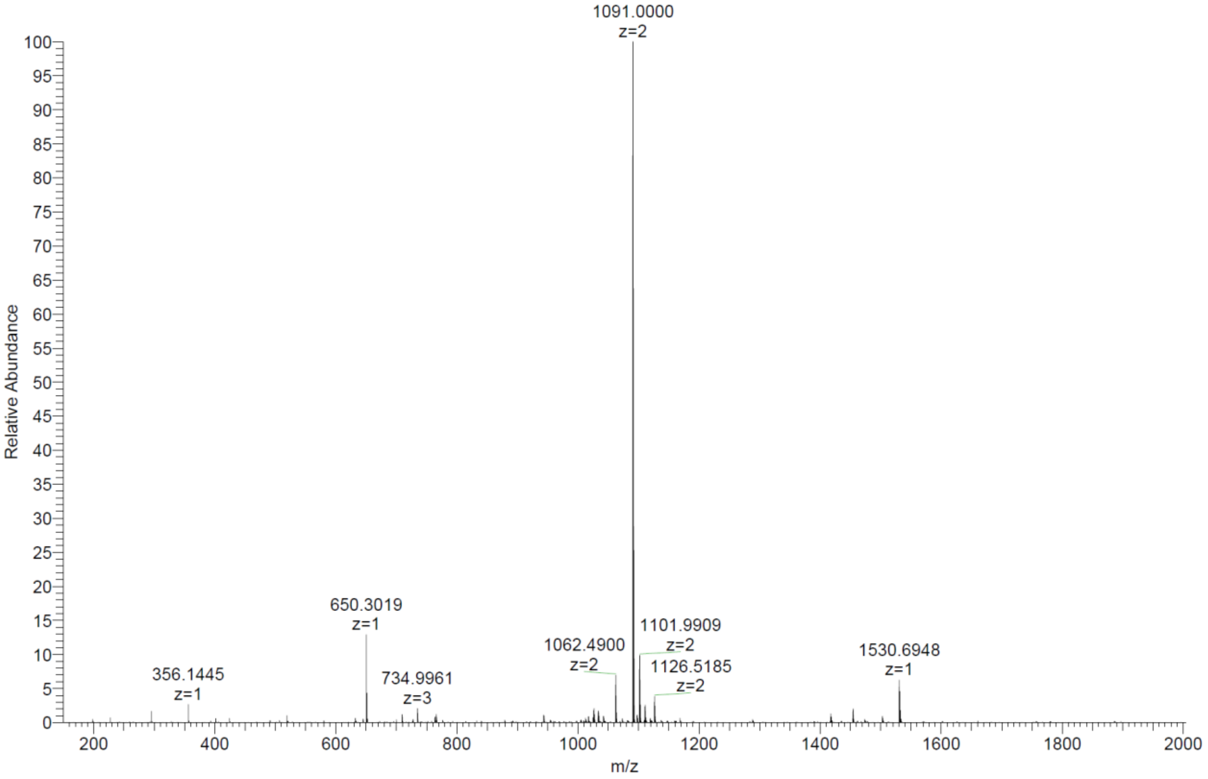
1091.0000
z=2
650.3019
z=1
356.1445
z=1
734.9961
z=3
1062.4900
z=2
1101.9909
z=2
1126.5185
z=2
1530.6948
z=1
Relative Abundance
m/z

**Bivaluridin** (20% v/v Piperidine, 90°C) was obtained as foamy white solid after lyophilization (57.2 mg, 6.6%). Analytical RP-HPLC: $t_R$ = 3.17 min (A/D 100:0 to 0:100 in 7.00 min, λ = 214nm). HRMS (ESI+): $C_{98}H_{138}N_{24}O_{33}$ calc./obs. 2179.99/2179.99 Da $[M+H]^+$.

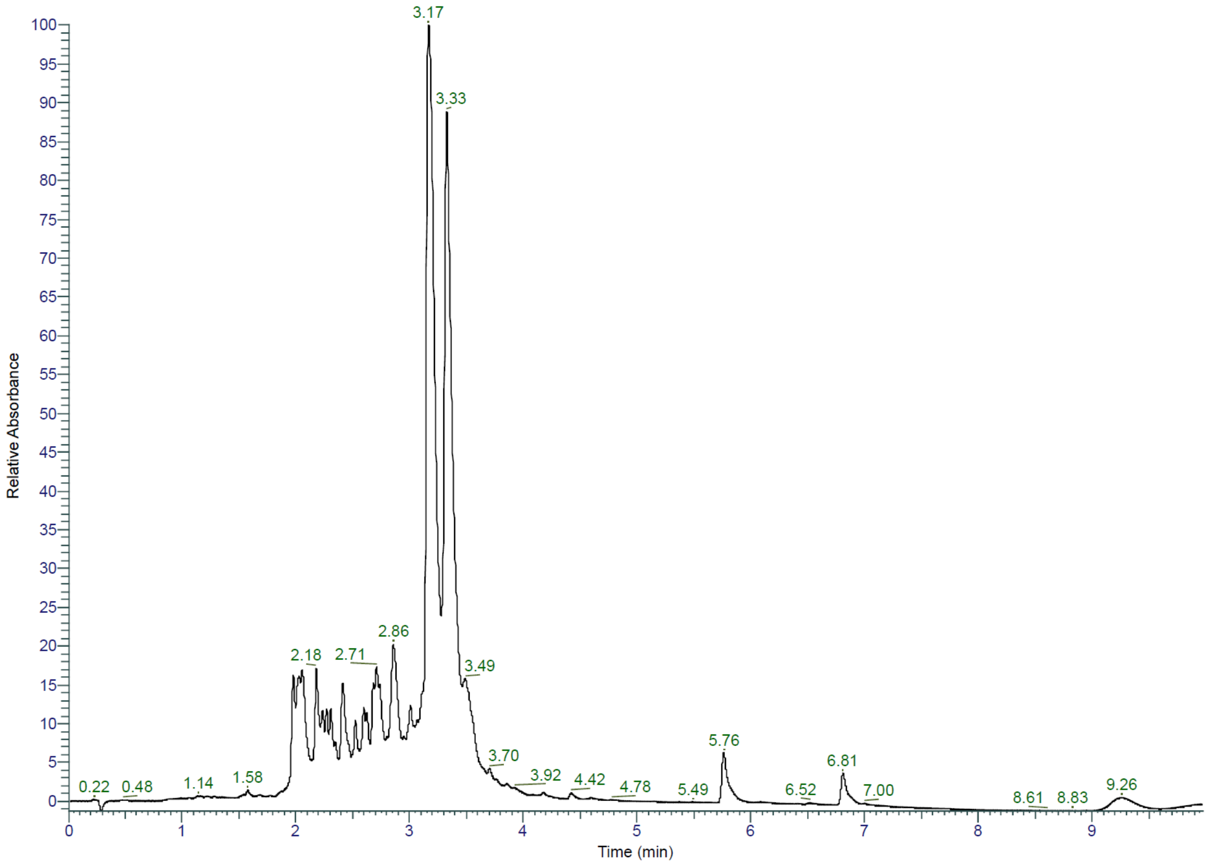

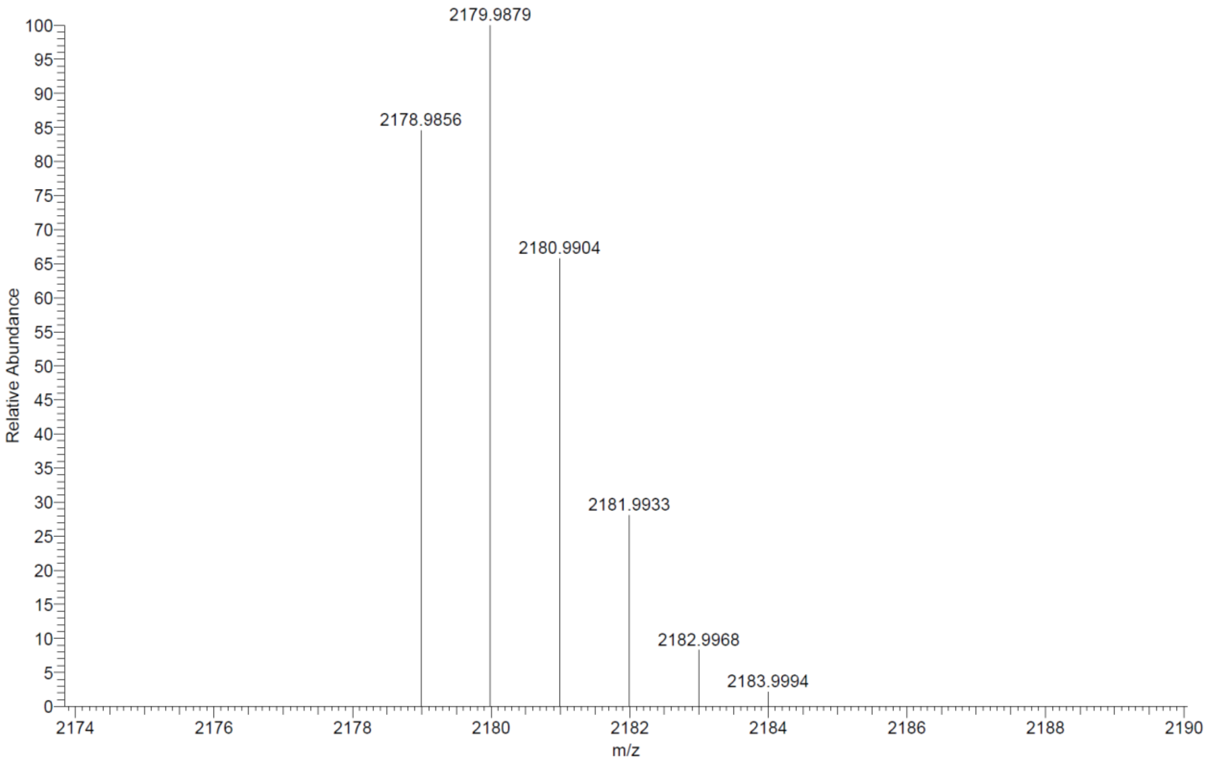
2179.9879
2178.9856
2180.9904
2181.9933
2182.9968
2183.9994
Relative Abundance
m/z

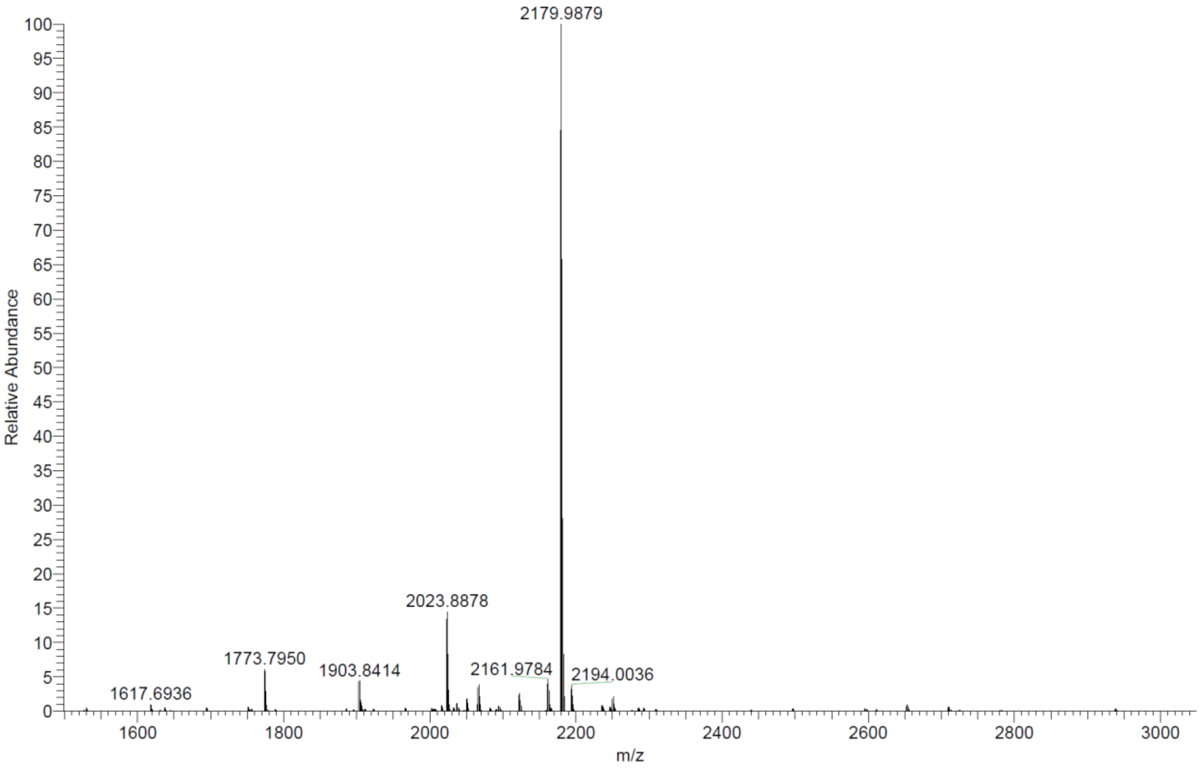
2179.9879
2023.8878
1773.7950
1903.8414
2161.9784
2194.0036
1617.6936
Relative Abundance
m/z

**Bivaluridin** (25% v/v Dipropylamine, 90°C) was obtained as foamy white solid after lyophilization (39.5 mg, 4.6%). Analytical RP-HPLC: $t_R$ = 3.19 min (A/D 100:0 to 0:100 in 7.00 min, λ = 214nm). HRMS (ESI+): $C_{98}H_{138}N_{24}O_{33}$ calc./obs. 2179.99/2179.99 Da $[M+H]^+$.

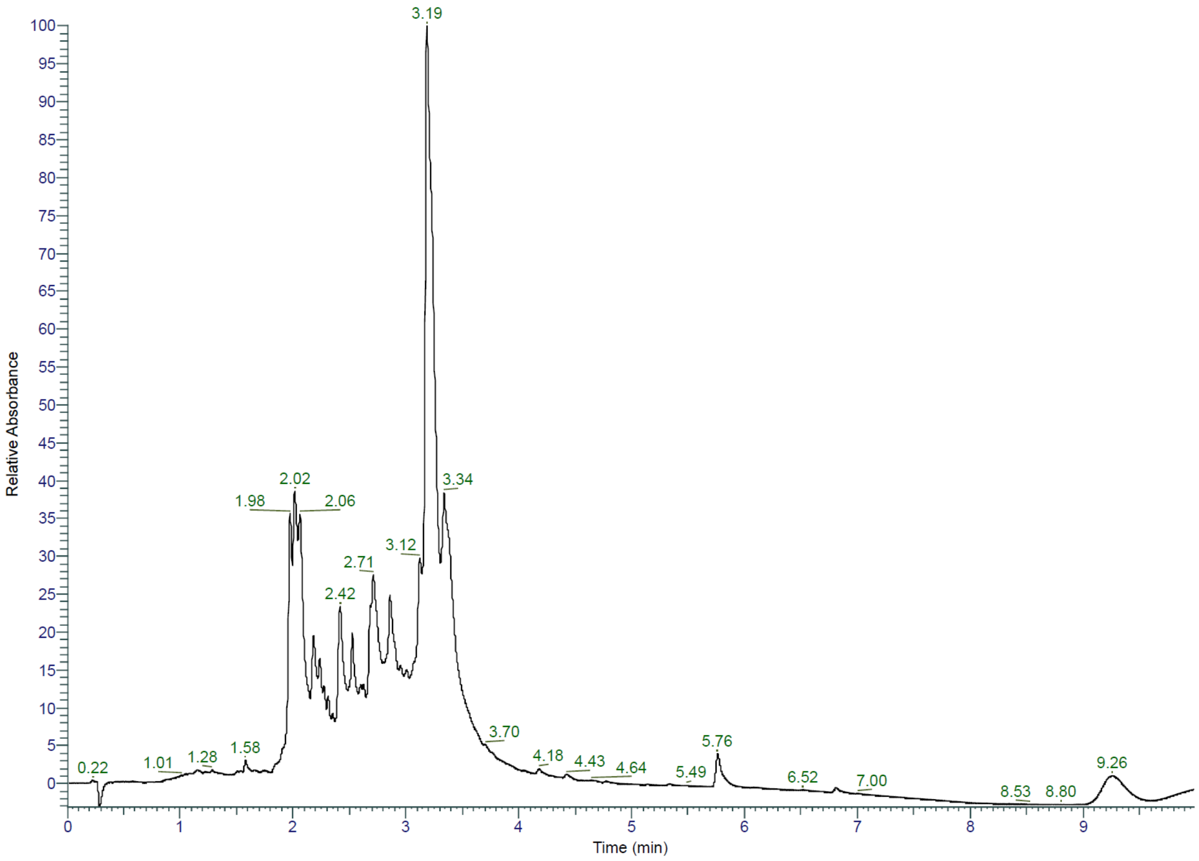

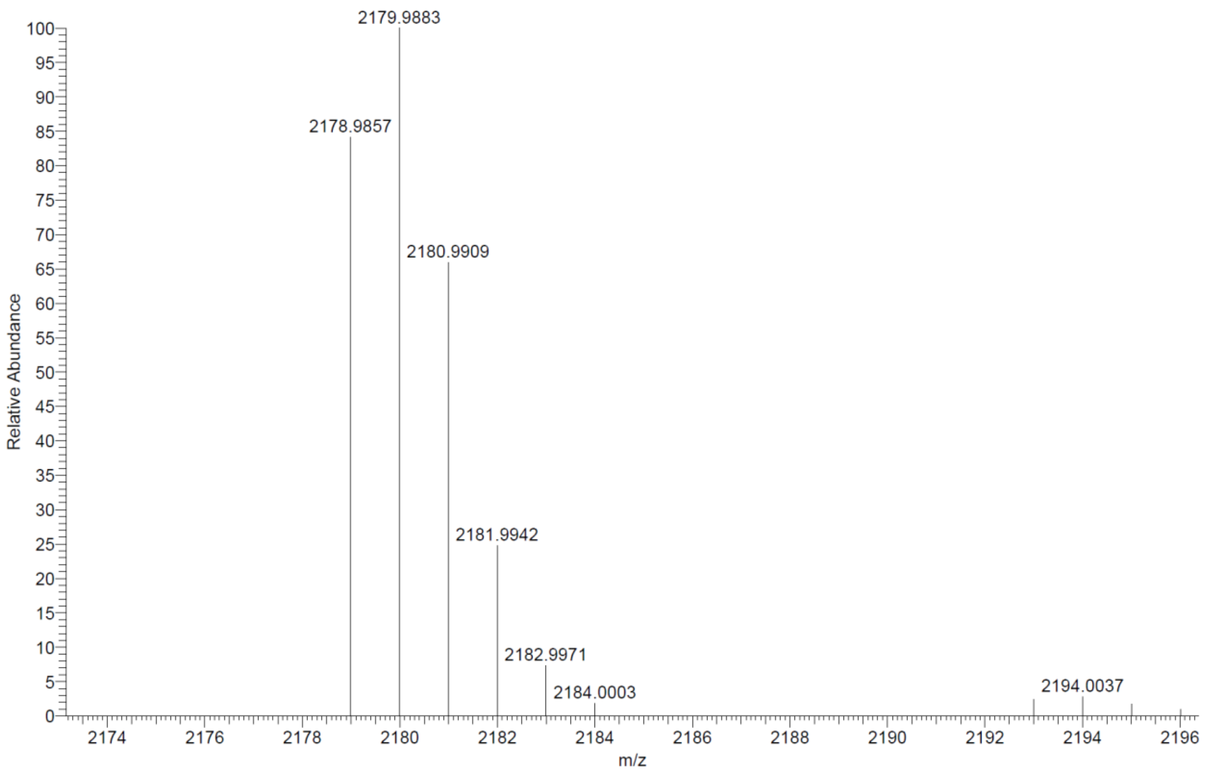
2179.9883
2178.9857
2180.9909
2181.9942
2182.9971
2184.0003
2194.0037
Relative Abundance
m/z

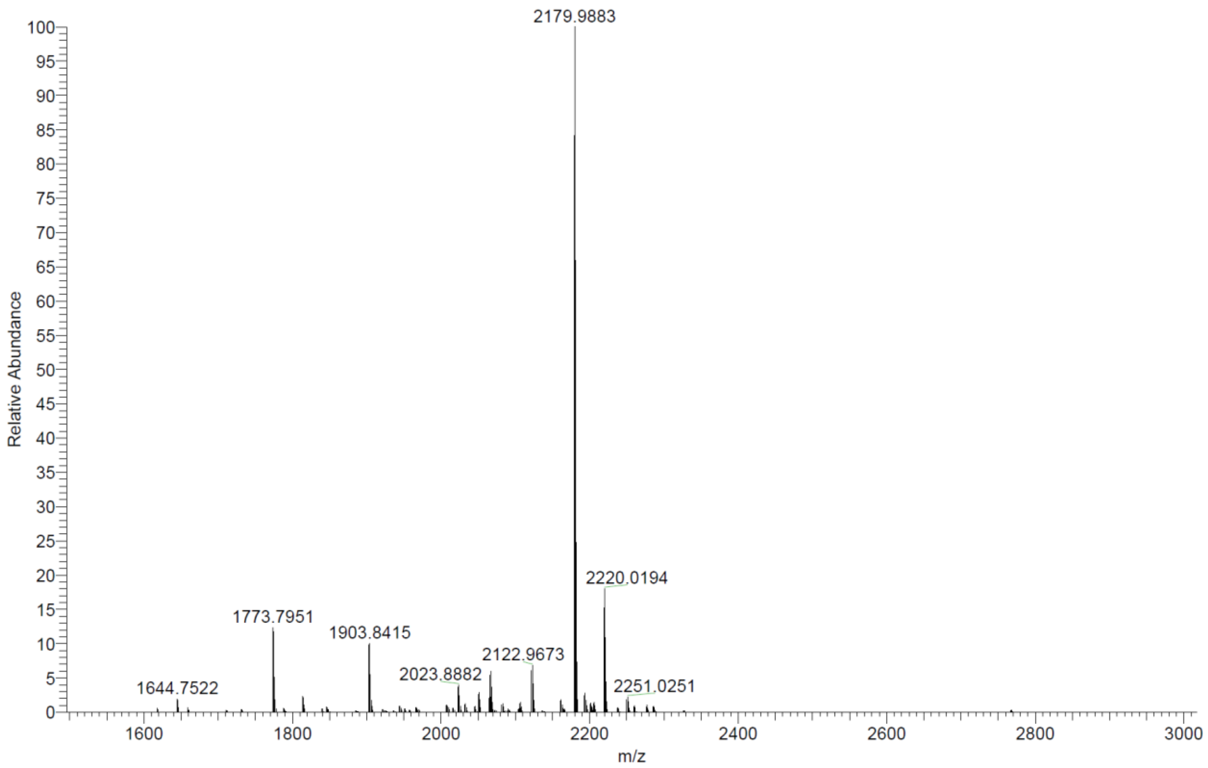
2179.9883
2220.0194
1773.7951
1903.8415
2122.9673
2023.8882
2251.0251
1644.7522
Relative Abundance
m/z

2.11 **G1KL** ((KL)$_2$*K*KL)

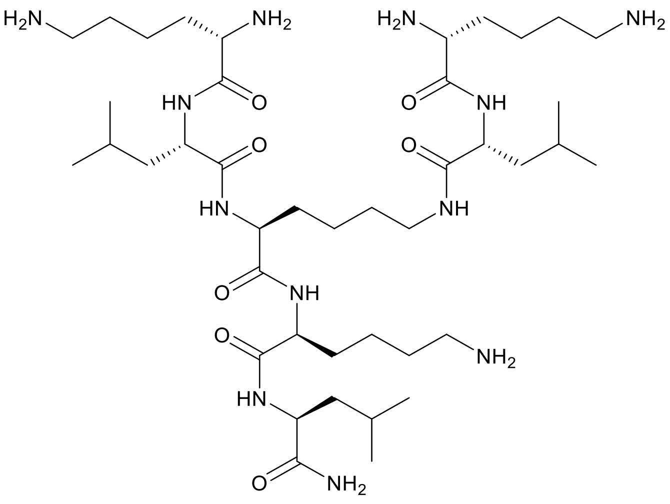


Chemical Formula: $C_{42}H_{84}N_{12}O_7$
Exact Mass: 868.6586
Molecular Weight: 869.2110

**G1KL** (20% v/v Piperidine, 60°C) was obtained as crude white solid after lyophilization (90.5 mg, 72.5%). Analytical RP-HPLC: $t_R$ = 2.11 min (A/D 100:0 to 0:100 in 7.00 min, λ = 214nm). HRMS (ESI+): $C_{42}H_{84}N_{12}O_7$ calc./obs. 869.66/869.66 Da $[M+H]^+$.

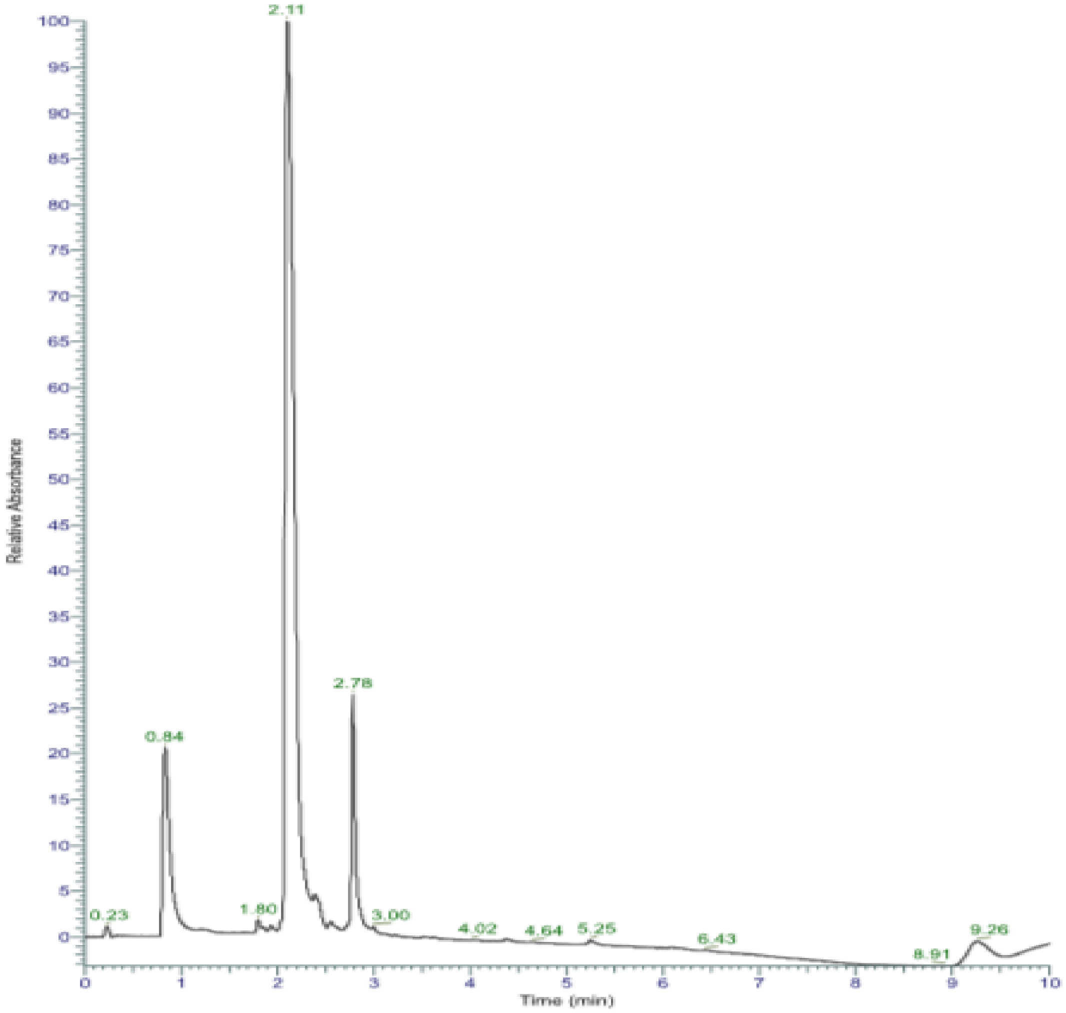


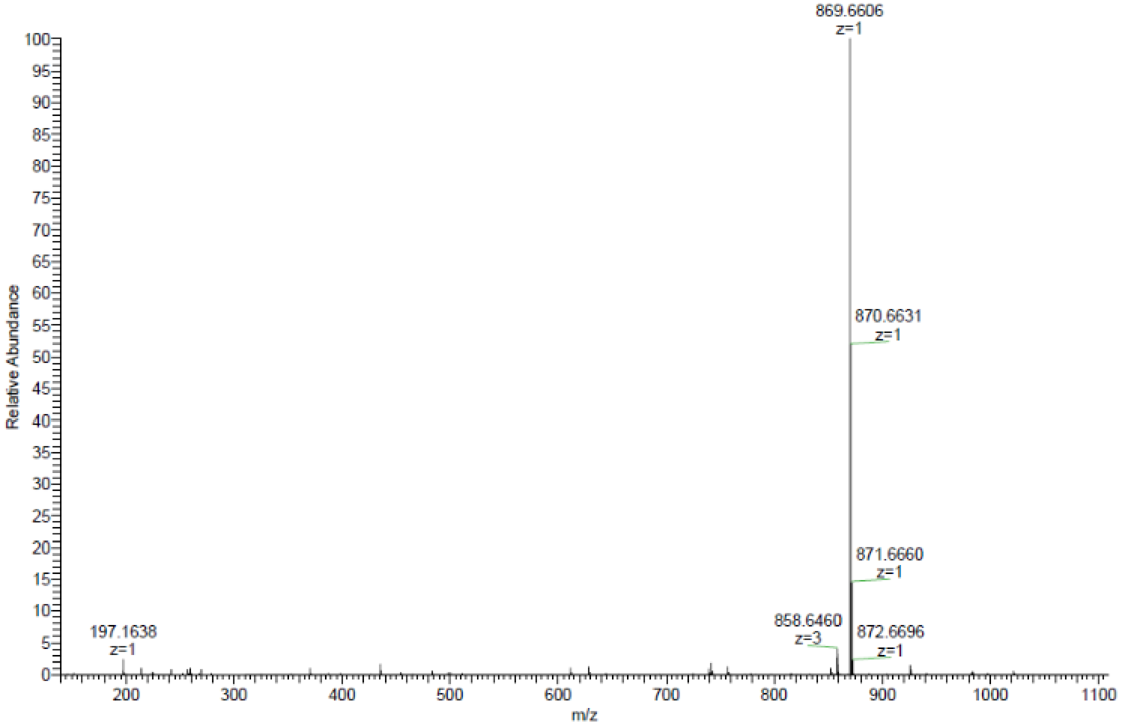

**G1KL** (5% w/v Piperazine + 2% v/v DBU, 60°C) was obtained as crude white solid after lyophilization (30.4 mg, 26.2%). Analytical RP-HPLC: $t_R$ = 1.85 min (A/D 100:0 to 0:100 in 7.00 min, λ = 214nm). HRMS (ESI+): $C_{42}H_{84}N_{12}O_7$ calc./obs. 869.66/869.66 Da $[M+H]^+$.

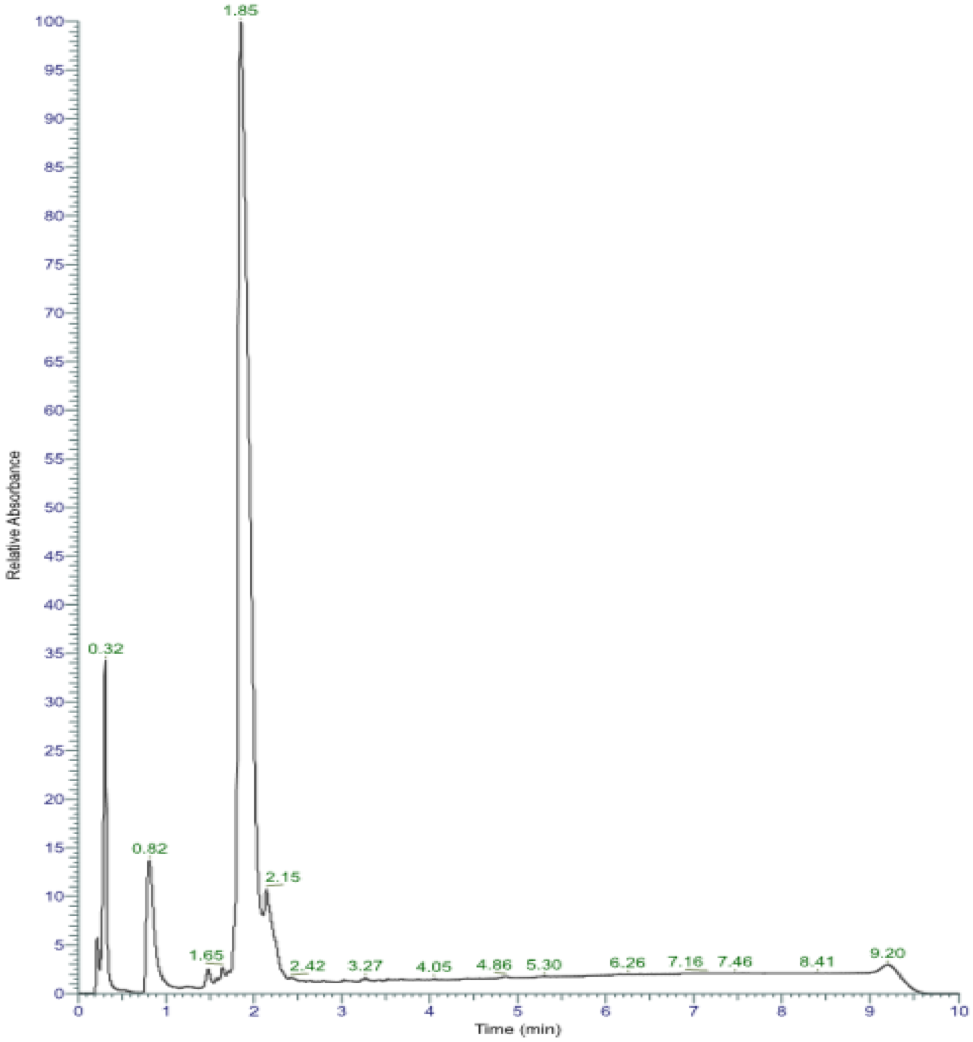


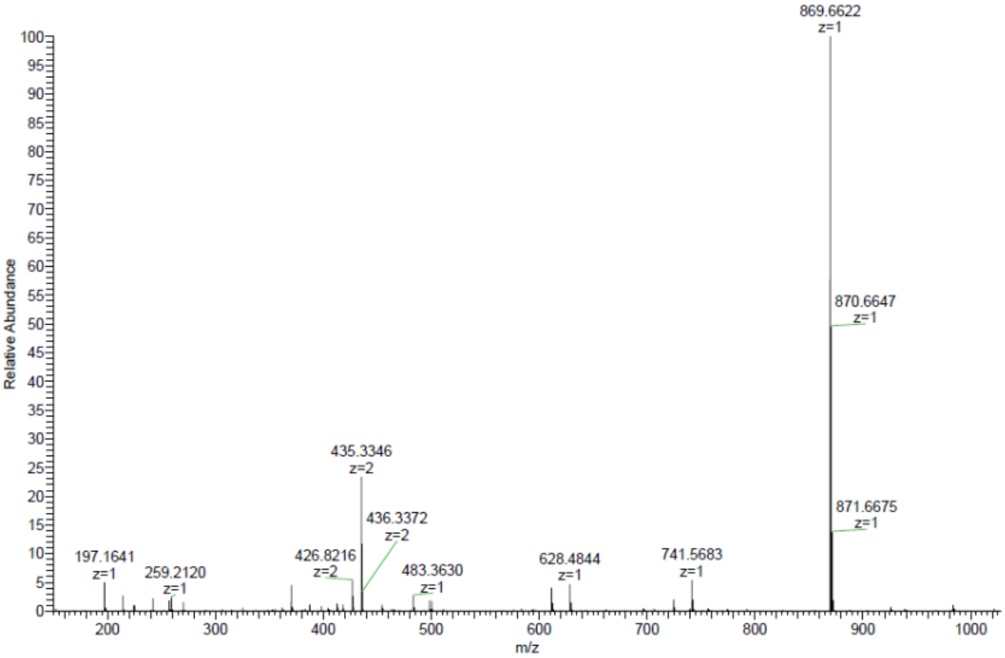

**G1KL** (20% v/v Diisopropylamine, 60°C) was obtained as crude white solid after lyophilization (0.3 mg, 0.0%). Analytical RP-HPLC: $t_R$ = - min (A/D 100:0 to 0:100 in 7.00 min, λ = 214nm). HRMS (ESI+): $C_{42}H_{84}N_{12}O_7$ calc./obs. 869.66/869.66 Da $[M+H]^+$.

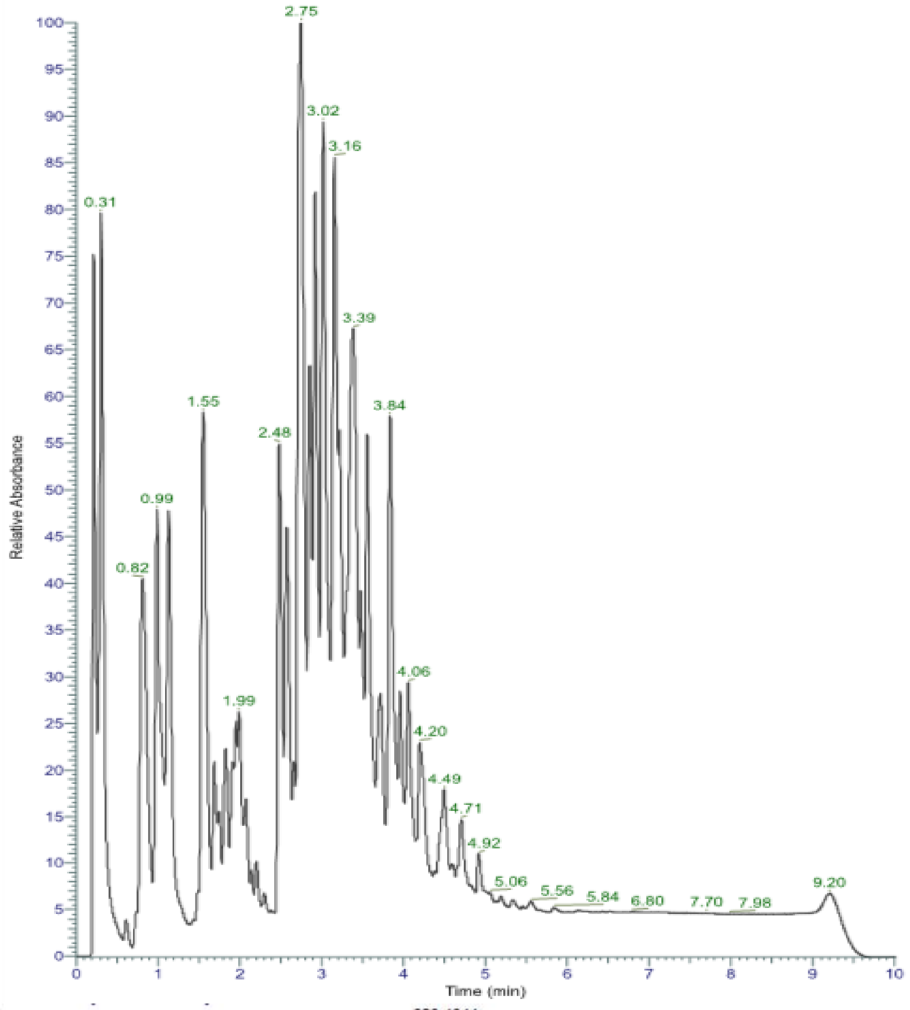


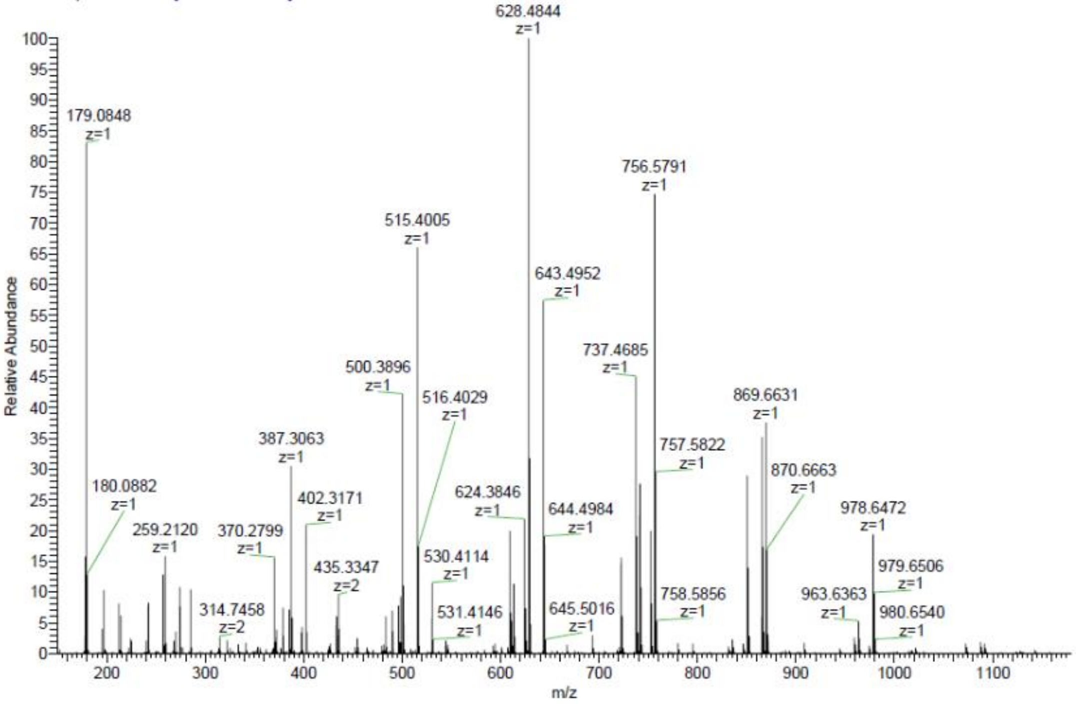

**G1KL** (20% v/v Diisopropylamine + 1% v/v DBU, 60°C) was obtained as crude white solid after lyophilization (38.8 mg, 35.6%). Analytical RP-HPLC: $t_R$ = 1.81 min (A/D 100:0 to 0:100 in 7.00 min, λ = 214nm). HRMS (ESI+): $C_{42}H_{84}N_{12}O_7$ calc./obs. 869.66/869.66 Da $[M+H]^+$.

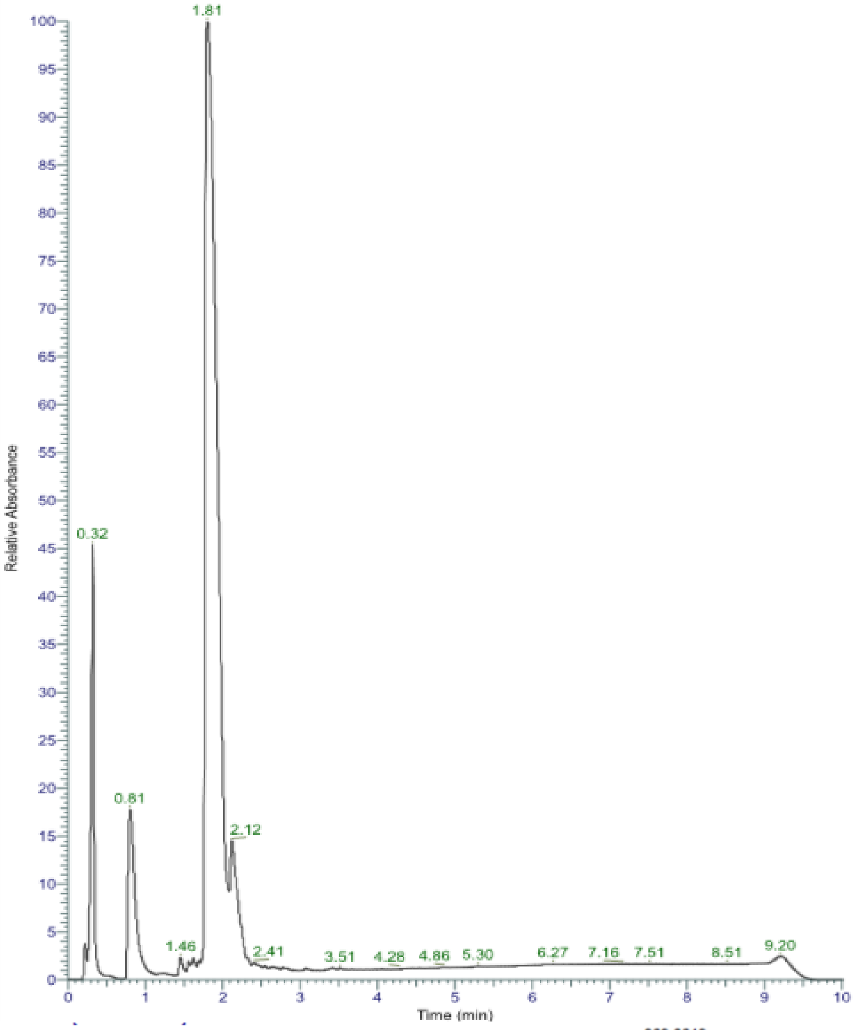


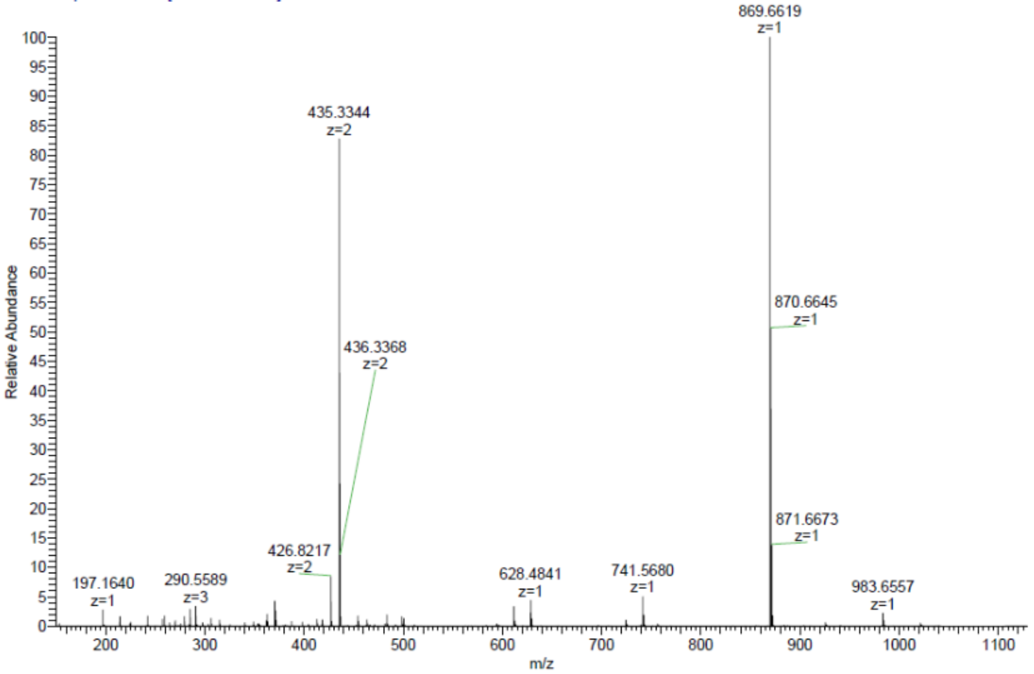

**G1KL** (20% v/v Dipropylamine, 60°C) was obtained as crude white solid after lyophilization (42.2 mg, 35.2%). Analytical RP-HPLC: $t_R$ = 1.79 min (A/D 100:0 to 0:100 in 7.00 min, λ = 214nm). HRMS (ESI+): $C_{42}H_{84}N_{12}O_7$ calc./obs. 869.66/869.66 Da $[M+H]^+$.

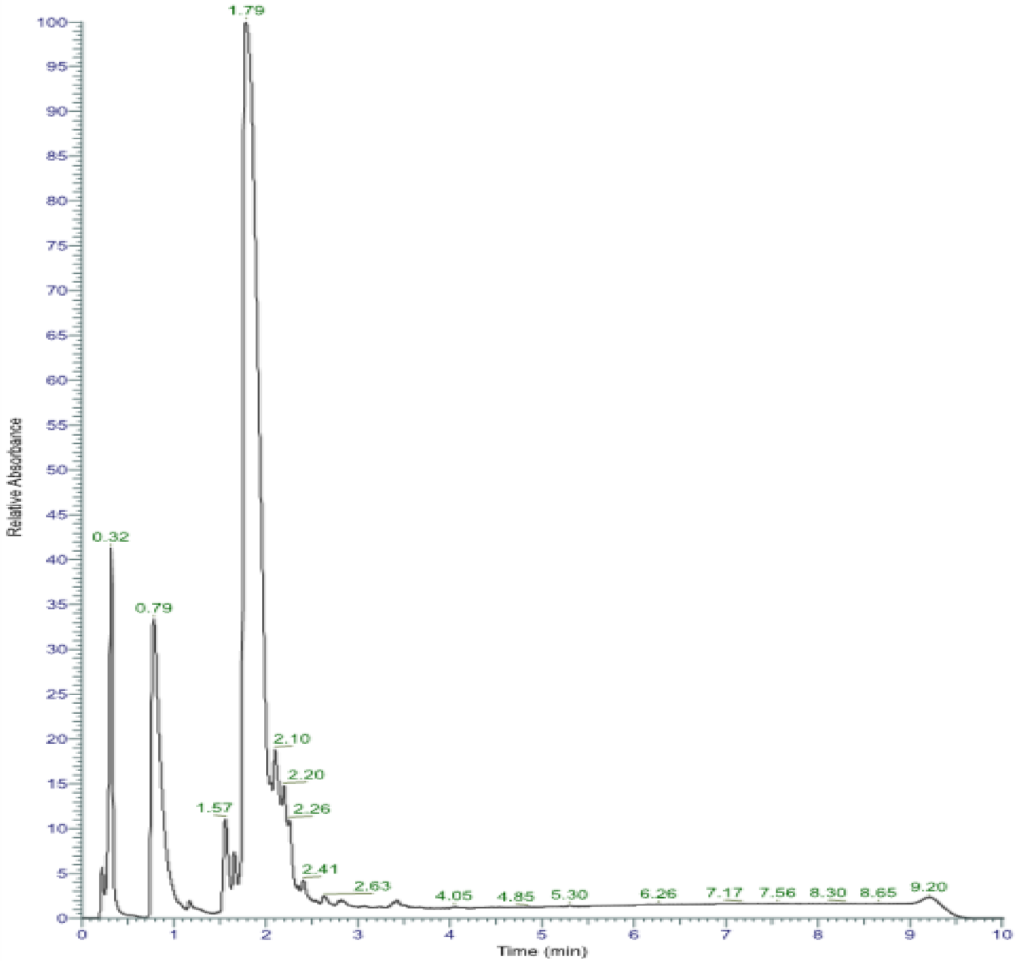


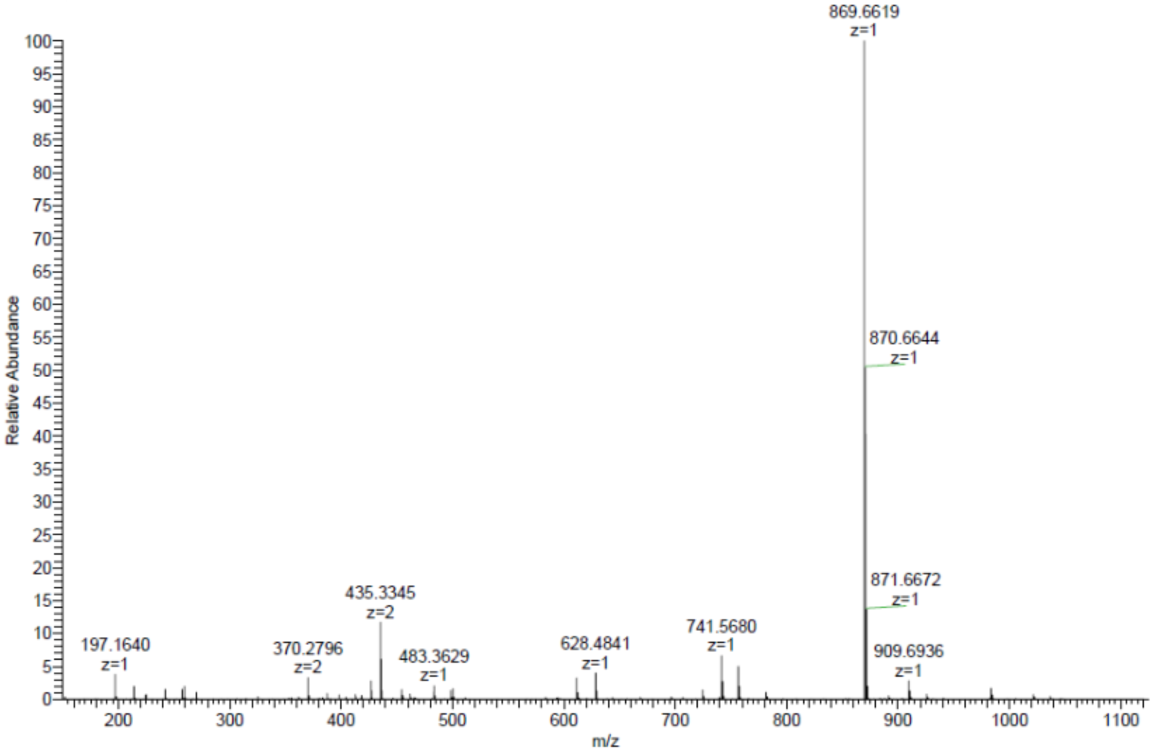

**G1KL** (20 v/v Dipropylamine + 1% v/v DBU, 60°C) was obtained as crude white solid after lyophilization (37.4 mg, 34.0%). Analytical RP-HPLC: $t_R$ = 1.83 min (A/D 100:0 to 0:100 in 7.00 min, $\lambda$ = 214nm). HRMS (ESI+): $C_{42}H_{84}N_{12}O_7$ calc./obs. 869.66/869.66 Da $[M+H]^+$.

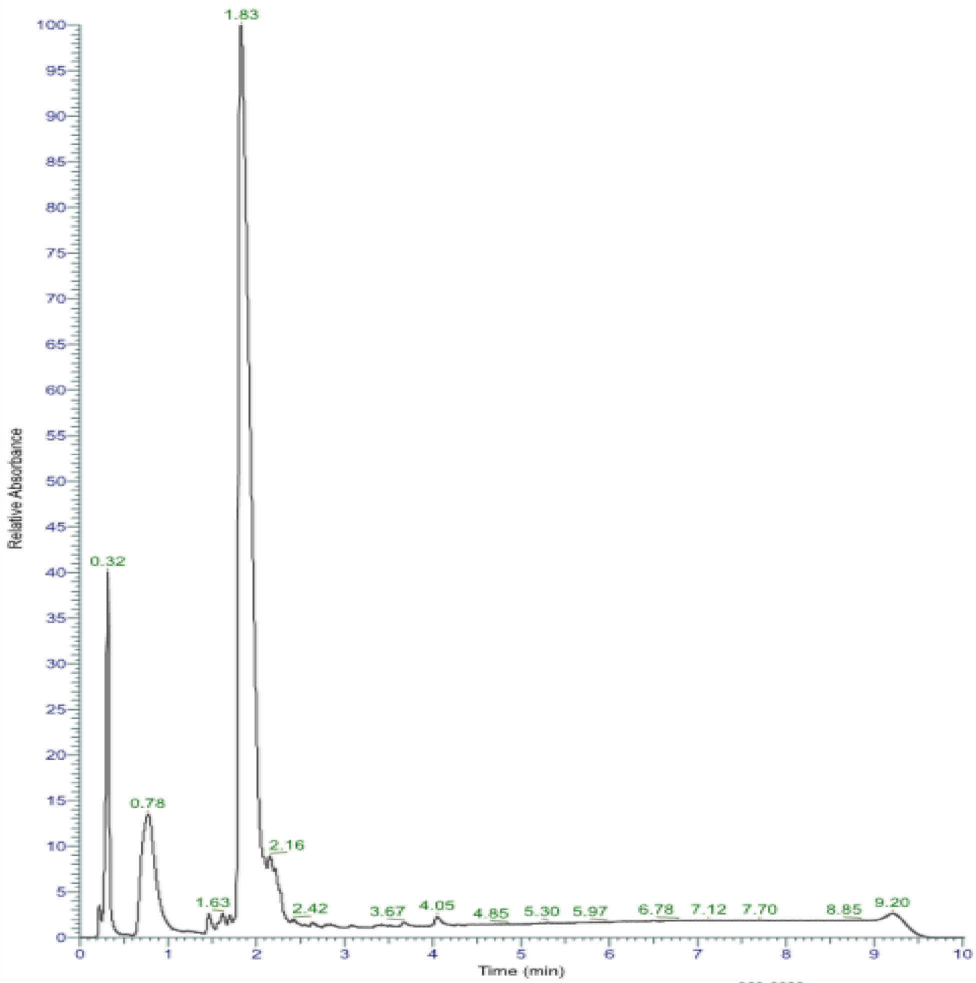


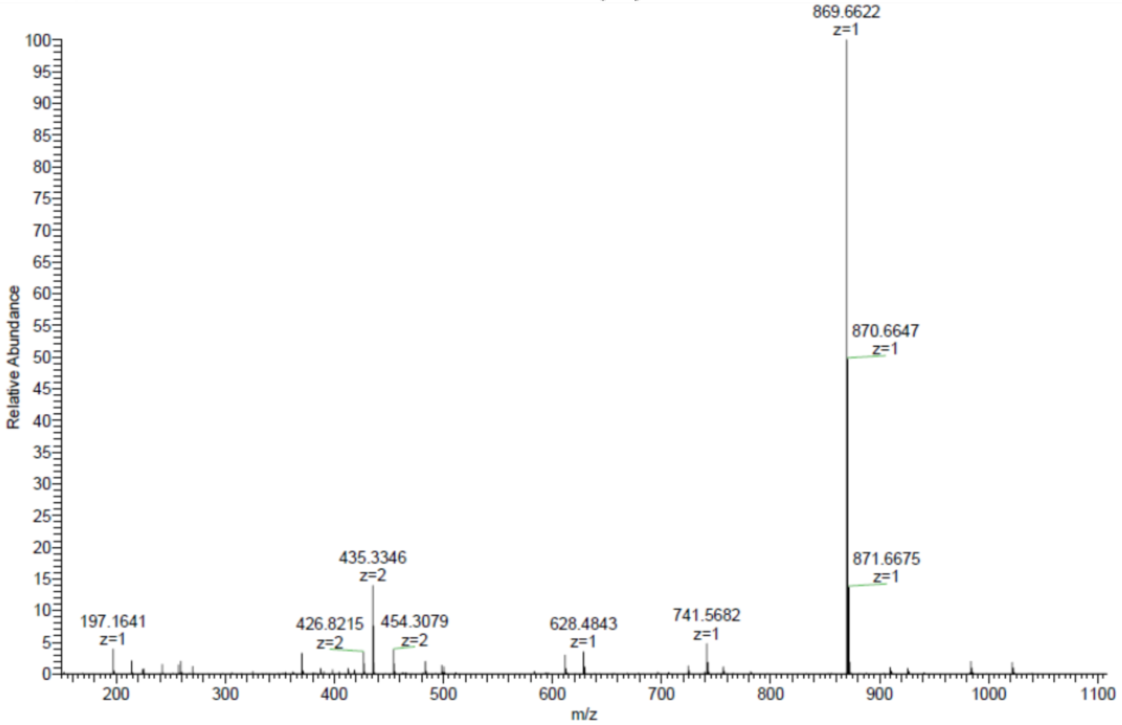

**G1KL** (25 v/v Dipropylamine, 60°C) was obtained as crude white solid after lyophilization (82.4 mg, 64.5%). Analytical RP-HPLC: $t_R$ = 1.88 min (A/D 100:0 to 0:100 in 7.00 min, λ = 214nm). HRMS (ESI+): $C_{42}H_{84}N_{12}O_7$ calc./obs. 869.66/869.66 Da $[M+H]^+$.

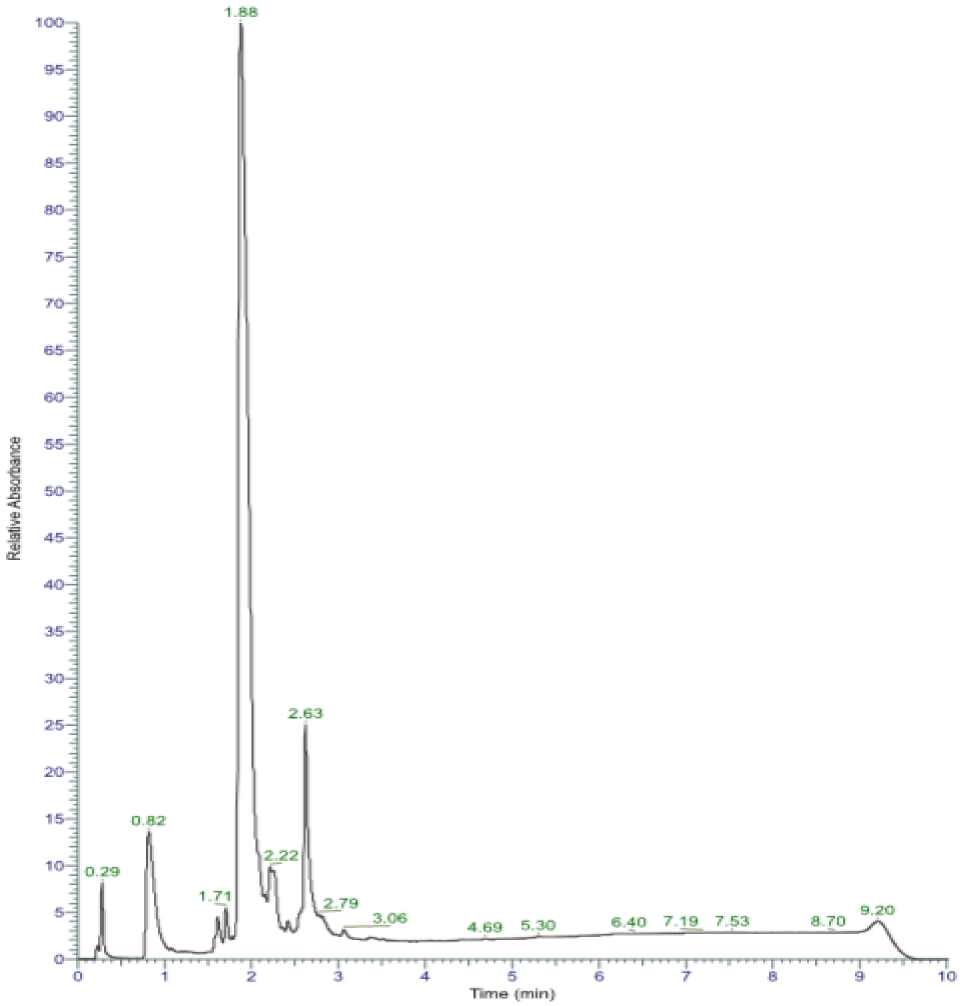


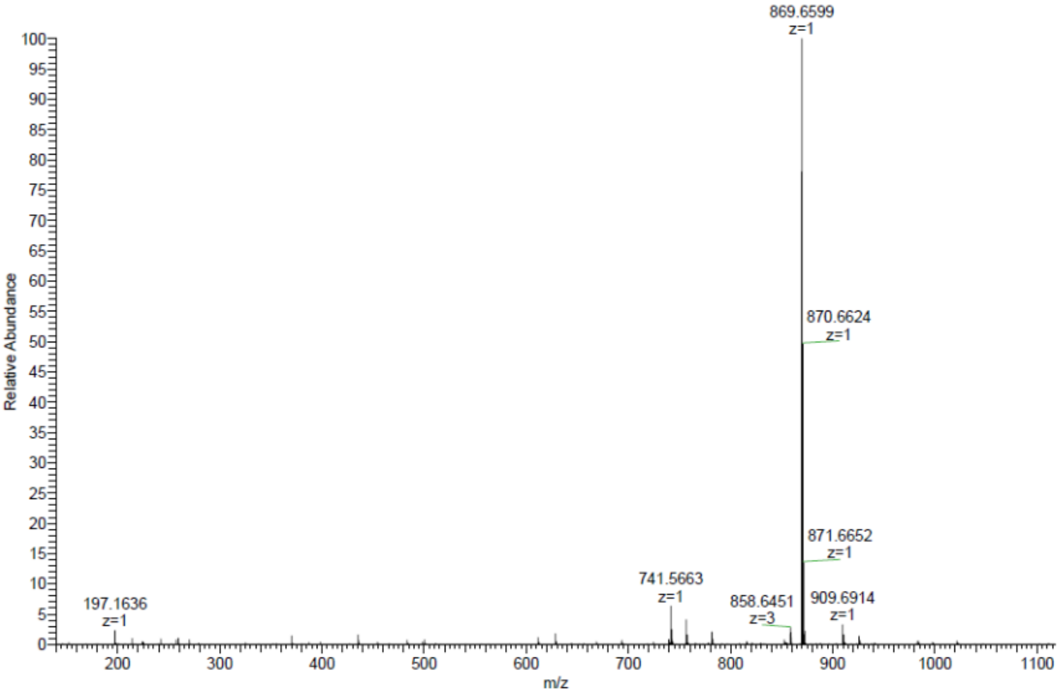

## 2.12 **G2KL** ((KL)$_4$(*K*KL)$_2$*K*KL)

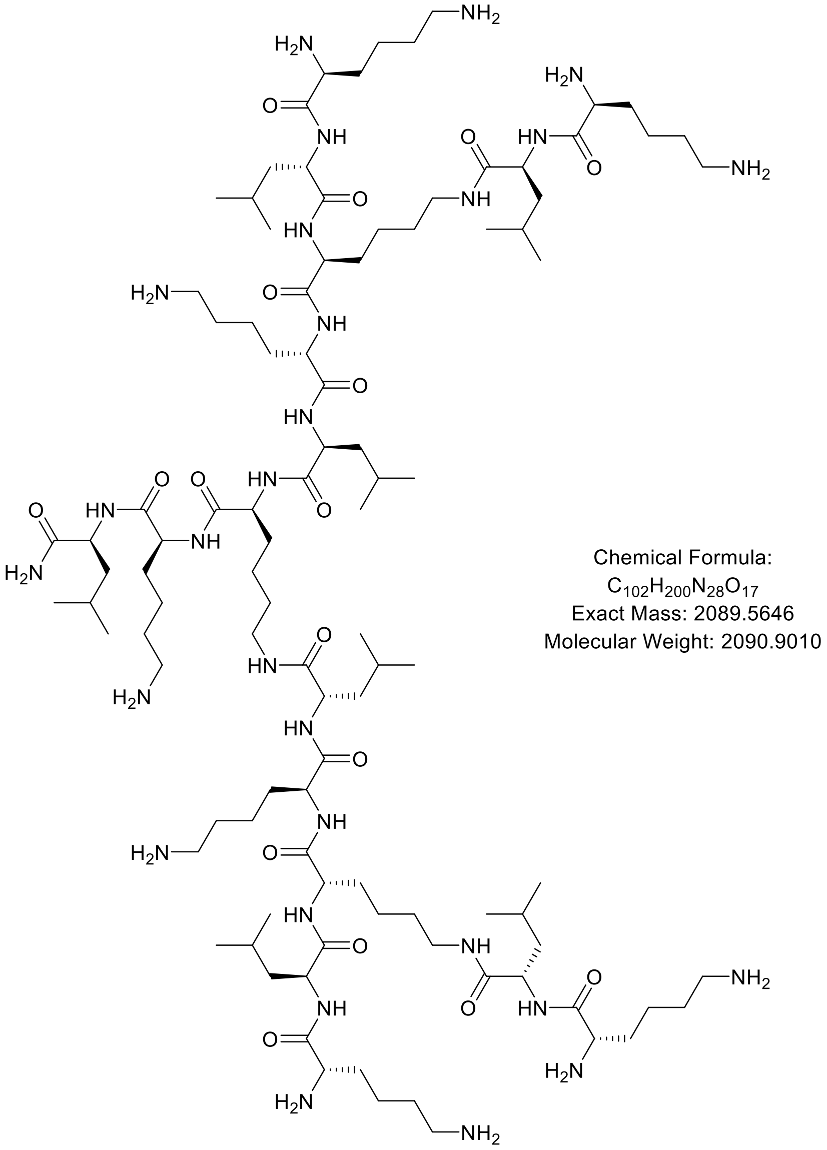

**G2KL** (20% v/v Piperidine, r. t.) was obtained as crude white solid after lyophilization (214.4 mg, 64.9%). Analytical RP-HPLC: $t_R$ = 2.35 min (A/D 100:0 to 0:100 in 7.00 min, λ = 214nm). HRMS (ESI+): $C_{102}H_{200}N_{28}O_{17}$ calc./obs. 2090.56/2090.56 Da $[M+H]^+$.

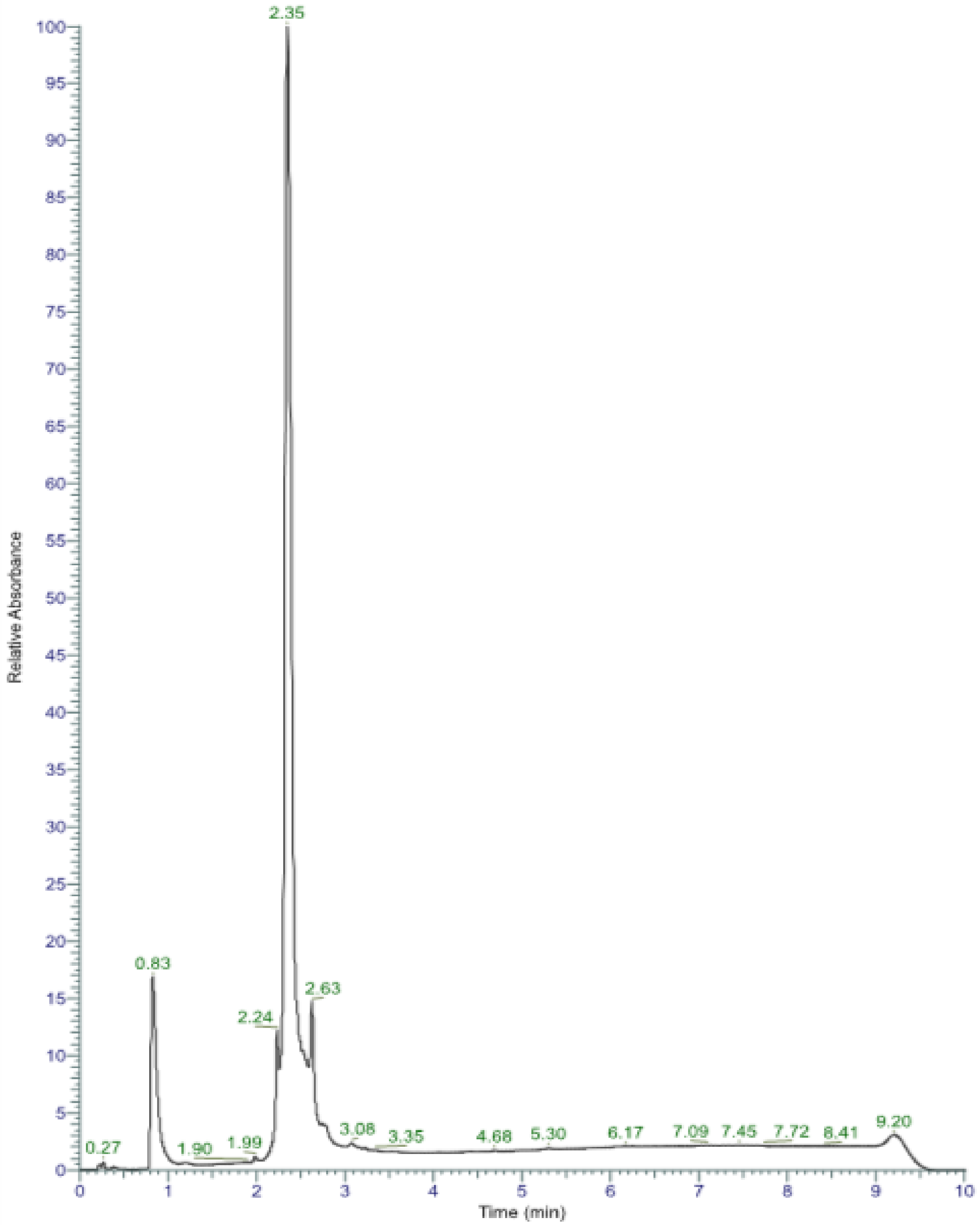

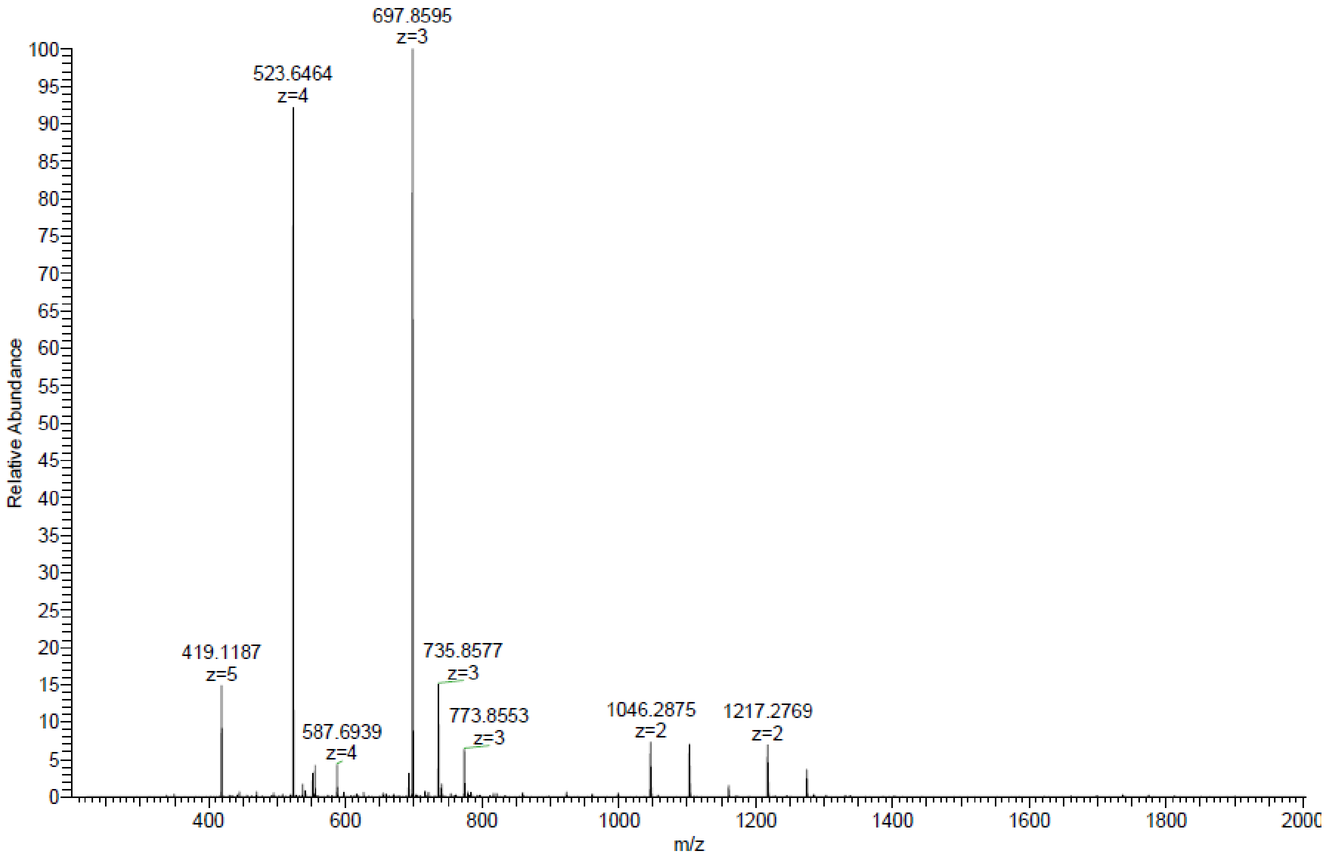
697.8595
z=3
523.6464
z=4
419.1187
z=5
735.8577
z=3
587.6939
z=4
773.8553
z=3
1046.2875
z=2
1217.2769
z=2
Relative Abundance
m/z

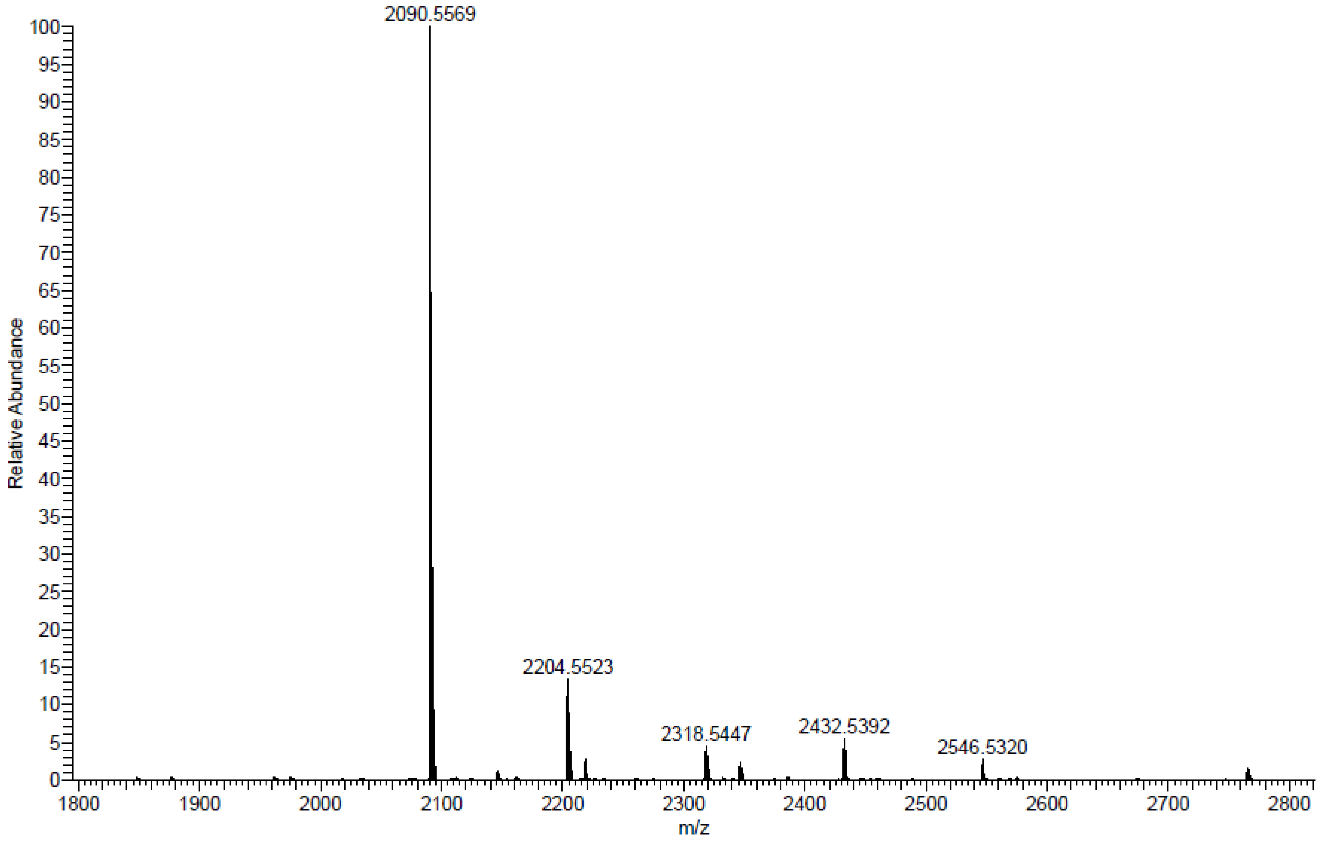
2090.5569
2204.5523
2318.5447
2432.5392
2546.5320
Relative Abundance
m/z

**G2KL** (5% w/v Piperazine + 2% DBU, r. t.) was obtained as crude white solid after lyophilization (189.9 mg, 53.9%). Analytical RP-HPLC: $t_R$ = 2.32 min (A/D 100:0 to 0:100 in 7.00 min, $\lambda$ = 214nm). HRMS (ESI+): $C_{102}H_{200}N_{28}O_{17}$ calc./obs. 2090.56/2090.56 Da $[M+H]^+$.

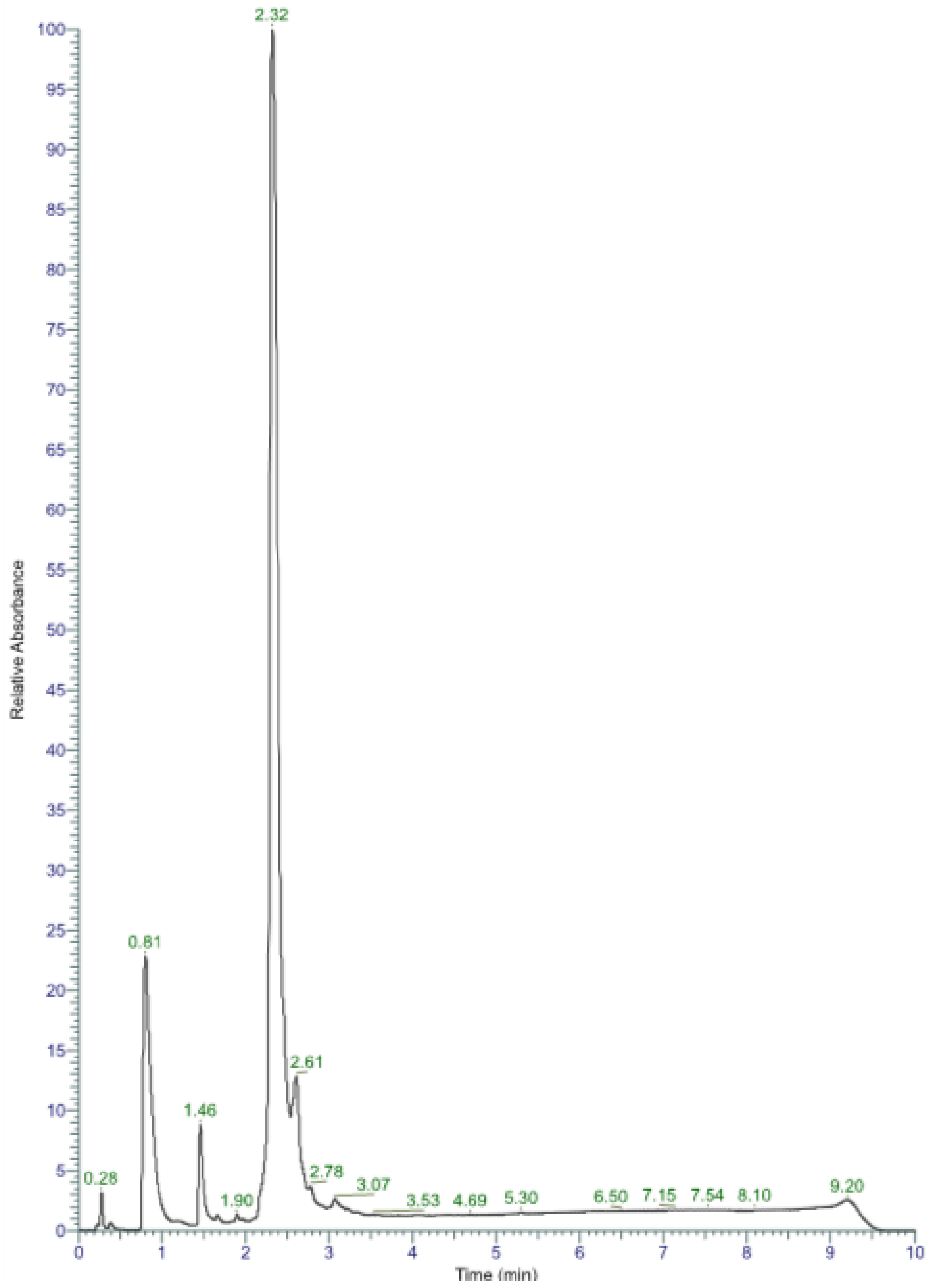

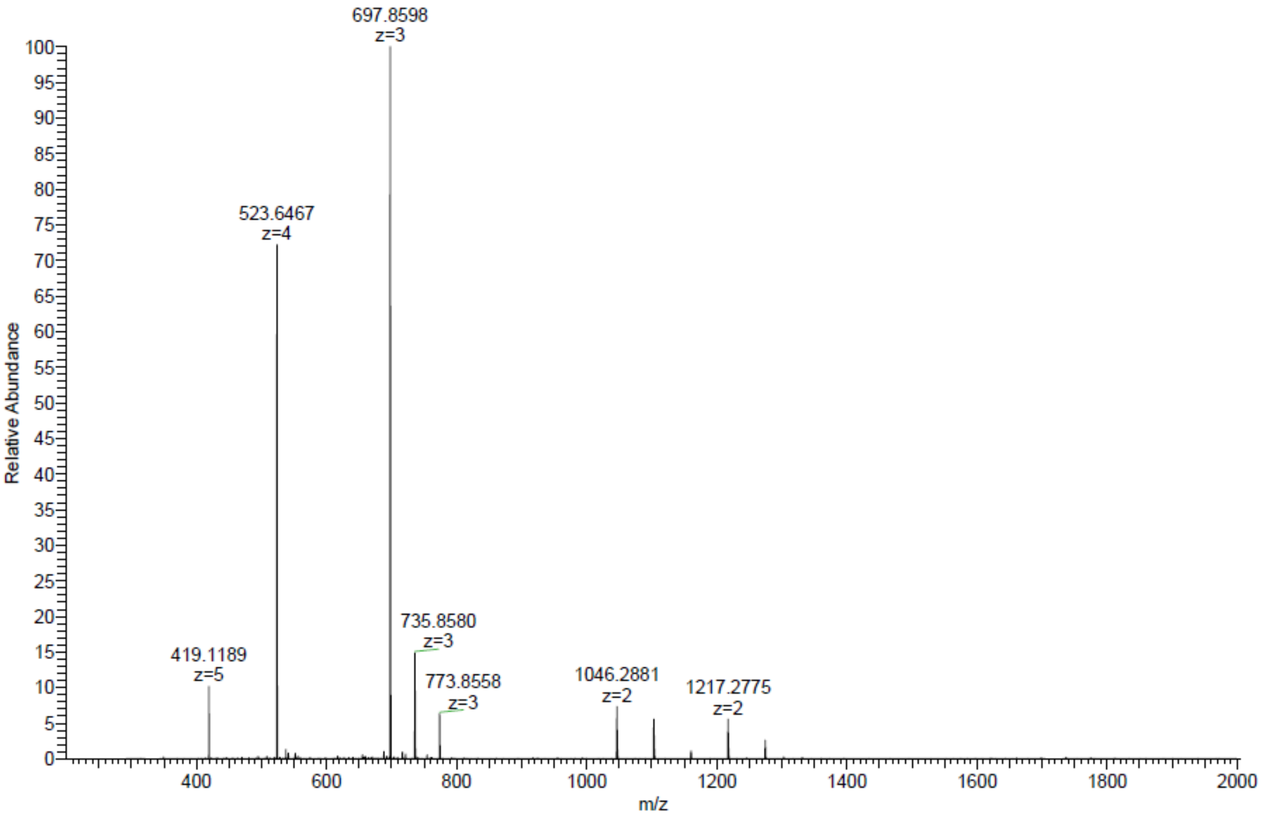
697.8598
z=3
523.6467
z=4
735.8580
z=3
419.1189
z=5
773.8558
z=3
1046.2881
z=2
1217.2775
z=2
Relative Abundance
m/z

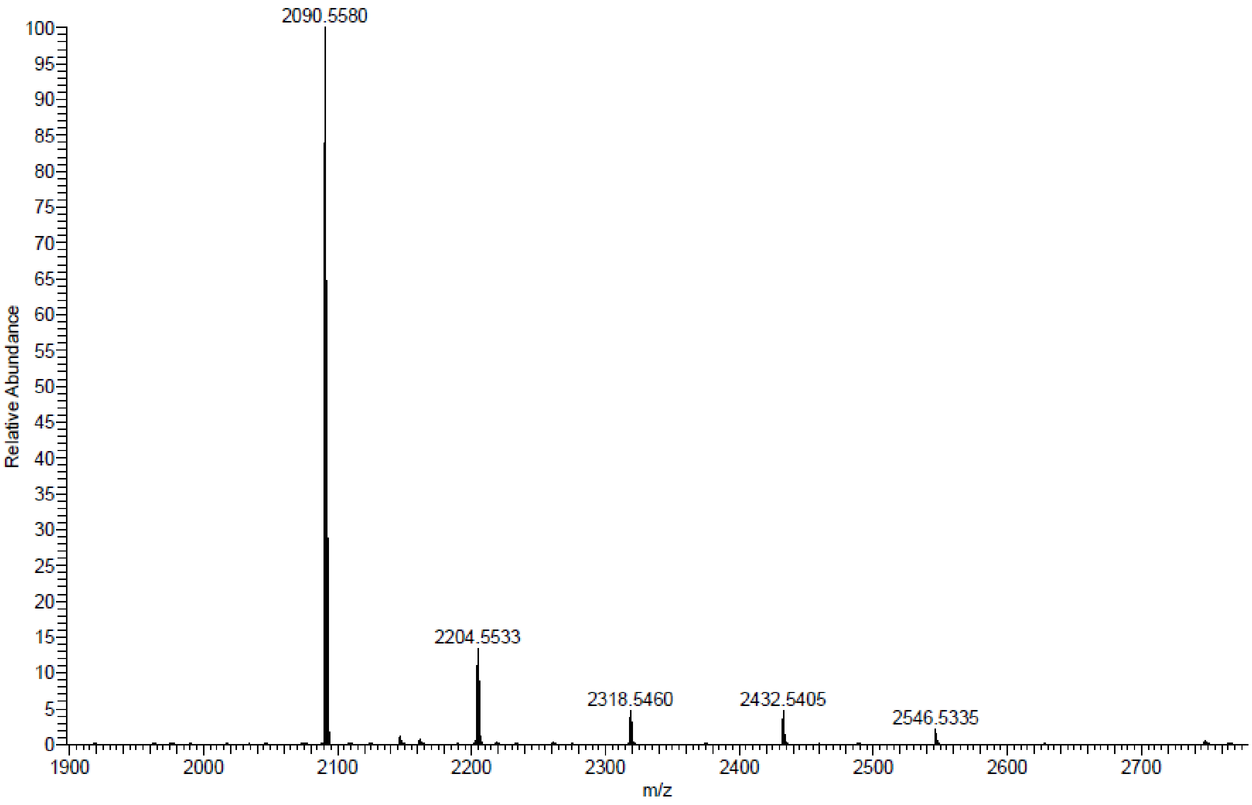
2090.5580
2204.5533
2318.5460
2432.5405
2546.5335
Relative Abundance
m/z

**G2KL** (20% v/v Dipropylamine, r. t.) was obtained as crude white solid after lyophilization (134.4 mg, 42.2%). Analytical RP-HPLC: $t_R$ = 2.40 min (A/D 100:0 to 0:100 in 7.00 min, λ = 214nm). HRMS (ESI+): $C_{102}H_{200}N_{28}O_{17}$ calc./obs. 2090.56/2090.56 Da $[M+H]^+$.

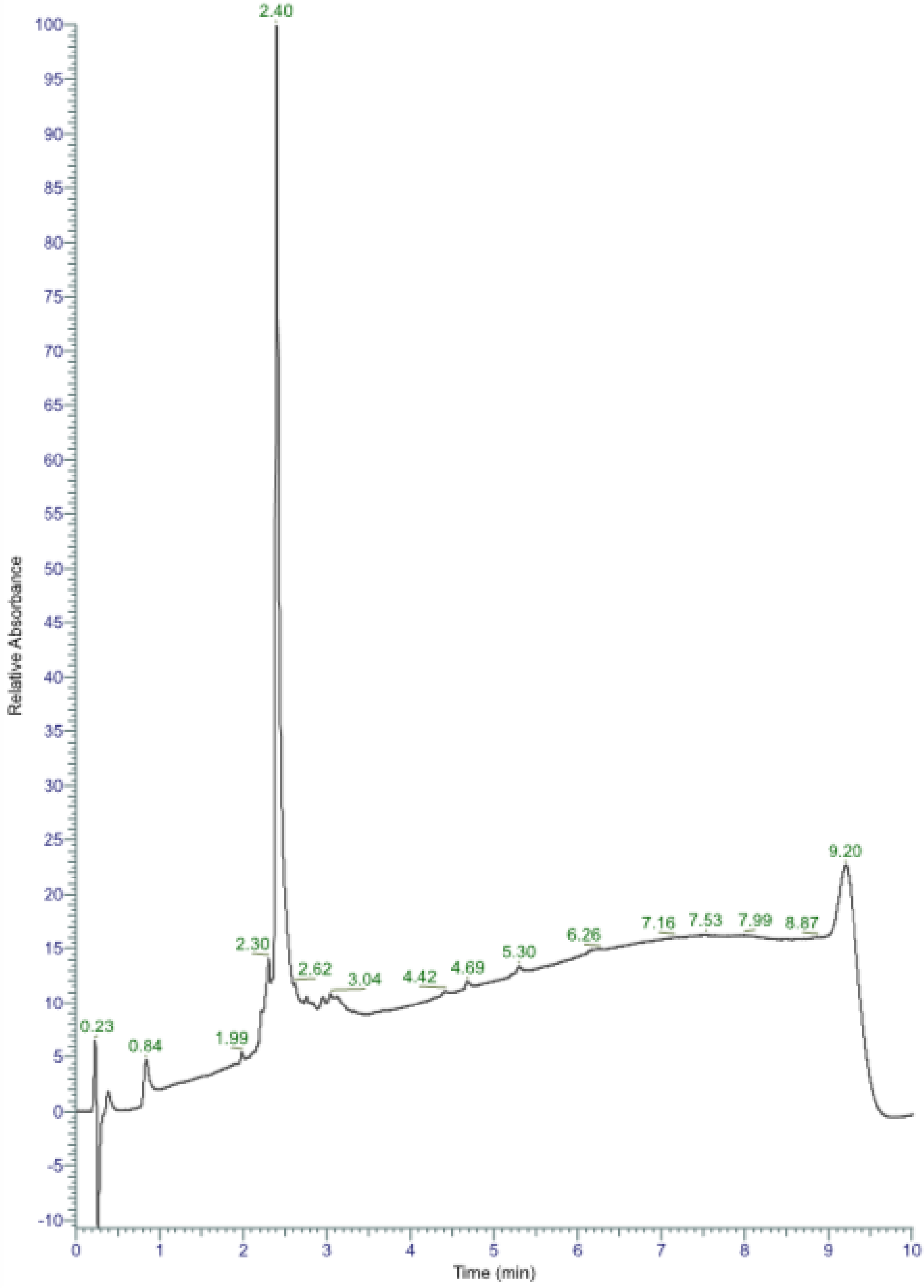

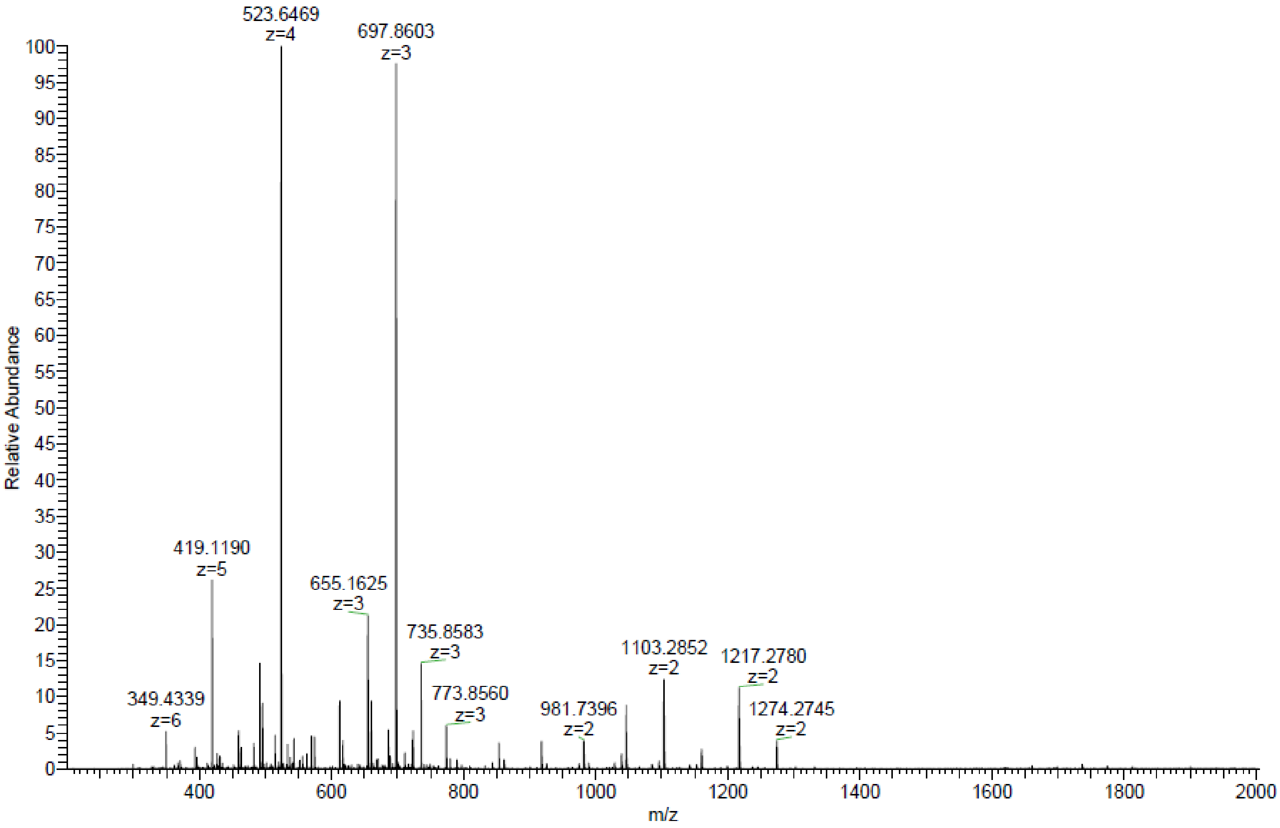

523.6469
z=4
697.8603
z=3
419.1190
z=5
655.1625
z=3
735.8583
z=3
773.8560
z=3
349.4339
z=6
981.7396
z=2
1103.2852
z=2
1217.2780
z=2
1274.2745
z=2
Relative Abundance
m/z


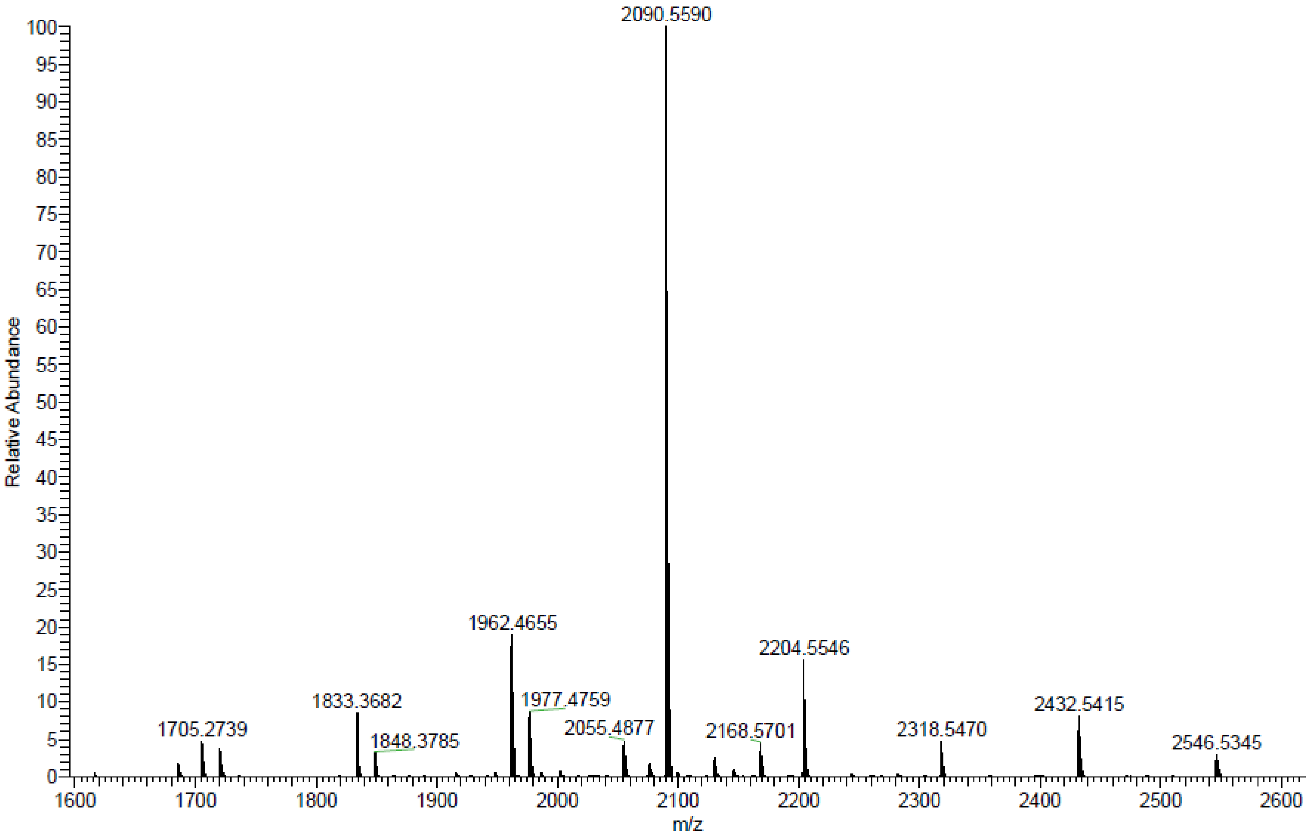

2090.5590
1962.4655
2204.5546
1833.3682
1977.4759
2432.5415
1705.2739
1848.3785
2055.4877
2168.5701
2318.5470
2546.5345
Relative Abundance
m/z

**G2KL** (25% v/v Dipropylamine, r. t.) was obtained as crude white solid after lyophilization (151.4 mg, 46.4%). Analytical RP-HPLC: $t_R$ = 2.38 min (A/D 100:0 to 0:100 in 7.00 min, λ = 214nm). HRMS (ESI+): $C_{102}H_{200}N_{28}O_{17}$ calc./obs. 2090.56/2090.56 Da $[M+H]^+$.

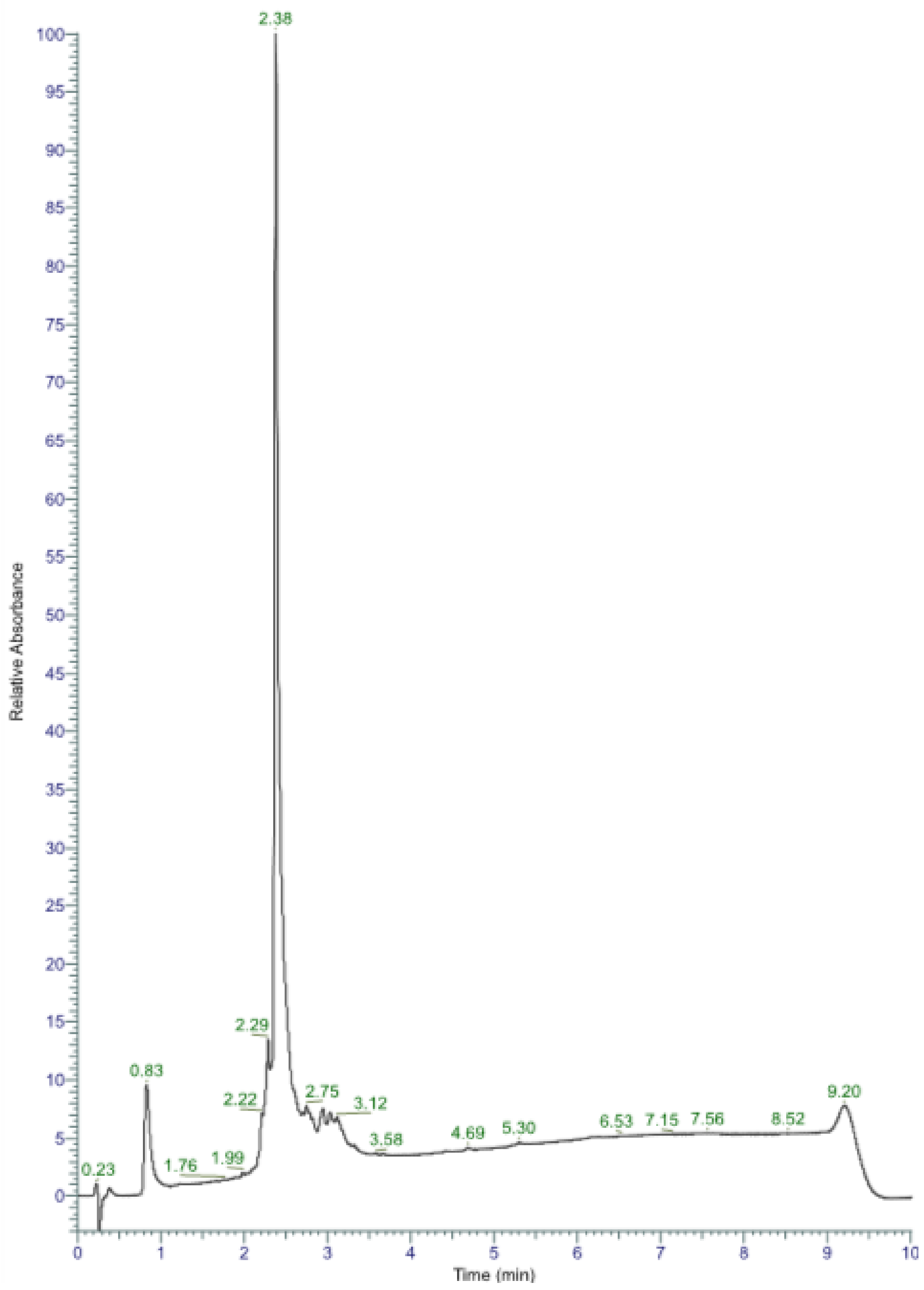

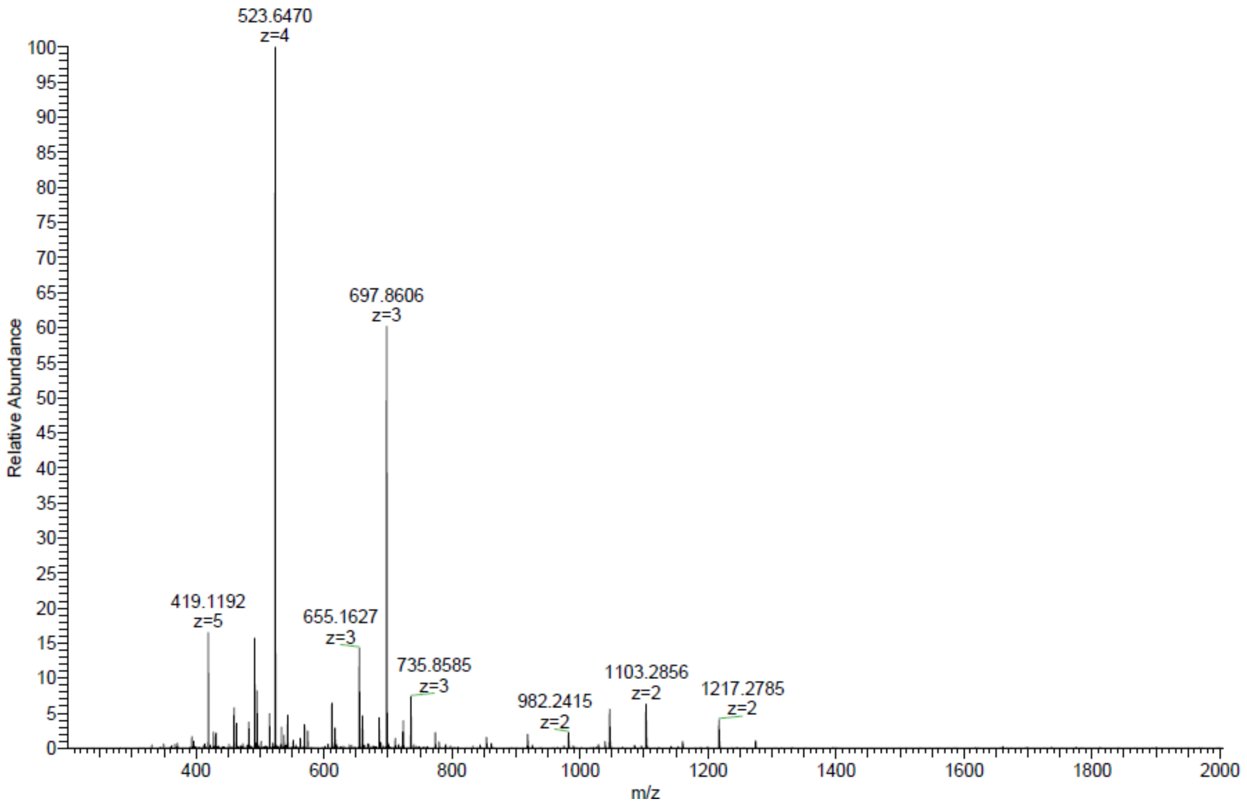

523.6470
z=4
697.8606
z=3
419.1192
z=5
655.1627
z=3
735.8585
z=3
982.2415
z=2
1103.2856
z=2
1217.2785
z=2
Relative Abundance
m/z


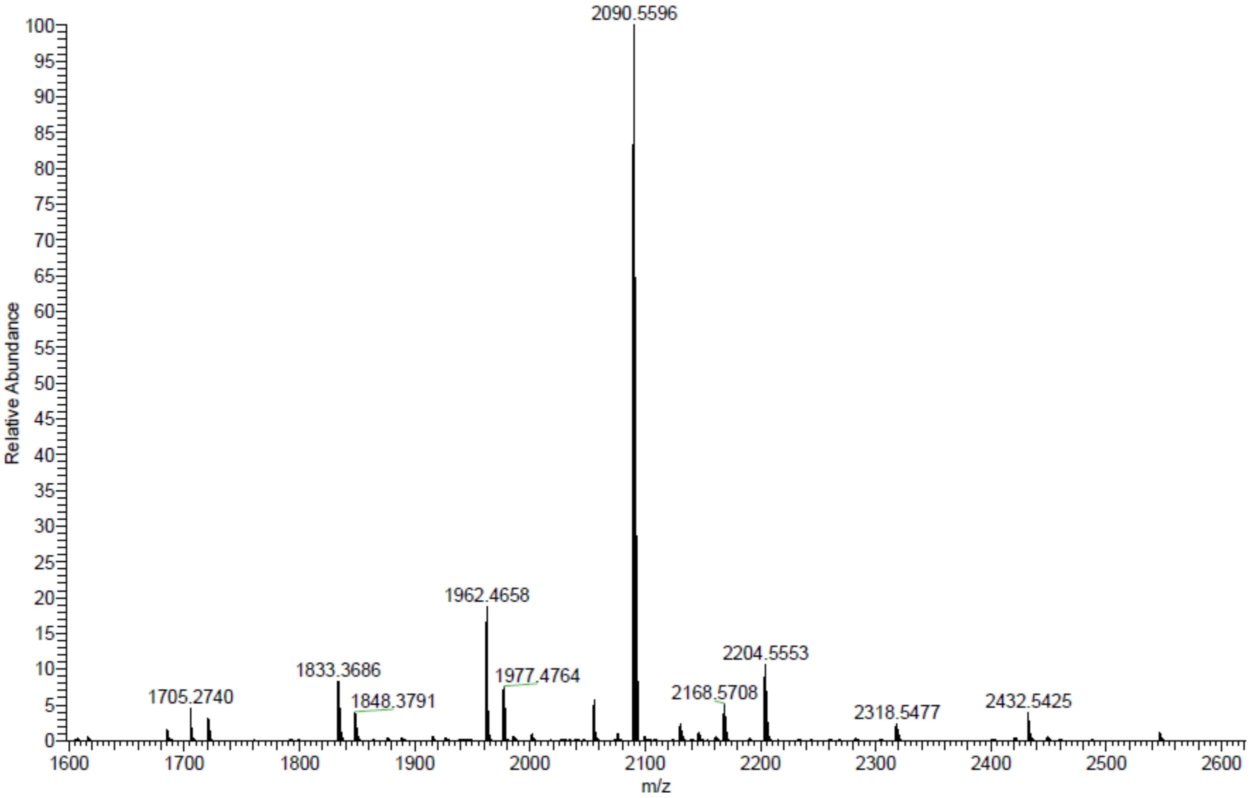

2090.5596
1962.4658
1833.3686
1848.3791
1977.4764
1705.2740
2204.5553
2168.5708
2318.5477
2432.5425
Relative Abundance
m/z

## 2.13 **G3KL** ((KL)$_8$(*K*KL)$_4$(*K*KL)$_2$*K*KL)

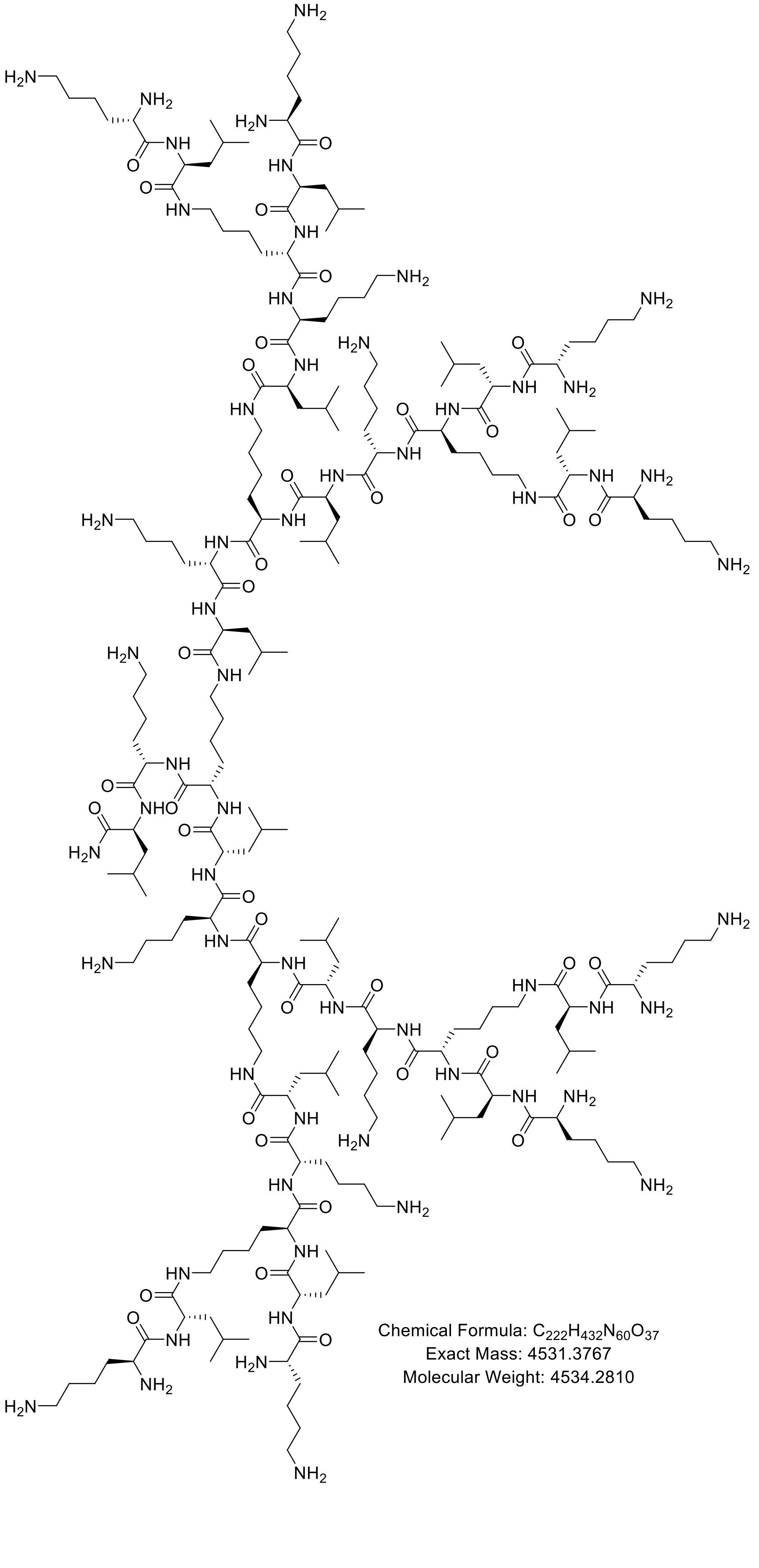

Chemical Formula: $C_{222}H_{432}N_{60}O_{37}$
Exact Mass: 4531.3767
Molecular Weight: 4534.2810

**G3KL** (20% v/v Piperidine, 60°C) was obtained as crude white solid after lyophilization (366.6 mg, 46.8%). Analytical RP-HPLC: $t_R$ = 2.77 min (A/D 100:0 to 0:100 in 7.00 min, λ = 214nm). HRMS (ESI+): $C_{222}H_{432}N_{60}O_{37}$ calc./obs. 4532.38/4532.39 Da $[M+H]^+$.

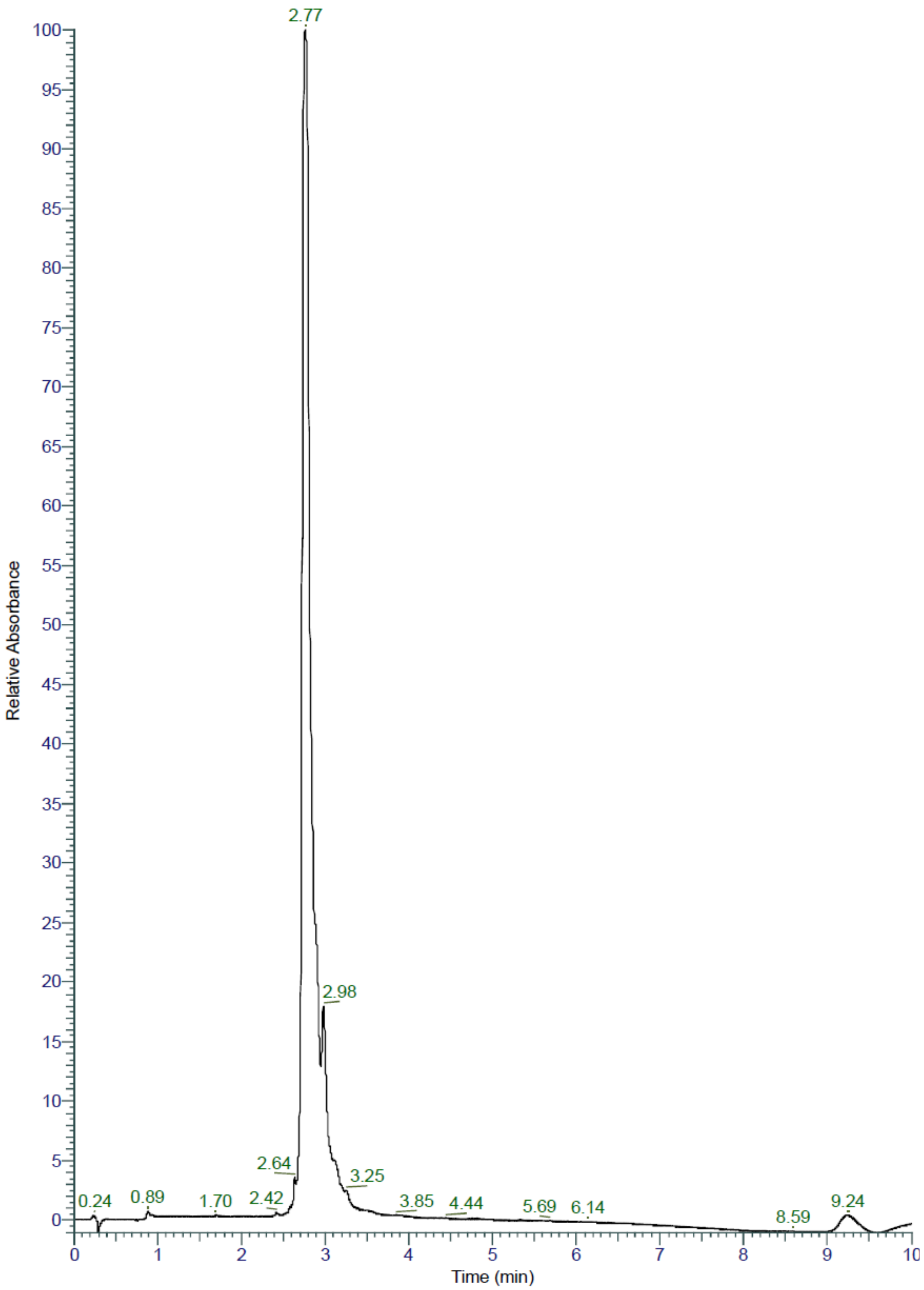

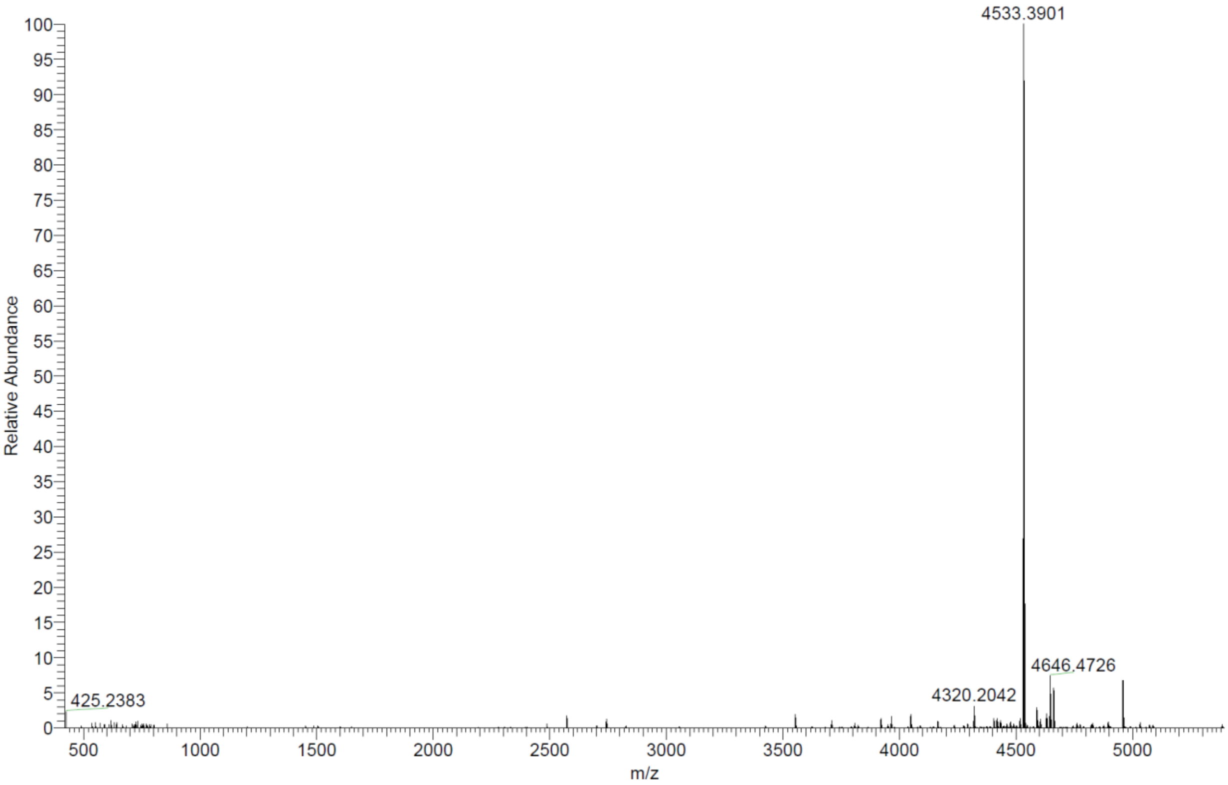
4533.3901
4646.4726
4320.2042
425.2383
Relative Abundance
m/z

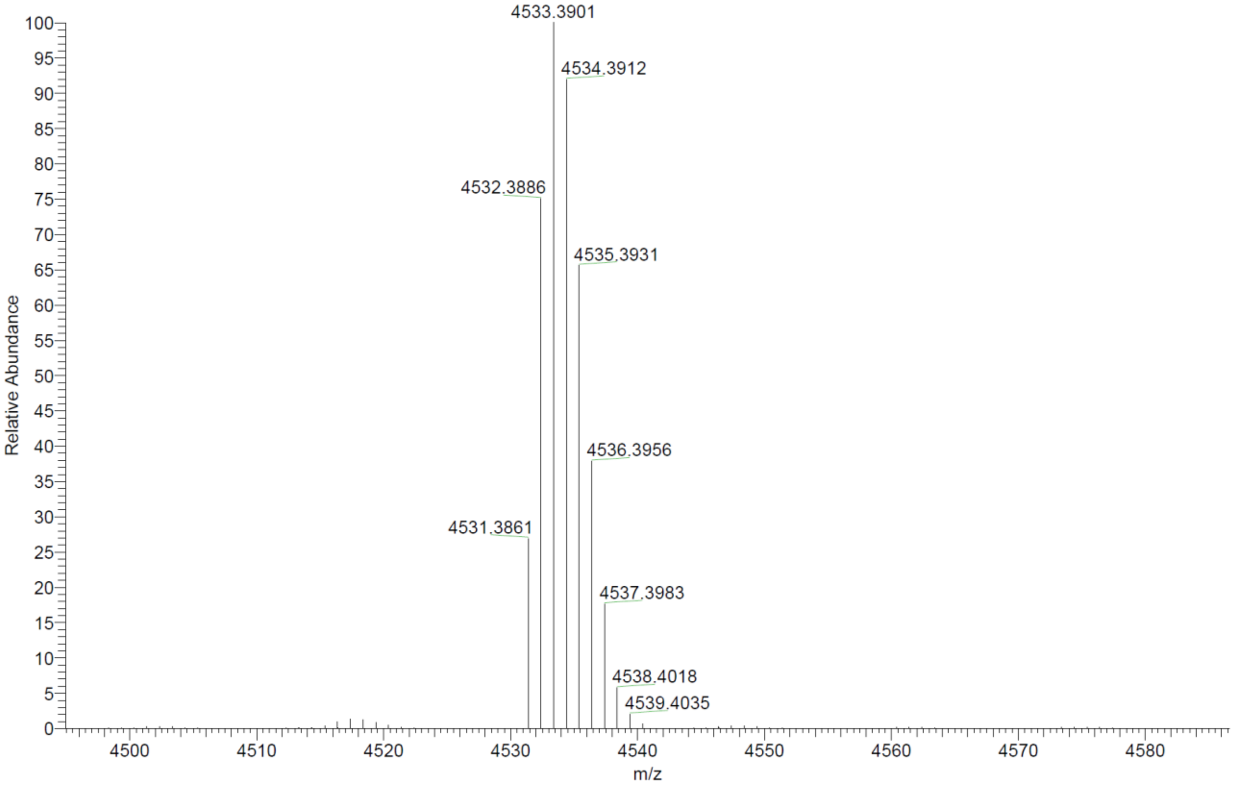
4533.3901
4534.3912
4532.3886
4535.3931
4536.3956
4531.3861
4537.3983
4538.4018
4539.4035
Relative Abundance
m/z

**G3KL** (25% v/v Dipropylamine, 60°C) was obtained as crude white solid after lyophilization (255.1 mg, n.d.). Analytical RP-HPLC: $t_R$ = 2.79 min (A/D 100:0 to 0:100 in 7.00 min, λ = 214nm). HRMS (ESI+): $C_{222}H_{432}N_{60}O_{37}$ calc./obs. 4532.38/4532.39 Da $[M+H]^+$.

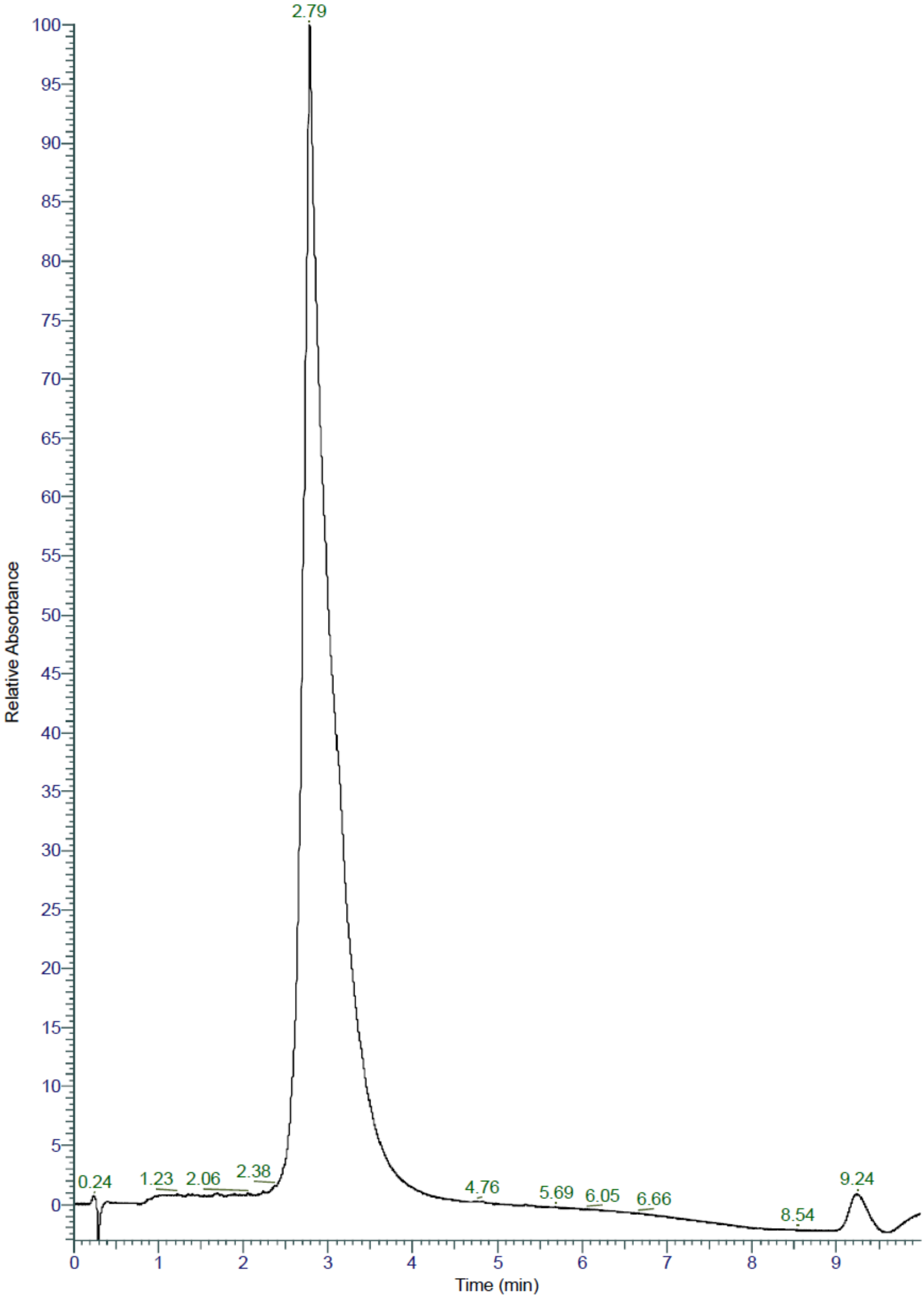

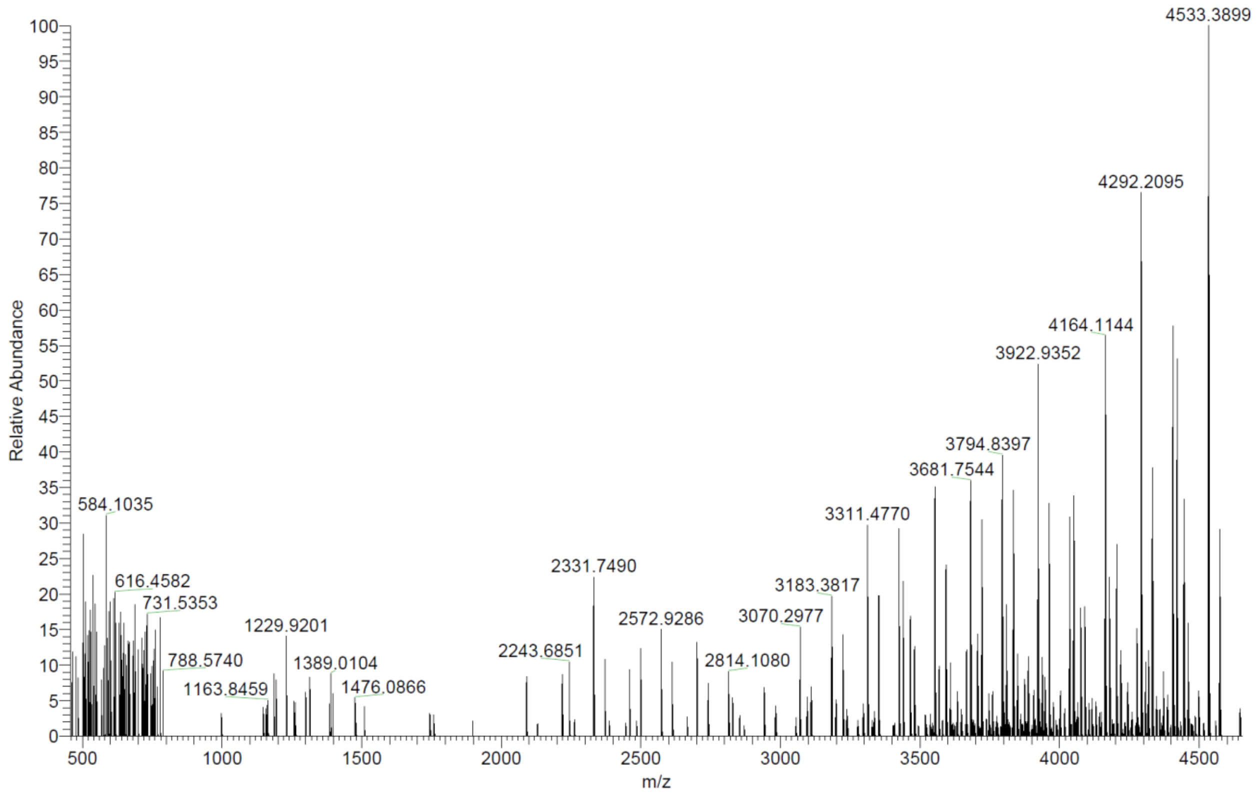
Relative Abundance
m/z
4533.3899
4292.2095
4164.1144
3922.9352
3794.8397
3681.7544
3311.4770
3183.3817
3070.2977
2814.1080
2572.9286
2331.7490
2243.6851
1476.0866
1389.0104
1229.9201
1163.8459
788.5740
731.5353
616.4582
584.1035

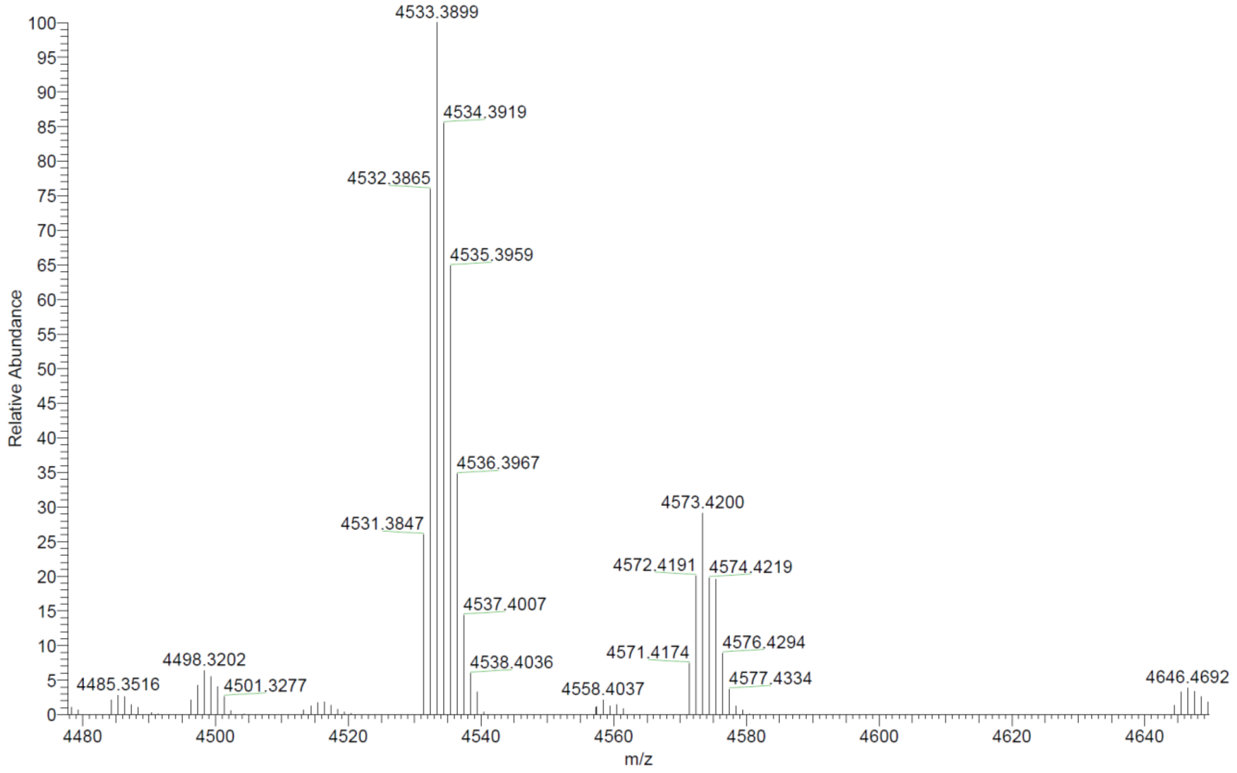
Relative Abundance
m/z
4533.3899
4534.3919
4532.3865
4535.3959
4536.3967
4531.3847
4537.4007
4538.4036
4573.4200
4572.4191
4574.4219
4571.4174
4576.4294
4577.4334
4558.4037
4498.3202
4485.3516
4501.3277
4646.4692

**G3KL** (25% v/v Dipropylamine (+ 1% v/v DBU for the last generation), 60°C) was obtained as crude white solid after lyophilization (252.0 mg, n.d.). Analytical RP-HPLC: $t_R$ = 2.79 min (A/D 100:0 to 0:100 in 7.00 min, λ = 214nm). HRMS (ESI+): $C_{222}H_{432}N_{60}O_{37}$ calc./obs. 4532.38/4532.39 Da $[M+H]^+$.

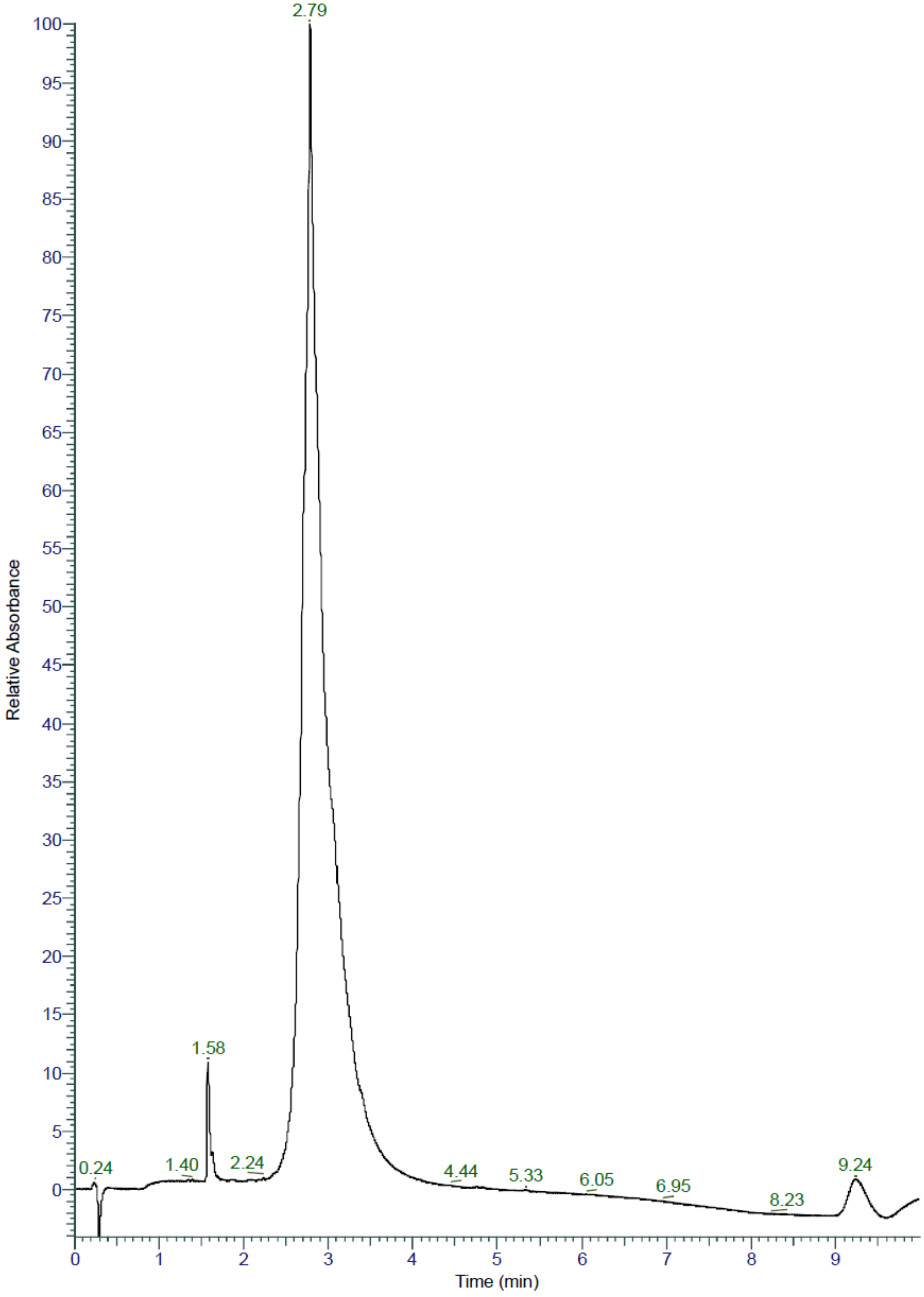

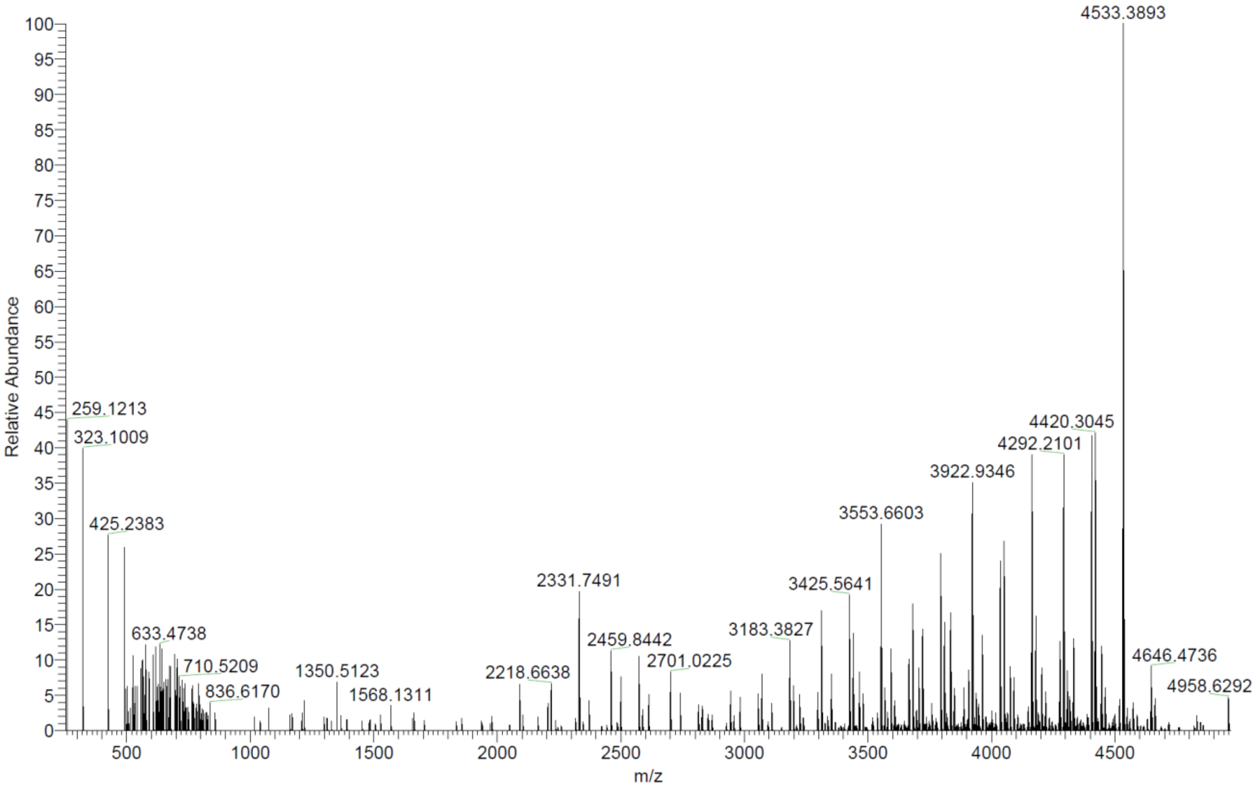

4533.3893
4420.3045
4292.2101
3922.9346
3553.6603
3425.5641
3183.3827
2331.7491
2459.8442
2701.0225
2218.6638
1350.5123
1568.1311
259.1213
323.1009
425.2383
633.4738
710.5209
836.6170
4646.4736
4958.6292
Relative Abundance
m/z


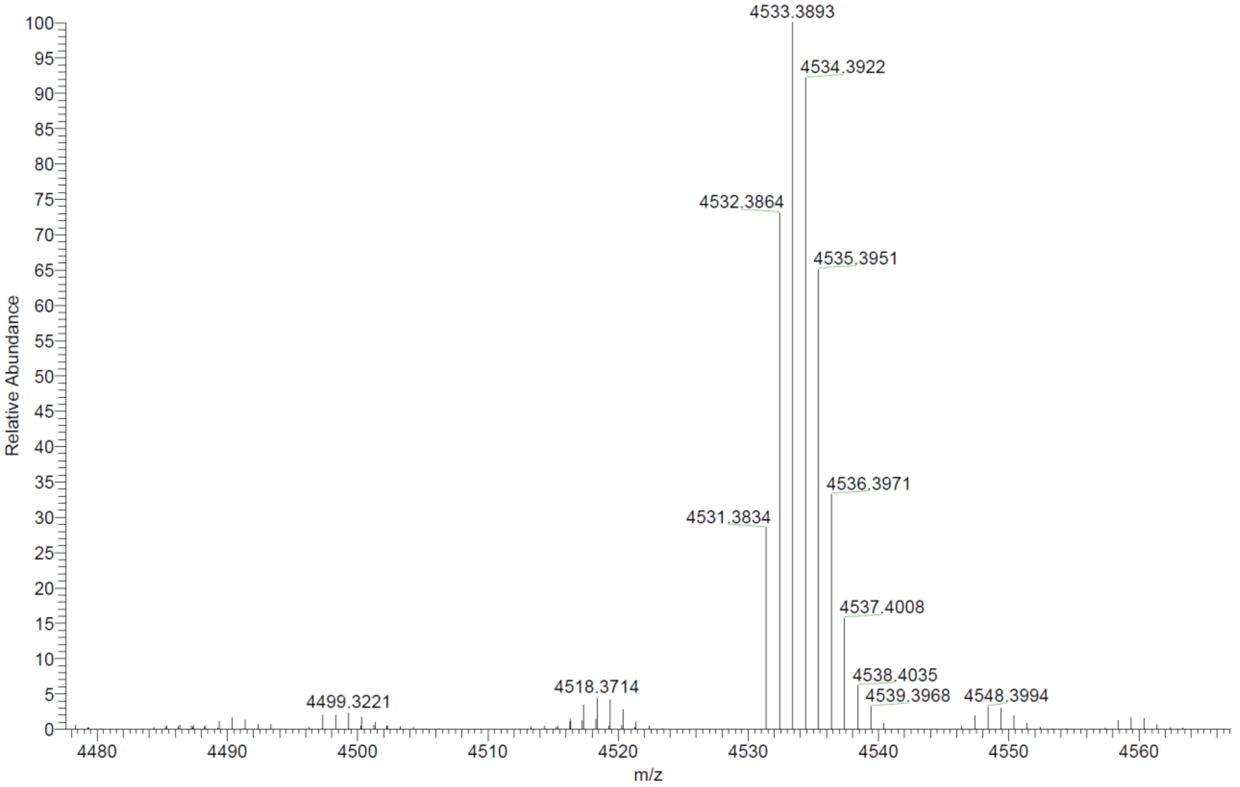

4533.3893
4534.3922
4532.3864
4535.3951
4536.3971
4531.3834
4537.4008
4538.4035
4539.3968
4548.3994
4518.3714
4499.3221
Relative Abundance
m/z

**G3KL** (2% v/v DBU, 60°C) was obtained as crude white solid after lyophilization (400.6 mg, 48.4%). Analytical RP-HPLC: $t_R$ = 2.75 min (A/D 100:0 to 0:100 in 7.00 min, λ = 214nm). HRMS (ESI+): $C_{222}H_{432}N_{60}O_{37}$ calc./obs. 4532.38/4532.39 Da $[M+H]^+$.

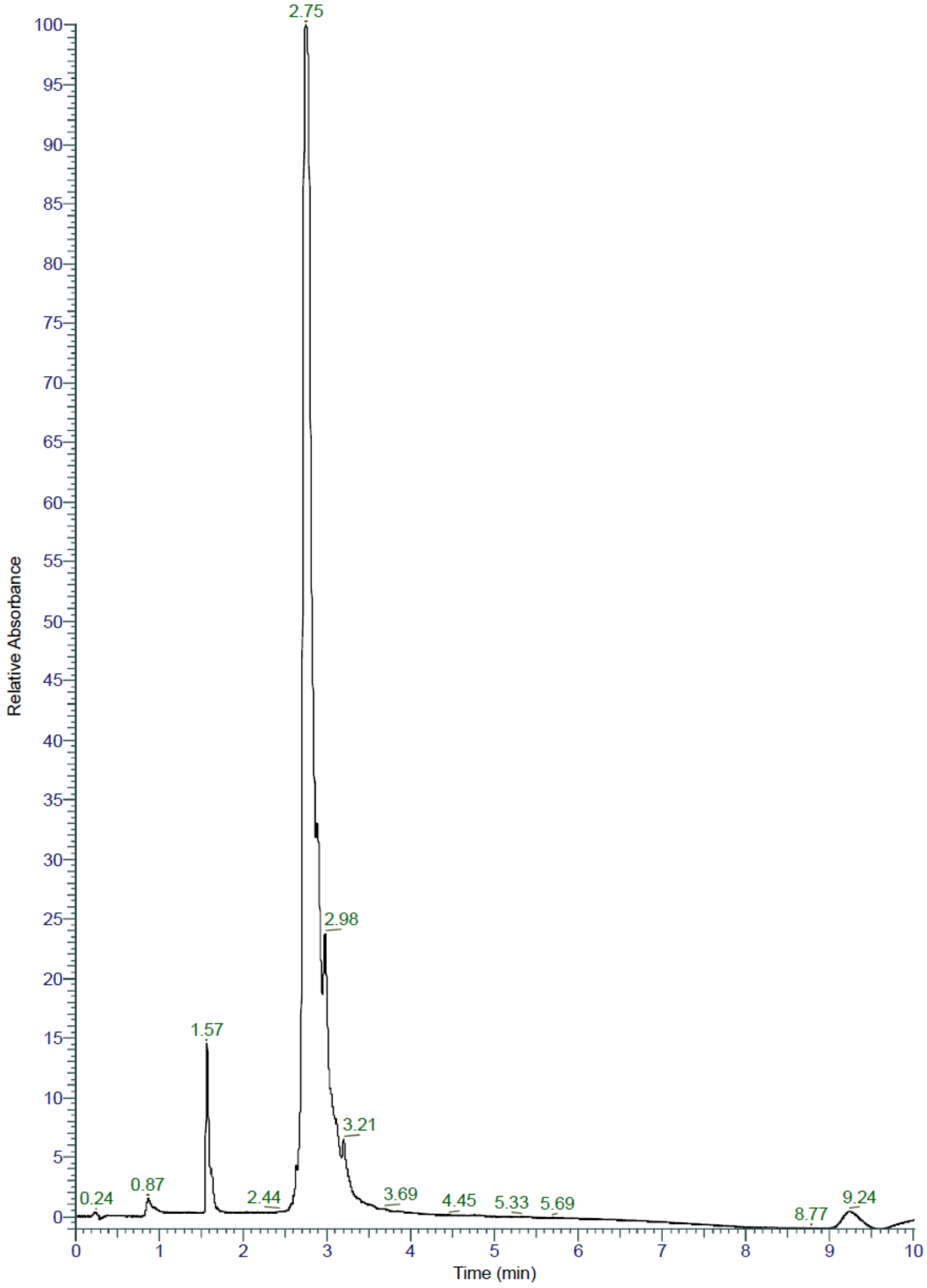

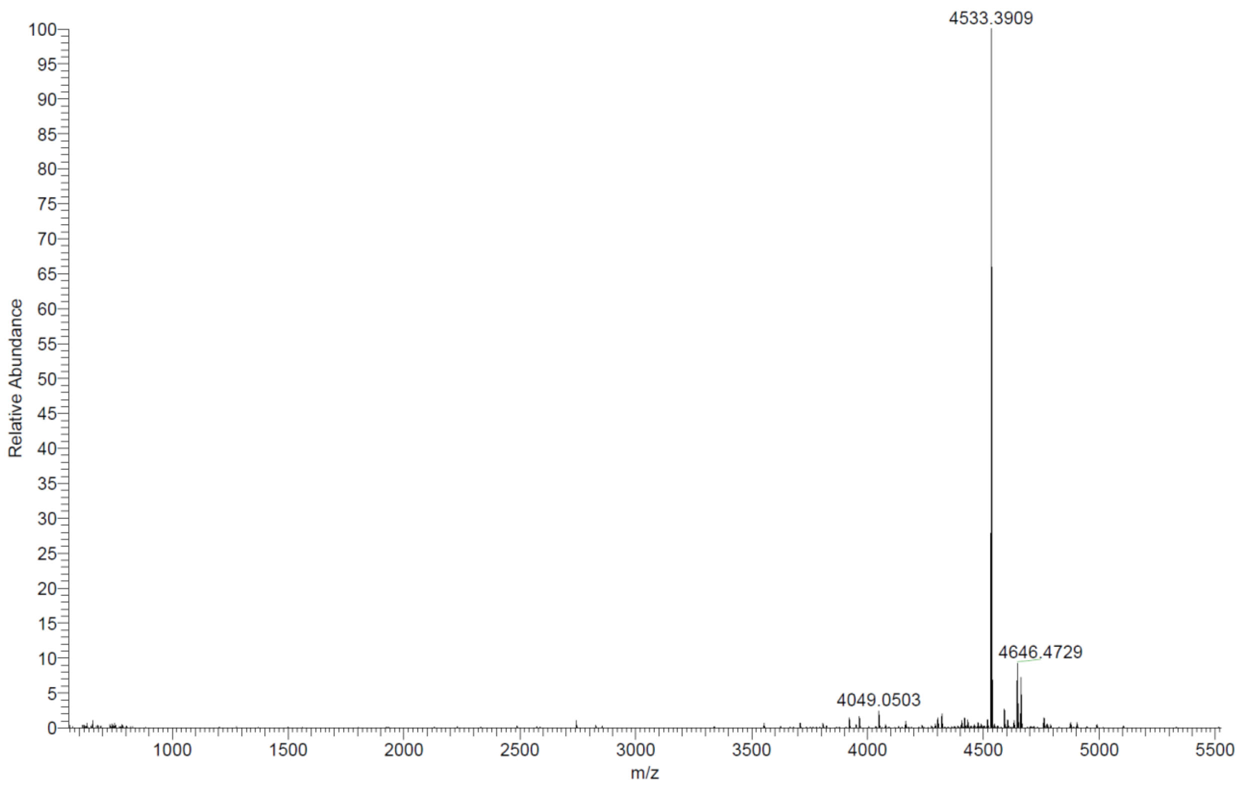
4533.3909
4646.4729
4049.0503
Relative Abundance
m/z

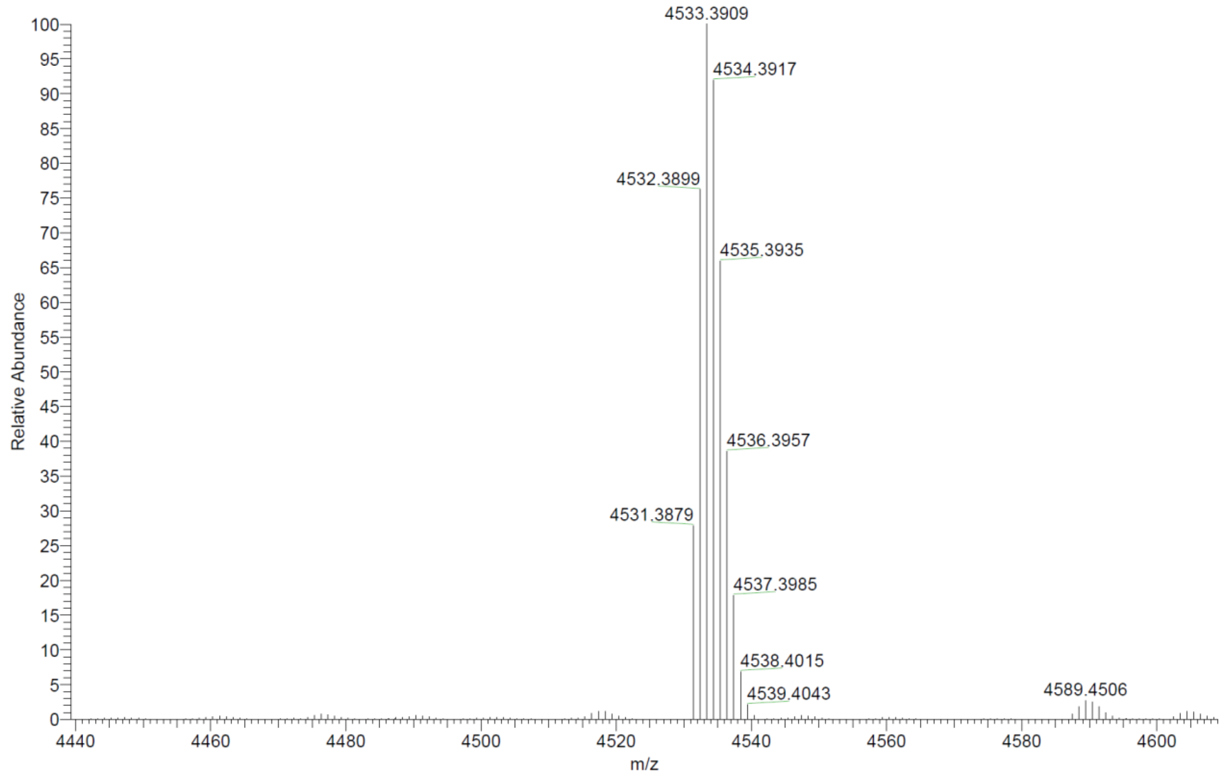
4533.3909
4534.3917
4532.3899
4535.3935
4536.3957
4531.3879
4537.3985
4538.4015
4539.4043
4589.4506
Relative Abundance
m/z

**G3KL** (20% v/v Piperidine, r.t.) was obtained as crude white solid after lyophilization (301.2 mg, 40.5%). Analytical RP-HPLC: $t_R$ = 2.68 min (A/D 100:0 to 0:100 in 7.00 min, λ = 214nm). HRMS (ESI+): $C_{222}H_{432}N_{60}O_{37}$ calc./obs. 4532.38/4532.39 Da $[M+H]^+$.

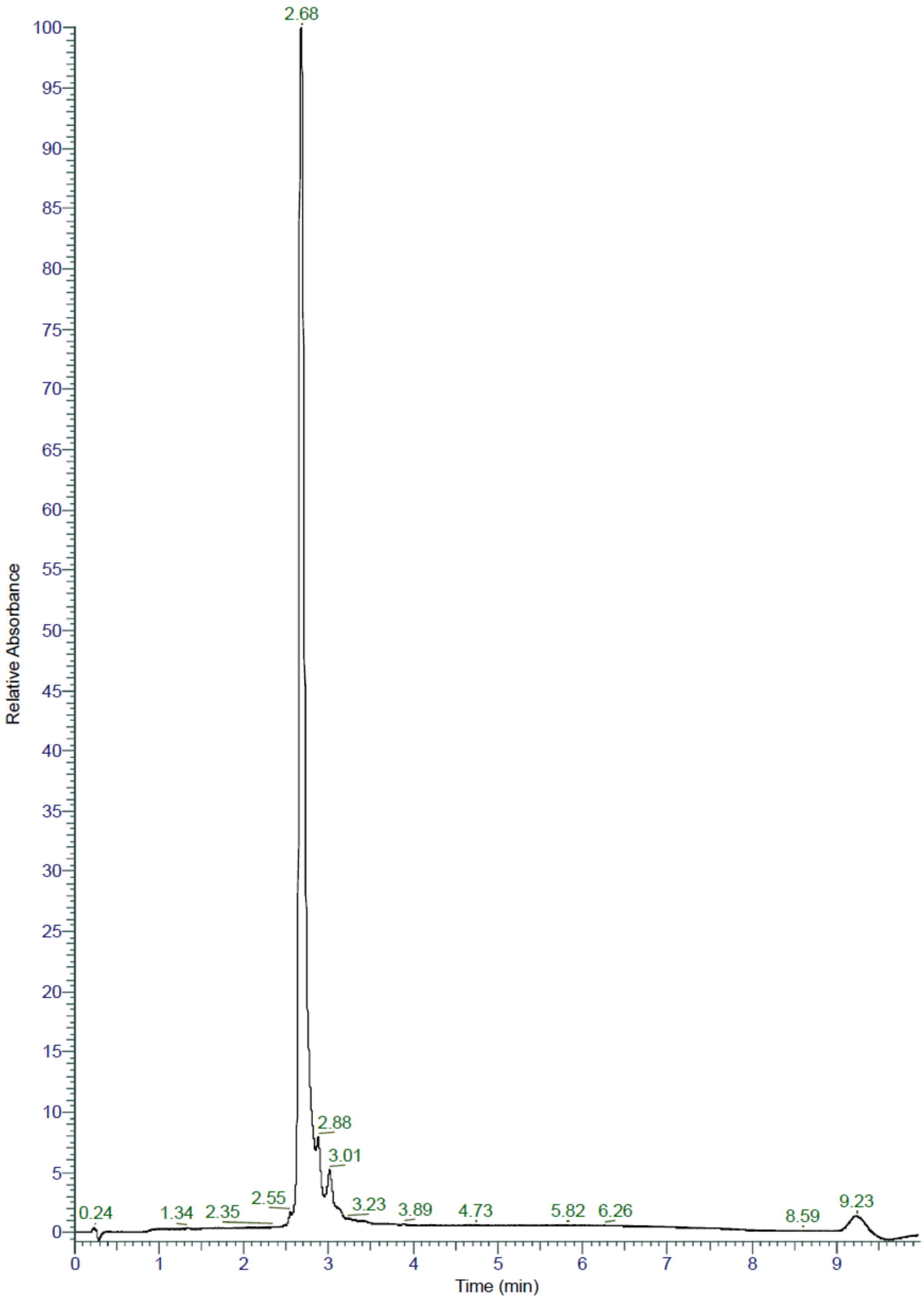

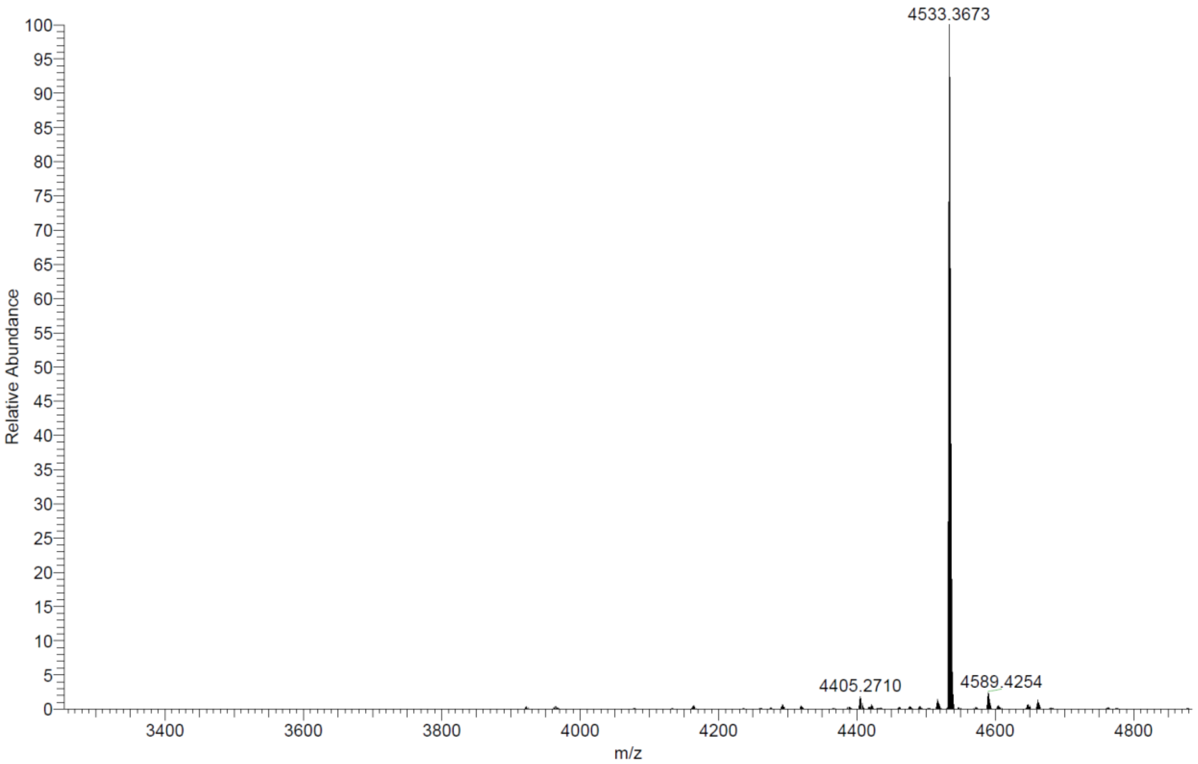

4533.3673
100
95
90
85
80
75
70
65
60
55
50
45
40
35
30
25
20
15
10
5
0
Relative Abundance
4405.2710
4589.4254
3400
3600
3800
4000
4200
4400
4600
4800
m/z


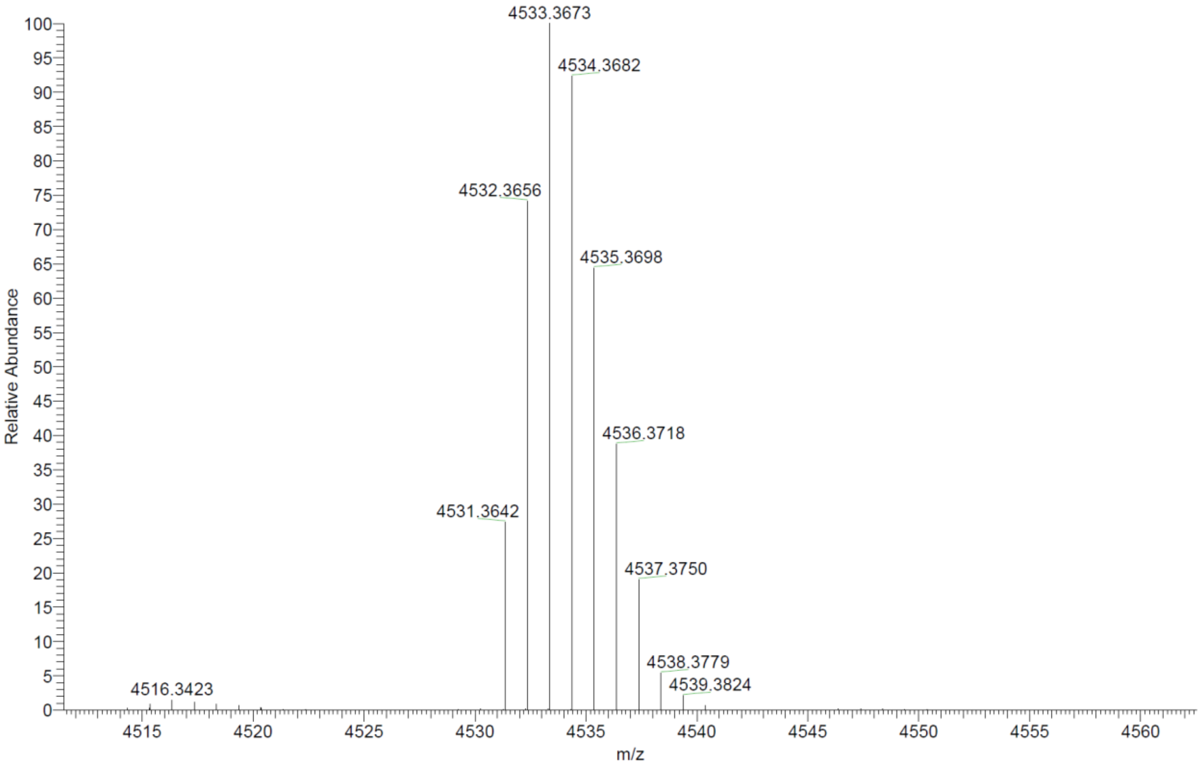

4533.3673
4534.3682
4532.3656
4535.3698
4536.3718
4531.3642
4537.3750
4538.3779
4539.3824
4516.3423
100
95
90
85
80
75
70
65
60
55
50
45
40
35
30
25
20
15
10
5
0
Relative Abundance
4515
4520
4525
4530
4535
4540
4545
4550
4555
4560
m/z

**G3KL** (25% v/v Dipropylamine, r.t.) was obtained as crude white solid after lyophilization (249.7 mg, 12.3%). Analytical RP-HPLC: $t_R$ = 2.71 min (A/D 100:0 to 0:100 in 7.00 min, λ = 214nm). HRMS (ESI+): $C_{222}H_{432}N_{60}O_{37}$ calc./obs. 4532.38/4532.39 Da $[M+H]^+$.

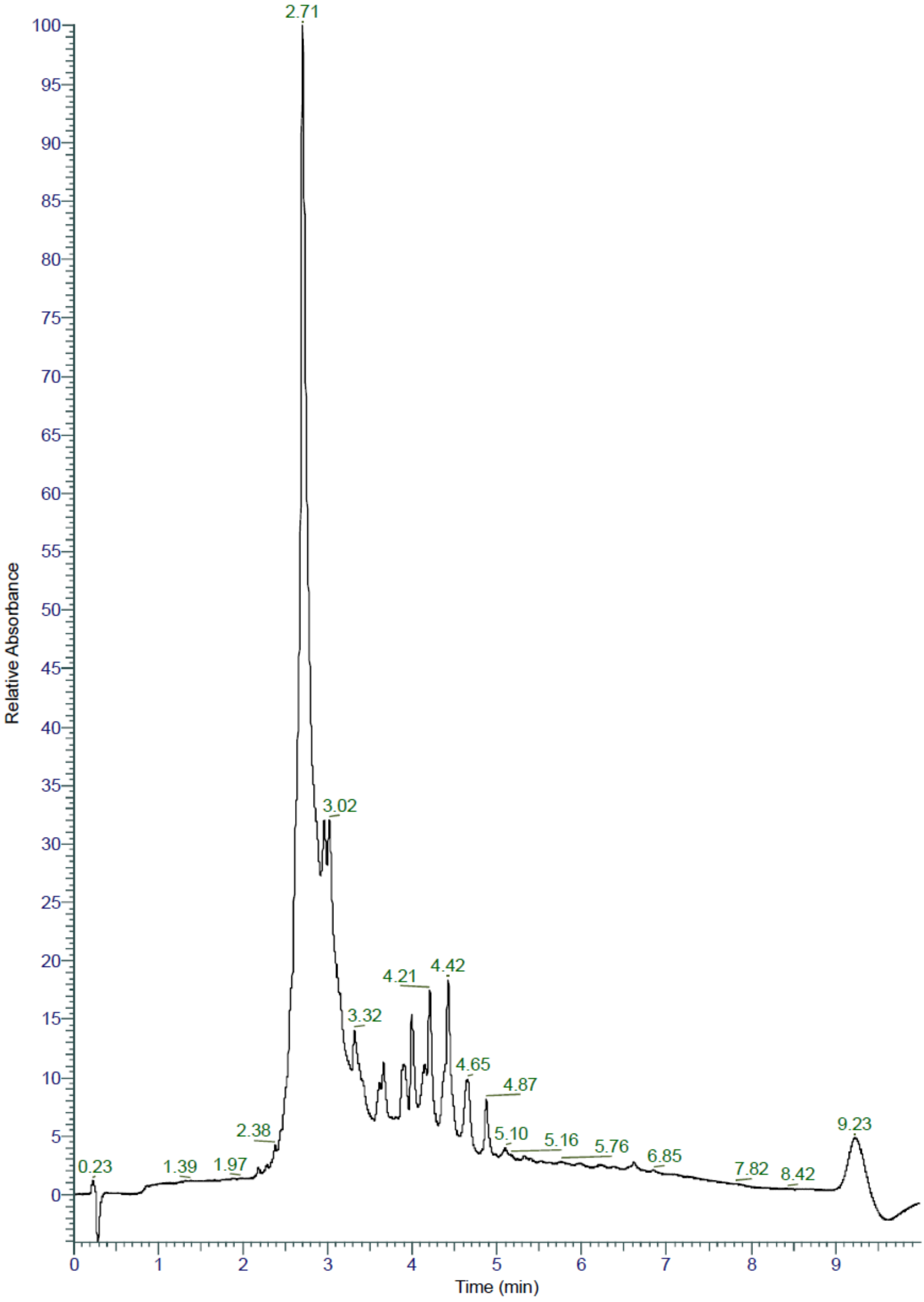

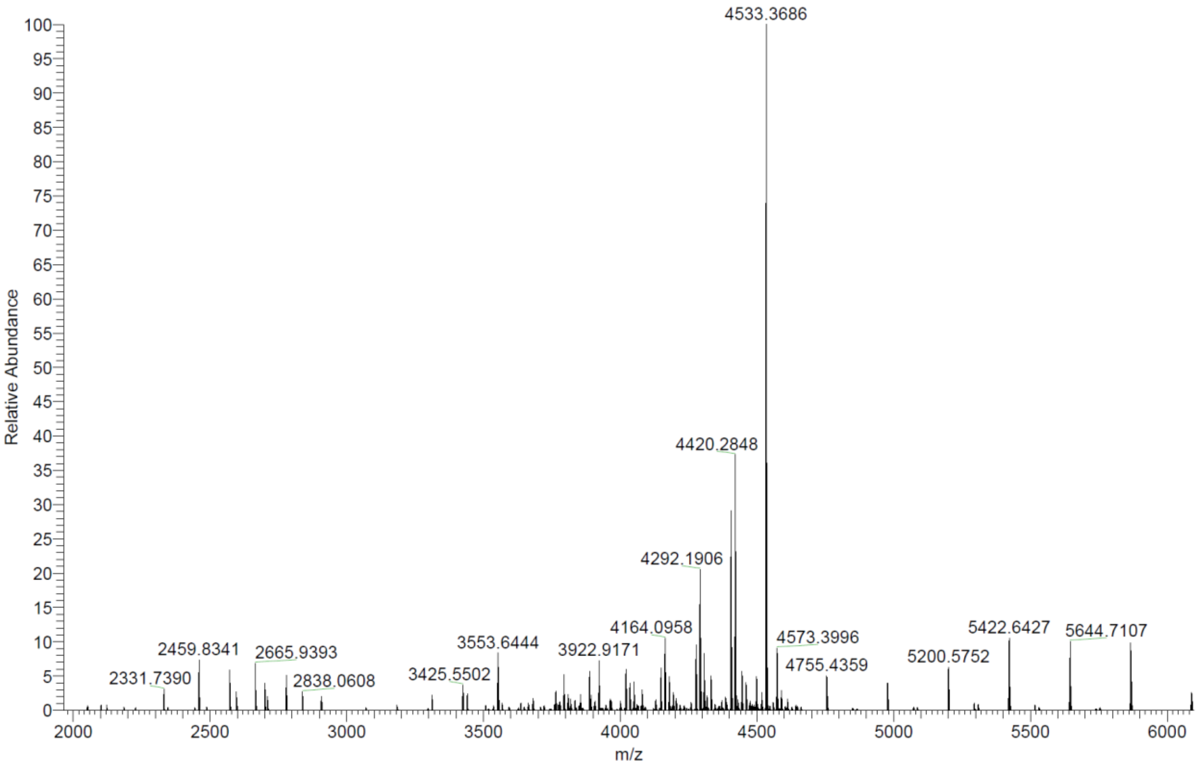
4533.3686
4420.2848
4292.1906
4164.0958
4573.3996
5422.6427
5644.7107
2459.8341
2665.9393
3553.6444
3922.9171
4755.4359
5200.5752
2331.7390
2838.0608
3425.5502
Relative Abundance
m/z

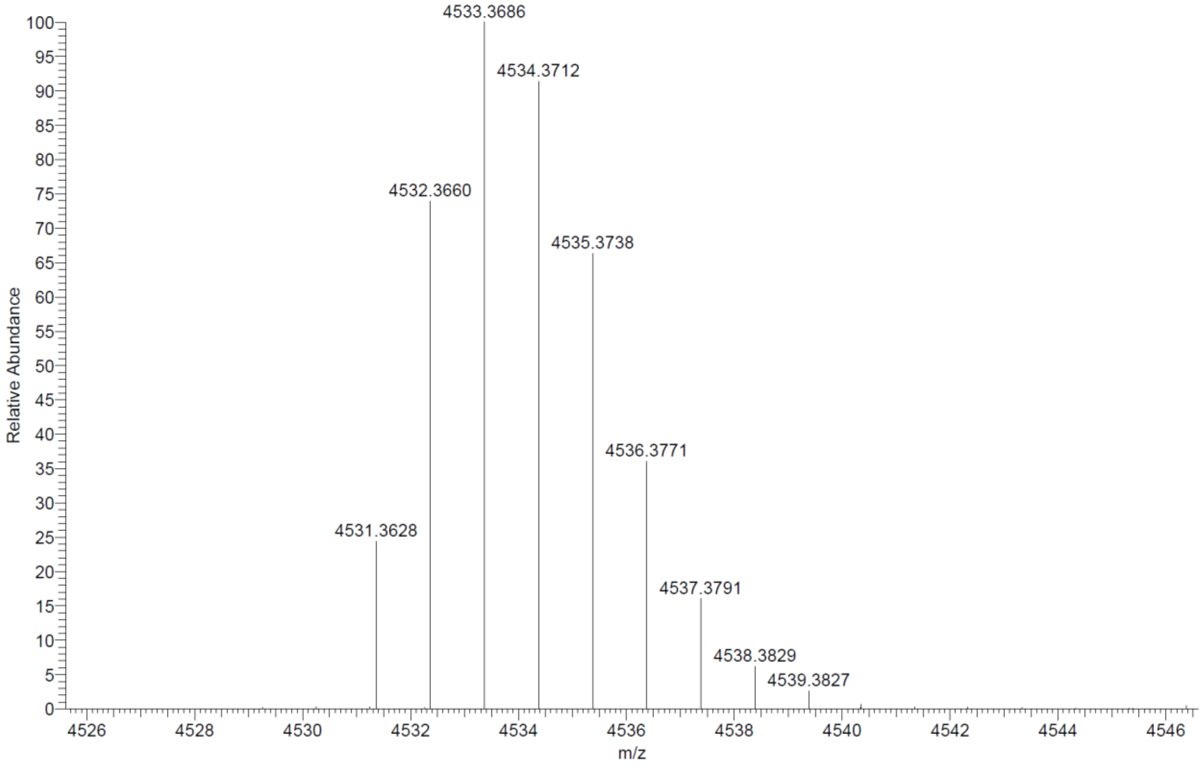
4533.3686
4534.3712
4532.3660
4535.3738
4536.3771
4531.3628
4537.3791
4538.3829
4539.3827
Relative Abundance
m/z
